# Supplementary material for: Altered microRNA Transcriptome in Cultured Human Liver Cells upon Infection with Ebola Virus
Source: Int J Mol Sci. 2021 Apr 6;22(7):3792. doi: 10.3390/ijms22073792 (PMC8038836; doi:10.3390/ijms22073792)
Supplement: Supplementary file 1 [file ijms-22-03792-s001.zip › Supplementary_File/C_ GO_Analysis_Results/16-30nt_go_Reston-24h-Huh7_vs_Control-24h-Huh7_up.mature_mirna_targets/BP_result(Human).html]

| GO.ID | Term | Ontology | Count | Pop.Hits | List.Total | Pop.Total | Fold.Enrichment | Pvalue | FDR | Enrichment.Score | Gene.Ratio | GENES |
| --- | --- | --- | --- | --- | --- | --- | --- | --- | --- | --- | --- | --- |
| GO:0006366 | transcription by RNA polymerase II | Biological process | 131 | 2405 | 574 | 17653 | 1.67518526299014 | 8.47177822358573e-10 | 4.77476624660175e-06 | 9.07202542182569 | 0.228222996515679 | TSHZ1//ZBTB18//CEBPB//ZNF268//TCERG1//STRAP//KLF12//ARX//EDN1//CC2D1B//EN1//ESR1//CPEB3//MYT1L//RCOR1//ZFPM2//GATA2//CNOT7//HOXC8//HSBP1//IFNG//IRF2//AR//JARID2//ZNF568//MAF//MDM4//MYB//NFIA//NR4A2//KLF3//POU4F1//PPARG//DUSP22//ZBTB4//RBBP8//TNF//ZNF217//LRP8//BHLHE41//TBL1XR1//ARID5B//BHLHE40//RUNX3//PHF14//SRSF2//MED6//KLF17//ELK3//EMX2//FOXJ3//FOXF2//EPC2//HBP1//ATAD2//HIVEP2//HOXA3//HOXB6//HOXD1//BOLA3//NR3C2//MYBL1//NEUROG1//NFE2//NHLH2//ARID4B//BRWD1//POU3F2//TRERF1//SLC2A4RG//MIER1//RFX3//RFX4//DMRTA1//RFX7//SMARCD2//ZNF3//ZNF131//FOSL1//TSC22D4//LCOR//RHOXF2//BLZF1//KLF7//CBFB//NRBF2//POLR2K//BMP3//BMPR2//ACVR1//SULF1//VWC2//ZMYND11//MYOG//C5AR1//INTS7//NABP1//ADCYAP1//NCOA7//TFEC//PTF1A//DLL1//MACC1//IGF1//IL2//IRF1//LRP6//MEOX2//MET//NCK1//NEUROD1//NOS1//OTX2//VGLL1//ARMCX3//EGLN1//TET2//PPP3CB//SOX4//WNT7A//YES1//PCGF5//NFATC2IP//TNKS//RPS6KA5//ST18//CARF//EGLN3//NPNT//BTF3//NMI// |
| GO:0006357 | regulation of transcription by RNA polymerase II | Biological process | 123 | 2227 | 574 | 17653 | 1.69860157803579 | 1.4120260968806e-09 | 4.77476624660175e-06 | 8.85015727663655 | 0.214285714285714 | TSHZ1//ZBTB18//CEBPB//ZNF268//TCERG1//STRAP//KLF12//ARX//EDN1//CC2D1B//EN1//ESR1//CPEB3//MYT1L//RCOR1//ZFPM2//GATA2//CNOT7//HOXC8//HSBP1//IFNG//IRF2//AR//JARID2//ZNF568//MAF//MDM4//MYB//NFIA//NR4A2//KLF3//POU4F1//PPARG//DUSP22//ZBTB4//RBBP8//TNF//ZNF217//LRP8//BHLHE41//TBL1XR1//ARID5B//BHLHE40//RUNX3//PHF14//BMP3//BMPR2//ACVR1//SULF1//VWC2//ZMYND11//MED6//ADCYAP1//NCOA7//ELK3//TFEC//FOXJ3//FOXF2//PTF1A//DLL1//ATAD2//MACC1//IGF1//IL2//IRF1//LRP6//MEOX2//MET//MYBL1//MYOG//NCK1//NEUROD1//NEUROG1//NHLH2//NOS1//OTX2//VGLL1//ARMCX3//ARID4B//POU3F2//EGLN1//TET2//PPP3CB//RFX3//RFX4//SOX4//WNT7A//YES1//FOSL1//PCGF5//NFATC2IP//KLF7//CBFB//TNKS//RPS6KA5//ST18//CARF//EGLN3//NPNT//KLF17//EMX2//EPC2//HBP1//HIVEP2//HOXA3//HOXB6//HOXD1//BOLA3//NR3C2//NFE2//BRWD1//TRERF1//SLC2A4RG//MIER1//DMRTA1//RFX7//SMARCD2//ZNF3//ZNF131//TSC22D4//LCOR//RHOXF2//BLZF1// |
| GO:0045944 | positive regulation of transcription by RNA polymerase II | Biological process | 68 | 1132 | 574 | 17653 | 1.84743477672031 | 6.59422748804306e-07 | 0.00114802044627648 | 6.18083607461816 | 0.118466898954704 | ESR1//CARF//CEBPB//NPNT//MED6//ZBTB18//ZNF268//ADCYAP1//NCOA7//EDN1//ELK3//EN1//TFEC//FOXJ3//FOXF2//ZFPM2//PTF1A//GATA2//DLL1//ATAD2//CNOT7//MACC1//IGF1//IL2//IRF1//IRF2//AR//LRP6//MAF//MEOX2//MET//MYB//MYBL1//MYOG//NCK1//NEUROD1//NEUROG1//NFIA//NHLH2//NOS1//NR4A2//OTX2//VGLL1//ARMCX3//ARID4B//POU3F2//POU4F1//EGLN1//PPARG//TET2//PPP3CB//RFX3//RFX4//BMPR2//SOX4//TNF//WNT7A//YES1//TBL1XR1//FOSL1//PCGF5//NFATC2IP//KLF7//CBFB//TNKS//ACVR1//RPS6KA5//ST18// |
| GO:0009893 | positive regulation of metabolic process | Biological process | 155 | 3329 | 574 | 17653 | 1.43193904689337 | 7.70049546414758e-07 | 0.00114802044627648 | 6.11348133061678 | 0.270034843205575 | GADD45A//TNF//CRKL//MAP3K1//MAP3K7CL//LAMTOR3//IGF1//NTF3//PIK3CB//PRKAA1//SOD1//C5AR1//EPS15//EFNA5//FMR1//NPTN//IFNG//EREG//ANP32B//EGLN3//PPARG//CASP3//MDFIC//BNIP3//TRIM8//MTPN//ARX//RAB3GAP1//PTPN22//GJA1//AR//LDLR//MYD88//POU4F1//PTGFR//RHOXF2B//RHOXF2//QKI//TGFBR2//NPNT//CALM2//INHBB//BMP3//BMPR2//ACVR1//EDN1//MTMR9//CPEB3//PRR16//SOX4//RAB12//TSC1//AGTR2//ESR1//FOXF2//ATAD2//IL5//IRF1//LRP6//MYB//MYBL1//NEUROD1//NFE2//NOS1//NPAT//OTX2//PPP3CB//TRERF1//RFX3//WNT7A//TBL1XR1//RUNX3//RAP2C//PAK6//ATR//TNKS//CCT6A//RNF180//RNF125//LONRF3//LONRF1//SOCS5//PPP1R15B//AKAP6//RPS6KA5//IFNA1//MET//PRKAR2B//NCK1//PTGES3//IL2//TNFAIP8L3//MID1IP1//HECW2//CCNY//POLR2K//GPD1//UNG//ADCYAP1//RAPGEF2//MED6//ZBTB18//CEBPB//ZNF268//NCOA7//ELK3//EN1//TFEC//FOXJ3//ZFPM2//PTF1A//GATA2//DLL1//CNOT7//MACC1//IRF2//MAF//MEOX2//MYOG//NEUROG1//NFIA//NHLH2//NR4A2//VGLL1//ARMCX3//ARID4B//POU3F2//EGLN1//TET2//RFX4//YES1//FOSL1//PCGF5//NFATC2IP//KLF7//CBFB//ST18//HPRT1//DUSP22//LRP8//SH3D19//DCUN1D4//ARRDC3//JARID2//CCL2//CNOT6L//YTHDF2//CARF//CCR1//GPR183//CCL8//ACSL1//CALCOCO2//NMI//PIGA// |
| GO:0031325 | positive regulation of cellular metabolic process | Biological process | 145 | 3074 | 574 | 17653 | 1.4506771415423 | 9.68356862152627e-07 | 0.00114802044627648 | 6.0139645655194 | 0.252613240418118 | GADD45A//TNF//CRKL//MAP3K1//MAP3K7CL//LAMTOR3//IGF1//NTF3//PIK3CB//PRKAA1//SOD1//C5AR1//EPS15//EFNA5//FMR1//NPTN//IFNG//EREG//ANP32B//EGLN3//PPARG//CASP3//MDFIC//BNIP3//TRIM8//TGFBR2//NPNT//CALM2//INHBB//BMP3//BMPR2//ACVR1//LDLR//EDN1//MTMR9//CPEB3//PRR16//SOX4//RAB12//TSC1//AGTR2//ESR1//FOXF2//ATAD2//IL5//IRF1//AR//LRP6//MYB//MYBL1//NEUROD1//NFE2//NOS1//NPAT//OTX2//PPP3CB//TRERF1//RFX3//WNT7A//TBL1XR1//RUNX3//RAP2C//PAK6//ATR//TNKS//CCT6A//RNF180//RNF125//LONRF3//LONRF1//SOCS5//PPP1R15B//AKAP6//RPS6KA5//IFNA1//MET//PRKAR2B//NCK1//PTGES3//IL2//TNFAIP8L3//MID1IP1//CCNY//GPD1//UNG//ADCYAP1//RAPGEF2//MED6//ZBTB18//CEBPB//ZNF268//NCOA7//ELK3//EN1//TFEC//FOXJ3//ZFPM2//PTF1A//GATA2//DLL1//CNOT7//MACC1//IRF2//MAF//MEOX2//MYOG//NEUROG1//NFIA//NHLH2//NR4A2//VGLL1//ARMCX3//ARID4B//POU3F2//POU4F1//EGLN1//TET2//RFX4//YES1//FOSL1//PCGF5//NFATC2IP//KLF7//CBFB//ST18//HPRT1//DUSP22//LRP8//SH3D19//DCUN1D4//ARRDC3//JARID2//CNOT6L//YTHDF2//QKI//CARF//CCR1//GPR183//PTPN22//CCL2//CCL8//ACSL1//CALCOCO2//NMI//RAB3GAP1// |
| GO:0031323 | regulation of cellular metabolic process | Biological process | 265 | 6454 | 574 | 17653 | 1.26276792395176 | 1.01850106131286e-06 | 0.00114802044627648 | 5.99203851411391 | 0.461672473867596 | GADD45A//DIRAS3//NPAT//RRM2//TSHZ1//ZBTB18//CEBPB//ZNF268//TCERG1//STRAP//KLF12//ARX//EDN1//CC2D1B//EN1//ESR1//CPEB3//MYT1L//RCOR1//ZFPM2//GATA2//CNOT7//HOXC8//HSBP1//IFNG//IRF2//AR//JARID2//ZNF568//MAF//MDM4//MYB//NFIA//NR4A2//KLF3//POU4F1//PPARG//DUSP22//ZBTB4//RBBP8//TNF//ZNF217//LRP8//BHLHE41//TBL1XR1//ARID5B//BHLHE40//RUNX3//PHF14//CRKL//MAP3K1//MAP3K7CL//LAMTOR3//IGF1//NTF3//PIK3CB//PRKAA1//SOD1//C5AR1//SPRED1//FMR1//MBNL1//SMU1//RBM25//SRSF2//SECISBP2L//EPS15//IL2//LRP6//PPP1R15B//SLIT2//EFNA5//NPTN//EREG//CBL//SOCS5//NLN//ESCO2//BAHD1//ZMYND11//ZNF782//ZNF800//ZFP30//KDM2A//CNOT6L//PTF1A//NRBF2//HIVEP2//ZNF680//MLLT6//COMMD2//VGLL1//SLC2A4RG//PAK6//ZNF630//CNOT6//ZBTB26//RFX3//SOX4//BTF3//BTG1//RHOXF2B//ZNF3//ZNF131//ZNF134//ZNF208//ZNF227//ZNF655//SNIP1//MED28//RPS6KA5//POLR2K//MED6//KLF17//ELK3//EMX2//FOXJ3//FOXF2//EPC2//HBP1//ATAD2//HOXA3//HOXB6//HOXD1//BOLA3//NR3C2//MYBL1//NEUROG1//NFE2//NHLH2//ARID4B//BRWD1//POU3F2//TRERF1//MIER1//RFX4//DMRTA1//RFX7//SMARCD2//FOSL1//TSC22D4//LCOR//RHOXF2//BLZF1//KLF7//CBFB//MTPN//DDX6//TSC1//KBTBD8//EIF3B//FLRT1//NCK1//DBNDD2//ANP32B//EGLN3//CASP3//G3BP2//MDFIC//ATR//DRAM1//RRAGD//ULK2//PTPN22//MET//MTMR9//BNIP3//TRIM8//TGFBR2//NPNT//CALM2//INHBB//BMP3//BMPR2//ACVR1//LDLR//DLG2//PLEKHF2//A2ML1//WFDC13//SPOCK1//PRR16//RAB12//SNX5//AGTR2//IL5//IRF1//NEUROD1//NOS1//OTX2//PPP3CB//WNT7A//RASD1//ATXN1//BTAF1//WRNIP1//SULF1//VWC2//EGLN1//SPOPL//CDK6//RAP2C//TNKS//DCP2//CCT6A//MTM1//RNF180//RNF125//LONRF3//LONRF1//PHACTR2//AKAP6//IFNA1//PRKAR2B//PTGES3//ITM2A//ARL6IP1//FZD6//FASTK//YTHDF2//TNFAIP8L3//UBXN2B//NMI//MID1IP1//CCNY//MYOG//GPD1//UNG//ADCYAP1//RAPGEF2//NCOA7//TFEC//DLL1//MACC1//MEOX2//ARMCX3//TET2//YES1//PCGF5//NFATC2IP//ST18//HPRT1//GRIK2//SAMSN1//SH3D19//IL1RAP//MYD88//DCUN1D4//ARRDC3//PMEPA1//LDLRAD4//QKI//CARF//CCR1//GPR183//CCL2//CCL8//ACSL1//PLAA//CPEB2//CALCOCO2//TMED10//RAB3GAP1//GJA1//CPEB1// |
| GO:0051173 | positive regulation of nitrogen compound metabolic process | Biological process | 140 | 2965 | 574 | 17653 | 1.45214494303459 | 1.52213761273111e-06 | 0.00147060238212864 | 5.81754608229074 | 0.24390243902439 | GADD45A//TNF//CRKL//MAP3K1//MAP3K7CL//LAMTOR3//IGF1//NTF3//PIK3CB//PRKAA1//SOD1//C5AR1//EFNA5//FMR1//NPTN//IFNG//EREG//ANP32B//EGLN3//PPARG//CASP3//MDFIC//CALM2//INHBB//BMP3//BMPR2//ACVR1//CPEB3//PRR16//SOX4//AGTR2//ESR1//FOXF2//ATAD2//IL5//IRF1//AR//LRP6//MYB//MYBL1//NEUROD1//NFE2//NOS1//NPAT//OTX2//PPP3CB//TRERF1//RFX3//WNT7A//TBL1XR1//RUNX3//RAP2C//PAK6//TGFBR2//ATR//TNKS//CCT6A//RNF180//RNF125//LONRF3//LONRF1//SOCS5//PPP1R15B//AKAP6//RPS6KA5//IFNA1//PRKAR2B//NCK1//IL2//EDN1//MET//GJA1//HECW2//CCNY//GPD1//UNG//ADCYAP1//RAPGEF2//MED6//ZBTB18//CEBPB//ZNF268//NCOA7//ELK3//EN1//TFEC//FOXJ3//ZFPM2//PTF1A//GATA2//DLL1//CNOT7//MACC1//IRF2//MAF//MEOX2//MYOG//NEUROG1//NFIA//NHLH2//NR4A2//VGLL1//ARMCX3//ARID4B//POU3F2//POU4F1//EGLN1//TET2//RFX4//YES1//FOSL1//PCGF5//NFATC2IP//KLF7//CBFB//ST18//HPRT1//DUSP22//LRP8//SH3D19//MTPN//DCUN1D4//ARRDC3//JARID2//PTGES3//CNOT6L//YTHDF2//QKI//CARF//CCR1//GPR183//NPNT//PTPN22//TNFAIP8L3//CCL2//CCL8//ACSL1//NMI//RAB3GAP1//LDLR// |
| GO:0010604 | positive regulation of macromolecule metabolic process | Biological process | 145 | 3106 | 574 | 17653 | 1.43573133712204 | 1.76504962602803e-06 | 0.00149212882760345 | 5.75324307950724 | 0.252613240418118 | GADD45A//TNF//CRKL//MAP3K1//MAP3K7CL//LAMTOR3//IGF1//NTF3//PIK3CB//PRKAA1//SOD1//C5AR1//EPS15//EFNA5//FMR1//NPTN//IFNG//EREG//ANP32B//EGLN3//PPARG//CASP3//MDFIC//MTPN//ARX//RAB3GAP1//PTPN22//GJA1//AR//LDLR//MYD88//POU4F1//PTGFR//RHOXF2B//RHOXF2//QKI//CALM2//INHBB//BMP3//BMPR2//ACVR1//EDN1//CPEB3//PRR16//SOX4//AGTR2//ESR1//FOXF2//ATAD2//IL5//IRF1//LRP6//MYB//MYBL1//NEUROD1//NFE2//NOS1//NPAT//OTX2//PPP3CB//TRERF1//RFX3//WNT7A//TBL1XR1//RUNX3//RAP2C//PAK6//TGFBR2//ATR//TNKS//CCT6A//RNF180//RNF125//LONRF3//LONRF1//SOCS5//PPP1R15B//AKAP6//RPS6KA5//IFNA1//PRKAR2B//NCK1//IL2//MET//HECW2//CCNY//POLR2K//UNG//ADCYAP1//RAPGEF2//MED6//ZBTB18//CEBPB//ZNF268//NCOA7//ELK3//EN1//TFEC//FOXJ3//ZFPM2//PTF1A//GATA2//DLL1//CNOT7//MACC1//IRF2//MAF//MEOX2//MYOG//NEUROG1//NFIA//NHLH2//NR4A2//VGLL1//ARMCX3//ARID4B//POU3F2//EGLN1//TET2//RFX4//YES1//FOSL1//PCGF5//NFATC2IP//KLF7//CBFB//ST18//DUSP22//LRP8//SH3D19//DCUN1D4//ARRDC3//JARID2//CCL2//PTGES3//CNOT6L//YTHDF2//CARF//CCR1//GPR183//NPNT//TNFAIP8L3//CCL8//ACSL1//NMI// |
| GO:0048468 | cell development | Biological process | 103 | 2045 | 574 | 17653 | 1.54899687348253 | 3.50367979351667e-06 | 0.00263282071595036 | 5.45547559150602 | 0.179442508710801 | EREG//YTHDF2//AGFG1//ACVR1//MET//FZD3//SULF1//BMPR2//ESR1//GJA1//CDK6//DLL1//AR//FRMD6//TGFBR2//TDRD5//SPO11//STRBP//QKI//OGN//POU4F1//WNT7A//SLIT2//ARX//DOK1//DPYSL2//EFNA5//OTX2//PIK3CB//ENAH//KLF7//RPS6KA5//NRXN1//CNTNAP2//GSTM3//FRZB//SNAP25//STMN2//ADCYAP1//NPTN//NCK1//RAPGEF2//PTPRG//SPOCK1//TSC1//POU3F2//EDN1//KBTBD8//IGF1//FLRT1//SOX4//NR4A2//GATA2//HPRT1//KCNJ10//CASP3//TMOD1//UBE4B//LRP12//DBNDD2//PSD//SOD1//SYCP1//SYCP3//IL5//NTF3//DDX6//VWC2//NEUROD1//NEUROG1//PPARG//RFX3//CBFB//PAQR5//INHBB//EN1//MYT1L//FMR1//ULK2//PPP3CB//MYOG//NYAP1//HECW2//RUNX3//NOS1//MYB//CPEB3//IL2//DLG5//LRP8//AGTR2//AKAP6//LDLR//RAP2C//ARID4B//IL1RAP//CRKL//TNF//PLAA//GPR183//MAF//BMP3//ARID5B// |
| GO:0051252 | regulation of RNA metabolic process | Biological process | 175 | 3984 | 574 | 17653 | 1.35090667548242 | 4.99220423813456e-06 | 0.00322329110107229 | 5.30170765536081 | 0.304878048780488 | NPAT//RRM2//TSHZ1//ZBTB18//CEBPB//ZNF268//TCERG1//STRAP//KLF12//ARX//EDN1//CC2D1B//EN1//ESR1//CPEB3//MYT1L//RCOR1//ZFPM2//GATA2//CNOT7//HOXC8//HSBP1//IFNG//IRF2//AR//JARID2//ZNF568//MAF//MDM4//MYB//NFIA//NR4A2//KLF3//POU4F1//PPARG//DUSP22//ZBTB4//RBBP8//TNF//ZNF217//LRP8//BHLHE41//TBL1XR1//ARID5B//BHLHE40//RUNX3//PHF14//FMR1//MBNL1//SMU1//RBM25//SRSF2//BAHD1//ZMYND11//ZNF782//ZNF800//ZFP30//KDM2A//CNOT6L//PTF1A//NRBF2//HIVEP2//ZNF680//MLLT6//COMMD2//VGLL1//SLC2A4RG//PAK6//ZNF630//CNOT6//ZBTB26//RFX3//SOX4//BTF3//BTG1//RHOXF2B//ZNF3//ZNF131//ZNF134//ZNF208//ZNF227//ZNF655//SNIP1//MED28//CBL//RPS6KA5//POLR2K//MED6//KLF17//ELK3//EMX2//FOXJ3//FOXF2//EPC2//HBP1//ATAD2//HOXA3//HOXB6//HOXD1//BOLA3//NR3C2//MYBL1//NEUROG1//NFE2//NHLH2//ARID4B//BRWD1//POU3F2//TRERF1//MIER1//RFX4//DMRTA1//RFX7//SMARCD2//FOSL1//TSC22D4//LCOR//RHOXF2//BLZF1//KLF7//CBFB//G3BP2//AGTR2//MDFIC//IGF1//IL5//IRF1//LRP6//NEUROD1//NOS1//OTX2//PPP3CB//WNT7A//ACVR1//EREG//RASD1//ATXN1//BTAF1//BMP3//BMPR2//SULF1//VWC2//EGLN1//FZD6//FASTK//DCP2//YTHDF2//ADCYAP1//NCOA7//TFEC//DLL1//MACC1//IL2//MEOX2//MET//MYOG//NCK1//ARMCX3//TET2//YES1//PCGF5//NFATC2IP//TNKS//ST18//TRIM8//MTPN//IL1RAP//MYD88//QKI//CARF//EGLN3//PRKAA1//RAP2C//NMI//NPNT// |
| GO:1903506 | regulation of nucleic acid-templated transcription | Biological process | 167 | 3766 | 574 | 17653 | 1.36377518638247 | 5.24267368206346e-06 | 0.00322329110107229 | 5.28044717308726 | 0.290940766550523 | NPAT//RRM2//TSHZ1//ZBTB18//CEBPB//ZNF268//TCERG1//STRAP//KLF12//ARX//EDN1//CC2D1B//EN1//ESR1//CPEB3//MYT1L//RCOR1//ZFPM2//GATA2//CNOT7//HOXC8//HSBP1//IFNG//IRF2//AR//JARID2//ZNF568//MAF//MDM4//MYB//NFIA//NR4A2//KLF3//POU4F1//PPARG//DUSP22//ZBTB4//RBBP8//TNF//ZNF217//LRP8//BHLHE41//TBL1XR1//ARID5B//BHLHE40//RUNX3//PHF14//BAHD1//ZMYND11//ZNF782//ZNF800//ZFP30//KDM2A//CNOT6L//PTF1A//NRBF2//HIVEP2//ZNF680//MLLT6//COMMD2//VGLL1//SLC2A4RG//PAK6//ZNF630//CNOT6//ZBTB26//RFX3//SOX4//BTF3//BTG1//RHOXF2B//ZNF3//ZNF131//ZNF134//ZNF208//ZNF227//ZNF655//SNIP1//MED28//CBL//RPS6KA5//POLR2K//MED6//KLF17//ELK3//EMX2//FOXJ3//FOXF2//EPC2//HBP1//ATAD2//HOXA3//HOXB6//HOXD1//BOLA3//NR3C2//MYBL1//NEUROG1//NFE2//NHLH2//ARID4B//BRWD1//POU3F2//TRERF1//MIER1//RFX4//DMRTA1//RFX7//SMARCD2//FOSL1//TSC22D4//LCOR//RHOXF2//BLZF1//KLF7//CBFB//G3BP2//AGTR2//MDFIC//IGF1//IL5//IRF1//LRP6//NEUROD1//NOS1//OTX2//PPP3CB//WNT7A//ACVR1//EREG//RASD1//ATXN1//BTAF1//BMP3//BMPR2//SULF1//VWC2//EGLN1//FZD6//ADCYAP1//NCOA7//TFEC//DLL1//MACC1//IL2//MEOX2//MET//MYOG//NCK1//ARMCX3//TET2//YES1//PCGF5//NFATC2IP//TNKS//ST18//TRIM8//MTPN//IL1RAP//MYD88//CARF//EGLN3//PRKAA1//SRSF2//NPNT//RAP2C//NMI// |
| GO:2001141 | regulation of RNA biosynthetic process | Biological process | 167 | 3774 | 574 | 17653 | 1.36088430098473 | 5.96556771098229e-06 | 0.0033620945357811 | 5.22434822055777 | 0.290940766550523 | NPAT//RRM2//TSHZ1//ZBTB18//CEBPB//ZNF268//TCERG1//STRAP//KLF12//ARX//EDN1//CC2D1B//EN1//ESR1//CPEB3//MYT1L//RCOR1//ZFPM2//GATA2//CNOT7//HOXC8//HSBP1//IFNG//IRF2//AR//JARID2//ZNF568//MAF//MDM4//MYB//NFIA//NR4A2//KLF3//POU4F1//PPARG//DUSP22//ZBTB4//RBBP8//TNF//ZNF217//LRP8//BHLHE41//TBL1XR1//ARID5B//BHLHE40//RUNX3//PHF14//BAHD1//ZMYND11//ZNF782//ZNF800//ZFP30//KDM2A//CNOT6L//PTF1A//NRBF2//HIVEP2//ZNF680//MLLT6//COMMD2//VGLL1//SLC2A4RG//PAK6//ZNF630//CNOT6//ZBTB26//RFX3//SOX4//BTF3//BTG1//RHOXF2B//ZNF3//ZNF131//ZNF134//ZNF208//ZNF227//ZNF655//SNIP1//MED28//CBL//RPS6KA5//POLR2K//MED6//KLF17//ELK3//EMX2//FOXJ3//FOXF2//EPC2//HBP1//ATAD2//HOXA3//HOXB6//HOXD1//BOLA3//NR3C2//MYBL1//NEUROG1//NFE2//NHLH2//ARID4B//BRWD1//POU3F2//TRERF1//MIER1//RFX4//DMRTA1//RFX7//SMARCD2//FOSL1//TSC22D4//LCOR//RHOXF2//BLZF1//KLF7//CBFB//G3BP2//AGTR2//MDFIC//IGF1//IL5//IRF1//LRP6//NEUROD1//NOS1//OTX2//PPP3CB//WNT7A//ACVR1//EREG//RASD1//ATXN1//BTAF1//BMP3//BMPR2//SULF1//VWC2//EGLN1//FZD6//ADCYAP1//NCOA7//TFEC//DLL1//MACC1//IL2//MEOX2//MET//MYOG//NCK1//ARMCX3//TET2//YES1//PCGF5//NFATC2IP//TNKS//ST18//TRIM8//MTPN//IL1RAP//MYD88//CARF//EGLN3//PRKAA1//RAP2C//NMI//SRSF2//NPNT// |
| GO:0051171 | regulation of nitrogen compound metabolic process | Biological process | 253 | 6240 | 574 | 17653 | 1.24693139685518 | 7.06727967232935e-06 | 0.00367661634030488 | 5.15074772207907 | 0.440766550522648 | GADD45A//DIRAS3//NPAT//RRM2//TSHZ1//ZBTB18//CEBPB//ZNF268//TCERG1//STRAP//KLF12//ARX//EDN1//CC2D1B//EN1//ESR1//CPEB3//MYT1L//RCOR1//ZFPM2//GATA2//CNOT7//HOXC8//HSBP1//IFNG//IRF2//AR//JARID2//ZNF568//MAF//MDM4//MYB//NFIA//NR4A2//KLF3//POU4F1//PPARG//DUSP22//ZBTB4//RBBP8//TNF//ZNF217//LRP8//BHLHE41//TBL1XR1//ARID5B//BHLHE40//RUNX3//PHF14//CRKL//MAP3K1//MAP3K7CL//LAMTOR3//IGF1//NTF3//PIK3CB//PRKAA1//SOD1//C5AR1//SPRED1//FMR1//MBNL1//SMU1//RBM25//SRSF2//SECISBP2L//IL2//LRP6//PPP1R15B//SLIT2//EFNA5//NPTN//EREG//CBL//SOCS5//ESCO2//BAHD1//ZMYND11//ZNF782//ZNF800//ZFP30//KDM2A//CNOT6L//PTF1A//NRBF2//HIVEP2//ZNF680//MLLT6//COMMD2//VGLL1//SLC2A4RG//PAK6//ZNF630//CNOT6//ZBTB26//RFX3//SOX4//BTF3//BTG1//RHOXF2B//ZNF3//ZNF131//ZNF134//ZNF208//ZNF227//ZNF655//SNIP1//MED28//RPS6KA5//POLR2K//MED6//KLF17//ELK3//EMX2//FOXJ3//FOXF2//EPC2//HBP1//ATAD2//HOXA3//HOXB6//HOXD1//BOLA3//NR3C2//MYBL1//NEUROG1//NFE2//NHLH2//ARID4B//BRWD1//POU3F2//TRERF1//MIER1//RFX4//DMRTA1//RFX7//SMARCD2//FOSL1//TSC22D4//LCOR//RHOXF2//BLZF1//KLF7//CBFB//MTPN//DDX6//TSC1//KBTBD8//EIF3B//FLRT1//NCK1//DBNDD2//ANP32B//EGLN3//CASP3//G3BP2//MDFIC//ATR//CALM2//INHBB//BMP3//BMPR2//ACVR1//LDLR//A2ML1//WFDC13//SPOCK1//PRR16//AGTR2//IL5//IRF1//NEUROD1//NOS1//OTX2//PPP3CB//WNT7A//RASD1//ATXN1//BTAF1//SPOPL//WRNIP1//SULF1//VWC2//EGLN1//CDK6//RAP2C//TGFBR2//TNKS//DCP2//CCT6A//MTM1//RNF180//RNF125//LONRF3//LONRF1//PHACTR2//AKAP6//IFNA1//PRKAR2B//ITM2A//ARL6IP1//MET//FZD6//FASTK//YTHDF2//PTPN22//UBXN2B//NMI//GJA1//HECW2//CCNY//MYOG//GPD1//UNG//ADCYAP1//RAPGEF2//NCOA7//TFEC//DLL1//MACC1//MEOX2//ARMCX3//TET2//YES1//PCGF5//NFATC2IP//ST18//HPRT1//GRIK2//SAMSN1//SH3D19//TRIM8//IL1RAP//MYD88//DCUN1D4//ARRDC3//PTGES3//PMEPA1//LDLRAD4//QKI//CARF//CCR1//GPR183//NPNT//TNFAIP8L3//CCL2//CCL8//ACSL1//PLAA//CPEB2//TMED10//RAB3GAP1//CPEB1// |
| GO:0051254 | positive regulation of RNA metabolic process | Biological process | 80 | 1523 | 574 | 17653 | 1.61546187265643 | 1.18138099326536e-05 | 0.00546914895625361 | 4.92761002060061 | 0.139372822299652 | AGTR2//ESR1//FOXF2//ATAD2//MDFIC//IGF1//IL5//IRF1//AR//LRP6//MYB//MYBL1//NEUROD1//NFE2//NOS1//NPAT//OTX2//PPARG//PPP3CB//TRERF1//RFX3//SOX4//TNF//WNT7A//TBL1XR1//RUNX3//ACVR1//MED6//ZBTB18//CEBPB//ZNF268//ADCYAP1//NCOA7//EDN1//ELK3//EN1//TFEC//FOXJ3//ZFPM2//PTF1A//GATA2//DLL1//CNOT7//MACC1//IL2//IRF2//MAF//MEOX2//MET//MYOG//NCK1//NEUROG1//NFIA//NHLH2//NR4A2//VGLL1//ARMCX3//ARID4B//POU3F2//POU4F1//EGLN1//TET2//RFX4//BMPR2//YES1//FOSL1//PCGF5//NFATC2IP//KLF7//CBFB//TNKS//RPS6KA5//ST18//CPEB3//CNOT6L//YTHDF2//QKI//CARF//PRKAA1//NPNT// |
| GO:0010557 | positive regulation of macromolecule biosynthetic process | Biological process | 87 | 1698 | 574 | 17653 | 1.5757531919085 | 1.2130302283573e-05 | 0.00546914895625361 | 4.91612837650754 | 0.151567944250871 | IGF1//EDN1//CPEB3//FMR1//PRR16//SOX4//AGTR2//ESR1//FOXF2//ATAD2//MDFIC//IL5//IRF1//AR//LRP6//MYB//MYBL1//NEUROD1//NFE2//NOS1//NPAT//OTX2//PPARG//PPP3CB//TRERF1//RFX3//TNF//WNT7A//TBL1XR1//RUNX3//ACVR1//ATR//TNKS//CCT6A//NCK1//EREG//MED6//ZBTB18//CEBPB//ZNF268//ADCYAP1//NCOA7//ELK3//EN1//TFEC//FOXJ3//ZFPM2//PTF1A//GATA2//DLL1//CNOT7//MACC1//IL2//IRF2//MAF//MEOX2//MET//MYOG//NEUROG1//NFIA//NHLH2//NR4A2//VGLL1//ARMCX3//ARID4B//POU3F2//POU4F1//EGLN1//TET2//RFX4//BMPR2//YES1//FOSL1//PCGF5//NFATC2IP//KLF7//CBFB//RPS6KA5//ST18//CCL2//PTGES3//CARF//RAB3GAP1//PRKAA1//YTHDF2//NPNT//MTPN// |
| GO:0010556 | regulation of macromolecule biosynthetic process | Biological process | 190 | 4467 | 574 | 17653 | 1.30811003495241 | 1.30159048318839e-05 | 0.00550166027362693 | 4.88552563548137 | 0.331010452961672 | NPAT//RRM2//TSHZ1//ZBTB18//CEBPB//ZNF268//TCERG1//STRAP//KLF12//ARX//EDN1//CC2D1B//EN1//ESR1//CPEB3//MYT1L//RCOR1//ZFPM2//GATA2//CNOT7//HOXC8//HSBP1//IFNG//IRF2//AR//JARID2//ZNF568//MAF//MDM4//MYB//NFIA//NR4A2//KLF3//POU4F1//PPARG//DUSP22//ZBTB4//RBBP8//TNF//ZNF217//LRP8//BHLHE41//TBL1XR1//ARID5B//BHLHE40//RUNX3//PHF14//SECISBP2L//ESCO2//BAHD1//ZMYND11//ZNF782//ZNF800//ZFP30//KDM2A//CNOT6L//PTF1A//NRBF2//HIVEP2//ZNF680//MLLT6//COMMD2//VGLL1//SLC2A4RG//PAK6//ZNF630//CNOT6//ZBTB26//RFX3//SOX4//BTF3//BTG1//RHOXF2B//ZNF3//ZNF131//ZNF134//ZNF208//ZNF227//ZNF655//SNIP1//MED28//CBL//RPS6KA5//POLR2K//MED6//KLF17//ELK3//EMX2//FOXJ3//FOXF2//EPC2//HBP1//ATAD2//HOXA3//HOXB6//HOXD1//BOLA3//NR3C2//MYBL1//NEUROG1//NFE2//NHLH2//ARID4B//BRWD1//POU3F2//TRERF1//MIER1//RFX4//DMRTA1//RFX7//SMARCD2//FOSL1//TSC22D4//LCOR//RHOXF2//BLZF1//KLF7//CBFB//MTPN//DDX6//TSC1//KBTBD8//EIF3B//G3BP2//ATR//IGF1//FMR1//PRR16//AGTR2//MDFIC//IL5//IRF1//LRP6//NEUROD1//NOS1//OTX2//PPP3CB//WNT7A//ACVR1//EREG//RASD1//ATXN1//BTAF1//WRNIP1//BMP3//BMPR2//SULF1//VWC2//TNKS//DCP2//CCT6A//NCK1//ITM2A//EGLN1//FZD6//NMI//ADCYAP1//NCOA7//TFEC//DLL1//MACC1//IL2//MEOX2//MET//MYOG//ARMCX3//TET2//YES1//PCGF5//NFATC2IP//ST18//PRKAA1//INHBB//TRIM8//IL1RAP//MYD88//CCL2//PTGES3//YTHDF2//QKI//CARF//EGLN3//CPEB2//RAB3GAP1//RAP2C//SRSF2//PPP1R15B//GJA1//NPNT//CPEB1// |
| GO:0006355 | regulation of transcription, DNA-templated | Biological process | 164 | 3745 | 574 | 17653 | 1.34678619111196 | 1.39488697462556e-05 | 0.00554660311157933 | 4.8554609811252 | 0.285714285714286 | NPAT//RRM2//TSHZ1//ZBTB18//CEBPB//ZNF268//TCERG1//STRAP//KLF12//ARX//EDN1//CC2D1B//EN1//ESR1//CPEB3//MYT1L//RCOR1//ZFPM2//GATA2//CNOT7//HOXC8//HSBP1//IFNG//IRF2//AR//JARID2//ZNF568//MAF//MDM4//MYB//NFIA//NR4A2//KLF3//POU4F1//PPARG//DUSP22//ZBTB4//RBBP8//TNF//ZNF217//LRP8//BHLHE41//TBL1XR1//ARID5B//BHLHE40//RUNX3//PHF14//BAHD1//POLR2K//MED6//KLF17//ELK3//EMX2//FOXJ3//FOXF2//EPC2//HBP1//ATAD2//HIVEP2//HOXA3//HOXB6//HOXD1//BOLA3//NR3C2//MYBL1//NEUROG1//NFE2//NHLH2//ARID4B//BRWD1//POU3F2//TRERF1//SLC2A4RG//MIER1//RFX3//RFX4//DMRTA1//RFX7//SMARCD2//ZNF3//ZNF131//FOSL1//TSC22D4//LCOR//RHOXF2//BLZF1//KLF7//CBFB//G3BP2//AGTR2//MDFIC//IGF1//IL5//IRF1//LRP6//NEUROD1//NOS1//OTX2//PPP3CB//SOX4//WNT7A//ACVR1//EREG//RASD1//ATXN1//BTAF1//RPS6KA5//BMP3//BMPR2//SULF1//VWC2//ZMYND11//EGLN1//FZD6//ADCYAP1//NCOA7//TFEC//PTF1A//DLL1//MACC1//IL2//MEOX2//MET//MYOG//NCK1//VGLL1//ARMCX3//TET2//YES1//PCGF5//NFATC2IP//TNKS//ST18//TRIM8//MTPN//IL1RAP//MYD88//CARF//EGLN3//PRKAA1//NPNT//ZNF782//ZNF800//ZFP30//KDM2A//CNOT6L//NRBF2//ZNF680//MLLT6//COMMD2//PAK6//ZNF630//CNOT6//ZBTB26//BTF3//BTG1//RHOXF2B//ZNF134//ZNF208//ZNF227//ZNF655//SNIP1//MED28//CBL// |
| GO:0080090 | regulation of primary metabolic process | Biological process | 256 | 6394 | 574 | 17653 | 1.23132858657779 | 1.66130682631339e-05 | 0.00554660311157933 | 4.77955015040904 | 0.445993031358885 | GADD45A//DIRAS3//NPAT//RRM2//TSHZ1//ZBTB18//CEBPB//ZNF268//TCERG1//STRAP//KLF12//ARX//EDN1//CC2D1B//EN1//ESR1//CPEB3//MYT1L//RCOR1//ZFPM2//GATA2//CNOT7//HOXC8//HSBP1//IFNG//IRF2//AR//JARID2//ZNF568//MAF//MDM4//MYB//NFIA//NR4A2//KLF3//POU4F1//PPARG//DUSP22//ZBTB4//RBBP8//TNF//ZNF217//LRP8//BHLHE41//TBL1XR1//ARID5B//BHLHE40//RUNX3//PHF14//CRKL//MAP3K1//MAP3K7CL//LAMTOR3//IGF1//NTF3//PIK3CB//PRKAA1//SOD1//C5AR1//SPRED1//FMR1//MBNL1//SMU1//RBM25//SRSF2//SECISBP2L//IL2//LRP6//PPP1R15B//SLIT2//EFNA5//NPTN//EREG//CBL//SOCS5//NLN//ESCO2//BAHD1//ZMYND11//ZNF782//ZNF800//ZFP30//KDM2A//CNOT6L//PTF1A//NRBF2//HIVEP2//ZNF680//MLLT6//COMMD2//VGLL1//SLC2A4RG//PAK6//ZNF630//CNOT6//ZBTB26//RFX3//SOX4//BTF3//BTG1//RHOXF2B//ZNF3//ZNF131//ZNF134//ZNF208//ZNF227//ZNF655//SNIP1//MED28//RPS6KA5//POLR2K//MED6//KLF17//ELK3//EMX2//FOXJ3//FOXF2//EPC2//HBP1//ATAD2//HOXA3//HOXB6//HOXD1//BOLA3//NR3C2//MYBL1//NEUROG1//NFE2//NHLH2//ARID4B//BRWD1//POU3F2//TRERF1//MIER1//RFX4//DMRTA1//RFX7//SMARCD2//FOSL1//TSC22D4//LCOR//RHOXF2//BLZF1//KLF7//CBFB//MTPN//DDX6//TSC1//KBTBD8//EIF3B//FLRT1//NCK1//DBNDD2//ANP32B//EGLN3//CASP3//G3BP2//MDFIC//ATR//CALM2//INHBB//BMP3//BMPR2//ACVR1//LDLR//A2ML1//WFDC13//SPOCK1//PRR16//AGTR2//IL5//IRF1//NEUROD1//NOS1//OTX2//PPP3CB//WNT7A//RASD1//ATXN1//BTAF1//ACSL1//TNFAIP8L3//SPOPL//WRNIP1//SULF1//VWC2//EGLN1//CDK6//RAP2C//TGFBR2//TNKS//DCP2//CCT6A//MTM1//RNF180//RNF125//LONRF3//LONRF1//PHACTR2//AKAP6//IFNA1//PRKAR2B//ITM2A//ARL6IP1//MET//FZD6//FASTK//YTHDF2//PTPN22//UBXN2B//NMI//MID1IP1//GJA1//HECW2//CCNY//MYOG//GPD1//UNG//ADCYAP1//RAPGEF2//NCOA7//TFEC//DLL1//MACC1//MEOX2//ARMCX3//TET2//YES1//PCGF5//NFATC2IP//ST18//HPRT1//GRIK2//SAMSN1//SH3D19//TRIM8//IL1RAP//MYD88//DCUN1D4//ARRDC3//PTGES3//MTMR9//PMEPA1//LDLRAD4//QKI//CARF//CCR1//GPR183//NPNT//CCL2//CCL8//PLAA//CPEB2//TMED10//RAB3GAP1//CPEB1// |
| GO:0097659 | nucleic acid-templated transcription | Biological process | 170 | 3924 | 574 | 17653 | 1.33237523397514 | 1.68973355510084e-05 | 0.00554660311157933 | 4.77218177152374 | 0.29616724738676 | NPAT//RRM2//TSHZ1//ZBTB18//CEBPB//ZNF268//TCERG1//STRAP//KLF12//ARX//EDN1//CC2D1B//EN1//ESR1//CPEB3//MYT1L//RCOR1//ZFPM2//GATA2//CNOT7//HOXC8//HSBP1//IFNG//IRF2//AR//JARID2//ZNF568//MAF//MDM4//MYB//NFIA//NR4A2//KLF3//POU4F1//PPARG//DUSP22//ZBTB4//RBBP8//TNF//ZNF217//LRP8//BHLHE41//TBL1XR1//ARID5B//BHLHE40//RUNX3//PHF14//ELK3//TFEC//FOXJ3//FOXF2//PTF1A//HIVEP2//MACC1//IRF1//MEOX2//MYBL1//NEUROD1//NHLH2//OTX2//POLR2K//POU3F2//RFX4//BMPR2//SOX4//BTF3//CARF//FOSL1//KLF7//CBFB//NMI//SRSF2//BAHD1//ZMYND11//KLF17//NCOA7//ZNF782//ZNF800//ZFP30//KDM2A//CNOT6L//EPC2//HBP1//ATAD2//MDFIC//HOXA3//HOXB6//HOXD1//ZNF680//NEUROG1//NFE2//COMMD2//VGLL1//ARID4B//BRWD1//PRKAA1//TRERF1//SLC2A4RG//ZNF630//CNOT6//ZBTB26//MIER1//RFX3//ATXN1//DMRTA1//SMARCD2//ZNF3//ZNF131//ZNF134//ZNF208//ZNF227//ZNF655//MED28//TSC22D4//PCGF5//LCOR//ST18//NRBF2//MLLT6//PAK6//BTG1//RHOXF2B//SNIP1//CBL//RPS6KA5//MED6//EMX2//BOLA3//NR3C2//RFX7//RHOXF2//BLZF1//G3BP2//EREG//AGTR2//IGF1//IL5//LRP6//NOS1//PPP3CB//WNT7A//ACVR1//RASD1//BTAF1//BMP3//SULF1//VWC2//MYOG//C5AR1//INTS7//NABP1//EGLN1//FZD6//ADCYAP1//DLL1//IL2//MET//NCK1//ARMCX3//TET2//YES1//NFATC2IP//TNKS//TRIM8//MTPN//IL1RAP//MYD88//EGLN3//RAP2C//NPNT// |
| GO:0009891 | positive regulation of biosynthetic process | Biological process | 92 | 1841 | 574 | 17653 | 1.53688250780234 | 1.75679540920941e-05 | 0.00554660311157933 | 4.75527881211025 | 0.160278745644599 | MTPN//IGF1//LDLR//EDN1//CPEB3//FMR1//PRR16//SOX4//AGTR2//ESR1//FOXF2//ATAD2//MDFIC//IL5//IRF1//AR//LRP6//MYB//MYBL1//NEUROD1//NFE2//NOS1//NPAT//OTX2//PPARG//PPP3CB//TRERF1//RFX3//TNF//WNT7A//TBL1XR1//RUNX3//ACVR1//CALM2//ATR//TNKS//CCT6A//NCK1//EREG//IFNG//PRKAA1//MID1IP1//GPD1//MED6//ZBTB18//CEBPB//ZNF268//ADCYAP1//NCOA7//ELK3//EN1//TFEC//FOXJ3//ZFPM2//PTF1A//GATA2//DLL1//CNOT7//MACC1//IL2//IRF2//MAF//MEOX2//MET//MYOG//NEUROG1//NFIA//NHLH2//NR4A2//VGLL1//ARMCX3//ARID4B//POU3F2//POU4F1//EGLN1//TET2//RFX4//BMPR2//YES1//FOSL1//PCGF5//NFATC2IP//KLF7//CBFB//RPS6KA5//ST18//CCL2//PTGES3//CARF//RAB3GAP1//YTHDF2//NPNT// |
| GO:0006351 | transcription, DNA-templated | Biological process | 169 | 3907 | 574 | 17653 | 1.33030101426101 | 1.98761464553872e-05 | 0.00554660311157933 | 4.70166781185948 | 0.294425087108014 | NPAT//RRM2//TSHZ1//ZBTB18//CEBPB//ZNF268//TCERG1//STRAP//KLF12//ARX//EDN1//CC2D1B//EN1//ESR1//CPEB3//MYT1L//RCOR1//ZFPM2//GATA2//CNOT7//HOXC8//HSBP1//IFNG//IRF2//AR//JARID2//ZNF568//MAF//MDM4//MYB//NFIA//NR4A2//KLF3//POU4F1//PPARG//DUSP22//ZBTB4//RBBP8//TNF//ZNF217//LRP8//BHLHE41//TBL1XR1//ARID5B//BHLHE40//RUNX3//PHF14//ELK3//TFEC//FOXJ3//FOXF2//PTF1A//HIVEP2//MACC1//IRF1//MEOX2//MYBL1//NEUROD1//NHLH2//OTX2//POLR2K//POU3F2//RFX4//BMPR2//SOX4//BTF3//CARF//FOSL1//KLF7//CBFB//NMI//SRSF2//BAHD1//ZMYND11//ZNF782//ZNF800//ZFP30//KDM2A//CNOT6L//NRBF2//ZNF680//MLLT6//COMMD2//VGLL1//SLC2A4RG//PAK6//ZNF630//CNOT6//ZBTB26//RFX3//BTG1//RHOXF2B//ZNF3//ZNF131//ZNF134//ZNF208//ZNF227//ZNF655//SNIP1//MED28//CBL//RPS6KA5//MED6//KLF17//EMX2//EPC2//HBP1//ATAD2//HOXA3//HOXB6//HOXD1//BOLA3//NR3C2//NEUROG1//NFE2//ARID4B//BRWD1//TRERF1//MIER1//DMRTA1//RFX7//SMARCD2//TSC22D4//LCOR//RHOXF2//BLZF1//G3BP2//EREG//AGTR2//MDFIC//IGF1//IL5//LRP6//NOS1//PPP3CB//WNT7A//ACVR1//RASD1//ATXN1//BTAF1//BMP3//SULF1//VWC2//MYOG//C5AR1//INTS7//NABP1//EGLN1//FZD6//ADCYAP1//NCOA7//DLL1//IL2//MET//NCK1//ARMCX3//TET2//YES1//PCGF5//NFATC2IP//TNKS//ST18//TRIM8//MTPN//IL1RAP//MYD88//EGLN3//PRKAA1//NPNT// |
| GO:0019219 | regulation of nucleobase-containing compound metabolic process | Biological process | 184 | 4328 | 574 | 17653 | 1.30748645880966 | 2.01288321234826e-05 | 0.00554660311157933 | 4.69618142218242 | 0.320557491289199 | NPAT//RRM2//TSHZ1//ZBTB18//CEBPB//ZNF268//TCERG1//STRAP//KLF12//ARX//EDN1//CC2D1B//EN1//ESR1//CPEB3//MYT1L//RCOR1//ZFPM2//GATA2//CNOT7//HOXC8//HSBP1//IFNG//IRF2//AR//JARID2//ZNF568//MAF//MDM4//MYB//NFIA//NR4A2//KLF3//POU4F1//PPARG//DUSP22//ZBTB4//RBBP8//TNF//ZNF217//LRP8//BHLHE41//TBL1XR1//ARID5B//BHLHE40//RUNX3//PHF14//FMR1//MBNL1//SMU1//RBM25//SRSF2//ESCO2//BAHD1//ZMYND11//ZNF782//ZNF800//ZFP30//KDM2A//CNOT6L//PTF1A//NRBF2//HIVEP2//ZNF680//MLLT6//COMMD2//VGLL1//SLC2A4RG//PAK6//ZNF630//CNOT6//ZBTB26//RFX3//SOX4//BTF3//BTG1//RHOXF2B//ZNF3//ZNF131//ZNF134//ZNF208//ZNF227//ZNF655//SNIP1//MED28//CBL//RPS6KA5//POLR2K//MED6//KLF17//ELK3//EMX2//FOXJ3//FOXF2//EPC2//HBP1//ATAD2//HOXA3//HOXB6//HOXD1//BOLA3//NR3C2//MYBL1//NEUROG1//NFE2//NHLH2//ARID4B//BRWD1//POU3F2//TRERF1//MIER1//RFX4//DMRTA1//RFX7//SMARCD2//FOSL1//TSC22D4//LCOR//RHOXF2//BLZF1//KLF7//CBFB//G3BP2//ATR//AGTR2//MDFIC//IGF1//IL5//IRF1//LRP6//NEUROD1//NOS1//OTX2//PPP3CB//WNT7A//ACVR1//EREG//RASD1//ATXN1//BTAF1//WRNIP1//BMP3//BMPR2//SULF1//VWC2//CALM2//EGLN1//TNKS//DCP2//CCT6A//FZD6//FASTK//YTHDF2//MYOG//GPD1//PRKAA1//UNG//ADCYAP1//NCOA7//TFEC//DLL1//MACC1//IL2//MEOX2//MET//NCK1//ARMCX3//TET2//YES1//PCGF5//NFATC2IP//ST18//TRIM8//MTPN//IL1RAP//MYD88//PTGES3//QKI//CARF//EGLN3//RAP2C//NMI//GJA1//NPNT// |
| GO:0032774 | RNA biosynthetic process | Biological process | 170 | 3940 | 574 | 17653 | 1.32696457312651 | 2.14578768480052e-05 | 0.00554660311157933 | 4.66841325155979 | 0.29616724738676 | NPAT//RRM2//TSHZ1//ZBTB18//CEBPB//ZNF268//TCERG1//STRAP//KLF12//ARX//EDN1//CC2D1B//EN1//ESR1//CPEB3//MYT1L//RCOR1//ZFPM2//GATA2//CNOT7//HOXC8//HSBP1//IFNG//IRF2//AR//JARID2//ZNF568//MAF//MDM4//MYB//NFIA//NR4A2//KLF3//POU4F1//PPARG//DUSP22//ZBTB4//RBBP8//TNF//ZNF217//LRP8//BHLHE41//TBL1XR1//ARID5B//BHLHE40//RUNX3//PHF14//ELK3//TFEC//FOXJ3//FOXF2//PTF1A//HIVEP2//MACC1//IRF1//MEOX2//MYBL1//NEUROD1//NHLH2//OTX2//POLR2K//POU3F2//RFX4//BMPR2//SOX4//BTF3//CARF//FOSL1//KLF7//CBFB//NMI//SRSF2//BAHD1//ZMYND11//KLF17//NCOA7//ZNF782//ZNF800//ZFP30//KDM2A//CNOT6L//EPC2//HBP1//ATAD2//MDFIC//HOXA3//HOXB6//HOXD1//ZNF680//NEUROG1//NFE2//COMMD2//VGLL1//ARID4B//BRWD1//PRKAA1//TRERF1//SLC2A4RG//ZNF630//CNOT6//ZBTB26//MIER1//RFX3//ATXN1//DMRTA1//SMARCD2//ZNF3//ZNF131//ZNF134//ZNF208//ZNF227//ZNF655//MED28//TSC22D4//PCGF5//LCOR//ST18//NRBF2//MLLT6//PAK6//BTG1//RHOXF2B//SNIP1//CBL//RPS6KA5//MED6//EMX2//BOLA3//NR3C2//RFX7//RHOXF2//BLZF1//G3BP2//EREG//AGTR2//IGF1//IL5//LRP6//NOS1//PPP3CB//WNT7A//ACVR1//RASD1//BTAF1//BMP3//SULF1//VWC2//MYOG//C5AR1//INTS7//NABP1//EGLN1//FZD6//ADCYAP1//DLL1//IL2//MET//NCK1//ARMCX3//TET2//YES1//NFATC2IP//TNKS//TRIM8//MTPN//IL1RAP//MYD88//EGLN3//RAP2C//NPNT// |
| GO:0045893 | positive regulation of transcription, DNA-templated | Biological process | 76 | 1450 | 574 | 17653 | 1.61195242100204 | 2.16286868169285e-05 | 0.00554660311157933 | 4.66496984789676 | 0.132404181184669 | ATAD2//ESR1//MED6//ZBTB18//CEBPB//ZNF268//ADCYAP1//NCOA7//EDN1//ELK3//EN1//TFEC//FOXJ3//FOXF2//ZFPM2//PTF1A//GATA2//DLL1//CNOT7//MACC1//IGF1//IL2//IRF1//IRF2//AR//LRP6//MAF//MEOX2//MET//MYB//MYBL1//MYOG//NCK1//NEUROD1//NEUROG1//NFIA//NHLH2//NOS1//NR4A2//OTX2//VGLL1//ARMCX3//ARID4B//POU3F2//POU4F1//EGLN1//PPARG//TET2//PPP3CB//RFX3//RFX4//BMPR2//SOX4//TNF//WNT7A//YES1//TBL1XR1//FOSL1//PCGF5//NFATC2IP//KLF7//CBFB//TNKS//ACVR1//RPS6KA5//ST18//CARF//PRKAA1//NPNT//AGTR2//MDFIC//IL5//NFE2//NPAT//TRERF1//RUNX3// |
| GO:1903508 | positive regulation of nucleic acid-templated transcription | Biological process | 76 | 1450 | 574 | 17653 | 1.61195242100204 | 2.16286868169285e-05 | 0.00554660311157933 | 4.66496984789676 | 0.132404181184669 | AGTR2//ESR1//FOXF2//ATAD2//MDFIC//IGF1//IL5//IRF1//AR//LRP6//MYB//MYBL1//NEUROD1//NFE2//NOS1//NPAT//OTX2//PPARG//PPP3CB//TRERF1//RFX3//SOX4//TNF//WNT7A//TBL1XR1//RUNX3//ACVR1//MED6//ZBTB18//CEBPB//ZNF268//ADCYAP1//NCOA7//EDN1//ELK3//EN1//TFEC//FOXJ3//ZFPM2//PTF1A//GATA2//DLL1//CNOT7//MACC1//IL2//IRF2//MAF//MEOX2//MET//MYOG//NCK1//NEUROG1//NFIA//NHLH2//NR4A2//VGLL1//ARMCX3//ARID4B//POU3F2//POU4F1//EGLN1//TET2//RFX4//BMPR2//YES1//FOSL1//PCGF5//NFATC2IP//KLF7//CBFB//TNKS//RPS6KA5//ST18//CARF//PRKAA1//NPNT// |
| GO:0010628 | positive regulation of gene expression | Biological process | 93 | 1877 | 574 | 17653 | 1.52379065117997 | 2.19140473425817e-05 | 0.00554660311157933 | 4.65927740444661 | 0.162020905923345 | CPEB3//FMR1//PRR16//SOX4//AGTR2//ESR1//FOXF2//ATAD2//MDFIC//IGF1//IL5//IRF1//AR//LRP6//MYB//MYBL1//NEUROD1//NFE2//NOS1//NPAT//OTX2//PPARG//PPP3CB//TRERF1//RFX3//TNF//WNT7A//TBL1XR1//RUNX3//ACVR1//NCK1//POLR2K//MED6//ZBTB18//CEBPB//ZNF268//ADCYAP1//NCOA7//EDN1//ELK3//EN1//TFEC//FOXJ3//ZFPM2//PTF1A//GATA2//DLL1//CNOT7//MACC1//IL2//IRF2//MAF//MEOX2//MET//MYOG//NEUROG1//NFIA//NHLH2//NR4A2//VGLL1//ARMCX3//ARID4B//POU3F2//POU4F1//EGLN1//TET2//RFX4//BMPR2//YES1//FOSL1//PCGF5//NFATC2IP//KLF7//CBFB//TNKS//RPS6KA5//ST18//CARF//MYD88//PRKAA1//YTHDF2//NPNT//ARX//RAB3GAP1//PTPN22//GJA1//IFNG//LDLR//PIK3CB//PTGFR//RHOXF2B//RHOXF2//QKI// |
| GO:1902680 | positive regulation of RNA biosynthetic process | Biological process | 76 | 1451 | 574 | 17653 | 1.6108414958325 | 2.2143765194831e-05 | 0.00554660311157933 | 4.6547485323141 | 0.132404181184669 | AGTR2//ESR1//FOXF2//ATAD2//MDFIC//IGF1//IL5//IRF1//AR//LRP6//MYB//MYBL1//NEUROD1//NFE2//NOS1//NPAT//OTX2//PPARG//PPP3CB//TRERF1//RFX3//SOX4//TNF//WNT7A//TBL1XR1//RUNX3//ACVR1//MED6//ZBTB18//CEBPB//ZNF268//ADCYAP1//NCOA7//EDN1//ELK3//EN1//TFEC//FOXJ3//ZFPM2//PTF1A//GATA2//DLL1//CNOT7//MACC1//IL2//IRF2//MAF//MEOX2//MET//MYOG//NCK1//NEUROG1//NFIA//NHLH2//NR4A2//VGLL1//ARMCX3//ARID4B//POU3F2//POU4F1//EGLN1//TET2//RFX4//BMPR2//YES1//FOSL1//PCGF5//NFATC2IP//KLF7//CBFB//TNKS//RPS6KA5//ST18//CARF//PRKAA1//NPNT// |
| GO:0009889 | regulation of biosynthetic process | Biological process | 197 | 4707 | 574 | 17653 | 1.28714850519169 | 2.33986561294299e-05 | 0.00565161112154766 | 4.63080908499724 | 0.343205574912892 | NPAT//RRM2//TSHZ1//ZBTB18//CEBPB//ZNF268//TCERG1//STRAP//KLF12//ARX//EDN1//CC2D1B//EN1//ESR1//CPEB3//MYT1L//RCOR1//ZFPM2//GATA2//CNOT7//HOXC8//HSBP1//IFNG//IRF2//AR//JARID2//ZNF568//MAF//MDM4//MYB//NFIA//NR4A2//KLF3//POU4F1//PPARG//DUSP22//ZBTB4//RBBP8//TNF//ZNF217//LRP8//BHLHE41//TBL1XR1//ARID5B//BHLHE40//RUNX3//PHF14//SECISBP2L//NLN//ESCO2//BAHD1//ZMYND11//ZNF782//ZNF800//ZFP30//KDM2A//CNOT6L//PTF1A//NRBF2//HIVEP2//ZNF680//MLLT6//COMMD2//VGLL1//SLC2A4RG//PAK6//ZNF630//CNOT6//ZBTB26//RFX3//SOX4//BTF3//BTG1//RHOXF2B//ZNF3//ZNF131//ZNF134//ZNF208//ZNF227//ZNF655//SNIP1//MED28//CBL//RPS6KA5//POLR2K//MED6//KLF17//ELK3//EMX2//FOXJ3//FOXF2//EPC2//HBP1//ATAD2//HOXA3//HOXB6//HOXD1//BOLA3//NR3C2//MYBL1//NEUROG1//NFE2//NHLH2//ARID4B//BRWD1//POU3F2//TRERF1//MIER1//RFX4//DMRTA1//RFX7//SMARCD2//FOSL1//TSC22D4//LCOR//RHOXF2//BLZF1//KLF7//CBFB//MTPN//DDX6//TSC1//KBTBD8//EIF3B//G3BP2//ATR//IGF1//LDLR//FMR1//PRR16//AGTR2//MDFIC//IL5//IRF1//LRP6//NEUROD1//NOS1//OTX2//PPP3CB//WNT7A//ACVR1//EREG//RASD1//ATXN1//BTAF1//WRNIP1//BMP3//BMPR2//SULF1//VWC2//CALM2//EGLN1//TNKS//DCP2//CCT6A//NCK1//ITM2A//FZD6//NMI//SOD1//PRKAA1//MID1IP1//MYOG//GPD1//ADCYAP1//NCOA7//TFEC//DLL1//MACC1//IL2//MEOX2//MET//ARMCX3//TET2//YES1//PCGF5//NFATC2IP//ST18//RAPGEF2//INHBB//TRIM8//IL1RAP//MYD88//CCL2//PTGES3//YTHDF2//QKI//CARF//EGLN3//CPEB2//RAB3GAP1//RAP2C//SRSF2//PPP1R15B//GJA1//NPNT//CPEB1// |
| GO:0007399 | nervous system development | Biological process | 109 | 2296 | 574 | 17653 | 1.46002819628744 | 2.44627629254278e-05 | 0.00570488502291959 | 4.61149449351795 | 0.189895470383275 | DCX//GATA2//GJA1//LRP12//NR4A2//SPOCK1//FZD3//RAPGEF2//SOX4//LRP6//TSC1//FZD6//OGN//POU4F1//WNT7A//SLIT2//ARX//DOK1//DPYSL2//EFNA5//OTX2//PIK3CB//ENAH//KLF7//RPS6KA5//NRXN1//PCDHB10//HAPLN1//FMR1//JARID2//NHLH2//HPCAL4//AGTR2//AK4//RAB3GAP1//CNTNAP2//SPHK2//PTPRG//ATXN1//BMPR2//TGFBR2//NTF3//GSTM3//SNAP25//STMN2//ADCYAP1//NPTN//NCK1//CDK6//DLL1//POU3F2//FLRT1//RFX4//C5AR1//LRP8//EMX2//NEUROD1//EN1//PTF1A//NEUROG1//ANP32B//HOXA3//MTPN//HPRT1//CALM2//CASP3//TACC2//BNIP3//KCNJ10//CEBPB//MYT1L//HOXC8//HOXD1//RUNX3//DLG5//UBE4B//DBNDD2//PSD//SOD1//SBF2//QKI//APCDD1//CCL2//DDX6//VWC2//ULK2//PPP3CB//PPARG//NYAP1//HECW2//NOS1//IL1RAP//NFIA//SYBU//MYB//SULF1//LRIG1//CPEB3//IL2//LDLR//PLAA//GPR183//FUT9//CHRM2//DLG2//GLRB//MBNL1//ZIC5//ITM2A// |
| GO:2000112 | regulation of cellular macromolecule biosynthetic process | Biological process | 185 | 4376 | 574 | 17653 | 1.30017270318303 | 2.66612756282224e-05 | 0.00601034023578894 | 4.57411907528808 | 0.322299651567944 | NPAT//RRM2//TSHZ1//ZBTB18//CEBPB//ZNF268//TCERG1//STRAP//KLF12//ARX//EDN1//CC2D1B//EN1//ESR1//CPEB3//MYT1L//RCOR1//ZFPM2//GATA2//CNOT7//HOXC8//HSBP1//IFNG//IRF2//AR//JARID2//ZNF568//MAF//MDM4//MYB//NFIA//NR4A2//KLF3//POU4F1//PPARG//DUSP22//ZBTB4//RBBP8//TNF//ZNF217//LRP8//BHLHE41//TBL1XR1//ARID5B//BHLHE40//RUNX3//PHF14//SECISBP2L//ESCO2//BAHD1//ZMYND11//ZNF782//ZNF800//ZFP30//KDM2A//CNOT6L//PTF1A//NRBF2//HIVEP2//ZNF680//MLLT6//COMMD2//VGLL1//SLC2A4RG//PAK6//ZNF630//CNOT6//ZBTB26//RFX3//SOX4//BTF3//BTG1//RHOXF2B//ZNF3//ZNF131//ZNF134//ZNF208//ZNF227//ZNF655//SNIP1//MED28//CBL//RPS6KA5//POLR2K//MED6//KLF17//ELK3//EMX2//FOXJ3//FOXF2//EPC2//HBP1//ATAD2//HOXA3//HOXB6//HOXD1//BOLA3//NR3C2//MYBL1//NEUROG1//NFE2//NHLH2//ARID4B//BRWD1//POU3F2//TRERF1//MIER1//RFX4//DMRTA1//RFX7//SMARCD2//FOSL1//TSC22D4//LCOR//RHOXF2//BLZF1//KLF7//CBFB//MTPN//DDX6//TSC1//KBTBD8//EIF3B//G3BP2//ATR//IGF1//FMR1//PRR16//AGTR2//MDFIC//IL5//IRF1//LRP6//NEUROD1//NOS1//OTX2//PPP3CB//WNT7A//ACVR1//EREG//RASD1//ATXN1//BTAF1//WRNIP1//BMP3//BMPR2//SULF1//VWC2//TNKS//DCP2//CCT6A//NCK1//ITM2A//EGLN1//FZD6//ADCYAP1//NCOA7//TFEC//DLL1//MACC1//IL2//MEOX2//MET//MYOG//ARMCX3//TET2//YES1//PCGF5//NFATC2IP//ST18//TRIM8//IL1RAP//MYD88//PTGES3//YTHDF2//QKI//CARF//EGLN3//CPEB2//RAB3GAP1//PRKAA1//PPP1R15B//GJA1//NPNT//CPEB1// |
| GO:0031328 | positive regulation of cellular biosynthetic process | Biological process | 90 | 1812 | 574 | 17653 | 1.52753420864388 | 2.77493651275585e-05 | 0.00605383730186058 | 4.55674694856841 | 0.156794425087108 | IGF1//LDLR//CPEB3//FMR1//PRR16//SOX4//AGTR2//ESR1//FOXF2//ATAD2//MDFIC//IL5//IRF1//AR//LRP6//MYB//MYBL1//NEUROD1//NFE2//NOS1//NPAT//OTX2//PPARG//PPP3CB//TRERF1//RFX3//TNF//WNT7A//TBL1XR1//RUNX3//ACVR1//CALM2//ATR//TNKS//CCT6A//NCK1//EREG//EDN1//IFNG//MID1IP1//GPD1//PRKAA1//MED6//ZBTB18//CEBPB//ZNF268//ADCYAP1//NCOA7//ELK3//EN1//TFEC//FOXJ3//ZFPM2//PTF1A//GATA2//DLL1//CNOT7//MACC1//IL2//IRF2//MAF//MEOX2//MET//MYOG//NEUROG1//NFIA//NHLH2//NR4A2//VGLL1//ARMCX3//ARID4B//POU3F2//POU4F1//EGLN1//TET2//RFX4//BMPR2//YES1//FOSL1//PCGF5//NFATC2IP//KLF7//CBFB//RPS6KA5//ST18//PTGES3//CARF//RAB3GAP1//YTHDF2//NPNT// |
| GO:0031326 | regulation of cellular biosynthetic process | Biological process | 194 | 4640 | 574 | 17653 | 1.28585020425327 | 3.01271134906763e-05 | 0.00636717714179512 | 4.52104247652855 | 0.337979094076655 | NPAT//RRM2//TSHZ1//ZBTB18//CEBPB//ZNF268//TCERG1//STRAP//KLF12//ARX//EDN1//CC2D1B//EN1//ESR1//CPEB3//MYT1L//RCOR1//ZFPM2//GATA2//CNOT7//HOXC8//HSBP1//IFNG//IRF2//AR//JARID2//ZNF568//MAF//MDM4//MYB//NFIA//NR4A2//KLF3//POU4F1//PPARG//DUSP22//ZBTB4//RBBP8//TNF//ZNF217//LRP8//BHLHE41//TBL1XR1//ARID5B//BHLHE40//RUNX3//PHF14//SECISBP2L//ESCO2//BAHD1//ZMYND11//ZNF782//ZNF800//ZFP30//KDM2A//CNOT6L//PTF1A//NRBF2//HIVEP2//ZNF680//MLLT6//COMMD2//VGLL1//SLC2A4RG//PAK6//ZNF630//CNOT6//ZBTB26//RFX3//SOX4//BTF3//BTG1//RHOXF2B//ZNF3//ZNF131//ZNF134//ZNF208//ZNF227//ZNF655//SNIP1//MED28//CBL//RPS6KA5//POLR2K//MED6//KLF17//ELK3//EMX2//FOXJ3//FOXF2//EPC2//HBP1//ATAD2//HOXA3//HOXB6//HOXD1//BOLA3//NR3C2//MYBL1//NEUROG1//NFE2//NHLH2//ARID4B//BRWD1//POU3F2//TRERF1//MIER1//RFX4//DMRTA1//RFX7//SMARCD2//FOSL1//TSC22D4//LCOR//RHOXF2//BLZF1//KLF7//CBFB//MTPN//DDX6//TSC1//KBTBD8//EIF3B//G3BP2//ATR//IGF1//LDLR//FMR1//PRR16//AGTR2//MDFIC//IL5//IRF1//LRP6//NEUROD1//NOS1//OTX2//PPP3CB//WNT7A//ACVR1//EREG//RASD1//ATXN1//BTAF1//WRNIP1//BMP3//BMPR2//SULF1//VWC2//CALM2//EGLN1//TNKS//DCP2//CCT6A//NCK1//ITM2A//FZD6//NMI//MID1IP1//MYOG//GPD1//PRKAA1//ADCYAP1//NCOA7//TFEC//DLL1//MACC1//IL2//MEOX2//MET//ARMCX3//TET2//YES1//PCGF5//NFATC2IP//ST18//RAPGEF2//INHBB//TRIM8//IL1RAP//MYD88//PTGES3//YTHDF2//QKI//CARF//EGLN3//CPEB2//RAB3GAP1//RAP2C//SRSF2//PPP1R15B//GJA1//NPNT//CPEB1// |
| GO:0045935 | positive regulation of nucleobase-containing compound metabolic process | Biological process | 87 | 1742 | 574 | 17653 | 1.5359523076123 | 3.1562095621644e-05 | 0.00646831674815692 | 4.50083416874839 | 0.151567944250871 | AGTR2//ESR1//FOXF2//ATAD2//MDFIC//IGF1//IL5//IRF1//AR//LRP6//MYB//MYBL1//NEUROD1//NFE2//NOS1//NPAT//OTX2//PPARG//PPP3CB//TRERF1//RFX3//SOX4//TNF//WNT7A//TBL1XR1//RUNX3//ACVR1//CALM2//ATR//TNKS//CCT6A//EREG//GPD1//PRKAA1//UNG//MED6//ZBTB18//CEBPB//ZNF268//ADCYAP1//NCOA7//EDN1//ELK3//EN1//TFEC//FOXJ3//ZFPM2//PTF1A//GATA2//DLL1//CNOT7//MACC1//IL2//IRF2//MAF//MEOX2//MET//MYOG//NCK1//NEUROG1//NFIA//NHLH2//NR4A2//VGLL1//ARMCX3//ARID4B//POU3F2//POU4F1//EGLN1//TET2//RFX4//BMPR2//YES1//FOSL1//PCGF5//NFATC2IP//KLF7//CBFB//RPS6KA5//ST18//PTGES3//CPEB3//CNOT6L//YTHDF2//QKI//CARF//NPNT// |
| GO:0060255 | regulation of macromolecule metabolic process | Biological process | 255 | 6425 | 574 | 17653 | 1.22060087582532 | 3.53872076647229e-05 | 0.00703893192460356 | 4.45115370533782 | 0.444250871080139 | GADD45A//DIRAS3//NPAT//RRM2//TSHZ1//ZBTB18//CEBPB//ZNF268//TCERG1//STRAP//KLF12//ARX//EDN1//CC2D1B//EN1//ESR1//CPEB3//MYT1L//RCOR1//ZFPM2//GATA2//CNOT7//HOXC8//HSBP1//IFNG//IRF2//AR//JARID2//ZNF568//MAF//MDM4//MYB//NFIA//NR4A2//KLF3//POU4F1//PPARG//DUSP22//ZBTB4//RBBP8//TNF//ZNF217//LRP8//BHLHE41//TBL1XR1//ARID5B//BHLHE40//RUNX3//PHF14//DCP2//CRKL//MAP3K1//MAP3K7CL//LAMTOR3//IGF1//NTF3//PIK3CB//PRKAA1//SOD1//C5AR1//SPRED1//CNOT6L//PAN3//CNOT6//FMR1//MBNL1//SMU1//RBM25//SRSF2//SECISBP2L//EPS15//IL2//LRP6//PPP1R15B//SLIT2//EFNA5//NPTN//EREG//CBL//SOCS5//ESCO2//BAHD1//ARID4B//ZMYND11//ZNF782//ZNF800//ZFP30//KDM2A//PTF1A//NRBF2//HIVEP2//ZNF680//MLLT6//COMMD2//VGLL1//SLC2A4RG//PAK6//ZNF630//ZBTB26//RFX3//SOX4//BTF3//BTG1//RHOXF2B//ZNF3//ZNF131//ZNF134//ZNF208//ZNF227//ZNF655//SNIP1//MED28//RPS6KA5//POLR2K//MED6//KLF17//ELK3//EMX2//FOXJ3//FOXF2//EPC2//HBP1//ATAD2//HOXA3//HOXB6//HOXD1//BOLA3//NR3C2//MYBL1//NEUROG1//NFE2//NHLH2//BRWD1//POU3F2//TRERF1//MIER1//RFX4//DMRTA1//RFX7//SMARCD2//FOSL1//TSC22D4//LCOR//RHOXF2//BLZF1//KLF7//CBFB//MTPN//DDX6//TSC1//KBTBD8//EIF3B//FLRT1//NCK1//DBNDD2//ANP32B//EGLN3//CASP3//G3BP2//MDFIC//ATR//CDK6//TGFBR2//RAB3GAP1//PTPN22//GJA1//LDLR//MYD88//PTGFR//QKI//CCR1//CALM2//INHBB//BMP3//BMPR2//ACVR1//A2ML1//WFDC13//SPOCK1//PRR16//AGTR2//IL5//IRF1//NEUROD1//NOS1//OTX2//PPP3CB//WNT7A//RASD1//ATXN1//BTAF1//SPOPL//WRNIP1//SULF1//VWC2//RAP2C//TNKS//CCT6A//MTM1//RNF180//RNF125//LONRF3//LONRF1//PHACTR2//AKAP6//IFNA1//PRKAR2B//ITM2A//ARL6IP1//MET//EGLN1//FZD6//FASTK//YTHDF2//UBXN2B//NMI//HECW2//CCNY//UNG//ADCYAP1//RAPGEF2//NCOA7//TFEC//DLL1//MACC1//MEOX2//MYOG//ARMCX3//TET2//YES1//PCGF5//NFATC2IP//ST18//GRIK2//SAMSN1//SH3D19//TRIM8//IL1RAP//DCUN1D4//ARRDC3//CCL2//PTGES3//MTMR9//PMEPA1//LDLRAD4//CARF//GPR183//NPNT//TNFAIP8L3//CCL8//ACSL1//PLAA//CPEB2//TMED10//CPEB1// |
| GO:0010922 | positive regulation of phosphatase activity | Biological process | 7 | 31 | 574 | 17653 | 6.94453186467349 | 4.93559755804055e-05 | 0.00953698465286521 | 4.30666025931673 | 0.0121951219512195 | NPNT//AGTR2//CALM2//PPP1R15B//AKAP6//IFNG//MTMR9// |
| GO:0048523 | negative regulation of cellular process | Biological process | 198 | 4821 | 574 | 17653 | 1.26309113655631 | 7.28953118495518e-05 | 0.0136941942788477 | 4.13730040176901 | 0.344947735191638 | RAD9B//INTS7//ATR//USP28//TSHZ1//ZBTB18//CEBPB//ZNF268//TCERG1//STRAP//KLF12//ARX//EDN1//CC2D1B//EN1//ESR1//CPEB3//MYT1L//RCOR1//ZFPM2//GATA2//CNOT7//HOXC8//HSBP1//IFNG//IRF2//AR//JARID2//ZNF568//MAF//MDM4//MYB//NFIA//NR4A2//KLF3//POU4F1//PPARG//DUSP22//ZBTB4//RBBP8//TNF//ZNF217//LRP8//BHLHE41//TBL1XR1//ARID5B//BHLHE40//RUNX3//PHF14//SPRED1//IL2//LRP6//PPP1R15B//SLIT2//SULF1//GJA1//AIMP1//BMPR2//CBL//SOCS5//BAHD1//GADD45A//FLRT1//NCK1//DBNDD2//CNOT6L//CNOT6//SOX4//STMN2//CLASP2//MID1IP1//CDK6//HBP1//MTBP//IRF1//PRKAA1//RRAGD//TSC1//LAMTOR3//NABP1//ARL6IP1//IGF1//MET//MYD88//ZFAND6//TEX11//SPHK2//PTGFR//BCL2A1//BNIP3//BTC//UNG//WNT7A//CASP3//EREG//FRZB//TES//DLL1//MYOG//BTG1//FOSL1//DLG5//RAPGEF2//ST18//ACVR1//SNIP1//PTPN22//MTMR9//NOS1//MYBL1//PTPRG//ADCYAP1//AGTR2//LDLRAD4//C5ORF30//CALM2//SPOCK1//AKAP6//DLG2//PLEKHF2//A2ML1//WFDC13//SKP1//PMEPA1//ELK3//FOXF2//MDFIC//RASD1//RFX3//ATXN1//TSC22D4//BTAF1//RPS6KA5//INHBB//FMR1//RFX4//APCDD1//SHISA2//EI24//RAP2C//TGFBR2//VWC2//EGLN1//MMRN2//SPOPL//ATAD2//MIER1//MTM1//DCP2//PHACTR2//CD84//CCL2//RNF125//EPS15//ITM2A//ZMYND11//GRIK2//NTF3//SOD1//C5AR1//NMI//ANP32B//REG3G//DDX6//YTHDF2//FZD3//NEUROD1//OGN//ULK2//SAMSN1//CD2AP//ARHGAP12//MED28//TMOD1//OTUD3//POLR2K//QKI//LDLR//RRM2//CCL8//CDC14A//MEOX2//FZD6//EFNA5//PLAA//CPEB2//NCOA7//CCL28//TFEC//NPAT//SRSF2//TNKS//ZNF655//CFDP1//PRKAR2B//CPEB1//MTPN//ARHGAP1// |
| GO:0048608 | reproductive structure development | Biological process | 30 | 430 | 574 | 17653 | 2.1456527023742 | 7.73814409205109e-05 | 0.0141341229624231 | 4.11136318753801 | 0.0522648083623693 | CEBPB//ADCYAP1//SPO11//INHBB//DMRTA1//SOD1//ESR1//EREG//PPARG//GATA2//BMPR2//ZFPM2//RNF38//TEX11//ARID5B//CBL//AR//MGST1//NEUROG1//LRP6//GJA1//GJB2//FOXF2//WNT7A//ZNF568//FOSL1//SULF1//EGLN1//SLIT2//ARID4B// |
| GO:0034654 | nucleobase-containing compound biosynthetic process | Biological process | 186 | 4488 | 574 | 17653 | 1.27457890029626 | 8.28757224264863e-05 | 0.0141341229624231 | 4.08157267283189 | 0.32404181184669 | NPAT//RRM2//TSHZ1//ZBTB18//CEBPB//ZNF268//TCERG1//STRAP//KLF12//ARX//EDN1//CC2D1B//EN1//ESR1//CPEB3//MYT1L//RCOR1//ZFPM2//GATA2//CNOT7//HOXC8//HSBP1//IFNG//IRF2//AR//JARID2//ZNF568//MAF//MDM4//MYB//NFIA//NR4A2//KLF3//POU4F1//PPARG//DUSP22//ZBTB4//RBBP8//TNF//ZNF217//LRP8//BHLHE41//TBL1XR1//ARID5B//BHLHE40//RUNX3//PHF14//SYCP1//WRNIP1//ELK3//TFEC//FOXJ3//FOXF2//PTF1A//HIVEP2//MACC1//IRF1//MEOX2//MYBL1//NEUROD1//NHLH2//OTX2//POLR2K//POU3F2//RFX4//BMPR2//SOX4//BTF3//CARF//FOSL1//KLF7//CBFB//NMI//SRSF2//SLC35D1//HPRT1//IMPDH1//CMPK1//DCTPP1//BAHD1//ZMYND11//KLF17//NCOA7//ZNF782//ZNF800//ZFP30//KDM2A//CNOT6L//EPC2//HBP1//ATAD2//MDFIC//HOXA3//HOXB6//HOXD1//ZNF680//NEUROG1//NFE2//COMMD2//VGLL1//ARID4B//BRWD1//PRKAA1//TRERF1//SLC2A4RG//ZNF630//CNOT6//ZBTB26//MIER1//RFX3//ATXN1//DMRTA1//SMARCD2//ZNF3//ZNF131//ZNF134//ZNF208//ZNF227//ZNF655//MED28//TSC22D4//PCGF5//LCOR//ST18//NRBF2//MLLT6//PAK6//BTG1//RHOXF2B//SNIP1//CBL//RPS6KA5//MED6//EMX2//BOLA3//NR3C2//RFX7//RHOXF2//BLZF1//PTGES3//G3BP2//AK4//EREG//AGTR2//IGF1//IL5//LRP6//NOS1//PPP3CB//WNT7A//ACVR1//RASD1//BTAF1//BMP3//SULF1//VWC2//CALM2//EGLN1//TNKS//DCP2//ATR//CCT6A//MYOG//C5AR1//INTS7//NABP1//FZD6//UNG//GPD1//ADCYAP1//DLL1//IL2//MET//NCK1//ARMCX3//TET2//YES1//NFATC2IP//TRIM8//MTPN//IL1RAP//MYD88//EGLN3//RAP2C//GJA1//NPNT// |
| GO:0022008 | neurogenesis | Biological process | 77 | 1536 | 574 | 17653 | 1.54172224339431 | 8.44516056464897e-05 | 0.0141341229624231 | 4.07339208893748 | 0.134146341463415 | DCX//GATA2//GJA1//LRP12//NR4A2//SPOCK1//FZD3//RAPGEF2//SOX4//OGN//POU4F1//WNT7A//SLIT2//ARX//DOK1//DPYSL2//EFNA5//OTX2//PIK3CB//ENAH//KLF7//RPS6KA5//NRXN1//CNTNAP2//SNAP25//STMN2//ADCYAP1//NPTN//NCK1//PTPRG//TSC1//CDK6//DLL1//POU3F2//FLRT1//MTPN//HPRT1//KCNJ10//CEBPB//EMX2//MYT1L//HOXC8//HOXD1//NEUROG1//CASP3//RUNX3//UBE4B//DBNDD2//PSD//SOD1//PTF1A//NEUROD1//APCDD1//CCL2//NTF3//DDX6//VWC2//EN1//FMR1//ULK2//PPP3CB//BNIP3//PPARG//NYAP1//HECW2//BMPR2//NOS1//MYB//CPEB3//IL2//DLG5//LRP8//LDLR//LRP6//IL1RAP//PLAA//GPR183// |
| GO:0048699 | generation of neurons | Biological process | 73 | 1437 | 574 | 17653 | 1.56232981506672 | 8.70912066446488e-05 | 0.0141341229624231 | 4.06002569227698 | 0.127177700348432 | DCX//GATA2//GJA1//LRP12//NR4A2//SPOCK1//FZD3//RAPGEF2//SOX4//OGN//POU4F1//WNT7A//SLIT2//ARX//DOK1//DPYSL2//EFNA5//OTX2//PIK3CB//ENAH//KLF7//RPS6KA5//NRXN1//CNTNAP2//SNAP25//STMN2//ADCYAP1//NPTN//NCK1//PTPRG//TSC1//FLRT1//MTPN//HPRT1//CEBPB//EMX2//MYT1L//HOXC8//HOXD1//NEUROG1//POU3F2//CASP3//RUNX3//UBE4B//DBNDD2//PSD//PTF1A//NEUROD1//DLL1//NTF3//DDX6//VWC2//EN1//FMR1//ULK2//PPP3CB//PPARG//NYAP1//HECW2//BMPR2//NOS1//SOD1//MYB//CPEB3//IL2//DLG5//LRP8//LDLR//LRP6//IL1RAP//PLAA//GPR183//CDK6// |
| GO:0061458 | reproductive system development | Biological process | 30 | 433 | 574 | 17653 | 2.13078674831618 | 8.76689299988352e-05 | 0.0141341229624231 | 4.05715429398675 | 0.0522648083623693 | CEBPB//ADCYAP1//SPO11//INHBB//DMRTA1//SOD1//ESR1//EREG//PPARG//GATA2//BMPR2//ZFPM2//RNF38//TEX11//ARID5B//CBL//AR//MGST1//NEUROG1//LRP6//GJA1//GJB2//FOXF2//WNT7A//ZNF568//FOSL1//SULF1//EGLN1//SLIT2//ARID4B// |
| GO:0019222 | regulation of metabolic process | Biological process | 269 | 6920 | 574 | 17653 | 1.19550890213692 | 8.87803673167006e-05 | 0.0141341229624231 | 4.05168306247256 | 0.468641114982578 | GADD45A//DIRAS3//NPAT//RRM2//TSHZ1//ZBTB18//CEBPB//ZNF268//TCERG1//STRAP//KLF12//ARX//EDN1//CC2D1B//EN1//ESR1//CPEB3//MYT1L//RCOR1//ZFPM2//GATA2//CNOT7//HOXC8//HSBP1//IFNG//IRF2//AR//JARID2//ZNF568//MAF//MDM4//MYB//NFIA//NR4A2//KLF3//POU4F1//PPARG//DUSP22//ZBTB4//RBBP8//TNF//ZNF217//LRP8//BHLHE41//TBL1XR1//ARID5B//BHLHE40//RUNX3//PHF14//DCP2//CRKL//MAP3K1//MAP3K7CL//LAMTOR3//IGF1//NTF3//PIK3CB//PRKAA1//SOD1//C5AR1//SPRED1//CNOT6L//PAN3//CNOT6//FMR1//MBNL1//SMU1//RBM25//SRSF2//SECISBP2L//EPS15//IL2//LRP6//PPP1R15B//SLIT2//EFNA5//NPTN//EREG//CBL//SOCS5//NLN//ESCO2//BAHD1//ARID4B//ZMYND11//ZNF782//ZNF800//ZFP30//KDM2A//PTF1A//NRBF2//HIVEP2//ZNF680//MLLT6//COMMD2//VGLL1//SLC2A4RG//PAK6//ZNF630//ZBTB26//RFX3//SOX4//BTF3//BTG1//RHOXF2B//ZNF3//ZNF131//ZNF134//ZNF208//ZNF227//ZNF655//SNIP1//MED28//RPS6KA5//POLR2K//MED6//KLF17//ELK3//EMX2//FOXJ3//FOXF2//EPC2//HBP1//ATAD2//HOXA3//HOXB6//HOXD1//BOLA3//NR3C2//MYBL1//NEUROG1//NFE2//NHLH2//BRWD1//POU3F2//TRERF1//MIER1//RFX4//DMRTA1//RFX7//SMARCD2//FOSL1//TSC22D4//LCOR//RHOXF2//BLZF1//KLF7//CBFB//MTPN//DDX6//TSC1//KBTBD8//EIF3B//FLRT1//NCK1//DBNDD2//ANP32B//EGLN3//CASP3//G3BP2//MDFIC//ATR//PIGA//CDK6//TGFBR2//DRAM1//RRAGD//ULK2//PTPN22//MET//MTMR9//BNIP3//TRIM8//RAB3GAP1//GJA1//LDLR//MYD88//PTGFR//QKI//CCR1//NPNT//CALM2//INHBB//BMP3//BMPR2//ACVR1//DLG2//PLEKHF2//A2ML1//WFDC13//SPOCK1//PRR16//RAB12//SNX5//AGTR2//IL5//IRF1//NEUROD1//NOS1//OTX2//PPP3CB//WNT7A//RASD1//ATXN1//BTAF1//ACSL1//TNFAIP8L3//SPOPL//WRNIP1//SULF1//VWC2//EGLN1//RAP2C//TNKS//CCT6A//MTM1//RNF180//RNF125//LONRF3//LONRF1//PHACTR2//AKAP6//IFNA1//PRKAR2B//PTGES3//ITM2A//ARL6IP1//FZD6//FASTK//YTHDF2//UBXN2B//NMI//MID1IP1//HECW2//CCNY//MYOG//GPD1//UNG//ADCYAP1//RAPGEF2//NCOA7//TFEC//DLL1//MACC1//MEOX2//ARMCX3//TET2//YES1//PCGF5//NFATC2IP//ST18//HPRT1//GRIK2//SAMSN1//SH3D19//IL1RAP//DCUN1D4//ARRDC3//CCL2//PMEPA1//LDLRAD4//CARF//GPR183//CCL8//PLAA//CPEB2//CALCOCO2//TMED10//CPEB1// |
| GO:0051247 | positive regulation of protein metabolic process | Biological process | 81 | 1641 | 574 | 17653 | 1.51803948047315 | 9.11871744195608e-05 | 0.0141341229624231 | 4.04006624139212 | 0.141114982578397 | GADD45A//TNF//CRKL//MAP3K1//MAP3K7CL//LAMTOR3//IGF1//NTF3//PIK3CB//PRKAA1//SOD1//C5AR1//EFNA5//FMR1//NPTN//IFNG//EREG//ANP32B//EGLN3//PPARG//CASP3//MDFIC//CALM2//INHBB//BMP3//BMPR2//ACVR1//CPEB3//PRR16//SOX4//RAP2C//PAK6//TGFBR2//WNT7A//RNF180//RNF125//LONRF3//LONRF1//SOCS5//AGTR2//PPP1R15B//AKAP6//RPS6KA5//IFNA1//PRKAR2B//NOS1//NCK1//IL2//EDN1//AR//MET//IRF1//GJA1//HECW2//CCNY//ADCYAP1//RAPGEF2//DUSP22//IL5//YES1//LRP8//SH3D19//DCUN1D4//ARRDC3//MYB//JARID2//CCR1//GPR183//NPNT//PTPN22//TNFAIP8L3//CCL2//CCL8//ACSL1//NMI//RAB3GAP1//YTHDF2//ESR1//LDLR//ST18//MTPN// |
| GO:2000765 | regulation of cytoplasmic translation | Biological process | 6 | 24 | 574 | 17653 | 7.68858885017422 | 9.39572874547376e-05 | 0.0141341229624231 | 4.02706952978762 | 0.0104529616724739 | CPEB2//CPEB3//NCK1//YTHDF2//FMR1//CPEB1// |
| GO:0048519 | negative regulation of biological process | Biological process | 214 | 5303 | 574 | 17653 | 1.24107713666776 | 9.40463600930119e-05 | 0.0141341229624231 | 4.0266580084295 | 0.372822299651568 | RAD9B//INTS7//ATR//USP28//TSHZ1//ZBTB18//CEBPB//ZNF268//TCERG1//STRAP//KLF12//ARX//EDN1//CC2D1B//EN1//ESR1//CPEB3//MYT1L//RCOR1//ZFPM2//GATA2//CNOT7//HOXC8//HSBP1//IFNG//IRF2//AR//JARID2//ZNF568//MAF//MDM4//MYB//NFIA//NR4A2//KLF3//POU4F1//PPARG//DUSP22//ZBTB4//RBBP8//TNF//ZNF217//LRP8//BHLHE41//TBL1XR1//ARID5B//BHLHE40//RUNX3//PHF14//DCP2//SPRED1//CNOT6L//PAN3//CNOT6//RPS6KA5//PPP3CB//IL2//LRP6//PPP1R15B//SLIT2//SULF1//GJA1//AIMP1//SAMSN1//ADCYAP1//BMPR2//CBL//SOCS5//NOS1//BAHD1//GADD45A//FLRT1//NCK1//DBNDD2//SOX4//STMN2//CLASP2//MID1IP1//CDK6//HBP1//MTBP//IRF1//PRKAA1//RRAGD//TSC1//LAMTOR3//NABP1//G3BP2//ARL6IP1//IGF1//MET//MYD88//ZFAND6//TEX11//SPHK2//PTGFR//BCL2A1//BNIP3//BTC//UNG//WNT7A//CASP3//EREG//FRZB//TES//DLL1//MYOG//BTG1//FOSL1//DLG5//RAPGEF2//ST18//PROCR//HS3ST5//ACVR1//SNIP1//AGTR2//PTPN22//MTMR9//MYBL1//CCR1//LDLR//POU3F2//PTPRG//LDLRAD4//C5ORF30//CALM2//SPOCK1//AKAP6//DLG2//PLEKHF2//A2ML1//WFDC13//SKP1//PMEPA1//IQGAP2//CPEB2//ELK3//FOXF2//MDFIC//RASD1//RFX3//ATXN1//TSC22D4//BTAF1//INHBB//FMR1//RFX4//APCDD1//SHISA2//EI24//RAP2C//TGFBR2//VWC2//EGLN1//MMRN2//SPOPL//ARRDC3//ATAD2//MIER1//MTM1//RNF125//PHACTR2//CD84//TRIM8//CD2AP//CCL2//EPS15//ITM2A//ZMYND11//PXK//FZD6//YTHDF2//GRIK2//NTF3//SOD1//C5AR1//DDX6//NMI//ANP32B//REG3G//FZD3//NEUROD1//OGN//ULK2//ARHGAP12//MED28//TMOD1//OTUD3//NRXN1//POLR2K//QKI//RRM2//CCL8//CDC14A//MEOX2//EFNA5//PLAA//NEUROG1//NCOA7//CHMP3//CCL28//TFEC//NPAT//SRSF2//TNKS//SESTD1//ZNF655//CFDP1//PRKAR2B//HECW2//CPEB1//MTPN//ARHGAP1// |
| GO:0007417 | central nervous system development | Biological process | 52 | 932 | 574 | 17653 | 1.7159082412406 | 0.000103296734053874 | 0.0149126448991829 | 3.98591340940781 | 0.0905923344947735 | DPYSL2//AGTR2//AK4//RAB3GAP1//CNTNAP2//SPHK2//PTPRG//ATXN1//BMPR2//TGFBR2//CDK6//DLL1//POU3F2//SOX4//GATA2//RFX4//C5AR1//POU4F1//LRP8//EMX2//NEUROD1//EN1//PTF1A//LRP6//ANP32B//RAPGEF2//MTPN//WNT7A//HPRT1//ARX//CALM2//TSC1//CASP3//SLIT2//NR4A2//SPOCK1//ADCYAP1//TACC2//BNIP3//KCNJ10//OTX2//DLG5//FZD3//FZD6//PPARG//LDLR//HAPLN1//DCX//FMR1//JARID2//NHLH2//HPCAL4// |
| GO:0032270 | positive regulation of cellular protein metabolic process | Biological process | 77 | 1546 | 574 | 17653 | 1.53174991323005 | 0.000104103415958269 | 0.0149126448991829 | 3.98253501969708 | 0.134146341463415 | GADD45A//TNF//CRKL//MAP3K1//MAP3K7CL//LAMTOR3//IGF1//NTF3//PIK3CB//PRKAA1//SOD1//C5AR1//EFNA5//FMR1//NPTN//IFNG//EREG//ANP32B//EGLN3//PPARG//CASP3//MDFIC//CALM2//INHBB//BMP3//BMPR2//ACVR1//CPEB3//PRR16//SOX4//RAP2C//PAK6//TGFBR2//RNF180//RNF125//LONRF3//LONRF1//SOCS5//AGTR2//PPP1R15B//AKAP6//RPS6KA5//IFNA1//PRKAR2B//NOS1//NCK1//IL2//EDN1//AR//MET//CCNY//ADCYAP1//RAPGEF2//DUSP22//WNT7A//IL5//YES1//LRP8//SH3D19//DCUN1D4//ARRDC3//MYB//JARID2//CCR1//GPR183//NPNT//PTPN22//TNFAIP8L3//CCL2//CCL8//ACSL1//NMI//RAB3GAP1//YTHDF2//ESR1//LDLR//ST18// |
| GO:0031331 | positive regulation of cellular catabolic process | Biological process | 25 | 335 | 574 | 17653 | 2.29510114930574 | 0.000106281056930115 | 0.0149126448991829 | 3.97354413532467 | 0.0435540069686411 | IFNG//PIK3CB//PRKAA1//BNIP3//TRIM8//RAB12//TSC1//RNF180//RNF125//LONRF3//LONRF1//SOCS5//GPD1//IGF1//SH3D19//TNF//CPEB3//CNOT7//CNOT6L//YTHDF2//QKI//CALCOCO2//FMR1//LDLR//RAB3GAP1// |
| GO:1903507 | negative regulation of nucleic acid-templated transcription | Biological process | 64 | 1224 | 574 | 17653 | 1.60807087062467 | 0.000109456862965288 | 0.0149126448991829 | 3.96075700289905 | 0.111498257839721 | TSHZ1//ZBTB18//CEBPB//ZNF268//TCERG1//STRAP//KLF12//ARX//EDN1//CC2D1B//EN1//ESR1//CPEB3//MYT1L//RCOR1//ZFPM2//GATA2//CNOT7//HOXC8//HSBP1//IFNG//IRF2//AR//JARID2//ZNF568//MAF//MDM4//MYB//NFIA//NR4A2//KLF3//POU4F1//PPARG//DUSP22//ZBTB4//RBBP8//TNF//ZNF217//LRP8//BHLHE41//TBL1XR1//ARID5B//BHLHE40//RUNX3//PHF14//BAHD1//ELK3//EREG//FOXF2//MDFIC//IRF1//RASD1//RFX3//ATXN1//TSC22D4//BTAF1//RPS6KA5//ATAD2//MIER1//NCK1//ZMYND11//TFEC//NPAT//SRSF2// |
| GO:0006914 | autophagy | Biological process | 32 | 482 | 574 | 17653 | 2.0417829311666 | 0.00011435479197632 | 0.0149126448991829 | 3.94174563179822 | 0.0557491289198606 | UBXN2B//STX12//ATG16L1//ULK2//BNIP3//DRAM1//RRAGD//PTPN22//MET//MTMR9//IFNG//PIK3CB//PRKAA1//TRIM8//VPS37A//DYNLL2//CHMP3//PLEKHF2//PLAA//EI24//RAB12//TSC1//SNX5//CASP3//LAMTOR3//ACBD5//WDFY3//CALCOCO2//MTM1//RAB3GAP1//NRBF2//PGC// |
| GO:0061919 | process utilizing autophagic mechanism | Biological process | 32 | 482 | 574 | 17653 | 2.0417829311666 | 0.00011435479197632 | 0.0149126448991829 | 3.94174563179822 | 0.0557491289198606 | UBXN2B//STX12//ATG16L1//ULK2//BNIP3//RAB12//PTPN22//NRBF2//PGC//PIK3CB//DRAM1//RRAGD//MET//MTMR9//IFNG//PRKAA1//TRIM8//VPS37A//DYNLL2//CHMP3//PLEKHF2//PLAA//EI24//TSC1//SNX5//CASP3//LAMTOR3//ACBD5//WDFY3//CALCOCO2//MTM1//RAB3GAP1// |
| GO:1902679 | negative regulation of RNA biosynthetic process | Biological process | 64 | 1226 | 574 | 17653 | 1.60544759024845 | 0.000114661767670784 | 0.0149126448991829 | 3.94058136725566 | 0.111498257839721 | TSHZ1//ZBTB18//CEBPB//ZNF268//TCERG1//STRAP//KLF12//ARX//EDN1//CC2D1B//EN1//ESR1//CPEB3//MYT1L//RCOR1//ZFPM2//GATA2//CNOT7//HOXC8//HSBP1//IFNG//IRF2//AR//JARID2//ZNF568//MAF//MDM4//MYB//NFIA//NR4A2//KLF3//POU4F1//PPARG//DUSP22//ZBTB4//RBBP8//TNF//ZNF217//LRP8//BHLHE41//TBL1XR1//ARID5B//BHLHE40//RUNX3//PHF14//BAHD1//ELK3//EREG//FOXF2//MDFIC//IRF1//RASD1//RFX3//ATXN1//TSC22D4//BTAF1//RPS6KA5//ATAD2//MIER1//ZMYND11//TFEC//NPAT//SRSF2//NCK1// |
| GO:0009719 | response to endogenous stimulus | Biological process | 79 | 1606 | 574 | 17653 | 1.51282321086865 | 0.000125738304321786 | 0.0159606738684735 | 3.90053240074308 | 0.137630662020906 | DUSP22//TGFBR2//CBL//ACVR1//CHRM2//APPL1//SPRED1//FLRT1//POLR2K//STEAP2//NOS1//SNRPN//PPARG//CEBPB//CPEB3//RRAGD//CPEB1//TNF//LAMTOR3//BMP3//BMPR2//NPNT//STRAP//PMEPA1//LDLRAD4//SULF1//VWC2//ESR1//AR//GJB2//PTGFR//RBBP8//WNT7A//CASP3//FOSL1//IGF1//TSC1//CPEB2//INHBB//SLIT2//UBA5//CBFB//ACSL1//EDN1//YES1//AGTR2//NTF3//RAPGEF2//OTX2//SHISA2//NR3C2//NR4A2//PAQR5//REG3G//EREG//GJA1//LRP6//BTG1//CCL2//NPTN//NCK1//PTPN22//SOD1//GPD1//AKAP6//EFNA5//PRKAR2B//PRKAA1//ADCYAP1//MYOG//POU4F1//LDLR//SOCS5//FIBIN//RUNX3//MYD88//KLF3//STMN2//DYNC1LI2// |
| GO:0018130 | heterocycle biosynthetic process | Biological process | 187 | 4550 | 574 | 17653 | 1.2639702109737 | 0.000127439951042077 | 0.0159606738684735 | 3.89469440404748 | 0.325783972125436 | NPAT//RRM2//TSHZ1//ZBTB18//CEBPB//ZNF268//TCERG1//STRAP//KLF12//ARX//EDN1//CC2D1B//EN1//ESR1//CPEB3//MYT1L//RCOR1//ZFPM2//GATA2//CNOT7//HOXC8//HSBP1//IFNG//IRF2//AR//JARID2//ZNF568//MAF//MDM4//MYB//NFIA//NR4A2//KLF3//POU4F1//PPARG//DUSP22//ZBTB4//RBBP8//TNF//ZNF217//LRP8//BHLHE41//TBL1XR1//ARID5B//BHLHE40//RUNX3//PHF14//SYCP1//WRNIP1//ELK3//TFEC//FOXJ3//FOXF2//PTF1A//HIVEP2//MACC1//IRF1//MEOX2//MYBL1//NEUROD1//NHLH2//OTX2//POLR2K//POU3F2//RFX4//BMPR2//SOX4//BTF3//CARF//FOSL1//KLF7//CBFB//NMI//SRSF2//SLC35D1//HPRT1//IMPDH1//CMPK1//DCTPP1//BAHD1//ZMYND11//KLF17//NCOA7//ZNF782//ZNF800//ZFP30//KDM2A//CNOT6L//EPC2//HBP1//ATAD2//MDFIC//HOXA3//HOXB6//HOXD1//ZNF680//NEUROG1//NFE2//COMMD2//VGLL1//ARID4B//BRWD1//PRKAA1//TRERF1//SLC2A4RG//ZNF630//CNOT6//ZBTB26//MIER1//RFX3//ATXN1//DMRTA1//SMARCD2//ZNF3//ZNF131//ZNF134//ZNF208//ZNF227//ZNF655//MED28//TSC22D4//PCGF5//LCOR//ST18//NRBF2//MLLT6//PAK6//BTG1//RHOXF2B//SNIP1//CBL//RPS6KA5//MED6//EMX2//BOLA3//NR3C2//RFX7//RHOXF2//BLZF1//GPHN//PTGES3//G3BP2//AK4//EREG//AGTR2//IGF1//IL5//LRP6//NOS1//PPP3CB//WNT7A//ACVR1//RASD1//BTAF1//BMP3//SULF1//VWC2//CALM2//EGLN1//TNKS//DCP2//ATR//CCT6A//MYOG//C5AR1//INTS7//NABP1//FZD6//UNG//GPD1//ADCYAP1//DLL1//IL2//MET//NCK1//ARMCX3//TET2//YES1//NFATC2IP//TRIM8//MTPN//IL1RAP//MYD88//EGLN3//RAP2C//GJA1//NPNT// |
| GO:0000122 | negative regulation of transcription by RNA polymerase II | Biological process | 46 | 800 | 574 | 17653 | 1.76837543554007 | 0.000131813029443462 | 0.0161343190513722 | 3.88004165851502 | 0.0801393728222996 | NCK1//TSHZ1//ZBTB18//CEBPB//ZNF268//TCERG1//STRAP//KLF12//ARX//EDN1//CC2D1B//EN1//ESR1//CPEB3//MYT1L//RCOR1//ZFPM2//GATA2//CNOT7//HOXC8//HSBP1//IFNG//IRF2//AR//JARID2//ZNF568//MAF//MDM4//MYB//NFIA//NR4A2//KLF3//POU4F1//PPARG//DUSP22//ZBTB4//RBBP8//TNF//ZNF217//LRP8//BHLHE41//TBL1XR1//ARID5B//BHLHE40//RUNX3//PHF14// |
| GO:0045934 | negative regulation of nucleobase-containing compound metabolic process | Biological process | 73 | 1457 | 574 | 17653 | 1.54088396997314 | 0.000133597791938022 | 0.0161343190513722 | 3.87420071968276 | 0.127177700348432 | TSHZ1//ZBTB18//CEBPB//ZNF268//TCERG1//STRAP//KLF12//ARX//EDN1//CC2D1B//EN1//ESR1//CPEB3//MYT1L//RCOR1//ZFPM2//GATA2//CNOT7//HOXC8//HSBP1//IFNG//IRF2//AR//JARID2//ZNF568//MAF//MDM4//MYB//NFIA//NR4A2//KLF3//POU4F1//PPARG//DUSP22//ZBTB4//RBBP8//TNF//ZNF217//LRP8//BHLHE41//TBL1XR1//ARID5B//BHLHE40//RUNX3//PHF14//BAHD1//ATR//ELK3//EREG//FOXF2//MDFIC//IRF1//RASD1//RFX3//ATXN1//TSC22D4//BTAF1//RPS6KA5//EGLN1//ATAD2//MIER1//DCP2//MYOG//MYD88//FMR1//ZMYND11//TFEC//NPAT//SRSF2//TNKS//NCK1//GJA1//BMPR2// |
| GO:0019438 | aromatic compound biosynthetic process | Biological process | 187 | 4558 | 574 | 17653 | 1.2617517463647 | 0.000140958662092164 | 0.0167246216092861 | 3.85090823101452 | 0.325783972125436 | NPAT//RRM2//TSHZ1//ZBTB18//CEBPB//ZNF268//TCERG1//STRAP//KLF12//ARX//EDN1//CC2D1B//EN1//ESR1//CPEB3//MYT1L//RCOR1//ZFPM2//GATA2//CNOT7//HOXC8//HSBP1//IFNG//IRF2//AR//JARID2//ZNF568//MAF//MDM4//MYB//NFIA//NR4A2//KLF3//POU4F1//PPARG//DUSP22//ZBTB4//RBBP8//TNF//ZNF217//LRP8//BHLHE41//TBL1XR1//ARID5B//BHLHE40//RUNX3//PHF14//SYCP1//WRNIP1//ELK3//TFEC//FOXJ3//FOXF2//PTF1A//HIVEP2//MACC1//IRF1//MEOX2//MYBL1//NEUROD1//NHLH2//OTX2//POLR2K//POU3F2//RFX4//BMPR2//SOX4//BTF3//CARF//FOSL1//KLF7//CBFB//NMI//SRSF2//SLC35D1//HPRT1//IMPDH1//CMPK1//DCTPP1//BAHD1//ZMYND11//KLF17//NCOA7//ZNF782//ZNF800//ZFP30//KDM2A//CNOT6L//EPC2//HBP1//ATAD2//MDFIC//HOXA3//HOXB6//HOXD1//ZNF680//NEUROG1//NFE2//COMMD2//VGLL1//ARID4B//BRWD1//PRKAA1//TRERF1//SLC2A4RG//ZNF630//CNOT6//ZBTB26//MIER1//RFX3//ATXN1//DMRTA1//SMARCD2//ZNF3//ZNF131//ZNF134//ZNF208//ZNF227//ZNF655//MED28//TSC22D4//PCGF5//LCOR//ST18//NRBF2//MLLT6//PAK6//BTG1//RHOXF2B//SNIP1//CBL//RPS6KA5//MED6//EMX2//BOLA3//NR3C2//RFX7//RHOXF2//BLZF1//PTGES3//G3BP2//AK4//EREG//AGTR2//IGF1//IL5//LRP6//NOS1//PPP3CB//WNT7A//ACVR1//RASD1//BTAF1//BMP3//SULF1//VWC2//CALM2//EGLN1//TNKS//DCP2//ATR//CCT6A//MYOG//C5AR1//INTS7//NABP1//FZD6//UNG//GPD1//ADCYAP1//DLL1//IL2//MET//NCK1//ARMCX3//TET2//YES1//NFATC2IP//RAPGEF2//TRIM8//MTPN//IL1RAP//MYD88//EGLN3//RAP2C//GJA1//NPNT// |
| GO:1901576 | organic substance biosynthetic process | Biological process | 259 | 6665 | 574 | 17653 | 1.195105483688 | 0.000145515952436333 | 0.0167359210312963 | 3.83708939378936 | 0.451219512195122 | RAD9B//IGF1//NFIA//ATR//WRNIP1//RBBP8//RRM2//DTD1//NPAT//TSHZ1//ZBTB18//CEBPB//ZNF268//TCERG1//STRAP//KLF12//ARX//EDN1//CC2D1B//EN1//ESR1//CPEB3//MYT1L//RCOR1//ZFPM2//GATA2//CNOT7//HOXC8//HSBP1//IFNG//IRF2//AR//JARID2//ZNF568//MAF//MDM4//MYB//NR4A2//KLF3//POU4F1//PPARG//DUSP22//ZBTB4//TNF//ZNF217//LRP8//BHLHE41//TBL1XR1//ARID5B//BHLHE40//RUNX3//PHF14//SYCP1//SECISBP2L//PTGES3//MGST1//ST8SIA3//ELK3//TFEC//FOXJ3//FOXF2//PTF1A//HIVEP2//MACC1//IRF1//MEOX2//MYBL1//NEUROD1//NHLH2//OTX2//POLR2K//POU3F2//RFX4//BMPR2//SOX4//BTF3//CARF//FOSL1//KLF7//CBFB//NMI//SRSF2//HS3ST5//SLC35D1//GPD1//GPD2//MDH1//NLN//HPRT1//IMPDH1//CMPK1//DCTPP1//GMNC//ESCO2//BAHD1//ZMYND11//KLF17//NCOA7//ZNF782//ZNF800//ZFP30//KDM2A//CNOT6L//EPC2//HBP1//ATAD2//MDFIC//HOXA3//HOXB6//HOXD1//ZNF680//NEUROG1//NFE2//COMMD2//VGLL1//ARID4B//BRWD1//PRKAA1//TRERF1//SLC2A4RG//ZNF630//CNOT6//ZBTB26//MIER1//RFX3//ATXN1//DMRTA1//SMARCD2//ZNF3//ZNF131//ZNF134//ZNF208//ZNF227//ZNF655//MED28//TSC22D4//PCGF5//LCOR//ST18//NRBF2//MLLT6//PAK6//BTG1//RHOXF2B//SNIP1//CBL//RPS6KA5//MED6//EMX2//BOLA3//NR3C2//RFX7//RHOXF2//BLZF1//CPEB2//MRPL35//CPEB1//METTL17//EIF3B//MTPN//DDX6//TSC1//KBTBD8//AIMP1//FUT9//FUT2//ST8SIA5//MAN1A1//CCDC126//TET2//VEGFB//PIGP//PIGA//GLUD2//LGSN//SERINC3//DDHD2//LCLAT1//PLA2G12A//CDS1//ABHD3//SLC44A1//MTM1//PIK3CB//MTMR9//SPHK2//ACBD3//MSMO1//HSD11B1//GGT6//GPHN//G3BP2//ACSL1//DGKE//MTRR//AK4//GLT6D1//EREG//LDLR//NDST3//FMR1//PRR16//PIGS//AGTR2//IL5//LRP6//NOS1//PPP3CB//WNT7A//ACVR1//RASD1//BTAF1//OGN//CHST1//ELOVL7//CHST7//DSEL//BMP3//SULF1//VWC2//CALM2//EGLN1//TNKS//DCP2//CCT6A//NCK1//IL1RAP//QKI//MYOG//C5AR1//INTS7//NABP1//ITM2A//FZD6//UNG//SOD1//MID1IP1//ADCYAP1//DLL1//IL2//MET//ARMCX3//YES1//NFATC2IP//RAPGEF2//INHBB//TRIM8//MYD88//CCL2//YTHDF2//EGLN3//ATG16L1//GSTA2//GSTM3//RAB3GAP1//RAP2C//PPP1R15B//GJA1//NPNT// |
| GO:1901362 | organic cyclic compound biosynthetic process | Biological process | 192 | 4704 | 574 | 17653 | 1.25527981227334 | 0.00014600315552957 | 0.0167359210312963 | 3.83563775781645 | 0.334494773519164 | NPAT//RRM2//TSHZ1//ZBTB18//CEBPB//ZNF268//TCERG1//STRAP//KLF12//ARX//EDN1//CC2D1B//EN1//ESR1//CPEB3//MYT1L//RCOR1//ZFPM2//GATA2//CNOT7//HOXC8//HSBP1//IFNG//IRF2//AR//JARID2//ZNF568//MAF//MDM4//MYB//NFIA//NR4A2//KLF3//POU4F1//PPARG//DUSP22//ZBTB4//RBBP8//TNF//ZNF217//LRP8//BHLHE41//TBL1XR1//ARID5B//BHLHE40//RUNX3//PHF14//SYCP1//WRNIP1//ELK3//TFEC//FOXJ3//FOXF2//PTF1A//HIVEP2//MACC1//IRF1//MEOX2//MYBL1//NEUROD1//NHLH2//OTX2//POLR2K//POU3F2//RFX4//BMPR2//SOX4//BTF3//CARF//FOSL1//KLF7//CBFB//NMI//SRSF2//SLC35D1//HPRT1//IMPDH1//CMPK1//DCTPP1//BAHD1//ZMYND11//KLF17//NCOA7//ZNF782//ZNF800//ZFP30//KDM2A//CNOT6L//EPC2//HBP1//ATAD2//MDFIC//HOXA3//HOXB6//HOXD1//ZNF680//NEUROG1//NFE2//COMMD2//VGLL1//ARID4B//BRWD1//PRKAA1//TRERF1//SLC2A4RG//ZNF630//CNOT6//ZBTB26//MIER1//RFX3//ATXN1//DMRTA1//SMARCD2//ZNF3//ZNF131//ZNF134//ZNF208//ZNF227//ZNF655//MED28//TSC22D4//PCGF5//LCOR//ST18//NRBF2//MLLT6//PAK6//BTG1//RHOXF2B//SNIP1//CBL//RPS6KA5//MED6//EMX2//BOLA3//NR3C2//RFX7//RHOXF2//BLZF1//ACBD3//MSMO1//HSD11B1//GPHN//PTGES3//G3BP2//AK4//EREG//AGTR2//IGF1//IL5//LRP6//NOS1//PPP3CB//WNT7A//ACVR1//RASD1//BTAF1//BMP3//SULF1//VWC2//CALM2//EGLN1//TNKS//DCP2//ATR//CCT6A//MYOG//C5AR1//INTS7//NABP1//FZD6//UNG//SOD1//GPD1//ADCYAP1//DLL1//IL2//MET//NCK1//ARMCX3//TET2//YES1//NFATC2IP//RAPGEF2//TRIM8//MTPN//IL1RAP//MYD88//EGLN3//RAP2C//GJA1//NPNT// |
| GO:0051172 | negative regulation of nitrogen compound metabolic process | Biological process | 116 | 2591 | 574 | 17653 | 1.37688353009681 | 0.000159012877011743 | 0.0179234014538403 | 3.79856770468145 | 0.202090592334495 | TSHZ1//ZBTB18//CEBPB//ZNF268//TCERG1//STRAP//KLF12//ARX//EDN1//CC2D1B//EN1//ESR1//CPEB3//MYT1L//RCOR1//ZFPM2//GATA2//CNOT7//HOXC8//HSBP1//IFNG//IRF2//AR//JARID2//ZNF568//MAF//MDM4//MYB//NFIA//NR4A2//KLF3//POU4F1//PPARG//DUSP22//ZBTB4//RBBP8//TNF//ZNF217//LRP8//BHLHE41//TBL1XR1//ARID5B//BHLHE40//RUNX3//PHF14//SPRED1//IL2//LRP6//PPP1R15B//SLIT2//CBL//SOCS5//BAHD1//GADD45A//FLRT1//NCK1//DBNDD2//ATR//SNIP1//CALM2//A2ML1//WFDC13//SPOCK1//ELK3//EREG//FOXF2//MDFIC//IRF1//RASD1//RFX3//ATXN1//TSC22D4//BTAF1//RPS6KA5//FMR1//TSC1//EGLN1//SPOPL//SOX4//CDK6//ATAD2//MIER1//DCP2//MTM1//PHACTR2//CNOT6//ITM2A//ARL6IP1//PTPN22//NMI//CASP3//MYOG//PRKAA1//ZMYND11//INHBB//NTF3//SAMSN1//LDLR//PMEPA1//LDLRAD4//POLR2K//CNOT6L//YTHDF2//QKI//MYD88//PLAA//CPEB2//NCOA7//TFEC//NPAT//SRSF2//TNKS//GJA1//BMPR2//PRKAR2B//CPEB1// |
| GO:0003006 | developmental process involved in reproduction | Biological process | 40 | 669 | 574 | 17653 | 1.83882543501924 | 0.000162309666892649 | 0.0179950865113932 | 3.78965561354955 | 0.0696864111498258 | CEBPB//ADCYAP1//SPO11//INHBB//DMRTA1//SOD1//ESR1//EREG//YTHDF2//AGFG1//PPARG//GATA2//BMPR2//ACVR1//TDRD5//STRBP//QKI//ZFPM2//AR//WNT7A//RNF38//TEX11//ARID5B//CBL//MGST1//SYCP1//SYCP3//NEUROG1//LRP6//GJA1//GJB2//PAQR5//FOXF2//IGF1//ZNF568//FOSL1//SULF1//EGLN1//SLIT2//ARID4B// |
| GO:0051130 | positive regulation of cellular component organization | Biological process | 63 | 1221 | 574 | 17653 | 1.58683406244382 | 0.000179712585486782 | 0.0193064056887956 | 3.74542150766527 | 0.109756097560976 | FMR1//NTF3//CNOT6L//PAN3//CNOT6//STMN2//ADCYAP1//NPTN//NCK1//RAPGEF2//MET//CLIP1//IFNG//TNF//NEUROG1//MIER1//ATR//TNKS//CCT6A//MIEF2//RPS6KA5//IQGAP2//ARFIP1//NOS1//BNIP3//AR//DLL1//LDLR//EDN1//EREG//IGF1//BTC//ESR1//CBL//EFNA5//BMPR2//GATA2//SLIT2//TSC1//MYB//JARID2//FLRT1//IL1RAP//WNT7A//DLG5//NRXN1//PPARG//CPEB3//IL2//LRP8//IL5//CLASP2//DDHD2//CRKL//DYNLL2//CALCOCO2//MYOG//RAB3GAP1//PLAA//PRKAA1//SNIP1//FOSL1//CCL2// |
| GO:0060284 | regulation of cell development | Biological process | 48 | 858 | 574 | 17653 | 1.72052337905996 | 0.00017984674824695 | 0.0193064056887956 | 3.74509741011777 | 0.0836236933797909 | FZD3//FRZB//SNAP25//STMN2//ADCYAP1//NPTN//NCK1//RAPGEF2//PTPRG//SPOCK1//TSC1//DPYSL2//WNT7A//DLL1//IL5//NTF3//DDX6//GATA2//VWC2//NEUROD1//NEUROG1//ULK2//EFNA5//PPARG//MYOG//FMR1//HECW2//BMPR2//NOS1//POU4F1//POU3F2//SLIT2//MYB//IGF1//EDN1//CPEB3//IL2//DLG5//LRP8//AKAP6//LDLR//IL1RAP//CRKL//TNF//YTHDF2//PLAA//RFX3//GPR183// |
| GO:0009890 | negative regulation of biosynthetic process | Biological process | 86 | 1809 | 574 | 17653 | 1.46206443585402 | 0.000195721735123557 | 0.0206822827287596 | 3.70836094276267 | 0.149825783972125 | TSHZ1//ZBTB18//CEBPB//ZNF268//TCERG1//STRAP//KLF12//ARX//EDN1//CC2D1B//EN1//ESR1//CPEB3//MYT1L//RCOR1//ZFPM2//GATA2//CNOT7//HOXC8//HSBP1//IFNG//IRF2//AR//JARID2//ZNF568//MAF//MDM4//MYB//NFIA//NR4A2//KLF3//POU4F1//PPARG//DUSP22//ZBTB4//RBBP8//TNF//ZNF217//LRP8//BHLHE41//TBL1XR1//ARID5B//BHLHE40//RUNX3//PHF14//BAHD1//ATR//SNIP1//ELK3//EREG//FOXF2//MDFIC//IRF1//RASD1//RFX3//ATXN1//TSC22D4//BTAF1//RPS6KA5//FMR1//TSC1//EGLN1//ATAD2//MIER1//DCP2//CNOT6//ITM2A//NMI//SOD1//MYOG//PRKAA1//RAPGEF2//INHBB//POLR2K//CNOT6L//YTHDF2//QKI//CPEB2//ZMYND11//TFEC//NPAT//SRSF2//NCK1//GJA1//BMPR2//CPEB1// |
| GO:0007050 | cell cycle arrest | Biological process | 20 | 250 | 574 | 17653 | 2.46034843205575 | 0.000205381362959254 | 0.0209601228037486 | 3.68743896838812 | 0.0348432055749129 | GADD45A//CNOT6L//CNOT7//MDM4//CNOT6//SOX4//NEUROD1//ZNF268//MYOG//POU4F1//CDC14A//CDK6//HBP1//MTBP//IFNG//IRF1//PRKAA1//RRAGD//TSC1//LAMTOR3// |
| GO:0007420 | brain development | Biological process | 41 | 700 | 574 | 17653 | 1.80132653061224 | 0.000208079879060471 | 0.0209601228037486 | 3.68176991323138 | 0.0714285714285714 | GATA2//C5AR1//POU4F1//RFX4//LRP8//CDK6//EMX2//NEUROD1//EN1//PTF1A//LRP6//ANP32B//RAPGEF2//DLL1//AGTR2//MTPN//WNT7A//CNTNAP2//HPRT1//ARX//CALM2//TSC1//CASP3//POU3F2//SLIT2//RAB3GAP1//ADCYAP1//NR4A2//TACC2//BNIP3//OTX2//DLG5//FZD3//FZD6//DPYSL2//AK4//SPHK2//PTPRG//ATXN1//BMPR2//TGFBR2// |
| GO:0030182 | neuron differentiation | Biological process | 66 | 1303 | 574 | 17653 | 1.55778008936761 | 0.000211579247293113 | 0.0209601228037486 | 3.67452693223269 | 0.114982578397213 | SOX4//OGN//POU4F1//WNT7A//SLIT2//ARX//DOK1//DPYSL2//EFNA5//OTX2//PIK3CB//ENAH//KLF7//RPS6KA5//NRXN1//CNTNAP2//SNAP25//STMN2//ADCYAP1//NPTN//NCK1//RAPGEF2//PTPRG//SPOCK1//TSC1//FLRT1//GATA2//MTPN//HPRT1//NR4A2//UBE4B//LRP12//DBNDD2//PSD//PTF1A//NEUROD1//FZD3//DLL1//NTF3//DDX6//VWC2//NEUROG1//MYT1L//POU3F2//EN1//FMR1//ULK2//PPP3CB//NYAP1//GJA1//HECW2//BMPR2//RUNX3//SOD1//CPEB3//IL2//DLG5//LRP8//LRP6//IL1RAP//PLAA//CEBPB//EMX2//HOXC8//HOXD1//CASP3// |
| GO:0031327 | negative regulation of cellular biosynthetic process | Biological process | 85 | 1789 | 574 | 17653 | 1.4612186747117 | 0.000218335519412797 | 0.0209601228037486 | 3.66087560634241 | 0.14808362369338 | TSHZ1//ZBTB18//CEBPB//ZNF268//TCERG1//STRAP//KLF12//ARX//EDN1//CC2D1B//EN1//ESR1//CPEB3//MYT1L//RCOR1//ZFPM2//GATA2//CNOT7//HOXC8//HSBP1//IFNG//IRF2//AR//JARID2//ZNF568//MAF//MDM4//MYB//NFIA//NR4A2//KLF3//POU4F1//PPARG//DUSP22//ZBTB4//RBBP8//TNF//ZNF217//LRP8//BHLHE41//TBL1XR1//ARID5B//BHLHE40//RUNX3//PHF14//BAHD1//ATR//SNIP1//ELK3//EREG//FOXF2//MDFIC//IRF1//RASD1//RFX3//ATXN1//TSC22D4//BTAF1//RPS6KA5//FMR1//TSC1//EGLN1//ATAD2//MIER1//DCP2//CNOT6//ITM2A//NMI//MYOG//PRKAA1//RAPGEF2//INHBB//POLR2K//CNOT6L//YTHDF2//QKI//CPEB2//ZMYND11//TFEC//NPAT//SRSF2//NCK1//GJA1//BMPR2//CPEB1// |
| GO:0031324 | negative regulation of cellular metabolic process | Biological process | 121 | 2748 | 574 | 17653 | 1.35417649326213 | 0.000221815102233246 | 0.0209601228037486 | 3.65400888833438 | 0.210801393728223 | TSHZ1//ZBTB18//CEBPB//ZNF268//TCERG1//STRAP//KLF12//ARX//EDN1//CC2D1B//EN1//ESR1//CPEB3//MYT1L//RCOR1//ZFPM2//GATA2//CNOT7//HOXC8//HSBP1//IFNG//IRF2//AR//JARID2//ZNF568//MAF//MDM4//MYB//NFIA//NR4A2//KLF3//POU4F1//PPARG//DUSP22//ZBTB4//RBBP8//TNF//ZNF217//LRP8//BHLHE41//TBL1XR1//ARID5B//BHLHE40//RUNX3//PHF14//SPRED1//IL2//LRP6//PPP1R15B//SLIT2//CBL//SOCS5//BAHD1//GADD45A//FLRT1//NCK1//DBNDD2//ATR//SNIP1//PTPN22//MET//MTMR9//BNIP3//CALM2//DLG2//PLEKHF2//A2ML1//WFDC13//SPOCK1//TSC1//ELK3//EREG//FOXF2//MDFIC//IRF1//RASD1//RFX3//ATXN1//TSC22D4//BTAF1//RPS6KA5//FMR1//EGLN1//SPOPL//SOX4//CDK6//ATAD2//MIER1//DCP2//MTM1//PHACTR2//CNOT6//ITM2A//ARL6IP1//NMI//CASP3//MYOG//PRKAA1//ZMYND11//RAPGEF2//INHBB//NTF3//SAMSN1//PMEPA1//LDLRAD4//POLR2K//CNOT6L//YTHDF2//QKI//MYD88//PLAA//CPEB2//NCOA7//TFEC//NPAT//SRSF2//TNKS//GJA1//BMPR2//PRKAR2B//CPEB1// |
| GO:0009896 | positive regulation of catabolic process | Biological process | 27 | 393 | 574 | 17653 | 2.1128946458494 | 0.000223778139842987 | 0.0209601228037486 | 3.65018234054786 | 0.0470383275261324 | IFNG//PIK3CB//PRKAA1//BNIP3//TRIM8//RAB12//TSC1//RNF180//RNF125//LONRF3//LONRF1//SOCS5//GJA1//HECW2//TNF//GPD1//IGF1//SH3D19//CPEB3//CNOT7//CNOT6L//YTHDF2//QKI//CALCOCO2//FMR1//LDLR//RAB3GAP1// |
| GO:0044260 | cellular macromolecule metabolic process | Biological process | 320 | 8555 | 574 | 17653 | 1.1503674726152 | 0.000224095493885297 | 0.0209601228037486 | 3.64956687617545 | 0.557491289198606 | RAD9B//IGF1//NFIA//ATR//WRNIP1//RBBP8//RRM2//DTD1//GADD45A//DIRAS3//NPAT//TSHZ1//ZBTB18//CEBPB//ZNF268//TCERG1//STRAP//KLF12//ARX//EDN1//CC2D1B//EN1//ESR1//CPEB3//MYT1L//RCOR1//ZFPM2//GATA2//CNOT7//HOXC8//HSBP1//IFNG//IRF2//AR//JARID2//ZNF568//MAF//MDM4//MYB//NR4A2//KLF3//POU4F1//PPARG//DUSP22//ZBTB4//TNF//ZNF217//LRP8//BHLHE41//TBL1XR1//ARID5B//BHLHE40//RUNX3//PHF14//CSF2RA//SPRED1//EREG//RASGEF1A//IL2//IL5//MAP3K1//MET//MAP3K7CL//CCL2//BTC//LAMTOR3//RAPGEF2//DCP2//CRKL//NTF3//PIK3CB//PRKAA1//SOD1//C5AR1//UBE4B//RNF145//ZNRF2//RNF180//RNF125//UBE2W//HECW2//SKP1//THOP1//UBE2G1//LONRF3//CBFB//TNKS//CBL//UBE3B//LONRF1//CNOT6L//PAN3//CNOT6//SPO11//SYCP1//TEX11//PTGES3//NABP1//SECISBP2L//CAMLG//EPS15//LRP6//PPP1R15B//SLIT2//EFNA5//FMR1//NPTN//ELK3//TFEC//FOXJ3//FOXF2//PTF1A//HIVEP2//MACC1//IRF1//MEOX2//MYBL1//NEUROD1//NHLH2//OTX2//POLR2K//POU3F2//RFX4//BMPR2//SOX4//BTF3//CARF//FOSL1//KLF7//NMI//SRSF2//SOCS5//RNF38//KLHL3//SPOPL//DCAF8//TRIM8//KBTBD8//GMNC//ESCO2//EPC2//USP28//UNG//KDM2A//MTRR//CASP3//BAHD1//ZMYND11//KLF17//NCOA7//ZNF782//ZNF800//ZFP30//HBP1//ATAD2//MDFIC//HOXA3//HOXB6//HOXD1//ZNF680//NEUROG1//NFE2//COMMD2//VGLL1//ARID4B//BRWD1//TRERF1//SLC2A4RG//ZNF630//ZBTB26//MIER1//RFX3//ATXN1//DMRTA1//SMARCD2//ZNF3//ZNF131//ZNF134//ZNF208//ZNF227//ZNF655//MED28//TSC22D4//PCGF5//LCOR//ST18//NRBF2//MLLT6//PAK6//BTG1//RHOXF2B//SNIP1//RPS6KA5//MED6//EMX2//BOLA3//NR3C2//RFX7//RHOXF2//BLZF1//CPEB2//MRPL35//CPEB1//METTL17//EIF3B//MTPN//DDX6//TSC1//AIMP1//YES1//IMMP1L//CDK6//FASTK//PDIK1L//CDK19//PPP3CB//CCL8//TGFBR2//ACVR1//FLRT1//NCK1//DBNDD2//PTPN22//MTM1//CPPED1//HS3ST5//FUT9//FUT2//ST8SIA5//MAN1A1//ST8SIA3//CCDC126//TET2//VEGFB//PIGP//PIGA//GPHN//ANP32B//EGLN3//DLL1//G3BP2//CALM2//INHBB//BMP3//A2ML1//WFDC13//SPOCK1//NDST3//PRR16//PIGS//GNPTG//AGTR2//NOS1//WNT7A//RASD1//BTAF1//OTUD3//WDR20//NFATC2IP//VKORC1L1//STK33//EGLN1//DCUN1D4//MSRB3//SULF1//DSEL//SLC35D1//CHST7//IDS//VWC2//CDC27//RAP2C//CCT6A//PHACTR2//AKAP6//IFNA1//PRKAR2B//PTPRG//PTP4A1//CDC14A//BNIP3//DCTPP1//MYOG//INTS7//ITM2A//TDRD5//ARL6IP1//UBXN2B//CD2AP//PLAA//VPS37A//FZD6//YTHDF2//ARSK//RAB12//CCNY//ADCYAP1//ARMCX3//GRIK2//ULK2//SAMSN1//SH3D19//IL1RAP//MYD88//ARRDC3//PMEPA1//LDLRAD4//QKI//ATG16L1//CCR1//GPR183//NPNT//TNFAIP8L3//UBA5//ACSL1//GLRX//SNAP25//RAB3GAP1//LDLR//GJA1// |
| GO:0051253 | negative regulation of RNA metabolic process | Biological process | 66 | 1306 | 574 | 17653 | 1.55420172775344 | 0.000225661259510531 | 0.0209601228037486 | 3.64654299220678 | 0.114982578397213 | TSHZ1//ZBTB18//CEBPB//ZNF268//TCERG1//STRAP//KLF12//ARX//EDN1//CC2D1B//EN1//ESR1//CPEB3//MYT1L//RCOR1//ZFPM2//GATA2//CNOT7//HOXC8//HSBP1//IFNG//IRF2//AR//JARID2//ZNF568//MAF//MDM4//MYB//NFIA//NR4A2//KLF3//POU4F1//PPARG//DUSP22//ZBTB4//RBBP8//TNF//ZNF217//LRP8//BHLHE41//TBL1XR1//ARID5B//BHLHE40//RUNX3//PHF14//BAHD1//ELK3//EREG//FOXF2//MDFIC//IRF1//RASD1//RFX3//ATXN1//TSC22D4//BTAF1//RPS6KA5//ATAD2//MIER1//MYD88//FMR1//ZMYND11//TFEC//NPAT//SRSF2//NCK1// |
| GO:0050794 | regulation of cellular process | Biological process | 400 | 11074 | 574 | 17653 | 1.11086709050738 | 0.000228442397858501 | 0.0209601228037486 | 3.64122328988684 | 0.696864111498258 | RAD9B//INTS7//ATR//USP28//GADD45A//DIRAS3//NPAT//RRM2//TSHZ1//ZBTB18//CEBPB//ZNF268//TCERG1//STRAP//KLF12//ARX//EDN1//CC2D1B//EN1//ESR1//CPEB3//MYT1L//RCOR1//ZFPM2//GATA2//CNOT7//HOXC8//HSBP1//IFNG//IRF2//AR//JARID2//ZNF568//MAF//MDM4//MYB//NFIA//NR4A2//KLF3//POU4F1//PPARG//DUSP22//ZBTB4//RBBP8//TNF//ZNF217//LRP8//BHLHE41//TBL1XR1//ARID5B//BHLHE40//RUNX3//PHF14//CSF2RA//SPRED1//EREG//RASGEF1A//IL2//IL5//MAP3K1//MET//MAP3K7CL//CCL2//BTC//LAMTOR3//RAPGEF2//CRKL//IGF1//NTF3//PIK3CB//PRKAA1//SOD1//C5AR1//FMR1//MBNL1//SMU1//RBM25//SRSF2//SECISBP2L//SPOCK1//BLZF1//BCL2A1//EPS15//LRP6//PPP1R15B//SLIT2//EFNA5//NPTN//SULF1//GJA1//AIMP1//BMPR2//VEGFB//CBFB//AGTR2//FZD3//TGFBR2//SKP1//RPS6KA5//MYD88//CCL8//REG3G//SPHK2//IRF1//TSC1//APCDD1//CCNY//HBP1//WNT7A//TNKS//PPP3CB//FZD6//CDK6//CDS1//PTGES3//IQGAP2//ARAP2//CLCN6//DOK1//DPYSL2//ELK3//HTR3D//EVI2A//CD2AP//APPL1//OSTF1//GLRB//LRP12//HIVEP2//NR3C2//HPCAL4//RASD1//KCNK10//PSD//ARRDC3//MIER1//SMOC1//SMOC2//CALCB//RERGL//CAMLG//OR6A2//CBL//AKAP4//DLG5//PLAA//ARHGAP12//CD69//ULK2//PTPRG//YES1//CD8A//AKAP11//DGKH//DCX//ARHGEF4//TREM1//PRKAR2B//STK33//DGKE//AKAP6//SOCS5//NLN//DNAJC16//GLRX//NHLRC2//NOS1//ESCO2//BAHD1//ZMYND11//ZNF782//ZNF800//ZFP30//KDM2A//CNOT6L//PTF1A//NRBF2//ZNF680//MLLT6//COMMD2//VGLL1//SLC2A4RG//PAK6//ZNF630//CNOT6//ZBTB26//RFX3//SOX4//BTF3//BTG1//RHOXF2B//ZNF3//ZNF131//ZNF134//ZNF208//ZNF227//ZNF655//SNIP1//MED28//POLR2K//MED6//KLF17//EMX2//FOXJ3//FOXF2//EPC2//ATAD2//HOXA3//HOXB6//HOXD1//BOLA3//MYBL1//NEUROG1//NFE2//NHLH2//ARID4B//BRWD1//POU3F2//TRERF1//RFX4//DMRTA1//RFX7//SMARCD2//FOSL1//TSC22D4//LCOR//RHOXF2//KLF7//MTPN//DDX6//KBTBD8//EIF3B//FLRT1//NCK1//DBNDD2//ANP32B//EGLN3//CASP3//STMN2//CLASP2//MID1IP1//MTBP//RRAGD//CDC27//NABP1//CDC14A//CCR1//ACVR1//RAPGEF4//CHRM2//ADCYAP1//GPR183//FRZB//CCL28//PTGFR//SOS2//OR13A1//CALM2//GRIK2//DLL1//G3BP2//MDFIC//NMI//NEUROD1//ARHGAP1//RALGPS2//CDK19//TRPC3//SNAP25//ARL6IP1//ZFAND6//TEX11//BNIP3//UNG//TES//MYOG//ST18//CFDP1//OTX2//UBE4B//SAMSN1//SH3BGR//SH3BGRL//C1QTNF9//IFNA1//MACC1//INHBB//OGN//IL36G//BMP3//DRAM1//PTPN22//MTMR9//TRIM8//PAN3//NPNT//LDLRAD4//C5ORF30//CHMP3//LDLR//DLG2//PLEKHF2//A2ML1//WFDC13//PMEPA1//NYAP1//PRR16//RAB12//SNX5//TNFAIP8L3//ATXN1//BTAF1//SYT6//IL1RAP//PLP2//IL20RA//SH3D19//DENND1B//HECW2//WRNIP1//SHISA2//H3F3C//EI24//FAM110C//PTP4A1//RAP2C//VWC2//EGLN1//MMRN2//CLIP1//EVI5//USP6NL//SPOPL//MTM1//AGFG1//DCP2//CCT6A//RNF180//RNF125//LONRF3//LONRF1//MIEF2//KIF13A//RAB30//RAB9B//RAB6B//RAB28//PHACTR2//MEMO1//FRMD6//CD84//UBA5//ACSL1//ARFIP1//GSTA2//CA1//SYBU//ITM2A//ZMAT3//PAQR5//FASTK//YTHDF2//UBXN2B//PEX5L//GPD1//NCOA7//TFEC//MEOX2//ARMCX3//TET2//PCGF5//NFATC2IP//HPRT1//KCNJ10//RAB3GAP1//PXK//GCSAML//DCUN1D4//TMOD1//OTUD3//NRXN1//XAF1//SLC30A7//SLC30A5//QKI//CARF//FIBIN//DDHD2//SIVA1//DSC2//PATE1//HSPB8//CPEB2//DYNLL2//CALCOCO2//TMED10//SERINC3//KCNS2//IER3IP1//CPEB1// |
| GO:0010646 | regulation of cell communication | Biological process | 149 | 3522 | 574 | 17653 | 1.30107863563425 | 0.000229343351689693 | 0.0209601228037486 | 3.63951384487723 | 0.259581881533101 | GADD45A//TNF//CRKL//MAP3K1//MAP3K7CL//LAMTOR3//IGF1//NTF3//PIK3CB//PRKAA1//SOD1//C5AR1//SPRED1//DUSP22//CBFB//AGTR2//EREG//CBL//SOCS5//NCK1//MDFIC//RAPGEF4//IFNG//NEUROD1//PPP3CB//RFX3//SNAP25//ADCYAP1//OTX2//SAMSN1//ARHGAP1//SH3BGR//SH3BGRL//MET//ACVR1//EDN1//C1QTNF9//IFNA1//MACC1//IL2//IL5//INHBB//OGN//IL36G//CCL28//CCL2//CCL8//BMP3//BTC//VEGFB//WNT7A//CALCB//AIMP1//GJA1//AKAP11//AKAP4//AKAP6//BMPR2//PMEPA1//LDLRAD4//RFX4//ESR1//SULF1//SOX4//APCDD1//FRZB//SHISA2//NPNT//STRAP//TGFBR2//VWC2//MMRN2//MTM1//TSC1//RRAGD//ARAP2//APPL1//AGFG1//PSD//RALGPS2//UBA5//AR//IRF1//PTPN22//ARHGEF4//SOS2//DLG5//SYBU//RNF125//EPS15//CNOT7//ZFAND6//MYD88//MIER1//TRIM8//ZMYND11//PAK6//ATR//PEX5L//RAPGEF2//DOK1//NPTN//GATA2//YTHDF2//DLL1//GRIK2//FLRT1//RASGEF1A//FMR1//CPEB3//KCNJ10//RAB3GAP1//PXK//LRP8//GCSAML//ARHGAP12//CD2AP//SLIT2//TNFAIP8L3//FAM110C//OTUD3//NRXN1//NOS1//CALM2//PPARG//CCNY//LRP6//EFNA5//CCR1//GPR183//ARRDC3//FZD6//BNIP3//TBL1XR1//TNKS//PATE1//DYNLL2//RAD9B//MDM4//POU4F1//RBBP8//SERINC3//PPP1R15B//PHF14//MYB//NR4A2//BCL2A1//IL20RA//ST18// |
| GO:0010033 | response to organic substance | Biological process | 138 | 3218 | 574 | 17653 | 1.3188629724351 | 0.000234255451694699 | 0.0211235949308167 | 3.63031029323261 | 0.240418118466899 | CBFB//HPRT1//NR4A2//SOD1//DNAJB4//DUSP22//TGFBR2//CBL//ACVR1//CHRM2//APPL1//SPRED1//FLRT1//POLR2K//STEAP2//NOS1//SNRPN//GJA1//IGF1//NEUROD1//CASP3//SLC2A5//PPARG//SNIP1//MGST1//TNF//ACSL1//TET2//CCR1//CRKL//CSF2RA//EREG//IFNA1//IL1RAP//IL2//IL5//PIK3CB//PLP2//IL20RA//IL36G//CCL2//LRP8//SOCS5//CEBPB//CPEB3//RRAGD//CPEB1//LAMTOR3//UBE4B//BMP3//BMPR2//NPNT//STRAP//PMEPA1//LDLRAD4//SULF1//VWC2//ESR1//AR//MMRN2//PRKAA1//PTPN22//GJB2//PTGFR//RBBP8//WNT7A//C5AR1//EDN1//FOSL1//TSC1//CPEB2//INHBB//SLIT2//UBA5//SRSF2//TRPC3//ST18//GATA2//PPP3CB//CALCOCO2//XAF1//IRF1//GSTA2//IFNG//CA1//SYBU//YES1//BRWD1//AGTR2//NTF3//RAPGEF2//OTX2//SHISA2//GLRB//TMED10//NR3C2//PAQR5//REG3G//LRP6//BTG1//ADCYAP1//NPTN//NCK1//IRF2//TRIM8//NMI//MYD88//CNOT7//EFNA5//NRXN1//CCL8//SKP1//RPS6KA5//UBE2W//CDK19//PLAA//MYB//MYOG//POU3F2//GPD1//AKAP6//SOX4//POU4F1//RAB12//ZFAND6//PRKAR2B//LDLR//FIBIN//RUNX3//FMR1//DLL1//KLF3//PPP1R15B//STMN2//DYNC1LI2//JARID2//PIGA//ARID5B// |
| GO:0007548 | sex differentiation | Biological process | 21 | 273 | 574 | 17653 | 2.36571964620745 | 0.000246590248292823 | 0.0218682772574751 | 3.60802410209696 | 0.0365853658536585 | CEBPB//ADCYAP1//SPO11//INHBB//DMRTA1//SOD1//ESR1//EREG//ZFPM2//RNF38//TEX11//ARID5B//CBL//AR//MGST1//NEUROG1//LRP6//FOXF2//SLIT2//ARID4B//WNT7A// |
| GO:0016236 | macroautophagy | Biological process | 22 | 293 | 574 | 17653 | 2.30920074681 | 0.000248980829339876 | 0.0218682772574751 | 3.60383409078548 | 0.0383275261324042 | UBXN2B//STX12//ATG16L1//ULK2//RAB12//BNIP3//TSC1//SNX5//PRKAA1//RRAGD//CASP3//LAMTOR3//WDFY3//CHMP3//CALCOCO2//MTM1//RAB3GAP1//VPS37A//DYNLL2//PLEKHF2//PLAA//EI24// |
| GO:0031399 | regulation of protein modification process | Biological process | 85 | 1800 | 574 | 17653 | 1.45228900503291 | 0.000266721501324083 | 0.0231261219673689 | 3.573941972949 | 0.14808362369338 | GADD45A//DIRAS3//TNF//CRKL//MAP3K1//MAP3K7CL//LAMTOR3//IGF1//NTF3//PIK3CB//PRKAA1//SOD1//C5AR1//SPRED1//DUSP22//IL2//LRP6//PPP1R15B//SLIT2//EFNA5//FMR1//NPTN//IFNG//EREG//CBL//SOCS5//FLRT1//NCK1//DBNDD2//MDFIC//CALM2//INHBB//BMP3//BMPR2//ACVR1//JARID2//SPOPL//SOX4//CDK6//RAP2C//PAK6//TGFBR2//PHACTR2//AGTR2//AKAP6//RPS6KA5//IFNA1//PRKAR2B//GATA2//NOS1//CNOT7//EDN1//AR//MET//PTPN22//UBXN2B//TSC1//CASP3//CCNY//ADCYAP1//RAPGEF2//GRIK2//ZMYND11//WNT7A//IL5//YES1//LRP8//SAMSN1//DCUN1D4//ARRDC3//MYB//STRAP//PMEPA1//LDLRAD4//CCR1//GPR183//NPNT//TNFAIP8L3//CCL2//CCL8//ACSL1//PLAA//NCOA7//NMI//RAB3GAP1// |
| GO:0070887 | cellular response to chemical stimulus | Biological process | 136 | 3175 | 574 | 17653 | 1.31735191637631 | 0.000277909181572618 | 0.0237765574632119 | 3.55609710475033 | 0.236933797909408 | CBFB//CCR1//GPR183//CCL2//CCL8//SLIT2//PTGES3//MGST1//NAT1//DUSP22//TGFBR2//CBL//ACVR1//CHRM2//APPL1//SPRED1//FLRT1//POLR2K//PPARG//SNIP1//C5AR1//TNF//CRKL//CSF2RA//EREG//IFNA1//IL1RAP//IL2//IL5//PIK3CB//PLP2//IL20RA//IL36G//LRP8//CASP3//SOCS5//SOD1//CEBPB//CPEB3//RRAGD//CPEB1//LAMTOR3//BMP3//BMPR2//NPNT//STRAP//PMEPA1//LDLRAD4//SULF1//VWC2//ESR1//AR//MMRN2//PTPN22//CPEB2//INHBB//UBA5//ST18//ACSL1//VKORC1L1//GJB2//NR4A2//IRF1//EDN1//PPP3CB//CD69//GSTA2//IFNG//CA1//SYBU//CARF//YES1//BRWD1//AGTR2//NTF3//RAPGEF2//OTX2//SHISA2//GSTM3//NR3C2//PAQR5//NPTN//NCK1//TSC1//IGF1//CCL28//IRF2//TRIM8//NMI//MYD88//XAF1//CNOT7//VEGFB//EFNA5//NRXN1//EGLN3//EGLN1//MYB//PRKAA1//BNIP3//SKP1//RPS6KA5//UBE2W//CDK19//PLAA//MYOG//SLC41A1//POU3F2//GPD1//AKAP6//SLC2A5//NEUROD1//SOX4//POU4F1//RAB12//ZFAND6//NOS1//GJA1//PRKAR2B//ADCYAP1//LRP6//LDLR//MDM4//WNT7A//PTGFR//FMR1//DLL1//MET//KLF3//NCOA7//PPP1R15B//STMN2//DYNC1LI2//JARID2//PIGA//ARID5B// |
| GO:0008585 | female gonad development | Biological process | 11 | 96 | 574 | 17653 | 3.52393655632985 | 0.000282229864021947 | 0.0237765574632119 | 3.54939703348132 | 0.0191637630662021 | CEBPB//ADCYAP1//SPO11//INHBB//DMRTA1//SOD1//ESR1//EREG//SLIT2//ZFPM2//ARID5B// |
| GO:0071310 | cellular response to organic substance | Biological process | 117 | 2656 | 574 | 17653 | 1.35476640884094 | 0.000284770243164301 | 0.0237765574632119 | 3.54550539391412 | 0.20383275261324 | CBFB//DUSP22//TGFBR2//CBL//ACVR1//CHRM2//APPL1//SPRED1//FLRT1//POLR2K//PPARG//SNIP1//TNF//CCR1//CRKL//CSF2RA//EREG//IFNA1//IL1RAP//IL2//IL5//PIK3CB//PLP2//IL20RA//IL36G//CCL2//LRP8//CASP3//SOCS5//CEBPB//CPEB3//RRAGD//CPEB1//LAMTOR3//BMP3//BMPR2//NPNT//STRAP//PMEPA1//LDLRAD4//SULF1//VWC2//ESR1//AR//MMRN2//PTPN22//CPEB2//INHBB//SLIT2//UBA5//ST18//ACSL1//IRF1//GSTA2//IFNG//SOD1//CA1//PPP3CB//SYBU//YES1//BRWD1//AGTR2//NTF3//RAPGEF2//OTX2//SHISA2//NR3C2//NR4A2//PAQR5//NPTN//NCK1//TSC1//IGF1//IRF2//TRIM8//NMI//MYD88//XAF1//CNOT7//EFNA5//NRXN1//CCL8//EDN1//SKP1//RPS6KA5//UBE2W//CDK19//PLAA//MYB//MYOG//GPD1//AKAP6//SLC2A5//NEUROD1//SOX4//POU4F1//RAB12//ZFAND6//PRKAA1//NOS1//GJA1//GJB2//PRKAR2B//ADCYAP1//LRP6//LDLR//WNT7A//PTGFR//DLL1//KLF3//PPP1R15B//STMN2//DYNC1LI2//JARID2//PIGA//ARID5B//POU3F2// |
| GO:0009058 | biosynthetic process | Biological process | 259 | 6735 | 574 | 17653 | 1.18268419432524 | 0.00031100029247979 | 0.0256499387565954 | 3.50723920254133 | 0.451219512195122 | RAD9B//IGF1//NFIA//ATR//WRNIP1//RBBP8//RRM2//DTD1//NPAT//TSHZ1//ZBTB18//CEBPB//ZNF268//TCERG1//STRAP//KLF12//ARX//EDN1//CC2D1B//EN1//ESR1//CPEB3//MYT1L//RCOR1//ZFPM2//GATA2//CNOT7//HOXC8//HSBP1//IFNG//IRF2//AR//JARID2//ZNF568//MAF//MDM4//MYB//NR4A2//KLF3//POU4F1//PPARG//DUSP22//ZBTB4//TNF//ZNF217//LRP8//BHLHE41//TBL1XR1//ARID5B//BHLHE40//RUNX3//PHF14//SYCP1//NOS1//SECISBP2L//PTGES3//MGST1//ST8SIA3//ELK3//TFEC//FOXJ3//FOXF2//PTF1A//HIVEP2//MACC1//IRF1//MEOX2//MYBL1//NEUROD1//NHLH2//OTX2//POLR2K//POU3F2//RFX4//BMPR2//SOX4//BTF3//CARF//FOSL1//KLF7//CBFB//NMI//SRSF2//HS3ST5//SLC35D1//GPD1//GPD2//MDH1//NLN//HPRT1//IMPDH1//CMPK1//DCTPP1//GMNC//ESCO2//BAHD1//ZMYND11//KLF17//NCOA7//ZNF782//ZNF800//ZFP30//KDM2A//CNOT6L//EPC2//HBP1//ATAD2//MDFIC//HOXA3//HOXB6//HOXD1//ZNF680//NEUROG1//NFE2//COMMD2//VGLL1//ARID4B//BRWD1//PRKAA1//TRERF1//SLC2A4RG//ZNF630//CNOT6//ZBTB26//MIER1//RFX3//ATXN1//DMRTA1//SMARCD2//ZNF3//ZNF131//ZNF134//ZNF208//ZNF227//ZNF655//MED28//TSC22D4//PCGF5//LCOR//ST18//NRBF2//MLLT6//PAK6//BTG1//RHOXF2B//SNIP1//CBL//RPS6KA5//MED6//EMX2//BOLA3//NR3C2//RFX7//RHOXF2//BLZF1//CPEB2//MRPL35//CPEB1//METTL17//EIF3B//MTPN//DDX6//TSC1//KBTBD8//AIMP1//FUT9//FUT2//ST8SIA5//MAN1A1//CCDC126//TET2//VEGFB//PIGP//PIGA//GLUD2//LGSN//SERINC3//DDHD2//LCLAT1//PLA2G12A//CDS1//ABHD3//SLC44A1//MTM1//PIK3CB//MTMR9//SPHK2//ACBD3//MSMO1//HSD11B1//GGT6//GPHN//G3BP2//ACSL1//DGKE//MTRR//AK4//GLT6D1//EREG//LDLR//NDST3//FMR1//PRR16//PIGS//AGTR2//IL5//LRP6//PPP3CB//WNT7A//ACVR1//RASD1//BTAF1//OGN//CHST1//ELOVL7//CHST7//DSEL//BMP3//SULF1//VWC2//CALM2//EGLN1//TNKS//DCP2//CCT6A//NCK1//IL1RAP//QKI//MYOG//C5AR1//INTS7//NABP1//ITM2A//FZD6//UNG//SOD1//MID1IP1//ADCYAP1//DLL1//IL2//MET//ARMCX3//YES1//NFATC2IP//RAPGEF2//INHBB//TRIM8//MYD88//CCL2//YTHDF2//EGLN3//ATG16L1//GSTA2//GSTM3//RAB3GAP1//RAP2C//PPP1R15B//GJA1//NPNT// |
| GO:0048634 | regulation of muscle organ development | Biological process | 14 | 147 | 574 | 17653 | 2.9289862286378 | 0.000317611162158866 | 0.0256794484270835 | 3.49810424308746 | 0.024390243902439 | MTPN//GJA1//DLL1//MTM1//PRKAA1//MYOG//JARID2//TGFBR2//ZFPM2//EDN1//IGF1//AKAP6//NLN//NEUROG1// |
| GO:0023051 | regulation of signaling | Biological process | 150 | 3575 | 574 | 17653 | 1.29039253429497 | 0.000319572306566865 | 0.0256794484270835 | 3.49543086273196 | 0.261324041811847 | GADD45A//TNF//CRKL//MAP3K1//MAP3K7CL//LAMTOR3//IGF1//NTF3//PIK3CB//PRKAA1//SOD1//C5AR1//SPRED1//DUSP22//CAMLG//EPS15//CBFB//AGTR2//EREG//CBL//SOCS5//NCK1//MDFIC//RAPGEF4//IFNG//NEUROD1//PPP3CB//RFX3//SNAP25//ADCYAP1//OTX2//SAMSN1//ARHGAP1//SH3BGR//SH3BGRL//MET//ACVR1//EDN1//C1QTNF9//IFNA1//MACC1//IL2//IL5//INHBB//OGN//IL36G//CCL28//CCL2//CCL8//BMP3//BTC//VEGFB//WNT7A//CALCB//AIMP1//AKAP11//AKAP4//AKAP6//BMPR2//PMEPA1//LDLRAD4//RFX4//ESR1//SULF1//SOX4//APCDD1//FRZB//SHISA2//NPNT//STRAP//TGFBR2//VWC2//MMRN2//MTM1//TSC1//RRAGD//ARAP2//APPL1//AGFG1//PSD//GJA1//RALGPS2//UBA5//AR//IRF1//PTPN22//ARHGEF4//SOS2//DLG5//SYBU//RNF125//CNOT7//ZFAND6//MYD88//MIER1//TRIM8//ZMYND11//PAK6//ATR//PEX5L//RAPGEF2//DOK1//NPTN//GATA2//YTHDF2//DLL1//GRIK2//FLRT1//RASGEF1A//FMR1//CPEB3//KCNJ10//RAB3GAP1//PXK//LRP8//GCSAML//ARHGAP12//CD2AP//SLIT2//TNFAIP8L3//FAM110C//OTUD3//NRXN1//NOS1//CALM2//PPARG//CCNY//LRP6//EFNA5//CCR1//GPR183//ARRDC3//FZD6//BNIP3//TBL1XR1//TNKS//PATE1//DYNLL2//RAD9B//MDM4//POU4F1//RBBP8//SERINC3//PPP1R15B//PHF14//MYB//NR4A2//BCL2A1//IL20RA//ST18// |
| GO:0009059 | macromolecule biosynthetic process | Biological process | 218 | 5526 | 574 | 17653 | 1.21325542478319 | 0.000325365990191841 | 0.0256794484270835 | 3.48762784489621 | 0.379790940766551 | RAD9B//IGF1//NFIA//ATR//WRNIP1//RBBP8//RRM2//DTD1//NPAT//TSHZ1//ZBTB18//CEBPB//ZNF268//TCERG1//STRAP//KLF12//ARX//EDN1//CC2D1B//EN1//ESR1//CPEB3//MYT1L//RCOR1//ZFPM2//GATA2//CNOT7//HOXC8//HSBP1//IFNG//IRF2//AR//JARID2//ZNF568//MAF//MDM4//MYB//NR4A2//KLF3//POU4F1//PPARG//DUSP22//ZBTB4//TNF//ZNF217//LRP8//BHLHE41//TBL1XR1//ARID5B//BHLHE40//RUNX3//PHF14//SYCP1//SECISBP2L//ELK3//TFEC//FOXJ3//FOXF2//PTF1A//HIVEP2//MACC1//IRF1//MEOX2//MYBL1//NEUROD1//NHLH2//OTX2//POLR2K//POU3F2//RFX4//BMPR2//SOX4//BTF3//CARF//FOSL1//KLF7//CBFB//NMI//SRSF2//HS3ST5//GMNC//ESCO2//BAHD1//ZMYND11//KLF17//NCOA7//ZNF782//ZNF800//ZFP30//KDM2A//CNOT6L//EPC2//HBP1//ATAD2//MDFIC//HOXA3//HOXB6//HOXD1//ZNF680//NEUROG1//NFE2//COMMD2//VGLL1//ARID4B//BRWD1//PRKAA1//TRERF1//SLC2A4RG//ZNF630//CNOT6//ZBTB26//MIER1//RFX3//ATXN1//DMRTA1//SMARCD2//ZNF3//ZNF131//ZNF134//ZNF208//ZNF227//ZNF655//MED28//TSC22D4//PCGF5//LCOR//ST18//NRBF2//MLLT6//PAK6//BTG1//RHOXF2B//SNIP1//CBL//RPS6KA5//MED6//EMX2//BOLA3//NR3C2//RFX7//RHOXF2//BLZF1//CPEB2//MRPL35//CPEB1//METTL17//EIF3B//MTPN//DDX6//TSC1//KBTBD8//AIMP1//FUT9//FUT2//ST8SIA5//MAN1A1//ST8SIA3//CCDC126//TET2//VEGFB//PIGP//PIGA//PTGES3//G3BP2//EREG//NDST3//FMR1//PRR16//PIGS//AGTR2//IL5//LRP6//NOS1//PPP3CB//WNT7A//ACVR1//RASD1//BTAF1//OGN//CHST1//SLC35D1//CHST7//DSEL//BMP3//SULF1//VWC2//TNKS//DCP2//CCT6A//NCK1//IL1RAP//MYOG//C5AR1//INTS7//NABP1//ITM2A//EGLN1//FZD6//ADCYAP1//DLL1//IL2//MET//ARMCX3//YES1//NFATC2IP//INHBB//TRIM8//MYD88//CCL2//YTHDF2//QKI//EGLN3//ATG16L1//RAB3GAP1//RAP2C//PPP1R15B//GJA1//NPNT// |
| GO:0007275 | multicellular organism development | Biological process | 212 | 5351 | 574 | 17653 | 1.21844951316534 | 0.000326546290807213 | 0.0256794484270835 | 3.48605524496434 | 0.369337979094077 | WNT7A//HAPLN1//EN1//FRZB//IGF1//BMP3//SOX4//ELK3//EREG//HOXA3//MEOX2//CCL2//VEGFB//MMRN2//AIMP1//CEBPB//ADCYAP1//SPO11//INHBB//DMRTA1//SOD1//ESR1//CRKL//SPHK2//TGFBR2//RAPGEF2//EDN1//ACVR1//ZFPM2//QKI//SMOC1//GATA2//NPNT//SLIT2//GJA1//AR//ZNF568//MBNL1//BTF3//FOSL1//LRP6//BMPR2//DLL1//DCX//LRP12//NR4A2//SPOCK1//FZD3//TMED10//SULF1//TSC1//ARID5B//RBBP8//FOXF2//DLG5//FZD6//AK4//JARID2//MET//PPARG//APCDD1//ANP32B//PPP3CB//RUNX3//ESCO2//GPR183//ITM2A//UBE4B//POU4F1//RFX3//CDK6//NEUROD1//DLG2//NEUROG1//BHLHE41//FUT9//CHRM2//DPYSL2//EFNA5//MYT1L//FMR1//GLRB//NTF3//POU3F2//ZIC5//OGN//ARX//DOK1//OTX2//PIK3CB//ENAH//KLF7//RPS6KA5//NRXN1//PCDHB10//NHLH2//HPCAL4//AGTR2//RAB3GAP1//CNTNAP2//PTPRG//ATXN1//CASP3//ZBTB18//MYOG//GSTM3//RNF38//TEX11//CBL//RAI2//TSHZ1//EMX2//HOXB6//HOXC8//CLASP2//STRAP//LDLRAD4//PTF1A//SNAP25//STMN2//NPTN//NCK1//KBTBD8//MTPN//FLRT1//UNG//SPRED1//RFX4//C5AR1//LRP8//HPRT1//CALM2//TACC2//BNIP3//KCNJ10//CBFB//TET2//HOXD1//IFNA1//PTPN22//IL2//TNF//HSD11B1//CCR1//TDRD5//IFNG//KRT27//DBNDD2//PSD//DSC2//KRTAP4-11//MAF//LRIG1//MGST1//HSBP1//SBF2//GPD2//IRF1//ARRDC3//CD8A//BTG1//REG3G//MYB//SOCS5//IL5//NFE2//DDX6//VWC2//EGLN1//ADAM12//GJB2//PHF14//YTHDF2//MTM1//PRKAA1//ULK2//SLC35D1//NYAP1//HECW2//NOS1//IL1RAP//NFIA//SYBU//TBL1XR1//BTBD7//CPEB3//AKAP6//LDLR//TMOD1//KLHL3//ARID4B//NLN//PLAA//CFDP1//WDR47//WDFY3//LCLAT1//HIVEP2//AGFG1//SHISA2//KLF3//ATR//PAQR5//STRBP//TRERF1//DUSP22//ZNF3//ZNF217//PTP4A1//EVI5//SPATA9//ADAM18// |
| GO:0010558 | negative regulation of macromolecule biosynthetic process | Biological process | 81 | 1709 | 574 | 17653 | 1.45763767551577 | 0.000337241853260706 | 0.0262157086620937 | 3.47205853276549 | 0.141114982578397 | TSHZ1//ZBTB18//CEBPB//ZNF268//TCERG1//STRAP//KLF12//ARX//EDN1//CC2D1B//EN1//ESR1//CPEB3//MYT1L//RCOR1//ZFPM2//GATA2//CNOT7//HOXC8//HSBP1//IFNG//IRF2//AR//JARID2//ZNF568//MAF//MDM4//MYB//NFIA//NR4A2//KLF3//POU4F1//PPARG//DUSP22//ZBTB4//RBBP8//TNF//ZNF217//LRP8//BHLHE41//TBL1XR1//ARID5B//BHLHE40//RUNX3//PHF14//BAHD1//ATR//SNIP1//ELK3//EREG//FOXF2//MDFIC//IRF1//RASD1//RFX3//ATXN1//TSC22D4//BTAF1//RPS6KA5//FMR1//TSC1//ATAD2//MIER1//DCP2//CNOT6//ITM2A//NMI//INHBB//POLR2K//CNOT6L//YTHDF2//QKI//CPEB2//ZMYND11//TFEC//NPAT//SRSF2//NCK1//GJA1//BMPR2//CPEB1// |
| GO:0045892 | negative regulation of transcription, DNA-templated | Biological process | 60 | 1176 | 574 | 17653 | 1.56909976534168 | 0.000345087088043919 | 0.0264257695866358 | 3.46207129019035 | 0.104529616724739 | TSHZ1//ZBTB18//CEBPB//ZNF268//TCERG1//STRAP//KLF12//ARX//EDN1//CC2D1B//EN1//ESR1//CPEB3//MYT1L//RCOR1//ZFPM2//GATA2//CNOT7//HOXC8//HSBP1//IFNG//IRF2//AR//JARID2//ZNF568//MAF//MDM4//MYB//NFIA//NR4A2//KLF3//POU4F1//PPARG//DUSP22//ZBTB4//RBBP8//TNF//ZNF217//LRP8//BHLHE41//TBL1XR1//ARID5B//BHLHE40//RUNX3//PHF14//BAHD1//ATAD2//MIER1//NCK1//ELK3//EREG//FOXF2//MDFIC//IRF1//RASD1//RFX3//ATXN1//TSC22D4//BTAF1//RPS6KA5// |
| GO:0060322 | head development | Biological process | 42 | 741 | 574 | 17653 | 1.74316184457391 | 0.000347758907764393 | 0.0264257695866358 | 3.45872173684023 | 0.0731707317073171 | DPYSL2//AGTR2//AK4//RAB3GAP1//CNTNAP2//SPHK2//PTPRG//ATXN1//BMPR2//TGFBR2//GATA2//C5AR1//POU4F1//RFX4//LRP8//CDK6//EMX2//NEUROD1//EN1//PTF1A//LRP6//ANP32B//RAPGEF2//DLL1//MTPN//WNT7A//HPRT1//ARX//CALM2//TSC1//CASP3//POU3F2//SLIT2//ADCYAP1//NR4A2//TACC2//BNIP3//OTX2//DLG5//FZD3//FZD6//ARID5B// |
| GO:0019220 | regulation of phosphate metabolic process | Biological process | 84 | 1791 | 574 | 17653 | 1.4424153286759 | 0.000361769205852423 | 0.0271849459908882 | 3.44156840347273 | 0.146341463414634 | GADD45A//DIRAS3//TNF//CRKL//MAP3K1//MAP3K7CL//LAMTOR3//IGF1//NTF3//PIK3CB//PRKAA1//SOD1//C5AR1//SPRED1//DUSP22//IL2//LRP6//PPP1R15B//SLIT2//EFNA5//FMR1//NPTN//IFNG//EREG//CBL//SOCS5//FLRT1//NCK1//DBNDD2//MDFIC//NPNT//CALM2//INHBB//BMP3//BMPR2//ACVR1//LDLR//MTMR9//DLG2//PLEKHF2//TBL1XR1//EGLN1//NOS1//CDK6//RAP2C//PAK6//TGFBR2//PHACTR2//AGTR2//AKAP6//RPS6KA5//IFNA1//MET//PRKAR2B//PTGES3//AR//CNOT7//EDN1//PTPN22//NRBF2//TNFAIP8L3//UBXN2B//TSC1//CASP3//CCNY//MYOG//GPD1//ADCYAP1//RAPGEF2//GRIK2//ZMYND11//WNT7A//IL5//YES1//LRP8//SAMSN1//STRAP//PMEPA1//LDLRAD4//CCR1//GPR183//CCL2//CCL8//ACSL1// |
| GO:0033962 | cytoplasmic mRNA processing body assembly | Biological process | 5 | 20 | 574 | 17653 | 7.68858885017422 | 0.000369066162466794 | 0.0274285105138783 | 3.43289577091814 | 0.00871080139372822 | CNOT6L//PAN3//CNOT6//DDX6//CNOT7// |
| GO:0001541 | ovarian follicle development | Biological process | 8 | 55 | 574 | 17653 | 4.47336078555591 | 0.000376651812845289 | 0.0276880022855727 | 3.42405993794383 | 0.0139372822299652 | ESR1//EREG//CEBPB//ADCYAP1//SPO11//INHBB//DMRTA1//SOD1// |
| GO:0050789 | regulation of biological process | Biological process | 418 | 11709 | 574 | 17653 | 1.09790080771127 | 0.000397972565657317 | 0.0289407361455961 | 3.40014686509792 | 0.728222996515679 | RAD9B//INTS7//ATR//USP28//GADD45A//DIRAS3//NPAT//RRM2//TSHZ1//ZBTB18//CEBPB//ZNF268//TCERG1//STRAP//KLF12//ARX//EDN1//CC2D1B//EN1//ESR1//CPEB3//MYT1L//RCOR1//ZFPM2//GATA2//CNOT7//HOXC8//HSBP1//IFNG//IRF2//AR//JARID2//ZNF568//MAF//MDM4//MYB//NFIA//NR4A2//KLF3//POU4F1//PPARG//DUSP22//ZBTB4//RBBP8//TNF//ZNF217//LRP8//BHLHE41//TBL1XR1//ARID5B//BHLHE40//RUNX3//PHF14//CSF2RA//SPRED1//EREG//RASGEF1A//IL2//IL5//MAP3K1//MET//MAP3K7CL//CCL2//BTC//LAMTOR3//RAPGEF2//DCP2//CRKL//IGF1//NTF3//PIK3CB//PRKAA1//SOD1//C5AR1//CNOT6L//PAN3//CNOT6//FMR1//MBNL1//SMU1//RBM25//SRSF2//SECISBP2L//SPOCK1//BLZF1//RPS6KA5//BCL2A1//CAMLG//PPP3CB//EPS15//LRP6//PPP1R15B//SLIT2//EFNA5//NPTN//SULF1//GJA1//AIMP1//BMPR2//VEGFB//TSC1//CDK6//CCL28//CBFB//AGTR2//CALM2//NOS1//ARL6IP1//FZD3//TGFBR2//SKP1//MYD88//CCL8//REG3G//PGC//IRF1//SAMSN1//ADCYAP1//SPHK2//APCDD1//CCNY//HBP1//WNT7A//TNKS//FZD6//CDS1//PTGES3//IQGAP2//ARAP2//CLCN6//DOK1//DPYSL2//ELK3//HTR3D//EVI2A//CD2AP//APPL1//OSTF1//GLRB//LRP12//HIVEP2//NR3C2//HPCAL4//RASD1//KCNK10//PSD//ARRDC3//MIER1//SMOC1//SMOC2//CALCB//RERGL//OR6A2//CBL//AKAP4//DLG5//PLAA//ARHGAP12//CD69//ULK2//PTPRG//YES1//CD8A//AKAP11//DGKH//DCX//ARHGEF4//TREM1//PRKAR2B//STK33//DGKE//AKAP6//SOCS5//NLN//DNAJC16//GLRX//NHLRC2//ESCO2//BAHD1//ARID4B//ZMYND11//ZNF782//ZNF800//ZFP30//KDM2A//PTF1A//NRBF2//ZNF680//MLLT6//COMMD2//VGLL1//SLC2A4RG//PAK6//ZNF630//ZBTB26//RFX3//SOX4//BTF3//BTG1//RHOXF2B//ZNF3//ZNF131//ZNF134//ZNF208//ZNF227//ZNF655//SNIP1//MED28//POLR2K//MED6//KLF17//EMX2//FOXJ3//FOXF2//EPC2//ATAD2//HOXA3//HOXB6//HOXD1//BOLA3//MYBL1//NEUROG1//NFE2//NHLH2//BRWD1//POU3F2//TRERF1//RFX4//DMRTA1//RFX7//SMARCD2//FOSL1//TSC22D4//LCOR//RHOXF2//KLF7//MTPN//DDX6//KBTBD8//EIF3B//FLRT1//NCK1//DBNDD2//ANP32B//EGLN3//CASP3//CHRM2//VSIG4//STMN2//CLASP2//MID1IP1//MTBP//RRAGD//CDC27//NABP1//CDC14A//CCR1//ACVR1//RAPGEF4//GPR183//FRZB//PTGFR//SOS2//OR13A1//GRIK2//DLL1//G3BP2//MDFIC//NMI//NEUROD1//ARHGAP1//RALGPS2//CDK19//TRPC3//SNAP25//ZFAND6//TEX11//BNIP3//UNG//TES//MYOG//ST18//CFDP1//PROCR//HS3ST5//OTX2//UBE4B//NPNT//PIGA//SH3BGR//SH3BGRL//SLC30A5//SLC1A1//C1QTNF9//IFNA1//MACC1//INHBB//OGN//IL36G//BMP3//DRAM1//PTPN22//MTMR9//TRIM8//RAB3GAP1//LDLR//QKI//LDLRAD4//C5ORF30//VWC2//CHMP3//DLG2//PLEKHF2//A2ML1//WFDC13//PMEPA1//NYAP1//PRR16//RAB12//SNX5//CPEB2//TNFAIP8L3//ATXN1//BTAF1//SYT6//ACSL1//IL1RAP//PLP2//IL20RA//SH3D19//DENND1B//HECW2//SPOPL//WRNIP1//SHISA2//ENPP4//H3F3C//EI24//FAM110C//PTP4A1//RAP2C//EGLN1//MMRN2//CLIP1//EVI5//USP6NL//MTM1//AGFG1//CCT6A//STIM2//RNF180//RNF125//LONRF3//LONRF1//MIEF2//KIF13A//RAB30//RAB9B//RAB6B//RAB28//PHACTR2//CD84//STX6//SYCP1//MEMO1//FRMD6//UBA5//ARFIP1//KCNA4//KCNJ3//KCNJ10//CACNA2D3//GSTA2//CA1//SYBU//ZMAT3//ITM2A//PXK//PAQR5//FASTK//YTHDF2//UBXN2B//PEX5L//ADAM12//GPD1//NCOA7//TFEC//MEOX2//ARMCX3//TET2//PCGF5//NFATC2IP//RAET1E//HPRT1//GCSAML//DCUN1D4//TMOD1//OTUD3//NRXN1//CRISP1//XAF1//BTBD7//SLC30A7//CARF//FIBIN//DSC2//DDHD2//SIVA1//PATE1//HSPB8//DYNLL2//CALCOCO2//TMED10//SERINC3//KCNS2//SESTD1//IER3IP1//CPEB1// |
| GO:2000026 | regulation of multicellular organismal development | Biological process | 92 | 2008 | 574 | 17653 | 1.4090640920638 | 0.000405709343972501 | 0.0289883824934427 | 3.391784989858 | 0.160278745644599 | FZD3//TGFBR2//BMPR2//CLASP2//STRAP//LDLRAD4//GATA2//SNAP25//STMN2//ADCYAP1//NPTN//NCK1//RAPGEF2//PTPRG//SPOCK1//TSC1//DLL1//MTPN//SPRED1//SULF1//PPARG//RFX4//GJA1//ACVR1//CCR1//DPYSL2//IFNG//WNT7A//MAF//SOD1//OTX2//EDN1//IL2//CBFB//IRF1//BTG1//REG3G//MYB//SOCS5//CDK6//IL5//NFE2//NTF3//DDX6//VWC2//NEUROD1//NEUROG1//CEBPB//POU4F1//TNF//EGLN1//C5AR1//VEGFB//ADAM12//UNG//MTM1//PRKAA1//ULK2//EFNA5//MYOG//FMR1//HECW2//NOS1//POU3F2//SLIT2//FLRT1//IL1RAP//DLG5//NRXN1//JARID2//ZFPM2//FZD6//AR//ESR1//BTBD7//CPEB3//LRP8//FRZB//IGF1//AKAP6//PTF1A//LDLR//AGTR2//MEOX2//MMRN2//YTHDF2//NLN//PLAA//LRP6//RFX3//GPR183//PHF14// |
| GO:0048518 | positive regulation of biological process | Biological process | 228 | 5841 | 574 | 17653 | 1.20047817691472 | 0.000410805863028728 | 0.0289883824934427 | 3.38636336677181 | 0.397212543554007 | GADD45A//TNF//CRKL//MAP3K1//MAP3K7CL//LAMTOR3//IGF1//NTF3//PIK3CB//PRKAA1//SOD1//C5AR1//EREG//EPS15//EFNA5//FMR1//NPTN//IFNG//BMPR2//VEGFB//CDK6//CCL28//ARL6IP1//FZD3//TGFBR2//SKP1//RPS6KA5//MYD88//CCL8//REG3G//PGC//AGTR2//EDN1//GJA1//ANP32B//EGLN3//PPARG//CASP3//VSIG4//CNOT6L//CNOT7//MDM4//CNOT6//SOX4//MTBP//MDFIC//ZNF268//ADCYAP1//DLL1//HOXA3//IL2//AR//POU3F2//SPHK2//PTGFR//BTC//FOSL1//NPNT//PIGA//ARHGAP1//SH3BGR//SH3BGRL//SLC1A1//BNIP3//TRIM8//TRPC3//MTPN//SULF1//WNT7A//PAN3//ARX//RAB3GAP1//PTPN22//LDLR//POU4F1//RHOXF2B//RHOXF2//QKI//CLASP2//CALM2//VWC2//SMOC2//MYOG//INHBB//BMP3//ACVR1//MTMR9//STMN2//NCK1//RAPGEF2//CBL//CPEB3//PRR16//RAB12//TSC1//ESR1//FOXF2//ATAD2//IL5//IRF1//LRP6//MYB//MYBL1//NEUROD1//NFE2//NOS1//NPAT//OTX2//PPP3CB//TRERF1//RFX3//TBL1XR1//RUNX3//DENND1B//ENPP4//H3F3C//AKAP6//CCR1//FAM110C//PTP4A1//GPR183//MET//CLIP1//YES1//NEUROG1//MIER1//RAP2C//RRAGD//PAK6//ATR//TNKS//CCT6A//STIM2//RNF180//RNF125//LONRF3//LONRF1//SOCS5//MIEF2//POLR2K//PPP1R15B//IL1RAP//CEBPB//LRP8//IFNA1//PRKAR2B//IQGAP2//ARFIP1//DLG5//SYBU//PTGES3//CDK19//FRZB//ARHGEF4//BCL2A1//ZMAT3//SOS2//SLIT2//CD84//GATA2//SPRED1//GRIK2//TNFAIP8L3//BTG1//MID1IP1//HECW2//CCNY//DOK1//ADAM12//GPD1//UNG//MED6//ZBTB18//NCOA7//ELK3//EN1//TFEC//FOXJ3//ZFPM2//PTF1A//MACC1//IRF2//MAF//MEOX2//NFIA//NHLH2//NR4A2//VGLL1//ARMCX3//ARID4B//EGLN1//TET2//RFX4//PCGF5//NFATC2IP//KLF7//CBFB//ST18//RAET1E//HPRT1//DUSP22//RASGEF1A//MTM1//GCSAML//CCL2//SH3D19//ARID5B//DCUN1D4//ARRDC3//JARID2//FLRT1//NRXN1//SNAP25//YTHDF2//CARF//AIMP1//ACSL1//DDHD2//CD2AP//DYNLL2//CALCOCO2//CHMP3//SERINC3//NMI//PLAA//SNIP1//GLRX//IL20RA// |
| GO:0034645 | cellular macromolecule biosynthetic process | Biological process | 213 | 5401 | 574 | 17653 | 1.21286385860923 | 0.00041148672473318 | 0.0289883824934427 | 3.38564417130985 | 0.371080139372822 | RAD9B//IGF1//NFIA//ATR//WRNIP1//RBBP8//RRM2//DTD1//NPAT//TSHZ1//ZBTB18//CEBPB//ZNF268//TCERG1//STRAP//KLF12//ARX//EDN1//CC2D1B//EN1//ESR1//CPEB3//MYT1L//RCOR1//ZFPM2//GATA2//CNOT7//HOXC8//HSBP1//IFNG//IRF2//AR//JARID2//ZNF568//MAF//MDM4//MYB//NR4A2//KLF3//POU4F1//PPARG//DUSP22//ZBTB4//TNF//ZNF217//LRP8//BHLHE41//TBL1XR1//ARID5B//BHLHE40//RUNX3//PHF14//SYCP1//SECISBP2L//ELK3//TFEC//FOXJ3//FOXF2//PTF1A//HIVEP2//MACC1//IRF1//MEOX2//MYBL1//NEUROD1//NHLH2//OTX2//POLR2K//POU3F2//RFX4//BMPR2//SOX4//BTF3//CARF//FOSL1//KLF7//CBFB//NMI//SRSF2//GMNC//ESCO2//BAHD1//ZMYND11//KLF17//NCOA7//ZNF782//ZNF800//ZFP30//KDM2A//CNOT6L//EPC2//HBP1//ATAD2//MDFIC//HOXA3//HOXB6//HOXD1//ZNF680//NEUROG1//NFE2//COMMD2//VGLL1//ARID4B//BRWD1//PRKAA1//TRERF1//SLC2A4RG//ZNF630//CNOT6//ZBTB26//MIER1//RFX3//ATXN1//DMRTA1//SMARCD2//ZNF3//ZNF131//ZNF134//ZNF208//ZNF227//ZNF655//MED28//TSC22D4//PCGF5//LCOR//ST18//NRBF2//MLLT6//PAK6//BTG1//RHOXF2B//SNIP1//CBL//RPS6KA5//MED6//EMX2//BOLA3//NR3C2//RFX7//RHOXF2//BLZF1//CPEB2//MRPL35//CPEB1//METTL17//EIF3B//MTPN//DDX6//TSC1//KBTBD8//AIMP1//FUT9//FUT2//ST8SIA5//MAN1A1//ST8SIA3//CCDC126//TET2//VEGFB//PIGP//PIGA//PTGES3//G3BP2//EREG//NDST3//HS3ST5//FMR1//PRR16//PIGS//AGTR2//IL5//LRP6//NOS1//PPP3CB//WNT7A//ACVR1//RASD1//BTAF1//SLC35D1//CHST7//DSEL//BMP3//SULF1//VWC2//TNKS//DCP2//CCT6A//NCK1//MYOG//C5AR1//INTS7//NABP1//ITM2A//EGLN1//FZD6//ADCYAP1//DLL1//IL2//MET//ARMCX3//YES1//NFATC2IP//TRIM8//IL1RAP//MYD88//YTHDF2//QKI//EGLN3//ATG16L1//RAB3GAP1//PPP1R15B//GJA1//NPNT// |
| GO:0006915 | apoptotic process | Biological process | 90 | 1958 | 574 | 17653 | 1.4136322707164 | 0.00042557923463158 | 0.0296720862248802 | 3.37101957080918 | 0.156794425087108 | BCL2A1//IL2//CASP3//ANP32B//EGLN3//PPARG//TNF//ZNF268//ARL6IP1//IGF1//MDM4//MET//MYD88//ZFAND6//PRKAA1//TEX11//SPHK2//PTGFR//BNIP3//BTC//UNG//WNT7A//CBL//UBE4B//ADCYAP1//ESR1//INHBB//TNFAIP8L3//MAP3K1//NTF3//PAK6//RBM25//BMP3//DLG5//PIK3CB//DLL1//CCL2//EDN1//LRP6//IFNG//USP28//GADD45A//CDK19//FRZB//NEUROD1//ARHGEF4//POU4F1//ZMAT3//SOD1//SOS2//SOX4//FOSL1//SLIT2//CEBPB//EN1//GRIK2//NR4A2//C5AR1//MYB//TSC22D4//BMPR2//FASTK//SIVA1//APPL1//FZD3//DYNLL2//SERINC3//NCK1//TGFBR2//IER3IP1//CFDP1//BTG1//GATA2//RAPGEF2//ZMYND11//AR//ACVR1//AGTR2//IL20RA//ST18//RTN3//SULF1//GJA1//IRF1//CHMP3//XAF1//DRAM1//DUSP22//AIMP1//EI24// |
| GO:0046545 | development of primary female sexual characteristics | Biological process | 11 | 101 | 574 | 17653 | 3.34948425156104 | 0.000439648983291417 | 0.030340266061223 | 3.35689392683077 | 0.0191637630662021 | CEBPB//ADCYAP1//SPO11//INHBB//DMRTA1//SOD1//ESR1//EREG//ARID5B//SLIT2//ZFPM2// |
| GO:0031401 | positive regulation of protein modification process | Biological process | 60 | 1188 | 574 | 17653 | 1.55325027276247 | 0.000447276460431828 | 0.030554855574752 | 3.34942395756974 | 0.104529616724739 | GADD45A//TNF//CRKL//MAP3K1//MAP3K7CL//LAMTOR3//IGF1//NTF3//PIK3CB//PRKAA1//SOD1//C5AR1//EFNA5//FMR1//NPTN//IFNG//EREG//MDFIC//CALM2//INHBB//BMP3//BMPR2//ACVR1//RAP2C//PAK6//TGFBR2//AGTR2//PPP1R15B//AKAP6//RPS6KA5//IFNA1//PRKAR2B//NOS1//IL2//EDN1//AR//MET//CCNY//ADCYAP1//RAPGEF2//DUSP22//WNT7A//IL5//YES1//LRP8//DCUN1D4//ARRDC3//MYB//JARID2//CCR1//GPR183//NPNT//PTPN22//TNFAIP8L3//CCL2//CCL8//ACSL1//NMI//RAB3GAP1//SOX4// |
| GO:0051174 | regulation of phosphorus metabolic process | Biological process | 84 | 1806 | 574 | 17653 | 1.43043513491613 | 0.00047013823287771 | 0.0317954486895195 | 3.32777442940599 | 0.146341463414634 | GADD45A//DIRAS3//TNF//CRKL//MAP3K1//MAP3K7CL//LAMTOR3//IGF1//NTF3//PIK3CB//PRKAA1//SOD1//C5AR1//SPRED1//DUSP22//IL2//LRP6//PPP1R15B//SLIT2//EFNA5//FMR1//NPTN//IFNG//EREG//CBL//SOCS5//FLRT1//NCK1//DBNDD2//MDFIC//NPNT//CALM2//INHBB//BMP3//BMPR2//ACVR1//LDLR//MTMR9//DLG2//PLEKHF2//TBL1XR1//EGLN1//NOS1//CDK6//RAP2C//PAK6//TGFBR2//PHACTR2//AGTR2//AKAP6//RPS6KA5//IFNA1//MET//PRKAR2B//PTGES3//AR//CNOT7//EDN1//PTPN22//NRBF2//TNFAIP8L3//UBXN2B//TSC1//CASP3//CCNY//MYOG//GPD1//ADCYAP1//RAPGEF2//GRIK2//ZMYND11//WNT7A//IL5//YES1//LRP8//SAMSN1//STRAP//PMEPA1//LDLRAD4//CCR1//GPR183//CCL2//CCL8//ACSL1// |
| GO:0009966 | regulation of signal transduction | Biological process | 136 | 3215 | 574 | 17653 | 1.30096184587707 | 0.000477765502310688 | 0.031991367248784 | 3.32078521226089 | 0.236933797909408 | GADD45A//TNF//CRKL//MAP3K1//MAP3K7CL//LAMTOR3//IGF1//NTF3//PIK3CB//PRKAA1//SOD1//C5AR1//SPRED1//DUSP22//CBFB//EREG//CBL//SOCS5//NCK1//MDFIC//ADCYAP1//OTX2//ARHGAP1//SH3BGR//SH3BGRL//MET//ACVR1//EDN1//C1QTNF9//IFNA1//IFNG//MACC1//IL2//IL5//INHBB//OGN//IL36G//CCL28//CCL2//CCL8//BMP3//BTC//VEGFB//WNT7A//CALCB//AIMP1//AKAP11//AKAP4//AKAP6//BMPR2//PMEPA1//LDLRAD4//RFX4//ESR1//SULF1//SOX4//APCDD1//FRZB//SHISA2//NPNT//STRAP//TGFBR2//VWC2//MMRN2//MTM1//TSC1//RRAGD//ARAP2//APPL1//AGFG1//PSD//RALGPS2//UBA5//AR//IRF1//PTPN22//ARHGEF4//SOS2//DLG5//RNF125//EPS15//CNOT7//ZFAND6//GJA1//MYD88//MIER1//TRIM8//ZMYND11//PAK6//ATR//PEX5L//RAPGEF2//DOK1//NPTN//GATA2//YTHDF2//DLL1//GRIK2//FLRT1//NEUROD1//RASGEF1A//GCSAML//ARHGAP12//CD2AP//SLIT2//AGTR2//TNFAIP8L3//FAM110C//OTUD3//NOS1//CALM2//PPARG//CCNY//LRP6//CCR1//GPR183//ARRDC3//FZD6//BNIP3//TBL1XR1//TNKS//PATE1//DYNLL2//RAD9B//MDM4//POU4F1//RBBP8//SERINC3//PPP1R15B//NRXN1//PHF14//NR4A2//BCL2A1//IL20RA//ST18//SAMSN1// |
| GO:0001932 | regulation of protein phosphorylation | Biological process | 69 | 1421 | 574 | 17653 | 1.4933501214976 | 0.00050030677891999 | 0.0331723014297636 | 3.30076361259211 | 0.120209059233449 | GADD45A//DIRAS3//TNF//CRKL//MAP3K1//MAP3K7CL//LAMTOR3//IGF1//NTF3//PIK3CB//PRKAA1//SOD1//C5AR1//SPRED1//DUSP22//IL2//LRP6//PPP1R15B//SLIT2//EFNA5//FMR1//NPTN//IFNG//EREG//CBL//SOCS5//FLRT1//NCK1//DBNDD2//MDFIC//CALM2//INHBB//BMP3//BMPR2//ACVR1//CDK6//RAP2C//PAK6//TGFBR2//RPS6KA5//IFNA1//PRKAR2B//CNOT7//EDN1//AR//MET//PTPN22//CASP3//CCNY//TSC1//ADCYAP1//RAPGEF2//GRIK2//ZMYND11//WNT7A//IL5//YES1//LRP8//SAMSN1//STRAP//PMEPA1//LDLRAD4//CCR1//GPR183//NPNT//TNFAIP8L3//CCL2//CCL8//ACSL1// |
| GO:0043403 | skeletal muscle tissue regeneration | Biological process | 6 | 32 | 574 | 17653 | 5.76644163763066 | 0.000507154435665901 | 0.0332998587224125 | 3.29485977174258 | 0.0104529616724739 | IGF1//WNT7A//TGFBR2//MTPN//GJA1//MYOG// |
| GO:0044249 | cellular biosynthetic process | Biological process | 252 | 6579 | 574 | 17653 | 1.17800540522505 | 0.000539991007388385 | 0.0348550514866894 | 3.26761347253652 | 0.439024390243902 | RAD9B//IGF1//NFIA//ATR//WRNIP1//RBBP8//RRM2//DTD1//NPAT//TSHZ1//ZBTB18//CEBPB//ZNF268//TCERG1//STRAP//KLF12//ARX//EDN1//CC2D1B//EN1//ESR1//CPEB3//MYT1L//RCOR1//ZFPM2//GATA2//CNOT7//HOXC8//HSBP1//IFNG//IRF2//AR//JARID2//ZNF568//MAF//MDM4//MYB//NR4A2//KLF3//POU4F1//PPARG//DUSP22//ZBTB4//TNF//ZNF217//LRP8//BHLHE41//TBL1XR1//ARID5B//BHLHE40//RUNX3//PHF14//SYCP1//NOS1//SECISBP2L//PTGES3//MGST1//ST8SIA3//ELK3//TFEC//FOXJ3//FOXF2//PTF1A//HIVEP2//MACC1//IRF1//MEOX2//MYBL1//NEUROD1//NHLH2//OTX2//POLR2K//POU3F2//RFX4//BMPR2//SOX4//BTF3//CARF//FOSL1//KLF7//CBFB//NMI//SRSF2//SLC35D1//HPRT1//IMPDH1//CMPK1//DCTPP1//GMNC//ESCO2//BAHD1//ZMYND11//KLF17//NCOA7//ZNF782//ZNF800//ZFP30//KDM2A//CNOT6L//EPC2//HBP1//ATAD2//MDFIC//HOXA3//HOXB6//HOXD1//ZNF680//NEUROG1//NFE2//COMMD2//VGLL1//ARID4B//BRWD1//PRKAA1//TRERF1//SLC2A4RG//ZNF630//CNOT6//ZBTB26//MIER1//RFX3//ATXN1//DMRTA1//SMARCD2//ZNF3//ZNF131//ZNF134//ZNF208//ZNF227//ZNF655//MED28//TSC22D4//PCGF5//LCOR//ST18//NRBF2//MLLT6//PAK6//BTG1//RHOXF2B//SNIP1//CBL//RPS6KA5//MED6//EMX2//BOLA3//NR3C2//RFX7//RHOXF2//BLZF1//CPEB2//MRPL35//CPEB1//METTL17//EIF3B//MTPN//DDX6//TSC1//KBTBD8//AIMP1//FUT9//FUT2//ST8SIA5//MAN1A1//CCDC126//TET2//VEGFB//PIGP//PIGA//GLUD2//LGSN//SERINC3//DDHD2//LCLAT1//GPD1//PLA2G12A//CDS1//ABHD3//SLC44A1//MTM1//PIK3CB//MTMR9//SPHK2//GGT6//GPHN//G3BP2//DGKE//MTRR//AK4//GLT6D1//EREG//LDLR//NDST3//HS3ST5//FMR1//PRR16//PIGS//AGTR2//IL5//LRP6//PPP3CB//WNT7A//ACVR1//RASD1//BTAF1//OGN//CHST1//ELOVL7//CHST7//DSEL//BMP3//SULF1//VWC2//CALM2//EGLN1//TNKS//DCP2//CCT6A//ACSL1//NCK1//QKI//MYOG//C5AR1//INTS7//NABP1//ITM2A//FZD6//UNG//MID1IP1//ADCYAP1//DLL1//IL2//MET//ARMCX3//YES1//NFATC2IP//RAPGEF2//INHBB//SOD1//TRIM8//IL1RAP//MYD88//YTHDF2//EGLN3//ATG16L1//GSTA2//GSTM3//RAB3GAP1//RAP2C//PPP1R15B//GJA1//NPNT// |
| GO:0010468 | regulation of gene expression | Biological process | 194 | 4873 | 574 | 17653 | 1.22436793509854 | 0.000541147479831789 | 0.0348550514866894 | 3.26668435975746 | 0.337979094076655 | NPAT//RRM2//TSHZ1//ZBTB18//CEBPB//ZNF268//TCERG1//STRAP//KLF12//ARX//EDN1//CC2D1B//EN1//ESR1//CPEB3//MYT1L//RCOR1//ZFPM2//GATA2//CNOT7//HOXC8//HSBP1//IFNG//IRF2//AR//JARID2//ZNF568//MAF//MDM4//MYB//NFIA//NR4A2//KLF3//POU4F1//PPARG//DUSP22//ZBTB4//RBBP8//TNF//ZNF217//LRP8//BHLHE41//TBL1XR1//ARID5B//BHLHE40//RUNX3//PHF14//DCP2//CNOT6L//PAN3//CNOT6//FMR1//MBNL1//SMU1//RBM25//SRSF2//SECISBP2L//BAHD1//ARID4B//DIRAS3//ZMYND11//ZNF782//ZNF800//ZFP30//KDM2A//PTF1A//NRBF2//HIVEP2//ZNF680//MLLT6//COMMD2//VGLL1//SLC2A4RG//PAK6//ZNF630//ZBTB26//RFX3//SOX4//BTF3//BTG1//RHOXF2B//ZNF3//ZNF131//ZNF134//ZNF208//ZNF227//ZNF655//SNIP1//MED28//CBL//RPS6KA5//POLR2K//MED6//KLF17//ELK3//EMX2//FOXJ3//FOXF2//EPC2//HBP1//ATAD2//HOXA3//HOXB6//HOXD1//BOLA3//NR3C2//MYBL1//NEUROG1//NFE2//NHLH2//BRWD1//POU3F2//TRERF1//MIER1//RFX4//DMRTA1//RFX7//SMARCD2//FOSL1//TSC22D4//LCOR//RHOXF2//BLZF1//KLF7//CBFB//MTPN//DDX6//TSC1//KBTBD8//EIF3B//G3BP2//RAB3GAP1//PTPN22//GJA1//IGF1//LDLR//MYD88//PIK3CB//PRKAA1//PTGFR//QKI//CCR1//PRR16//AGTR2//MDFIC//IL5//IRF1//LRP6//NEUROD1//NOS1//OTX2//PPP3CB//WNT7A//ACVR1//EREG//RASD1//ATXN1//BTAF1//C5AR1//BMP3//BMPR2//SULF1//VWC2//NCK1//EGLN1//FZD6//FASTK//YTHDF2//ADCYAP1//NCOA7//TFEC//DLL1//MACC1//IL2//MEOX2//MET//MYOG//ARMCX3//TET2//YES1//PCGF5//NFATC2IP//TNKS//ST18//TRIM8//IL1RAP//CARF//EGLN3//CPEB2//PPP1R15B//NPNT//CPEB1//CDK6//TGFBR2// |
| GO:0048522 | positive regulation of cellular process | Biological process | 203 | 5142 | 574 | 17653 | 1.21414510819554 | 0.000588670494066381 | 0.0368884543825182 | 3.23012773178643 | 0.353658536585366 | GADD45A//TNF//CRKL//MAP3K1//MAP3K7CL//LAMTOR3//IGF1//NTF3//PIK3CB//PRKAA1//SOD1//C5AR1//EPS15//EFNA5//FMR1//NPTN//IFNG//BMPR2//VEGFB//FZD3//TGFBR2//CCL8//EREG//ANP32B//EGLN3//PPARG//CASP3//CNOT6L//CNOT7//MDM4//CNOT6//SOX4//MTBP//MDFIC//ZNF268//ADCYAP1//REG3G//AGTR2//EDN1//DLL1//HOXA3//IL2//AR//POU3F2//SPHK2//PTGFR//BTC//FOSL1//ARHGAP1//SH3BGR//SH3BGRL//BNIP3//TRIM8//TRPC3//WNT7A//PAN3//CLASP2//GJA1//NPNT//CALM2//MYOG//INHBB//BMP3//ACVR1//LDLR//MTMR9//STMN2//NCK1//RAPGEF2//CBL//CPEB3//PRR16//RAB12//TSC1//ESR1//FOXF2//ATAD2//IL5//IRF1//LRP6//MYB//MYBL1//NEUROD1//NFE2//NOS1//NPAT//OTX2//PPP3CB//TRERF1//RFX3//TBL1XR1//RUNX3//SULF1//MTPN//H3F3C//AKAP6//CCR1//FAM110C//PTP4A1//GPR183//MET//CLIP1//YES1//NEUROG1//MIER1//RAP2C//RRAGD//PAK6//ATR//TNKS//CCT6A//RNF180//RNF125//LONRF3//LONRF1//SOCS5//MIEF2//PPP1R15B//RPS6KA5//IFNA1//PTPN22//PRKAR2B//IQGAP2//ARFIP1//DLG5//SYBU//PTGES3//CDK19//FRZB//ARHGEF4//POU4F1//BCL2A1//ZMAT3//SOS2//SLIT2//MYD88//GATA2//SPRED1//GRIK2//TNFAIP8L3//CEBPB//BTG1//VWC2//MID1IP1//CCNY//DOK1//GPD1//UNG//MED6//ZBTB18//NCOA7//ELK3//EN1//TFEC//FOXJ3//ZFPM2//PTF1A//MACC1//IRF2//MAF//MEOX2//NFIA//NHLH2//NR4A2//VGLL1//ARMCX3//ARID4B//EGLN1//TET2//RFX4//PCGF5//NFATC2IP//KLF7//CBFB//ST18//HPRT1//DUSP22//RASGEF1A//CDK6//LRP8//CCL2//SH3D19//DCUN1D4//ARRDC3//JARID2//FLRT1//IL1RAP//NRXN1//SNAP25//YTHDF2//QKI//CARF//RAB3GAP1//AIMP1//ACSL1//DDHD2//DYNLL2//CALCOCO2//SERINC3//NMI//PLAA//SNIP1//IL20RA// |
| GO:0010562 | positive regulation of phosphorus metabolic process | Biological process | 57 | 1126 | 574 | 17653 | 1.55683681868537 | 0.00058908074424249 | 0.0368884543825182 | 3.22982517316608 | 0.0993031358885017 | GADD45A//TNF//CRKL//MAP3K1//MAP3K7CL//LAMTOR3//IGF1//NTF3//PIK3CB//PRKAA1//SOD1//C5AR1//EFNA5//FMR1//NPTN//IFNG//EREG//MDFIC//NPNT//CALM2//INHBB//BMP3//BMPR2//ACVR1//MTMR9//NOS1//RAP2C//PAK6//TGFBR2//AGTR2//PPP1R15B//AKAP6//RPS6KA5//IFNA1//MET//PRKAR2B//PTGES3//AR//IL2//EDN1//TNFAIP8L3//CCNY//GPD1//ADCYAP1//RAPGEF2//DUSP22//WNT7A//IL5//YES1//LRP8//CCR1//GPR183//PTPN22//CCL2//CCL8//ACSL1//MYOG// |
| GO:0045937 | positive regulation of phosphate metabolic process | Biological process | 57 | 1126 | 574 | 17653 | 1.55683681868537 | 0.00058908074424249 | 0.0368884543825182 | 3.22982517316608 | 0.0993031358885017 | GADD45A//TNF//CRKL//MAP3K1//MAP3K7CL//LAMTOR3//IGF1//NTF3//PIK3CB//PRKAA1//SOD1//C5AR1//EFNA5//FMR1//NPTN//IFNG//EREG//MDFIC//NPNT//CALM2//INHBB//BMP3//BMPR2//ACVR1//MTMR9//NOS1//RAP2C//PAK6//TGFBR2//AGTR2//PPP1R15B//AKAP6//RPS6KA5//IFNA1//MET//PRKAR2B//PTGES3//AR//IL2//EDN1//TNFAIP8L3//CCNY//GPD1//ADCYAP1//RAPGEF2//DUSP22//WNT7A//IL5//YES1//LRP8//CCR1//GPR183//PTPN22//CCL2//CCL8//ACSL1//MYOG// |
| GO:0023056 | positive regulation of signaling | Biological process | 81 | 1743 | 574 | 17653 | 1.42920412361242 | 0.000615197831879267 | 0.0381704856605457 | 3.21098520377316 | 0.141114982578397 | GADD45A//TNF//CRKL//MAP3K1//MAP3K7CL//LAMTOR3//IGF1//NTF3//PIK3CB//PRKAA1//SOD1//C5AR1//EPS15//EREG//MDFIC//ARHGAP1//SH3BGR//SH3BGRL//INHBB//BMP3//BMPR2//ACVR1//CBL//EDN1//SULF1//SOX4//NPNT//RRAGD//GJA1//AR//PTPN22//DLG5//PPP3CB//SYBU//IFNG//IL2//MYD88//MIER1//TRIM8//MET//SPRED1//ATR//ADCYAP1//RAPGEF2//DOK1//NPTN//DLL1//DUSP22//WNT7A//IL5//RASGEF1A//GRIK2//SOS2//ESR1//TNFAIP8L3//FAM110C//BTC//NRXN1//SNAP25//CALM2//AKAP6//RAB3GAP1//AIMP1//CCR1//GPR183//CCL2//CCL8//ARRDC3//BNIP3//LRP6//TBL1XR1//TNKS//NOS1//FMR1//DYNLL2//SERINC3//NCK1//MYB//AGTR2//IL20RA//ST18// |
| GO:0008406 | gonad development | Biological process | 17 | 215 | 574 | 17653 | 2.43173972935743 | 0.000686329045578244 | 0.0421967575931424 | 3.16346762129375 | 0.029616724738676 | CEBPB//ADCYAP1//SPO11//INHBB//DMRTA1//SOD1//ESR1//EREG//ZFPM2//RNF38//TEX11//ARID5B//CBL//AR//MGST1//SLIT2//ARID4B// |
| GO:0065007 | biological regulation | Biological process | 436 | 12351 | 574 | 17653 | 1.08565289893157 | 0.000696815410677743 | 0.042455519120843 | 3.15688225309469 | 0.759581881533101 | RAD9B//INTS7//ATR//USP28//GADD45A//DIRAS3//NPAT//RRM2//TSHZ1//ZBTB18//CEBPB//ZNF268//TCERG1//STRAP//KLF12//ARX//EDN1//CC2D1B//EN1//ESR1//CPEB3//MYT1L//RCOR1//ZFPM2//GATA2//CNOT7//HOXC8//HSBP1//IFNG//IRF2//AR//JARID2//ZNF568//MAF//MDM4//MYB//NFIA//NR4A2//KLF3//POU4F1//PPARG//DUSP22//ZBTB4//RBBP8//TNF//ZNF217//LRP8//BHLHE41//TBL1XR1//ARID5B//BHLHE40//RUNX3//PHF14//CSF2RA//SPRED1//EREG//RASGEF1A//IL2//IL5//MAP3K1//MET//MAP3K7CL//CCL2//BTC//LAMTOR3//RAPGEF2//DCP2//CRKL//IGF1//NTF3//PIK3CB//PRKAA1//SOD1//C5AR1//CNOT6L//PAN3//CNOT6//FMR1//MBNL1//SMU1//RBM25//SRSF2//PTGES3//SNAP25//NOS1//AKAP6//SECISBP2L//SPOCK1//BLZF1//ARRDC3//CASP3//RPS6KA5//BCL2A1//CAMLG//PPP3CB//EPS15//LRP6//PPP1R15B//SLIT2//EFNA5//NPTN//SULF1//GJA1//AIMP1//BMPR2//VEGFB//TSC1//CDK6//CCL28//CBFB//AGTR2//CALM2//ARL6IP1//FZD3//TGFBR2//SKP1//MYD88//CCL8//REG3G//PGC//IRF1//SAMSN1//ADCYAP1//SLC2A5//PRKAR2B//SPHK2//PROCR//ENPP4//MMRN1//IFNA1//NFE2//APCDD1//CCNY//HBP1//WNT7A//TNKS//FZD6//CDS1//IQGAP2//ARAP2//CLCN6//DOK1//DPYSL2//ELK3//HTR3D//EVI2A//CD2AP//APPL1//OSTF1//GLRB//LRP12//HIVEP2//NR3C2//HPCAL4//RASD1//KCNK10//PSD//MIER1//SMOC1//SMOC2//CALCB//RERGL//OR6A2//CBL//AKAP4//DLG5//PLAA//ARHGAP12//CD69//ULK2//PTPRG//YES1//CD8A//AKAP11//DGKH//DCX//ARHGEF4//TREM1//STK33//DGKE//SOCS5//NLN//DNAJC16//GLRX//NHLRC2//ESCO2//BAHD1//ARID4B//ZMYND11//ZNF782//ZNF800//ZFP30//KDM2A//PTF1A//NRBF2//ZNF680//MLLT6//COMMD2//VGLL1//SLC2A4RG//PAK6//ZNF630//ZBTB26//RFX3//SOX4//BTF3//BTG1//RHOXF2B//ZNF3//ZNF131//ZNF134//ZNF208//ZNF227//ZNF655//SNIP1//MED28//POLR2K//MED6//KLF17//EMX2//FOXJ3//FOXF2//EPC2//ATAD2//HOXA3//HOXB6//HOXD1//BOLA3//MYBL1//NEUROG1//NHLH2//BRWD1//POU3F2//TRERF1//RFX4//DMRTA1//RFX7//SMARCD2//FOSL1//TSC22D4//LCOR//RHOXF2//KLF7//MTPN//DDX6//KBTBD8//EIF3B//FLRT1//NCK1//DBNDD2//HSD11B1//CCR1//GRIK2//STIM2//EGLN1//SLC30A5//ANP32B//EGLN3//CHRM2//VSIG4//STMN2//CLASP2//MID1IP1//MTBP//RRAGD//CDC27//NABP1//CDC14A//ACVR1//RAPGEF4//GPR183//FRZB//PTGFR//SOS2//OR13A1//DLL1//G3BP2//MDFIC//NMI//NEUROD1//ARHGAP1//RALGPS2//NRXN1//CDK19//TRPC3//ZFAND6//TEX11//BNIP3//UNG//TES//MYOG//ST18//CFDP1//HS3ST5//OTX2//UBE4B//NPNT//PIGA//SH3BGR//SH3BGRL//SLC1A1//C1QTNF9//MACC1//INHBB//OGN//IL36G//BMP3//DRAM1//PTPN22//MTMR9//TRIM8//RAB3GAP1//LDLR//QKI//LDLRAD4//C5ORF30//VWC2//CHMP3//DLG2//PLEKHF2//A2ML1//WFDC13//SLC41A1//PMEPA1//NYAP1//PRR16//RAB12//SNX5//CPEB2//TNFAIP8L3//ATXN1//BTAF1//OTUD3//STX12//CCT6A//SYT6//ACSL1//IL1RAP//PLP2//IL20RA//SH3D19//DENND1B//HECW2//SPOPL//WRNIP1//SHISA2//H3F3C//EI24//FAM110C//PTP4A1//RAP2C//MMRN2//CLIP1//EVI5//USP6NL//MTM1//AGFG1//SLC30A7//RNF180//RNF125//LONRF3//LONRF1//MIEF2//KIF13A//RAB30//RAB9B//RAB6B//RAB28//PHACTR2//CD84//PXK//DNAJB4//STX6//SYCP1//MEMO1//FRMD6//SBF2//RP2//UBA5//ARFIP1//KCNA4//KCNJ3//KCNJ10//CACNA2D3//GSTA2//CA1//SYBU//ZMAT3//SLC44A1//ADH4//RDH14//ITM2A//PAQR5//FASTK//YTHDF2//UBXN2B//PEX5L//ADAM12//GPD1//NCOA7//TFEC//MEOX2//ARMCX3//TET2//PCGF5//NFATC2IP//RAET1E//HPRT1//KLHL3//GCSAML//DCUN1D4//SLC4A4//TMOD1//STEAP2//CRISP1//XAF1//BTBD7//CARF//FIBIN//DSC2//DDHD2//SIVA1//FAM155A//GPHN//PATE1//DTD1//HSPB8//DYNLL2//CALCOCO2//TMED10//SERINC3//KCNS2//MTRR//SESTD1//IER3IP1//CPEB1// |
| GO:0042325 | regulation of phosphorylation | Biological process | 73 | 1544 | 574 | 17653 | 1.45405954938528 | 0.000726977334982903 | 0.0438977474686551 | 3.13847912895568 | 0.127177700348432 | GADD45A//DIRAS3//TNF//CRKL//MAP3K1//MAP3K7CL//LAMTOR3//IGF1//NTF3//PIK3CB//PRKAA1//SOD1//C5AR1//SPRED1//DUSP22//IL2//LRP6//PPP1R15B//SLIT2//EFNA5//FMR1//NPTN//IFNG//EREG//CBL//SOCS5//FLRT1//NCK1//DBNDD2//MDFIC//CALM2//INHBB//BMP3//BMPR2//ACVR1//CDK6//RAP2C//PAK6//TGFBR2//RPS6KA5//IFNA1//MET//PRKAR2B//PTGES3//AR//CNOT7//EDN1//PTPN22//NRBF2//TNFAIP8L3//CASP3//CCNY//MYOG//GPD1//TSC1//ADCYAP1//RAPGEF2//GRIK2//ZMYND11//WNT7A//IL5//YES1//LRP8//SAMSN1//STRAP//PMEPA1//LDLRAD4//CCR1//GPR183//NPNT//CCL2//CCL8//ACSL1// |
| GO:0007610 | behavior | Biological process | 34 | 583 | 574 | 17653 | 1.79356446590685 | 0.000769196846649831 | 0.0460360909194054 | 3.11396250483577 | 0.0592334494773519 | ADCYAP1//GRIK2//POU4F1//CASP3//CNTNAP2//PPP3CB//PRKAR2B//ATXN1//FOSL1//NRXN1//CEBPB//NTF3//KCNK10//CPEB3//LDLR//NHLH2//HPRT1//DDHD2//SNAP25//SOD1//GLRB//KCNJ10//STRBP//RNF180//EN1//NR4A2//TMOD1//TSC1//NPTN//NEUROG1//AGTR2//DMRTA1//ARRDC3//GJA1// |
| GO:0021536 | diencephalon development | Biological process | 9 | 76 | 574 | 17653 | 3.64196313955621 | 0.000781919999298603 | 0.046097928280686 | 3.10683767870547 | 0.0156794425087108 | ARX//CNTNAP2//LRP6//RAB3GAP1//POU3F2//ADCYAP1//GATA2//NR4A2//POU4F1// |
| GO:0000209 | protein polyubiquitination | Biological process | 21 | 298 | 574 | 17653 | 2.16725323293501 | 0.000783862450433076 | 0.046097928280686 | 3.10576013917631 | 0.0365853658536585 | UBE2G1//KLHL3//UBE2W//CDC27//PLAA//PTPN22//NMI//UBE4B//RNF145//ZNRF2//RNF180//RNF125//HECW2//SKP1//THOP1//LONRF3//CBFB//TNKS//CBL//UBE3B//LONRF1// |
| GO:0016202 | regulation of striated muscle tissue development | Biological process | 13 | 143 | 574 | 17653 | 2.79585049097244 | 0.000799772858468628 | 0.0466281365674425 | 3.09703333840869 | 0.0226480836236934 | GJA1//DLL1//MTM1//PRKAA1//MYOG//JARID2//TGFBR2//ZFPM2//EDN1//IGF1//AKAP6//NLN//MTPN// |
| GO:0010647 | positive regulation of cell communication | Biological process | 80 | 1737 | 574 | 17653 | 1.41643548189738 | 0.000867260334011724 | 0.0500688773765037 | 3.06185051653343 | 0.139372822299652 | GADD45A//TNF//CRKL//MAP3K1//MAP3K7CL//LAMTOR3//IGF1//NTF3//PIK3CB//PRKAA1//SOD1//C5AR1//EREG//MDFIC//ARHGAP1//SH3BGR//SH3BGRL//GJA1//INHBB//BMP3//BMPR2//ACVR1//CBL//EDN1//SULF1//SOX4//NPNT//RRAGD//AR//PTPN22//DLG5//PPP3CB//SYBU//IFNG//IL2//MYD88//MIER1//TRIM8//MET//SPRED1//ATR//ADCYAP1//RAPGEF2//DOK1//NPTN//DLL1//DUSP22//WNT7A//IL5//RASGEF1A//GRIK2//SOS2//ESR1//TNFAIP8L3//FAM110C//BTC//NRXN1//SNAP25//CALM2//AKAP6//RAB3GAP1//AIMP1//CCR1//GPR183//CCL2//CCL8//ARRDC3//BNIP3//LRP6//TBL1XR1//TNKS//NOS1//FMR1//DYNLL2//SERINC3//NCK1//MYB//AGTR2//IL20RA//ST18// |
| GO:0045786 | negative regulation of cell cycle | Biological process | 36 | 634 | 574 | 17653 | 1.74630409215313 | 0.000879670330468328 | 0.0500688773765037 | 3.0556800556718 | 0.0627177700348432 | RAD9B//INTS7//ATR//USP28//GADD45A//CNOT6L//CNOT7//MDM4//CNOT6//SOX4//CDK6//HBP1//MTBP//IFNG//IRF1//PRKAA1//RRAGD//TSC1//LAMTOR3//NABP1//SKP1//RBBP8//CASP3//BTG1//TNF//FZD3//RRM2//NEUROD1//ZNF268//MYOG//POU4F1//CDC14A//TNKS//ZNF655//ADCYAP1//RUNX3// |
| GO:1902533 | positive regulation of intracellular signal transduction | Biological process | 52 | 1021 | 574 | 17653 | 1.56633347780239 | 0.000895425515669486 | 0.0500688773765037 | 3.04797053430306 | 0.0905923344947735 | GADD45A//TNF//CRKL//MAP3K1//MAP3K7CL//LAMTOR3//IGF1//NTF3//PIK3CB//PRKAA1//SOD1//C5AR1//MDFIC//CBL//RRAGD//DLG5//IFNG//IL2//GJA1//MYD88//MIER1//TRIM8//EDN1//AR//MET//SPRED1//ATR//ADCYAP1//RAPGEF2//DUSP22//WNT7A//IL5//RASGEF1A//SOS2//EREG//ESR1//TNFAIP8L3//FAM110C//BTC//CALM2//AKAP6//CCR1//GPR183//NPNT//PTPN22//NPTN//CCL2//CCL8//NOS1//SERINC3//NCK1//IL20RA// |
| GO:0002064 | epithelial cell development | Biological process | 16 | 201 | 574 | 17653 | 2.44810789259279 | 0.00090858855669128 | 0.0500688773765037 | 3.04163273725189 | 0.0278745644599303 | MET//GJA1//CDK6//DLL1//AR//FRMD6//GSTM3//WNT7A//SOD1//RAPGEF2//TMOD1//RFX3//RAP2C//ARID4B//TNF//ESR1// |
| GO:0071495 | cellular response to endogenous stimulus | Biological process | 65 | 1350 | 574 | 17653 | 1.48076526003355 | 0.000910435611150759 | 0.0500688773765037 | 3.04075076344483 | 0.113240418118467 | DUSP22//TGFBR2//CBL//ACVR1//CHRM2//APPL1//SPRED1//FLRT1//POLR2K//PPARG//CEBPB//CPEB3//RRAGD//CPEB1//TNF//LAMTOR3//BMP3//BMPR2//NPNT//STRAP//PMEPA1//LDLRAD4//SULF1//VWC2//ESR1//AR//CPEB2//INHBB//SLIT2//UBA5//CBFB//ACSL1//YES1//AGTR2//NTF3//RAPGEF2//OTX2//SHISA2//NR3C2//NR4A2//PAQR5//CCL2//NPTN//NCK1//TSC1//IGF1//PTPN22//SOD1//GPD1//AKAP6//EFNA5//GJA1//EDN1//GJB2//PRKAR2B//PRKAA1//ADCYAP1//MYOG//POU4F1//WNT7A//PTGFR//CASP3//KLF3//STMN2//DYNC1LI2// |
| GO:0090304 | nucleic acid metabolic process | Biological process | 208 | 5329 | 574 | 17653 | 1.20039518171232 | 0.000910448292444987 | 0.0500688773765037 | 3.04074471427707 | 0.362369337979094 | RAD9B//IGF1//NFIA//ATR//WRNIP1//RBBP8//RRM2//DTD1//NPAT//TSHZ1//ZBTB18//CEBPB//ZNF268//TCERG1//STRAP//KLF12//ARX//EDN1//CC2D1B//EN1//ESR1//CPEB3//MYT1L//RCOR1//ZFPM2//GATA2//CNOT7//HOXC8//HSBP1//IFNG//IRF2//AR//JARID2//ZNF568//MAF//MDM4//MYB//NR4A2//KLF3//POU4F1//PPARG//DUSP22//ZBTB4//TNF//ZNF217//LRP8//BHLHE41//TBL1XR1//ARID5B//BHLHE40//RUNX3//PHF14//DCP2//CNOT6L//PAN3//CNOT6//FMR1//MBNL1//SMU1//RBM25//SRSF2//POLR2K//RBM41//SNRPN//NOB1//SPO11//SYCP1//TEX11//PTGES3//NABP1//ELK3//TFEC//FOXJ3//FOXF2//PTF1A//HIVEP2//MACC1//IRF1//MEOX2//MYBL1//NEUROD1//NHLH2//OTX2//POU3F2//RFX4//BMPR2//SOX4//BTF3//CARF//FOSL1//KLF7//CBFB//NMI//GMNC//ESCO2//GADD45A//EPC2//UBE2W//USP28//UNG//KDM2A//MTRR//CASP3//BAHD1//ZMYND11//KLF17//NCOA7//ZNF782//ZNF800//ZFP30//HBP1//ATAD2//MDFIC//HOXA3//HOXB6//HOXD1//ZNF680//NEUROG1//NFE2//COMMD2//VGLL1//ARID4B//BRWD1//PRKAA1//TRERF1//SLC2A4RG//ZNF630//ZBTB26//MIER1//RFX3//ATXN1//DMRTA1//SMARCD2//ZNF3//ZNF131//ZNF134//ZNF208//ZNF227//ZNF655//MED28//TSC22D4//PCGF5//LCOR//ST18//NRBF2//MLLT6//PAK6//BTG1//RHOXF2B//SNIP1//CBL//RPS6KA5//MED6//EMX2//BOLA3//NR3C2//RFX7//RHOXF2//BLZF1//ELAVL4//RTCA//SCAF8//CPEB1//QKI//AIMP1//G3BP2//EREG//DDX6//INTS7//AGTR2//IL5//LRP6//NOS1//PPP3CB//WNT7A//ACVR1//RASD1//BTAF1//BMP3//SULF1//VWC2//TNKS//CCT6A//DCTPP1//MYOG//C5AR1//TDRD5//EGLN1//FZD6//FASTK//YTHDF2//ADCYAP1//DLL1//IL2//MET//NCK1//ARMCX3//TET2//YES1//NFATC2IP//TRIM8//MTPN//IL1RAP//MYD88//EGLN3//RAP2C//GJA1//NPNT// |
| GO:1901861 | regulation of muscle tissue development | Biological process | 13 | 145 | 574 | 17653 | 2.75728703592455 | 0.000910612437869283 | 0.0500688773765037 | 3.0406664220477 | 0.0226480836236934 | MTPN//GJA1//DLL1//MTM1//PRKAA1//MYOG//JARID2//TGFBR2//ZFPM2//EDN1//IGF1//AKAP6//NLN// |
| GO:0045137 | development of primary sexual characteristics | Biological process | 17 | 221 | 574 | 17653 | 2.36571964620745 | 0.000933270686037266 | 0.0509008842715325 | 3.02999237511473 | 0.029616724738676 | CEBPB//ADCYAP1//SPO11//INHBB//DMRTA1//SOD1//ESR1//EREG//ZFPM2//RNF38//TEX11//ARID5B//CBL//AR//MGST1//SLIT2//ARID4B// |
| GO:0048856 | anatomical structure development | Biological process | 225 | 5836 | 574 | 17653 | 1.18569738950596 | 0.000978641174901531 | 0.0529484021268724 | 3.00937651588413 | 0.39198606271777 | WNT7A//HAPLN1//EN1//FRZB//IGF1//BMP3//SOX4//ELK3//EREG//HOXA3//MEOX2//CCL2//VEGFB//MMRN2//AIMP1//CEBPB//ADCYAP1//SPO11//INHBB//DMRTA1//SOD1//ESR1//YTHDF2//CRKL//SPHK2//TGFBR2//RAPGEF2//EDN1//ACVR1//ZFPM2//QKI//SMOC1//GATA2//NPNT//SLIT2//AGFG1//GJA1//AR//ZNF568//MBNL1//BTF3//FOSL1//LRP6//BMPR2//FZD3//DLL1//DCX//LRP12//NR4A2//SPOCK1//TMED10//SULF1//TSC1//ARID5B//RBBP8//FOXF2//DLG5//FZD6//MET//AK4//JARID2//PPARG//APCDD1//ANP32B//PPP3CB//ARHGAP12//RUNX3//ESCO2//GPR183//ITM2A//UBE4B//POU4F1//RFX3//CDK6//NEUROD1//FRMD6//TSHZ1//CFDP1//WDR47//WDFY3//LCLAT1//HIVEP2//SHISA2//NFE2//KLF3//ATR//PAQR5//STRBP//BTBD7//TRERF1//DUSP22//ZNF3//ZNF217//PTP4A1//EVI5//SPATA9//ADAM18//TDRD5//FOXJ3//DLG2//NEUROG1//BHLHE41//PTF1A//FUT9//CHRM2//DPYSL2//EFNA5//MYT1L//FMR1//GLRB//NTF3//POU3F2//ZIC5//OGN//ARX//DOK1//OTX2//PIK3CB//ENAH//KLF7//RPS6KA5//NRXN1//PCDHB10//NHLH2//HPCAL4//AGTR2//RAB3GAP1//CNTNAP2//PTPRG//ATXN1//CASP3//ZBTB18//MYOG//NOS1//ADAM12//GSTM3//BRWD1//RNF38//TEX11//CBL//RAI2//EMX2//HOXB6//HOXC8//CLASP2//BNIP3//STRAP//LDLRAD4//SNAP25//STMN2//NPTN//NCK1//KBTBD8//MTPN//FLRT1//UNG//SPRED1//RFX4//C5AR1//LRP8//HPRT1//CALM2//TACC2//KCNJ10//SH3D19//CBFB//TET2//HOXD1//IFNA1//PTPN22//IL2//TMOD1//TNF//HSD11B1//CCR1//GSTA2//IFNG//KRT27//DBNDD2//PSD//DSC2//KRTAP4-11//MAF//LRIG1//MGST1//SYCP1//SYCP3//HSBP1//SBF2//GPD2//IRF1//ARRDC3//CD8A//BTG1//REG3G//MYB//SOCS5//IL5//DDX6//VWC2//EGLN1//GJB2//PHF14//MTM1//PRKAA1//ULK2//SLC35D1//NYAP1//HECW2//MED28//IL1RAP//NFIA//SYBU//TBL1XR1//CPEB3//AKAP6//LDLR//KLHL3//SNX2//MIEF2//DDHD2//RAP2C//ARID4B//NLN//PLAA// |
| GO:0051094 | positive regulation of developmental process | Biological process | 64 | 1329 | 574 | 17653 | 1.48102238197487 | 0.000993429604353492 | 0.0533219397955767 | 3.00286290210626 | 0.111498257839721 | FZD3//TGFBR2//MYOG//STMN2//ADCYAP1//NPTN//NCK1//RAPGEF2//BMPR2//ACVR1//IFNG//POU3F2//OTX2//EDN1//IL2//ZNF268//AR//NEUROD1//BTC//CEBPB//FRZB//PPARG//BTG1//MYB//SOCS5//IL5//GATA2//VWC2//NEUROG1//NPNT//GJA1//IGF1//CCR1//POU4F1//TNF//C5AR1//VEGFB//ADAM12//UNG//ARX//DLL1//MTM1//PRKAA1//EFNA5//SLIT2//FLRT1//IL1RAP//WNT7A//DLG5//NRXN1//ZFPM2//CPEB3//FMR1//LRP8//AKAP6//AGTR2//MIEF2//DDHD2//BNIP3//CRKL//PLAA//CLASP2//LRP6//RFX3// |
| GO:0048731 | system development | Biological process | 188 | 4760 | 574 | 17653 | 1.21466781834685 | 0.00105061940938063 | 0.0559475516979622 | 2.9785545802043 | 0.327526132404181 | HAPLN1//EN1//FRZB//IGF1//BMP3//SOX4//WNT7A//ELK3//EREG//HOXA3//MEOX2//CCL2//VEGFB//MMRN2//AIMP1//CEBPB//ADCYAP1//SPO11//INHBB//DMRTA1//SOD1//ESR1//CRKL//SPHK2//TGFBR2//RAPGEF2//EDN1//ACVR1//ZFPM2//QKI//SMOC1//GATA2//NPNT//SLIT2//DCX//GJA1//LRP12//NR4A2//SPOCK1//FZD3//TMED10//SULF1//TSC1//ARID5B//FOXF2//DLG5//LRP6//FZD6//AK4//JARID2//MET//PPARG//BMPR2//APCDD1//ANP32B//PPP3CB//DLL1//RUNX3//ESCO2//GPR183//ITM2A//UBE4B//POU4F1//RFX3//CDK6//NEUROD1//BHLHE41//FUT9//CHRM2//DLG2//DPYSL2//EFNA5//MYT1L//FMR1//GLRB//MBNL1//NEUROG1//NTF3//POU3F2//ZIC5//OGN//ARX//DOK1//OTX2//PIK3CB//ENAH//KLF7//RPS6KA5//NRXN1//PCDHB10//NHLH2//HPCAL4//AGTR2//RAB3GAP1//CNTNAP2//PTPRG//ATXN1//CASP3//ZBTB18//MYOG//GSTM3//RNF38//TEX11//CBL//CLASP2//STRAP//LDLRAD4//PTF1A//SNAP25//STMN2//NPTN//NCK1//KBTBD8//MTPN//FLRT1//UNG//SPRED1//RFX4//C5AR1//LRP8//EMX2//HPRT1//CALM2//TACC2//BNIP3//KCNJ10//CBFB//TET2//HOXC8//HOXD1//IFNA1//PTPN22//IL2//TNF//HSD11B1//CCR1//AR//KRT27//DBNDD2//PSD//DSC2//KRTAP4-11//MAF//LRIG1//IFNG//MGST1//TSHZ1//SBF2//GPD2//IRF1//ARRDC3//CD8A//REG3G//MYB//SOCS5//IL5//NFE2//DDX6//VWC2//EGLN1//BTG1//ADAM12//GJB2//PHF14//MTM1//PRKAA1//ULK2//HOXB6//SLC35D1//NYAP1//HECW2//NOS1//IL1RAP//NFIA//SYBU//TBL1XR1//ZNF568//FOSL1//BTBD7//CPEB3//AKAP6//LDLR//TMOD1//KLHL3//ARID4B//YTHDF2//NLN//PLAA// |
| GO:0060389 | pathway-restricted SMAD protein phosphorylation | Biological process | 8 | 64 | 574 | 17653 | 3.84429442508711 | 0.00106058593850098 | 0.0560370523600166 | 2.97445413515816 | 0.0139372822299652 | INHBB//BMP3//BMPR2//ACVR1//STRAP//PMEPA1//LDLRAD4//TGFBR2// |
| GO:0042981 | regulation of apoptotic process | Biological process | 73 | 1566 | 574 | 17653 | 1.43363214830835 | 0.00107138187548241 | 0.0561686482471902 | 2.97005570484066 | 0.127177700348432 | IL2//ANP32B//EGLN3//PPARG//TNF//CASP3//ZNF268//ARL6IP1//IGF1//MDM4//MET//MYD88//ZFAND6//PRKAA1//TEX11//SPHK2//PTGFR//BCL2A1//BNIP3//BTC//UNG//WNT7A//CBL//ADCYAP1//PIK3CB//DLL1//CCL2//EDN1//LRP6//IFNG//GADD45A//CDK19//FRZB//NEUROD1//ARHGEF4//POU4F1//ZMAT3//SOD1//SOS2//SOX4//FOSL1//SLIT2//CEBPB//EN1//GRIK2//NTF3//NR4A2//C5AR1//MYB//TSC22D4//FZD3//DYNLL2//SERINC3//NCK1//IER3IP1//CFDP1//BTG1//GATA2//RAPGEF2//INHBB//ZMYND11//AR//ACVR1//AGTR2//IL20RA//ST18//ESR1//TNFAIP8L3//MAP3K1//PAK6//RBM25//BMP3//DLG5// |
| GO:0042327 | positive regulation of phosphorylation | Biological process | 52 | 1032 | 574 | 17653 | 1.54963806282581 | 0.00113412371476358 | 0.058162183330741 | 2.94533956827505 | 0.0905923344947735 | GADD45A//TNF//CRKL//MAP3K1//MAP3K7CL//LAMTOR3//IGF1//NTF3//PIK3CB//PRKAA1//SOD1//C5AR1//EFNA5//FMR1//NPTN//IFNG//EREG//MDFIC//CALM2//INHBB//BMP3//BMPR2//ACVR1//RAP2C//PAK6//TGFBR2//RPS6KA5//IFNA1//MET//PRKAR2B//IL2//EDN1//AR//TNFAIP8L3//CCNY//GPD1//ADCYAP1//RAPGEF2//DUSP22//WNT7A//IL5//YES1//LRP8//CCR1//GPR183//NPNT//PTPN22//CCL2//CCL8//ACSL1//MYOG//PTGES3// |
| GO:0007638 | mechanosensory behavior | Biological process | 4 | 15 | 574 | 17653 | 8.20116144018583 | 0.00113431774668672 | 0.058162183330741 | 2.94526527321814 | 0.00696864111498258 | NEUROG1//CNTNAP2//NRXN1//STRBP// |
| GO:0051091 | positive regulation of DNA binding transcription factor activity | Biological process | 18 | 245 | 574 | 17653 | 2.25950366209201 | 0.00113520748183614 | 0.058162183330741 | 2.94492475522725 | 0.0313588850174216 | LRP8//RPS6KA5//MTPN//IL1RAP//AR//MYD88//TNF//TRIM8//EDN1//ESR1//IL5//LRP6//NEUROD1//NEUROG1//NHLH2//PPARG//FOSL1//ARID5B// |
| GO:0050793 | regulation of developmental process | Biological process | 110 | 2563 | 574 | 17653 | 1.31992941633892 | 0.00115378813925892 | 0.0586696931263765 | 2.93787392982655 | 0.191637630662021 | FZD3//TGFBR2//BMPR2//CFDP1//BRWD1//CCL2//CLASP2//BNIP3//STRAP//LDLRAD4//FRZB//GATA2//PPARG//MYOG//BHLHE41//SNAP25//STMN2//ADCYAP1//NPTN//NCK1//RAPGEF2//PTPRG//SPOCK1//TSC1//DLL1//MTPN//SPRED1//SULF1//RFX4//SH3D19//EFNA5//GJA1//ACVR1//CCR1//DPYSL2//IFNG//WNT7A//MAF//SOD1//IGF1//POU3F2//OTX2//EDN1//IL2//CBFB//IRF1//RUNX3//CDK6//ANP32B//ZNF268//AR//NEUROD1//BTC//ZFPM2//TNF//CEBPB//BTG1//REG3G//MYB//SOCS5//IL5//NFE2//NTF3//DDX6//VWC2//NEUROG1//SMOC1//NPNT//POU4F1//EGLN1//C5AR1//VEGFB//ADAM12//UNG//ARX//MTM1//PRKAA1//ULK2//FMR1//HECW2//NOS1//SLIT2//EREG//MED28//FLRT1//IL1RAP//DLG5//NRXN1//JARID2//FZD6//ESR1//BTBD7//CPEB3//LRP8//AKAP6//PTF1A//LDLR//AGTR2//MEOX2//MMRN2//MIEF2//DDHD2//CRKL//YTHDF2//NLN//PLAA//LRP6//RFX3//GPR183//PHF14// |
| GO:0048666 | neuron development | Biological process | 53 | 1060 | 574 | 17653 | 1.53771777003484 | 0.00120759803334791 | 0.0609476529815815 | 2.91807760292523 | 0.0923344947735192 | OGN//POU4F1//WNT7A//SLIT2//ARX//DOK1//DPYSL2//EFNA5//OTX2//PIK3CB//ENAH//KLF7//RPS6KA5//NRXN1//CNTNAP2//SNAP25//STMN2//ADCYAP1//NPTN//NCK1//RAPGEF2//PTPRG//SPOCK1//TSC1//FLRT1//NR4A2//GATA2//HPRT1//UBE4B//LRP12//DBNDD2//PSD//FZD3//ULK2//PPP3CB//NYAP1//GJA1//NTF3//FMR1//HECW2//BMPR2//RUNX3//POU3F2//SOD1//CPEB3//IL2//DLG5//LRP8//IL1RAP//PLAA//EN1//MYT1L//NEUROD1// |
| GO:0031175 | neuron projection development | Biological process | 48 | 936 | 574 | 17653 | 1.57714643080497 | 0.00121899930789746 | 0.0610673505134113 | 2.91399654095783 | 0.0836236933797909 | OGN//POU4F1//WNT7A//SLIT2//ARX//DOK1//DPYSL2//EFNA5//OTX2//PIK3CB//ENAH//KLF7//RPS6KA5//NRXN1//SNAP25//STMN2//ADCYAP1//NPTN//NCK1//RAPGEF2//PTPRG//SPOCK1//TSC1//FLRT1//NR4A2//FZD3//ULK2//PPP3CB//NYAP1//CNTNAP2//GJA1//NTF3//HPRT1//FMR1//HECW2//BMPR2//POU3F2//SOD1//CPEB3//IL2//DLG5//LRP8//IL1RAP//PLAA//UBE4B//LRP12//DBNDD2//PSD// |
| GO:0035306 | positive regulation of dephosphorylation | Biological process | 7 | 51 | 574 | 17653 | 4.22118603538977 | 0.00123926014396283 | 0.0616258555413281 | 2.90683751749116 | 0.0121951219512195 | NPNT//MTMR9//AGTR2//CALM2//PPP1R15B//AKAP6//IFNG// |
| GO:0008219 | cell death | Biological process | 97 | 2216 | 574 | 17653 | 1.34619696474169 | 0.00127680937017017 | 0.0630296479595683 | 2.89387393880799 | 0.168989547038328 | BCL2A1//IL2//RTN3//EGLN3//GADD45A//ARL6IP1//SULF1//GJA1//IFNG//IRF1//MYD88//CHMP3//ZFAND6//XAF1//DRAM1//PAK6//DUSP22//BNIP3//TGFBR2//C5AR1//CASP3//AIMP1//EI24//ANP32B//PPARG//TNF//ZNF268//IGF1//MDM4//MET//PRKAA1//TEX11//SPHK2//PTGFR//BTC//UNG//WNT7A//CBL//UBE4B//ADCYAP1//ESR1//INHBB//TNFAIP8L3//MAP3K1//NTF3//RBM25//BMP3//DLG5//PIK3CB//DLL1//CCL2//EDN1//LRP6//USP28//CDK19//FRZB//NEUROD1//ARHGEF4//POU4F1//ZMAT3//SOD1//SOS2//SOX4//FOSL1//SLIT2//CEBPB//EN1//GRIK2//NR4A2//MYB//ZFPM2//NCK1//TSC22D4//DSC2//KRT27//MEOX2//BMPR2//FASTK//SIVA1//APPL1//FZD3//DYNLL2//EGLN1//TSC1//SERINC3//NCOA7//IER3IP1//CFDP1//BTG1//GATA2//RAPGEF2//ZMYND11//AR//ACVR1//AGTR2//IL20RA//ST18// |
| GO:0043067 | regulation of programmed cell death | Biological process | 73 | 1579 | 574 | 17653 | 1.42182897039321 | 0.00133753810007585 | 0.0655490592087897 | 2.87369383815381 | 0.127177700348432 | IL2//ANP32B//EGLN3//PPARG//TNF//CASP3//ZNF268//ARL6IP1//IGF1//MDM4//MET//MYD88//ZFAND6//PRKAA1//TEX11//SPHK2//PTGFR//BCL2A1//BNIP3//BTC//UNG//WNT7A//CBL//ADCYAP1//ESR1//INHBB//TNFAIP8L3//MAP3K1//NTF3//PAK6//RBM25//BMP3//DLG5//PIK3CB//DLL1//CCL2//EDN1//LRP6//IFNG//GADD45A//CDK19//FRZB//NEUROD1//ARHGEF4//POU4F1//ZMAT3//SOD1//SOS2//SOX4//FOSL1//SLIT2//CEBPB//EN1//GRIK2//NR4A2//C5AR1//MYB//TSC22D4//FZD3//DYNLL2//SERINC3//NCK1//IER3IP1//CFDP1//BTG1//GATA2//RAPGEF2//ZMYND11//AR//ACVR1//AGTR2//IL20RA//ST18// |
| GO:0009612 | response to mechanical stimulus | Biological process | 16 | 209 | 574 | 17653 | 2.35440041345048 | 0.00137070184744133 | 0.0665729620191255 | 2.86305700187916 | 0.0278745644599303 | STRBP//MTPN//GADD45A//GJA1//IRF1//MAP3K1//MYD88//BNIP3//NEUROG1//EDN1//CNTNAP2//NRXN1//CLCN6//PPARG//TGFBR2//FOSL1// |
| GO:0046660 | female sex differentiation | Biological process | 11 | 116 | 574 | 17653 | 2.91636128799712 | 0.00140568230113542 | 0.0665729620191255 | 2.85211282330944 | 0.0191637630662021 | CEBPB//ADCYAP1//SPO11//INHBB//DMRTA1//SOD1//ESR1//EREG//ARID5B//SLIT2//ZFPM2// |
| GO:0012501 | programmed cell death | Biological process | 92 | 2088 | 574 | 17653 | 1.35507696209967 | 0.00141521810451607 | 0.0665729620191255 | 2.84917662424677 | 0.160278745644599 | BCL2A1//IL2//RTN3//EGLN3//GADD45A//ARL6IP1//SULF1//GJA1//IFNG//IRF1//MYD88//CHMP3//ZFAND6//XAF1//DRAM1//PAK6//DUSP22//BNIP3//TGFBR2//C5AR1//CASP3//AIMP1//EI24//ANP32B//PPARG//TNF//ZNF268//IGF1//MDM4//MET//PRKAA1//TEX11//SPHK2//PTGFR//BTC//UNG//WNT7A//CBL//UBE4B//ADCYAP1//ESR1//INHBB//TNFAIP8L3//MAP3K1//NTF3//RBM25//BMP3//DLG5//PIK3CB//DLL1//CCL2//EDN1//LRP6//USP28//CDK19//FRZB//NEUROD1//ARHGEF4//POU4F1//ZMAT3//SOD1//SOS2//SOX4//FOSL1//SLIT2//CEBPB//EN1//GRIK2//NR4A2//MYB//TSC22D4//DSC2//KRT27//BMPR2//FASTK//SIVA1//APPL1//FZD3//DYNLL2//SERINC3//NCK1//IER3IP1//CFDP1//BTG1//GATA2//RAPGEF2//ZMYND11//AR//ACVR1//AGTR2//IL20RA//ST18// |
| GO:0010921 | regulation of phosphatase activity | Biological process | 14 | 171 | 574 | 17653 | 2.51790044216232 | 0.00142487965468325 | 0.0665729620191255 | 2.84622181461273 | 0.024390243902439 | TNF//NPNT//MTMR9//SPRED1//DLG2//PLEKHF2//PHACTR2//AGTR2//CALM2//PPP1R15B//AKAP6//UBXN2B//TSC1//IFNG// |
| GO:1902531 | regulation of intracellular signal transduction | Biological process | 85 | 1901 | 574 | 17653 | 1.37512898951038 | 0.00143396218405023 | 0.0665729620191255 | 2.84346230157956 | 0.14808362369338 | GADD45A//TNF//CRKL//MAP3K1//MAP3K7CL//LAMTOR3//IGF1//NTF3//PIK3CB//PRKAA1//SOD1//C5AR1//SPRED1//DUSP22//MDFIC//AKAP11//AKAP4//AKAP6//CBL//MTM1//TSC1//RRAGD//ARAP2//APPL1//AGFG1//PSD//RALGPS2//IFNA1//IFNG//ARHGEF4//SOS2//MET//DLG5//RNF125//CNOT7//IL2//ZFAND6//GJA1//MYD88//MIER1//TRIM8//ZMYND11//ESR1//EDN1//INHBB//PAK6//BMP3//AR//PTPN22//ATR//PEX5L//ADCYAP1//RAPGEF2//GRIK2//WNT7A//FLRT1//NEUROD1//SOCS5//IL5//RASGEF1A//ARHGAP1//ARHGAP12//CD2AP//SLIT2//EREG//TNFAIP8L3//FAM110C//BTC//OTUD3//NOS1//CALM2//CCR1//GPR183//NPNT//NPTN//CCL2//CCL8//RAD9B//MDM4//POU4F1//RBBP8//SERINC3//NCK1//BCL2A1//IL20RA// |
| GO:2000113 | negative regulation of cellular macromolecule biosynthetic process | Biological process | 75 | 1636 | 574 | 17653 | 1.40988793096104 | 0.00144075203582082 | 0.0665729620191255 | 2.84141075807656 | 0.130662020905923 | TSHZ1//ZBTB18//CEBPB//ZNF268//TCERG1//STRAP//KLF12//ARX//EDN1//CC2D1B//EN1//ESR1//CPEB3//MYT1L//RCOR1//ZFPM2//GATA2//CNOT7//HOXC8//HSBP1//IFNG//IRF2//AR//JARID2//ZNF568//MAF//MDM4//MYB//NFIA//NR4A2//KLF3//POU4F1//PPARG//DUSP22//ZBTB4//RBBP8//TNF//ZNF217//LRP8//BHLHE41//TBL1XR1//ARID5B//BHLHE40//RUNX3//PHF14//BAHD1//ATR//SNIP1//ELK3//EREG//FOXF2//MDFIC//IRF1//RASD1//RFX3//ATXN1//TSC22D4//BTAF1//RPS6KA5//FMR1//TSC1//ATAD2//MIER1//DCP2//CNOT6//ITM2A//POLR2K//CNOT6L//YTHDF2//QKI//CPEB2//NCK1//GJA1//BMPR2//CPEB1// |
| GO:0044093 | positive regulation of molecular function | Biological process | 79 | 1742 | 574 | 17653 | 1.39471531380887 | 0.00144879838356941 | 0.0665729620191255 | 2.83899204723795 | 0.137630662020906 | GADD45A//TNF//CRKL//MAP3K1//MAP3K7CL//LAMTOR3//IGF1//NTF3//PIK3CB//PRKAA1//SOD1//C5AR1//EREG//ANP32B//EGLN3//PPARG//CASP3//EFNA5//MDFIC//NPNT//ESR1//MTMR9//NOS1//RAPGEF2//PAK6//TGFBR2//STIM2//PLAA//AGTR2//CALM2//PPP1R15B//AKAP6//DNAJB4//LRP8//RPS6KA5//ADCYAP1//ARAP2//DENND1B//RAB3GAP1//AGFG1//ARHGAP1//RP2//CCL2//CCL8//EVI5//ARHGAP12//USP6NL//MET//PRKAR2B//NR4A2//EDN1//TNFAIP8L3//CCNY//IL5//LRP6//NEUROD1//NEUROG1//NHLH2//FOSL1//TRIM8//ARID5B//MTPN//IL1RAP//AR//MYD88//MID1IP1//DCUN1D4//ARRDC3//PTGES3//TNKS//IFNG//ACSL1//TSC1//FMR1//CNOT6//DYNLL2//GLRX//POU4F1//ST18// |
| GO:0016070 | RNA metabolic process | Biological process | 189 | 4821 | 574 | 17653 | 1.20567790307648 | 0.0014689461284481 | 0.0665729620191255 | 2.83299413106252 | 0.329268292682927 | NPAT//RRM2//TSHZ1//ZBTB18//CEBPB//ZNF268//TCERG1//STRAP//KLF12//ARX//EDN1//CC2D1B//EN1//ESR1//CPEB3//MYT1L//RCOR1//ZFPM2//GATA2//CNOT7//HOXC8//HSBP1//IFNG//IRF2//AR//JARID2//ZNF568//MAF//MDM4//MYB//NFIA//NR4A2//KLF3//POU4F1//PPARG//DUSP22//ZBTB4//RBBP8//TNF//ZNF217//LRP8//BHLHE41//TBL1XR1//ARID5B//BHLHE40//RUNX3//PHF14//DCP2//CNOT6L//PAN3//CNOT6//FMR1//MBNL1//SMU1//RBM25//SRSF2//POLR2K//RBM41//SNRPN//NOB1//ELK3//TFEC//FOXJ3//FOXF2//PTF1A//HIVEP2//MACC1//IRF1//MEOX2//MYBL1//NEUROD1//NHLH2//OTX2//POU3F2//RFX4//BMPR2//SOX4//BTF3//CARF//FOSL1//KLF7//CBFB//NMI//BAHD1//ZMYND11//KLF17//NCOA7//ZNF782//ZNF800//ZFP30//KDM2A//EPC2//HBP1//ATAD2//MDFIC//HOXA3//HOXB6//HOXD1//ZNF680//NEUROG1//NFE2//COMMD2//VGLL1//ARID4B//BRWD1//PRKAA1//TRERF1//SLC2A4RG//ZNF630//ZBTB26//MIER1//RFX3//ATXN1//DMRTA1//SMARCD2//ZNF3//ZNF131//ZNF134//ZNF208//ZNF227//ZNF655//MED28//TSC22D4//PCGF5//LCOR//ST18//NRBF2//MLLT6//PAK6//BTG1//RHOXF2B//SNIP1//CBL//RPS6KA5//MED6//EMX2//BOLA3//NR3C2//RFX7//RHOXF2//BLZF1//ELAVL4//RTCA//SCAF8//CPEB1//QKI//DTD1//AIMP1//G3BP2//EREG//DDX6//INTS7//AGTR2//IGF1//IL5//LRP6//NOS1//PPP3CB//WNT7A//ACVR1//RASD1//BTAF1//BMP3//SULF1//VWC2//MYOG//C5AR1//NABP1//EGLN1//FZD6//FASTK//YTHDF2//ADCYAP1//DLL1//IL2//MET//NCK1//ARMCX3//TET2//YES1//NFATC2IP//TNKS//TRIM8//MTPN//IL1RAP//MYD88//EGLN3//RAP2C//NPNT// |
| GO:0048583 | regulation of response to stimulus | Biological process | 169 | 4243 | 574 | 17653 | 1.22495547082672 | 0.0014713288106192 | 0.0665729620191255 | 2.83229026086875 | 0.294425087108014 | GADD45A//TNF//CRKL//MAP3K1//MAP3K7CL//LAMTOR3//IGF1//NTF3//PIK3CB//PRKAA1//SOD1//C5AR1//SPRED1//DUSP22//PPP3CB//CBFB//ARL6IP1//SKP1//RPS6KA5//MYD88//PPARG//SLIT2//REG3G//PGC//IRF1//SAMSN1//ADCYAP1//EREG//CBL//SOCS5//VSIG4//NCK1//MDFIC//OTX2//ARHGAP1//SH3BGR//SH3BGRL//MET//ACVR1//EDN1//C1QTNF9//IFNA1//IFNG//MACC1//IL2//IL5//INHBB//OGN//IL36G//CCL28//CCL2//CCL8//BMP3//BTC//VEGFB//WNT7A//CALCB//AIMP1//MTPN//JARID2//AKAP11//AKAP4//AKAP6//BMPR2//PMEPA1//LDLRAD4//MYOG//RFX4//DENND1B//PTPN22//ESR1//SULF1//SOX4//APCDD1//FRZB//SHISA2//ENPP4//NPNT//STRAP//TGFBR2//VWC2//MMRN2//MTM1//TSC1//RRAGD//ARAP2//APPL1//AGFG1//PSD//RALGPS2//UBA5//AR//ARHGEF4//SOS2//DLG5//SYBU//YES1//RNF125//EPS15//CNOT7//ZFAND6//GJA1//MIER1//TRIM8//ZMYND11//CD84//GATA2//PAK6//ATR//PEX5L//RAPGEF2//MYB//DOK1//NPTN//YTHDF2//DLL1//NMI//UNG//RAET1E//GRIK2//FLRT1//NEUROD1//RASGEF1A//CDK19//LDLR//TREM1//CD8A//GCSAML//ARHGAP12//CD2AP//AGTR2//TNFAIP8L3//FAM110C//OTUD3//NRXN1//NOS1//CALM2//CCNY//LRP6//EFNA5//CCR1//GPR183//ARRDC3//FZD6//BNIP3//TBL1XR1//TNKS//PATE1//C5ORF30//PTGES3//HSPB8//HSBP1//DYNLL2//RAD9B//MDM4//POU4F1//RBBP8//SERINC3//NCOA7//TRPC3//CLASP2//PPP1R15B//PHF14//CDK6//FMR1//NR4A2//BCL2A1//IL20RA//ST18// |
| GO:0072148 | epithelial cell fate commitment | Biological process | 4 | 16 | 574 | 17653 | 7.68858885017422 | 0.00147381587310429 | 0.0665729620191255 | 2.83155677040484 | 0.00696864111498258 | NEUROD1//DLL1//ACVR1//ARX// |
| GO:0045844 | positive regulation of striated muscle tissue development | Biological process | 9 | 83 | 574 | 17653 | 3.33480962176231 | 0.00147655541961686 | 0.0665729620191255 | 2.83075024799988 | 0.0156794425087108 | DLL1//MTM1//PRKAA1//MYOG//ZFPM2//EDN1//IGF1//AKAP6//GJA1// |
| GO:0048636 | positive regulation of muscle organ development | Biological process | 9 | 83 | 574 | 17653 | 3.33480962176231 | 0.00147655541961686 | 0.0665729620191255 | 2.83075024799988 | 0.0156794425087108 | GJA1//DLL1//MTM1//PRKAA1//MYOG//ZFPM2//EDN1//IGF1//AKAP6// |
| GO:1901863 | positive regulation of muscle tissue development | Biological process | 9 | 84 | 574 | 17653 | 3.29510950721752 | 0.00160716145530133 | 0.0719816749814761 | 2.79394049185878 | 0.0156794425087108 | GJA1//DLL1//MTM1//PRKAA1//MYOG//ZFPM2//EDN1//IGF1//AKAP6// |
| GO:0010941 | regulation of cell death | Biological process | 77 | 1696 | 574 | 17653 | 1.39627674873447 | 0.00161934545945484 | 0.0720502193571913 | 2.7906604921172 | 0.134146341463415 | IL2//ANP32B//EGLN3//PPARG//TNF//CASP3//ZNF268//ARL6IP1//IGF1//MDM4//MET//MYD88//ZFAND6//PRKAA1//TEX11//SPHK2//PTGFR//BCL2A1//BNIP3//BTC//UNG//WNT7A//CBL//ADCYAP1//ESR1//INHBB//TNFAIP8L3//MAP3K1//NTF3//PAK6//RBM25//BMP3//DLG5//PIK3CB//DLL1//CCL2//EDN1//LRP6//IFNG//GADD45A//CDK19//FRZB//NEUROD1//ARHGEF4//POU4F1//ZMAT3//SOD1//SOS2//SOX4//FOSL1//SLIT2//CEBPB//EN1//GRIK2//NR4A2//C5AR1//MYB//ZFPM2//NCK1//TSC22D4//FZD3//DYNLL2//EGLN1//TSC1//SERINC3//NCOA7//IER3IP1//CFDP1//BTG1//GATA2//RAPGEF2//ZMYND11//AR//ACVR1//AGTR2//IL20RA//ST18// |
| GO:0065009 | regulation of molecular function | Biological process | 138 | 3373 | 574 | 17653 | 1.25825705463865 | 0.0016991221151966 | 0.0751056396410105 | 2.7697754074463 | 0.240418118466899 | GADD45A//DIRAS3//TNF//CRKL//MAP3K1//MAP3K7CL//LAMTOR3//IGF1//NTF3//PIK3CB//PRKAA1//SOD1//C5AR1//SPRED1//DUSP22//EREG//CBL//SOCS5//FLRT1//LRP6//NCK1//DBNDD2//ANP32B//EGLN3//PPARG//CASP3//EFNA5//ADCYAP1//G3BP2//MDFIC//EDN1//C1QTNF9//IFNA1//IFNG//MACC1//IL2//IL5//INHBB//OGN//IL36G//CCL28//CCL2//CCL8//BMP3//BTC//VEGFB//WNT7A//CALCB//AIMP1//NPNT//ESR1//MTMR9//DLG2//PLEKHF2//A2ML1//WFDC13//SPOCK1//IQGAP2//CPEB2//TSC1//SLIT2//GJA1//NOS1//CDK6//RAPGEF2//PAK6//TGFBR2//STIM2//PLAA//PHACTR2//AGTR2//CALM2//PPP1R15B//AKAP6//PXK//DNAJB4//LRP8//RPS6KA5//RAB3GAP1//ARHGAP1//SBF2//ARAP2//DENND1B//AGFG1//RP2//EVI5//ARHGAP12//USP6NL//MET//PRKAR2B//BTAF1//NR4A2//ARL6IP1//EGLN1//FZD6//BHLHE40//PTPN22//NRBF2//TNFAIP8L3//UBXN2B//CCNY//AR//POU4F1//NEUROD1//NEUROG1//NHLH2//FOSL1//TRIM8//ARID5B//MTPN//IL1RAP//MYD88//MID1IP1//DCUN1D4//ARRDC3//PTGES3//TNKS//RAP2C//ACSL1//PATE1//FMR1//KCNS2//MTRR//CNOT6//DYNLL2//HECW2//GLRX//ST18//RAPGEF4//CSF2RA//CRISP1//RASGEF1A//ARHGEF4//HPCAL4//RALGPS2//PSD//SOS2//NRXN1// |
| GO:0060412 | ventricular septum morphogenesis | Biological process | 6 | 40 | 574 | 17653 | 4.61315331010453 | 0.00172418814679242 | 0.0757187301088126 | 2.76341534485972 | 0.0104529616724739 | TGFBR2//ZFPM2//EGLN1//BMPR2//SOX4//ACVR1// |
| GO:0035556 | intracellular signal transduction | Biological process | 120 | 2871 | 574 | 17653 | 1.28544850159653 | 0.00174520153145464 | 0.0761470835950176 | 2.75815441458161 | 0.209059233449477 | CSF2RA//SPRED1//EREG//RASGEF1A//IL2//IL5//MAP3K1//MET//MAP3K7CL//CCL2//BTC//TNF//LAMTOR3//RAPGEF2//GADD45A//CRKL//IGF1//NTF3//PIK3CB//PRKAA1//SOD1//C5AR1//DUSP22//CNOT6L//CNOT7//MDM4//CNOT6//SOX4//ADCYAP1//PTGFR//EDN1//SNIP1//MDFIC//NMI//SOCS5//AGTR2//NEUROD1//NOS1//RASD1//RAPGEF4//ARHGAP1//RALGPS2//SOS2//DIRAS3//DOK1//USP28//G3BP2//PAK6//CASP3//BCL2A1//AKAP11//AKAP4//AKAP6//CALM2//NYAP1//CBL//TNFAIP8L3//MTM1//TSC1//RRAGD//ARAP2//APPL1//AGFG1//PSD//RAB12//RAB30//RAB9B//RAB6B//RAB28//RAP2C//IFNA1//IFNG//PPP3CB//ARHGEF4//DLG5//SKP1//RNF125//ZFAND6//GJA1//MYD88//MIER1//TRIM8//ZMYND11//ESR1//INHBB//BMP3//AR//PTPN22//ATR//PEX5L//GRIK2//WNT7A//FLRT1//ARHGAP12//CD2AP//SLIT2//FAM110C//OTUD3//CEBPB//CCR1//GPR183//NPNT//NPTN//CCL8//FIBIN//POU4F1//ZMAT3//RAD9B//RBBP8//SERINC3//NCK1//BNIP3//IL20RA//DGKH//DCX//TREM1//PRKAR2B//STK33//DGKE//RPS6KA5// |
| GO:0008285 | negative regulation of cell proliferation | Biological process | 39 | 732 | 574 | 17653 | 1.63855172216828 | 0.00176618511938871 | 0.0765686536052939 | 2.7529637786092 | 0.0679442508710801 | SULF1//GJA1//TNF//AIMP1//BMPR2//CASP3//CEBPB//DLG5//AGTR2//IFNG//OGN//PPARG//IL2//CDK6//STRAP//EREG//AR//RUNX3//JARID2//TGFBR2//ADCYAP1//GATA2//CCL8//PHF14//ZNF268//FRZB//TES//MTBP//DLL1//CNOT7//IRF1//MDM4//MYOG//SOX4//BTG1//TSC1//FOSL1//RAPGEF2//ST18// |
| GO:0010638 | positive regulation of organelle organization | Biological process | 35 | 637 | 574 | 17653 | 1.68979974729104 | 0.00180428198919052 | 0.0777220324388248 | 2.74369558609627 | 0.0609756097560976 | CNOT6L//PAN3//CNOT6//NCK1//MET//CLIP1//STMN2//NEUROG1//MIER1//ATR//TNKS//CCT6A//FMR1//RPS6KA5//IQGAP2//ARFIP1//NOS1//EDN1//EREG//IGF1//BTC//TSC1//MYB//JARID2//IL5//TNF//MIEF2//DDHD2//BNIP3//DYNLL2//CALCOCO2//RAB3GAP1//PRKAA1//SNIP1//NTF3// |
| GO:0006029 | proteoglycan metabolic process | Biological process | 9 | 86 | 574 | 17653 | 3.2184790535613 | 0.00189614043935856 | 0.0811620113378604 | 2.72212949939263 | 0.0156794425087108 | NDST3//HS3ST5//IGF1//BMPR2//SULF1//DSEL//SLC35D1//CHST7//IDS// |
| GO:0009892 | negative regulation of metabolic process | Biological process | 127 | 3075 | 574 | 17653 | 1.27017988158976 | 0.0019387303442158 | 0.0824631026284997 | 2.71248259224314 | 0.221254355400697 | TSHZ1//ZBTB18//CEBPB//ZNF268//TCERG1//STRAP//KLF12//ARX//EDN1//CC2D1B//EN1//ESR1//CPEB3//MYT1L//RCOR1//ZFPM2//GATA2//CNOT7//HOXC8//HSBP1//IFNG//IRF2//AR//JARID2//ZNF568//MAF//MDM4//MYB//NFIA//NR4A2//KLF3//POU4F1//PPARG//DUSP22//ZBTB4//RBBP8//TNF//ZNF217//LRP8//BHLHE41//TBL1XR1//ARID5B//BHLHE40//RUNX3//PHF14//DCP2//SPRED1//CNOT6L//PAN3//CNOT6//IL2//LRP6//PPP1R15B//SLIT2//CBL//SOCS5//BAHD1//GADD45A//FLRT1//NCK1//DBNDD2//ATR//SNIP1//PTPN22//MET//MTMR9//CCR1//GJA1//LDLR//POU3F2//BNIP3//CALM2//DLG2//PLEKHF2//A2ML1//WFDC13//SPOCK1//TSC1//ELK3//EREG//FOXF2//MDFIC//IRF1//RASD1//RFX3//ATXN1//TSC22D4//BTAF1//RPS6KA5//FMR1//EGLN1//SPOPL//SOX4//CDK6//ATAD2//MIER1//MTM1//PHACTR2//ITM2A//ARL6IP1//YTHDF2//DDX6//NMI//SOD1//CASP3//MYOG//PRKAA1//ZMYND11//RAPGEF2//INHBB//NTF3//SAMSN1//PMEPA1//LDLRAD4//POLR2K//QKI//MYD88//PLAA//CPEB2//NCOA7//TFEC//NPAT//SRSF2//TNKS//BMPR2//PRKAR2B//CPEB1// |
| GO:0030900 | forebrain development | Biological process | 23 | 366 | 574 | 17653 | 1.93265074922412 | 0.00207813574667641 | 0.0873125120482594 | 2.68232608714122 | 0.0400696864111498 | RFX4//LRP8//CDK6//EMX2//NEUROD1//CNTNAP2//HPRT1//ARX//TSC1//CASP3//LRP6//POU3F2//SLIT2//WNT7A//RAB3GAP1//RAPGEF2//GATA2//ADCYAP1//NR4A2//POU4F1//TACC2//BNIP3//OTX2// |
| GO:0051240 | positive regulation of multicellular organismal process | Biological process | 73 | 1606 | 574 | 17653 | 1.39792524548622 | 0.00208518748930174 | 0.0873125120482594 | 2.68085488941523 | 0.127177700348432 | EREG//SOD1//TNF//FZD3//TGFBR2//AGTR2//EDN1//GJA1//NPNT//SLC1A1//SULF1//C5AR1//BMPR2//WNT7A//MTPN//IGF1//CLASP2//IFNG//STMN2//ADCYAP1//NPTN//NCK1//RAPGEF2//LDLR//MYOG//INHBB//ENPP4//ACVR1//PTPN22//IRF1//MYD88//POLR2K//IL2//IL1RAP//CEBPB//RNF125//POU3F2//OTX2//MYB//SOCS5//IL5//GATA2//VWC2//NEUROD1//NEUROG1//CCR1//POU4F1//BTG1//VEGFB//ADAM12//UNG//ARX//DLL1//MTM1//PRKAA1//EFNA5//PPARG//SLIT2//FLRT1//DLG5//NRXN1//ZFPM2//AR//CPEB3//FMR1//LRP8//AKAP6//NOS1//TRPC3//PLAA//LRP6//RFX3//MET// |
| GO:0098739 | import across plasma membrane | Biological process | 8 | 71 | 574 | 17653 | 3.46527948176866 | 0.00209147226849298 | 0.0873125120482594 | 2.67954788949613 | 0.0139372822299652 | ARL6IP1//KCNJ10//SLC1A1//FAM155A//STEAP2//ARHGAP1//SLC2A5//KCNJ3// |
| GO:0048584 | positive regulation of response to stimulus | Biological process | 100 | 2334 | 574 | 17653 | 1.31766732650801 | 0.00211598280893511 | 0.087793814336369 | 2.6744878649993 | 0.174216027874564 | GADD45A//TNF//CRKL//MAP3K1//MAP3K7CL//LAMTOR3//IGF1//NTF3//PIK3CB//PRKAA1//SOD1//C5AR1//ARL6IP1//SKP1//RPS6KA5//MYD88//REG3G//PGC//EREG//VSIG4//MDFIC//ARHGAP1//SH3BGR//SH3BGRL//INHBB//BMP3//BMPR2//ACVR1//CBL//MYOG//DENND1B//PTPN22//NCK1//SULF1//SOX4//ENPP4//NPNT//RRAGD//IFNG//AR//ESR1//IRF1//DLG5//PPP3CB//SYBU//YES1//RNF125//IL2//GJA1//MIER1//TRIM8//GATA2//EDN1//MET//TGFBR2//SPRED1//ATR//ADCYAP1//RAPGEF2//MYB//SOCS5//DOK1//NPTN//DLL1//UNG//RAET1E//DUSP22//WNT7A//IL5//RASGEF1A//CDK19//LDLR//GCSAML//CBFB//SLIT2//VEGFB//SOS2//TNFAIP8L3//FAM110C//BTC//CALM2//AKAP6//CCR1//GPR183//CCL2//CCL8//ARRDC3//BNIP3//LRP6//TBL1XR1//TNKS//NOS1//DYNLL2//SERINC3//TRPC3//NRXN1//FMR1//AGTR2//IL20RA//ST18// |
| GO:0045446 | endothelial cell differentiation | Biological process | 10 | 105 | 574 | 17653 | 2.9289862286378 | 0.00221233116066473 | 0.0912316807291193 | 2.65514986358588 | 0.0174216027874564 | MET//GSTM3//WNT7A//BTG1//BMPR2//DLL1//RAPGEF2//ACVR1//RAP2C//TNF// |
| GO:0010720 | positive regulation of cell development | Biological process | 29 | 504 | 574 | 17653 | 1.76959584646867 | 0.00223269299886576 | 0.0913498573300052 | 2.65117098945192 | 0.0505226480836237 | FZD3//STMN2//ADCYAP1//NPTN//NCK1//RAPGEF2//IL5//GATA2//VWC2//NEUROD1//NEUROG1//EFNA5//PPARG//MYOG//BMPR2//SLIT2//MYB//EDN1//CPEB3//FMR1//IL2//DLG5//LRP8//IGF1//AKAP6//IL1RAP//CRKL//PLAA//RFX3// |
| GO:0003158 | endothelium development | Biological process | 11 | 123 | 574 | 17653 | 2.75038950737939 | 0.00224972570943255 | 0.0913498573300052 | 2.64787042861816 | 0.0191637630662021 | MET//GSTM3//WNT7A//BTG1//BMPR2//DLL1//RAPGEF2//ACVR1//RAP2C//TNF//GJA1// |
| GO:0001934 | positive regulation of protein phosphorylation | Biological process | 49 | 990 | 574 | 17653 | 1.52218526730722 | 0.00225671196256224 | 0.0913498573300052 | 2.64652386894733 | 0.0853658536585366 | GADD45A//TNF//CRKL//MAP3K1//MAP3K7CL//LAMTOR3//IGF1//NTF3//PIK3CB//PRKAA1//SOD1//C5AR1//EREG//EFNA5//MDFIC//CALM2//INHBB//BMP3//BMPR2//ACVR1//RAP2C//PAK6//TGFBR2//FMR1//RPS6KA5//IFNA1//IFNG//PRKAR2B//IL2//EDN1//AR//MET//CCNY//ADCYAP1//RAPGEF2//DUSP22//WNT7A//IL5//YES1//LRP8//CCR1//GPR183//NPNT//PTPN22//NPTN//TNFAIP8L3//CCL2//CCL8//ACSL1// |
| GO:0007611 | learning or memory | Biological process | 17 | 240 | 574 | 17653 | 2.17843350754936 | 0.00226922608774817 | 0.0913498573300052 | 2.64412223229051 | 0.029616724738676 | CNTNAP2//PPP3CB//PRKAR2B//ATXN1//FOSL1//NRXN1//CEBPB//NTF3//KCNK10//CPEB3//LDLR//SNAP25//DDHD2//NPTN//EN1//NEUROG1//CASP3// |
| GO:0046883 | regulation of hormone secretion | Biological process | 18 | 261 | 574 | 17653 | 2.12099002763427 | 0.00229115397846809 | 0.0916868305111224 | 2.63994572279145 | 0.0313588850174216 | AGTR2//RAPGEF4//IFNG//NEUROD1//PPP3CB//RFX3//SNAP25//TNF//INHBB//EDN1//GJA1//SOX4//SYBU//ADCYAP1//EFNA5//NRXN1//AIMP1//MYB// |
| GO:0009967 | positive regulation of signal transduction | Biological process | 72 | 1586 | 574 | 17653 | 1.39616241415522 | 0.00231113769336746 | 0.0919424954132008 | 2.63617417927715 | 0.125435540069686 | GADD45A//TNF//CRKL//MAP3K1//MAP3K7CL//LAMTOR3//IGF1//NTF3//PIK3CB//PRKAA1//SOD1//C5AR1//EREG//MDFIC//INHBB//BMP3//BMPR2//ACVR1//CBL//SULF1//SOX4//NPNT//RRAGD//AR//PTPN22//DLG5//IFNG//IL2//GJA1//MYD88//MIER1//TRIM8//EDN1//MET//SPRED1//ATR//ADCYAP1//RAPGEF2//DOK1//NPTN//DLL1//DUSP22//WNT7A//IL5//RASGEF1A//SOS2//ESR1//TNFAIP8L3//FAM110C//BTC//CALM2//AKAP6//CCR1//GPR183//CCL2//CCL8//ARRDC3//BNIP3//LRP6//TBL1XR1//TNKS//NOS1//DYNLL2//SERINC3//NCK1//NRXN1//AGTR2//IL20RA//ST18//ARHGAP1//SH3BGR//SH3BGRL// |
| GO:0032516 | positive regulation of phosphoprotein phosphatase activity | Biological process | 4 | 18 | 574 | 17653 | 6.83430120015486 | 0.00235327097457782 | 0.0930711789536245 | 2.62832806177929 | 0.00696864111498258 | AGTR2//CALM2//PPP1R15B//AKAP6// |
| GO:1901700 | response to oxygen-containing compound | Biological process | 71 | 1565 | 574 | 17653 | 1.39524551658114 | 0.00252043924597561 | 0.0991030850030991 | 2.59852376656765 | 0.123693379790941 | SOD1//CHRM2//APPL1//GJA1//IGF1//NEUROD1//TGFBR2//CASP3//SLC2A5//EDN1//CEBPB//CPEB3//RRAGD//CPEB1//TNF//LAMTOR3//PTPN22//CCL2//ESR1//GJB2//PTGFR//RBBP8//WNT7A//C5AR1//MGST1//NOS1//PPARG//FOSL1//TSC1//CPEB2//INHBB//SRSF2//TRPC3//CBL//ACSL1//PPP3CB//SYBU//AGTR2//PPP1R15B//GLRB//REG3G//EREG//LRP6//BTG1//ADCYAP1//IL2//NCK1//SLIT2//EFNA5//NRXN1//MYB//PRKAA1//BNIP3//CDK19//PLAA//MYOG//YES1//GPD1//AKAP6//RAPGEF2//SOX4//NR4A2//PRKAR2B//POU4F1//AR//LRP8//LDLR//FIBIN//EGLN1//MET//KLF3// |
| GO:0035270 | endocrine system development | Biological process | 11 | 125 | 574 | 17653 | 2.70638327526132 | 0.00255465051895743 | 0.0991724709740116 | 2.59266850377568 | 0.0191637630662021 | RFX3//CDK6//DLL1//NEUROD1//ADCYAP1//GATA2//POU3F2//ARID5B//HOXA3//SOX4//CRKL// |
| GO:0034390 | smooth muscle cell apoptotic process | Biological process | 5 | 30 | 574 | 17653 | 5.12572590011614 | 0.00259444930381588 | 0.0991724709740116 | 2.58595481110824 | 0.00871080139372822 | EDN1//IGF1//LRP6//IFNG//PPARG// |
| GO:0034391 | regulation of smooth muscle cell apoptotic process | Biological process | 5 | 30 | 574 | 17653 | 5.12572590011614 | 0.00259444930381588 | 0.0991724709740116 | 2.58595481110824 | 0.00871080139372822 | EDN1//IGF1//LRP6//IFNG//PPARG// |
| GO:0032268 | regulation of cellular protein metabolic process | Biological process | 116 | 2792 | 574 | 17653 | 1.27775975160488 | 0.00259740566865328 | 0.0991724709740116 | 2.58546021598532 | 0.202090592334495 | GADD45A//DIRAS3//TNF//CRKL//MAP3K1//MAP3K7CL//LAMTOR3//IGF1//NTF3//PIK3CB//PRKAA1//SOD1//C5AR1//SPRED1//DUSP22//SECISBP2L//IL2//LRP6//PPP1R15B//SLIT2//EFNA5//FMR1//NPTN//IFNG//EREG//CBL//SOCS5//MTPN//DDX6//TSC1//KBTBD8//EIF3B//FLRT1//NCK1//DBNDD2//ANP32B//EGLN3//PPARG//CASP3//MDFIC//SNIP1//CALM2//INHBB//BMP3//BMPR2//ACVR1//A2ML1//WFDC13//SPOCK1//CPEB3//PRR16//SOX4//CNOT7//JARID2//SPOPL//CDK6//RAP2C//PAK6//TGFBR2//EDN1//WNT7A//MTM1//RNF180//RNF125//LONRF3//LONRF1//PHACTR2//AGTR2//AKAP6//RPS6KA5//IFNA1//PRKAR2B//GATA2//NOS1//CNOT6//ARL6IP1//AR//MET//PTPN22//UBXN2B//CCNY//ADCYAP1//RAPGEF2//GRIK2//ZMYND11//IL5//YES1//LRP8//SAMSN1//SH3D19//DCUN1D4//ARRDC3//MYB//STRAP//PMEPA1//LDLRAD4//POLR2K//CNOT6L//YTHDF2//QKI//CCR1//GPR183//NPNT//TNFAIP8L3//CCL2//CCL8//ACSL1//PLAA//CPEB2//NCOA7//NMI//RAB3GAP1//ESR1//LDLR//CPEB1//ST18// |
| GO:0021543 | pallium development | Biological process | 13 | 163 | 574 | 17653 | 2.45280135097582 | 0.00261894408068517 | 0.0991724709740116 | 2.58187377446867 | 0.0226480836236934 | LRP8//CDK6//EMX2//NEUROD1//TSC1//CASP3//POU3F2//ARX//SLIT2//TACC2//CNTNAP2//LRP6//BNIP3// |
| GO:0043170 | macromolecule metabolic process | Biological process | 346 | 9622 | 574 | 17653 | 1.10590386288101 | 0.00262175207383055 | 0.0991724709740116 | 2.58140837979246 | 0.602787456445993 | RAD9B//IGF1//NFIA//ATR//WRNIP1//RBBP8//RRM2//DTD1//GADD45A//DIRAS3//NPAT//TSHZ1//ZBTB18//CEBPB//ZNF268//TCERG1//STRAP//KLF12//ARX//EDN1//CC2D1B//EN1//ESR1//CPEB3//MYT1L//RCOR1//ZFPM2//GATA2//CNOT7//HOXC8//HSBP1//IFNG//IRF2//AR//JARID2//ZNF568//MAF//MDM4//MYB//NR4A2//KLF3//POU4F1//PPARG//DUSP22//ZBTB4//TNF//ZNF217//LRP8//BHLHE41//TBL1XR1//ARID5B//BHLHE40//RUNX3//PHF14//CSF2RA//SPRED1//EREG//RASGEF1A//IL2//IL5//MAP3K1//MET//MAP3K7CL//CCL2//BTC//LAMTOR3//RAPGEF2//DCP2//CRKL//NTF3//PIK3CB//PRKAA1//SOD1//C5AR1//UBE4B//RNF145//ZNRF2//RNF180//RNF125//UBE2W//HECW2//SKP1//THOP1//UBE2G1//LONRF3//CBFB//TNKS//CBL//UBE3B//LONRF1//CNOT6L//PAN3//CNOT6//FMR1//MBNL1//SMU1//RBM25//SRSF2//POLR2K//RBM41//SNRPN//NOB1//SPO11//SYCP1//TEX11//PTGES3//NABP1//SECISBP2L//CAMLG//EPS15//LRP6//PPP1R15B//SLIT2//EFNA5//NPTN//ELK3//TFEC//FOXJ3//FOXF2//PTF1A//HIVEP2//MACC1//IRF1//MEOX2//MYBL1//NEUROD1//NHLH2//OTX2//POU3F2//RFX4//BMPR2//SOX4//BTF3//CARF//FOSL1//KLF7//NMI//SOCS5//RNF38//KLHL3//SPOPL//DCAF8//TRIM8//KBTBD8//CHST7//CHST1//HS3ST5//IDS//GMNC//ESCO2//EPC2//USP28//UNG//KDM2A//MTRR//CASP3//BAHD1//ARID4B//ZMYND11//KLF17//NCOA7//ZNF782//ZNF800//ZFP30//HBP1//ATAD2//MDFIC//HOXA3//HOXB6//HOXD1//ZNF680//NEUROG1//NFE2//COMMD2//VGLL1//BRWD1//TRERF1//SLC2A4RG//ZNF630//ZBTB26//MIER1//RFX3//ATXN1//DMRTA1//SMARCD2//ZNF3//ZNF131//ZNF134//ZNF208//ZNF227//ZNF655//MED28//TSC22D4//PCGF5//LCOR//ST18//NRBF2//MLLT6//PAK6//BTG1//RHOXF2B//SNIP1//RPS6KA5//MED6//EMX2//BOLA3//NR3C2//RFX7//RHOXF2//BLZF1//ELAVL4//RTCA//SCAF8//CPEB1//QKI//AGFG1//CPEB2//MRPL35//METTL17//EIF3B//MTPN//DDX6//TSC1//AIMP1//YES1//IMMP1L//CDK6//FASTK//PDIK1L//CDK19//PPP3CB//CCL8//TGFBR2//ACVR1//FLRT1//NCK1//DBNDD2//PTPN22//MTM1//CPPED1//FUT9//FUT2//ST8SIA5//MAN1A1//ST8SIA3//CCDC126//TET2//VEGFB//PIGP//PIGA//GGT6//PRSS55//MEP1A//DESI2//PGC//NLN//NPEPL1//ADAM12//ADAM18//LMLN//GPHN//ANP32B//EGLN3//VSIG4//DLL1//G3BP2//RAB3GAP1//GJA1//LDLR//MYD88//PTGFR//CCR1//CALM2//INHBB//BMP3//A2ML1//WFDC13//SPOCK1//NDST3//PRR16//INTS7//PIGS//GNPTG//AGTR2//NOS1//WNT7A//RASD1//BTAF1//OTUD3//WDR20//NFATC2IP//VKORC1L1//STK33//OGN//EGLN1//DCUN1D4//MSRB3//SULF1//DSEL//SLC35D1//VWC2//CDC27//RAP2C//CCT6A//PHACTR2//AKAP6//IFNA1//PRKAR2B//PTPRG//PTP4A1//CDC14A//BNIP3//IL1RAP//DCTPP1//MYOG//ITM2A//TDRD5//ARL6IP1//UBXN2B//CD2AP//PLAA//VPS37A//FZD6//YTHDF2//ARSK//RAB12//CCNY//ADCYAP1//ARMCX3//GRIK2//ULK2//SAMSN1//SH3D19//ARRDC3//MTMR9//PMEPA1//LDLRAD4//ATG16L1//GPR183//NPNT//TNFAIP8L3//UBA5//ACSL1//GLRX//SNAP25//TMED10// |
| GO:0071496 | cellular response to external stimulus | Biological process | 20 | 307 | 574 | 17653 | 2.00354106844931 | 0.00262485173803757 | 0.0991724709740116 | 2.58089522222778 | 0.0348432055749129 | DSC2//INHBB//RRAGD//BMPR2//PLEKHF2//MTPN//GADD45A//GJA1//IRF1//MAP3K1//MYD88//BNIP3//NR4A2//FOSL1//PRKAA1//DCTPP1//PPARG//SOD1//TSC1//CBL// |
| GO:0008283 | cell proliferation | Biological process | 90 | 2078 | 574 | 17653 | 1.33199806836512 | 0.00265416138841649 | 0.0997227414992262 | 2.57607267304365 | 0.156794425087108 | PIK3CB//BMPR2//SULF1//GJA1//TNF//AIMP1//VEGFB//FZD3//TGFBR2//ZNF268//ADCYAP1//REG3G//CRKL//AGTR2//EDN1//EREG//CNOT6L//DLL1//CNOT7//HOXA3//IFNG//IGF1//IL2//AR//NTF3//POU3F2//PRKAA1//SPHK2//PTGFR//CNOT6//SOX4//BTC//FOSL1//CDK6//FRZB//TES//MTBP//IRF1//MDM4//MYOG//BTG1//TSC1//DLG5//RAPGEF2//ST18//C5AR1//ARX//EMX2//WNT7A//CASP3//GPR183//IL5//PTPN22//FZD6//CEBPB//PPP3CB//IFNA1//NCK1//CFDP1//EGLN3//EPS15//DUSP22//YES1//LRP6//HPRT1//IMPDH1//ESR1//MYB//OGN//PPARG//STRAP//RUNX3//JARID2//ZFPM2//GATA2//CCL8//PHF14//CMC4//TACC2//ZMYND11//APPL1//IRF2//MET//USP28//GMNC//EVI5//BHLHE41//BLZF1//CDC14A//CDC27// |
| GO:0090276 | regulation of peptide hormone secretion | Biological process | 15 | 203 | 574 | 17653 | 2.27248931532243 | 0.00269138800040929 | 0.10056274611474 | 2.57002368823034 | 0.0261324041811847 | RAPGEF4//IFNG//NEUROD1//PPP3CB//RFX3//SNAP25//TNF//GJA1//SOX4//SYBU//INHBB//ADCYAP1//EFNA5//NRXN1//AIMP1// |
| GO:0030902 | hindbrain development | Biological process | 12 | 145 | 574 | 17653 | 2.54518803316112 | 0.00278215487066844 | 0.103383040606212 | 2.5556186983538 | 0.0209059233449477 | GATA2//C5AR1//POU4F1//EN1//PTF1A//NEUROD1//LRP6//DLL1//AGTR2//RFX4//MTPN//WNT7A// |
| GO:0021987 | cerebral cortex development | Biological process | 10 | 109 | 574 | 17653 | 2.82150049547678 | 0.00291444069516832 | 0.107195370297609 | 2.53544477754584 | 0.0174216027874564 | EMX2//POU3F2//ARX//SLIT2//CNTNAP2//TACC2//LRP6//BNIP3//TSC1//LRP8// |
| GO:0045595 | regulation of cell differentiation | Biological process | 78 | 1761 | 574 | 17653 | 1.36220313529492 | 0.00292406954201898 | 0.107195370297609 | 2.53401230293307 | 0.13588850174216 | FZD3//CLASP2//TGFBR2//STRAP//LDLRAD4//FRZB//GATA2//PPARG//MYOG//BHLHE41//SNAP25//STMN2//ADCYAP1//NPTN//NCK1//RAPGEF2//PTPRG//SPOCK1//TSC1//DPYSL2//DLL1//IFNG//WNT7A//MAF//SOD1//IL2//CBFB//IRF1//CDK6//ANP32B//ZNF268//AR//NEUROD1//BTC//ZFPM2//TNF//CEBPB//BTG1//REG3G//MYB//SOCS5//IL5//NFE2//NTF3//DDX6//VWC2//NEUROG1//SMOC1//NPNT//GJA1//IGF1//BMPR2//ACVR1//CCR1//POU4F1//ULK2//EFNA5//FMR1//HECW2//NOS1//POU3F2//SLIT2//EREG//MED28//EDN1//CPEB3//DLG5//LRP8//AKAP6//LDLR//IL1RAP//CRKL//YTHDF2//NLN//PLAA//RFX3//GPR183//RUNX3// |
| GO:0044237 | cellular metabolic process | Biological process | 388 | 10957 | 574 | 17653 | 1.08904717490831 | 0.00293296255963805 | 0.107195370297609 | 2.53269348091525 | 0.67595818815331 | UBXN2B//STX12//ATG16L1//ULK2//RAD9B//IGF1//NFIA//ATR//WRNIP1//RBBP8//RRM2//DTD1//GADD45A//DIRAS3//NPAT//MTRR//TSHZ1//ZBTB18//CEBPB//ZNF268//TCERG1//STRAP//KLF12//ARX//EDN1//CC2D1B//EN1//ESR1//CPEB3//MYT1L//RCOR1//ZFPM2//GATA2//CNOT7//HOXC8//HSBP1//IFNG//IRF2//AR//JARID2//ZNF568//MAF//MDM4//MYB//NR4A2//KLF3//POU4F1//PPARG//DUSP22//ZBTB4//TNF//ZNF217//LRP8//BHLHE41//TBL1XR1//ARID5B//BHLHE40//RUNX3//PHF14//CSF2RA//SPRED1//EREG//RASGEF1A//IL2//IL5//MAP3K1//MET//MAP3K7CL//CCL2//BTC//LAMTOR3//RAPGEF2//DCP2//CRKL//NTF3//PIK3CB//PRKAA1//SOD1//C5AR1//UBE4B//RNF145//ZNRF2//RNF180//RNF125//UBE2W//HECW2//SKP1//THOP1//UBE2G1//LONRF3//CBFB//TNKS//CBL//UBE3B//LONRF1//CNOT6L//PAN3//CNOT6//FMR1//MBNL1//SMU1//RBM25//SRSF2//POLR2K//RBM41//SNRPN//BNIP3//NOB1//SPO11//SYCP1//TEX11//PTGES3//NABP1//NOS1//SECISBP2L//MGST1//ADH4//CHST7//CHST1//ST8SIA3//ACSL1//CAMLG//EPS15//LRP6//PPP1R15B//SLIT2//EFNA5//NPTN//ELK3//TFEC//FOXJ3//FOXF2//PTF1A//HIVEP2//MACC1//IRF1//MEOX2//MYBL1//NEUROD1//NHLH2//OTX2//POU3F2//RFX4//BMPR2//SOX4//BTF3//CARF//FOSL1//KLF7//NMI//SOCS5//RNF38//KLHL3//SPOPL//DCAF8//TRIM8//KBTBD8//SLC35D1//MDH1//NLN//COX8C//COX7A2//GLRX//GPD1//GPD2//GSTA2//GSTM3//DPYSL2//HPRT1//AK4//CMPK1//IMPDH1//TET2//DCTPP1//GMNC//ESCO2//EPC2//USP28//UNG//KDM2A//CASP3//BAHD1//ARID4B//ZMYND11//KLF17//NCOA7//ZNF782//ZNF800//ZFP30//HBP1//ATAD2//MDFIC//HOXA3//HOXB6//HOXD1//ZNF680//NEUROG1//NFE2//COMMD2//VGLL1//BRWD1//TRERF1//SLC2A4RG//ZNF630//ZBTB26//MIER1//RFX3//ATXN1//DMRTA1//SMARCD2//ZNF3//ZNF131//ZNF134//ZNF208//ZNF227//ZNF655//MED28//TSC22D4//PCGF5//LCOR//ST18//NRBF2//MLLT6//PAK6//BTG1//RHOXF2B//SNIP1//RPS6KA5//MED6//EMX2//BOLA3//NR3C2//RFX7//RHOXF2//BLZF1//ELAVL4//RTCA//SCAF8//CPEB1//QKI//CPEB2//MRPL35//METTL17//EIF3B//MTPN//DDX6//TSC1//AIMP1//YES1//IMMP1L//CDK6//FASTK//PDIK1L//CDK19//PPP3CB//CCL8//TGFBR2//ACVR1//FLRT1//NCK1//DBNDD2//PTPN22//MTM1//CPPED1//HS3ST5//FUT9//FUT2//ST8SIA5//MAN1A1//CCDC126//VEGFB//PIGP//PIGA//SLC6A14//SLC6A6//GLUD2//LGSN//SERINC3//PRKAR2B//MSMO1//TNFAIP8L3//PLAA//DDHD2//LCLAT1//PLA2G12A//CDS1//ABHD3//SLC44A1//MTMR9//SPHK2//ARSK//NEU1//CA1//GGT6//GPHN//NAT1//RAB12//PGC//DRAM1//ANP32B//EGLN3//DLL1//G3BP2//DGKE//ACBD5//GLT6D1//RRAGD//NPNT//CALM2//INHBB//BMP3//LDLR//DLG2//PLEKHF2//A2ML1//WFDC13//SPOCK1//NDST3//PRR16//INTS7//VPS37A//DYNLL2//CHMP3//EI24//SNX5//PIGS//GNPTG//DGKH//AGTR2//WNT7A//RASD1//BTAF1//OTUD3//WDR20//NFATC2IP//VKORC1L1//STK33//OGN//EGLN1//ELOVL7//DCUN1D4//MSRB3//SULF1//DSEL//IDS//VWC2//CDC27//RAP2C//CCT6A//PHACTR2//AKAP6//IFNA1//PTPRG//PTP4A1//CDC14A//WDFY3//RDH14//MYOG//ITM2A//TDRD5//ARL6IP1//CD2AP//FZD6//YTHDF2//MID1IP1//CCNY//ADCYAP1//ARMCX3//ENPP4//GRIK2//TMEM86A//EFR3A//SULT6B1//SAMSN1//SH3D19//IL1RAP//MYD88//ARRDC3//PMEPA1//LDLRAD4//CCR1//GPR183//UBA5//CALCOCO2//SNAP25//TMED10//RAB3GAP1//GJA1// |
| GO:0009952 | anterior/posterior pattern specification | Biological process | 15 | 205 | 574 | 17653 | 2.25031868785587 | 0.00295488912712792 | 0.107195370297609 | 2.52945881000435 | 0.0261324041811847 | DLL1//MEOX2//NEUROG1//TDRD5//EN1//LRP6//OTX2//TSHZ1//CRKL//EMX2//HOXA3//HOXB6//HOXC8//NEUROD1//BMPR2// |
| GO:0061138 | morphogenesis of a branching epithelium | Biological process | 14 | 185 | 574 | 17653 | 2.3273566249176 | 0.00296400033204981 | 0.107195370297609 | 2.52812175203974 | 0.024390243902439 | EDN1//TGFBR2//ACVR1//NPNT//MET//SLIT2//DLG5//LRP6//AR//ESR1//SULF1//BTBD7//TNF//AGTR2// |
| GO:0007049 | cell cycle | Biological process | 83 | 1898 | 574 | 17653 | 1.34489541530972 | 0.00303397101474 | 0.109142265812163 | 2.51798857259299 | 0.144599303135889 | RAD9B//INTS7//ATR//USP28//GADD45A//DIRAS3//CDK6//RBBP8//ACVR1//NPAT//RRM2//CCNY//PRKAR2B//SKP1//MYB//MYBL1//CLIP1//SRSF2//KIF4B//SPO11//SYCP1//TEX11//PAK6//IRF1//TSC1//CNOT6L//CNOT7//MDM4//CNOT6//SOX4//HBP1//MTBP//IFNG//PRKAA1//RRAGD//LAMTOR3//CLASP2//TNKS//DYNLL2//DYNC1LI2//CENPO//CDC27//NABP1//CDC14A//HEPACAM2//SYCE1//SYCP3//EREG//ZNF268//CDK19//MAP3K1//CHMP3//HECW2//NEUROG1//KIF13A//CALM2//CASP3//ADCYAP1//RUNX3//LRP6//FOSL1//EDN1//IGF1//BTC//BTG1//TNF//FZD3//NEUROD1//MYOG//POU4F1//MZT1//YTHDF2//ZNF655//CETN1//ZMYND11//ESCO2//CD2AP//APPL1//TET2//GMNC//PTP4A1//EVI5//LMLN// |
| GO:0006796 | phosphate-containing compound metabolic process | Biological process | 132 | 3255 | 574 | 17653 | 1.24718123283932 | 0.00305527565720221 | 0.109327138992902 | 2.51494960015104 | 0.229965156794425 | GADD45A//DIRAS3//CSF2RA//SPRED1//EREG//RASGEF1A//IL2//IL5//MAP3K1//MET//MAP3K7CL//CCL2//BTC//TNF//LAMTOR3//RAPGEF2//CRKL//IGF1//NTF3//PIK3CB//PRKAA1//SOD1//C5AR1//DUSP22//LRP6//PPP1R15B//SLIT2//EFNA5//FMR1//NPTN//IFNG//CBL//SOCS5//COX8C//GPD1//GPD2//HPRT1//AK4//CMPK1//IMPDH1//DCTPP1//CDK6//FASTK//PDIK1L//CDK19//PAN3//PPP3CB//CCL8//TGFBR2//RUNX3//ACVR1//RPS6KA5//FLRT1//NCK1//DBNDD2//PTPN22//MTM1//CPPED1//PIGP//PIGA//TNFAIP8L3//PLAA//DDHD2//LCLAT1//PLA2G12A//CDS1//ABHD3//SLC44A1//SERINC3//MTMR9//SPHK2//MDH1//GPHN//MDFIC//DGKE//RRM2//NPNT//CALM2//INHBB//BMP3//BMPR2//LDLR//DLG2//PLEKHF2//PIGS//DGKH//ATR//STK33//TNKS//PAK6//TBL1XR1//EGLN1//NOS1//RAP2C//PHACTR2//AGTR2//AKAP6//IFNA1//PRKAR2B//PTPRG//PTP4A1//CDC14A//YES1//PTGES3//AR//CNOT7//EDN1//NRBF2//UBXN2B//TSC1//UNG//CASP3//CCNY//MYOG//ADCYAP1//GRIK2//ZMYND11//WNT7A//ULK2//GNPTG//ESR1//EFR3A//SULT6B1//LRP8//SAMSN1//SKP1//STRAP//PMEPA1//LDLRAD4//CCR1//GPR183//ACSL1// |
| GO:0040011 | locomotion | Biological process | 81 | 1845 | 574 | 17653 | 1.35019121271352 | 0.00307402239973591 | 0.109419018365337 | 2.51229297221485 | 0.141114982578397 | ACVR1//DCX//GATA2//GJA1//LRP12//NR4A2//SPOCK1//FZD3//RAPGEF2//SLIT2//CCR1//GPR183//CCL2//CCL8//CD2AP//NCK1//PIK3CB//PLP2//CCL28//C5AR1//FOSL1//AIMP1//ARX//DOK1//DPYSL2//EFNA5//OTX2//ENAH//KLF7//RPS6KA5//NRXN1//EDN1//BMPR2//WNT7A//STRAP//PTPRG//CLASP2//IFNG//TGFBR2//ARID5B//C5ORF30//IGF1//RASGEF1A//PAK6//BTG1//YES1//POU4F1//POU3F2//EMX2//AKAP4//ZNF268//NTF3//FAM110C//PTP4A1//SULF1//RAP2C//LDLRAD4//DLG5//PPP3CB//AGTR2//PPARG//APCDD1//PROCR//TREM1//CD84//TNF//MET//VEGFB//RFX3//SPRED1//MEOX2//MMRN2//ARRDC3//CHMP3//TRIM8//CDK6//EREG//MEMO1//BTC//GCSAML//PLAA// |
| GO:0050767 | regulation of neurogenesis | Biological process | 38 | 732 | 574 | 17653 | 1.59653757544601 | 0.00313604936846037 | 0.111042418214123 | 2.50361710916325 | 0.0662020905923345 | FZD3//SNAP25//STMN2//ADCYAP1//NPTN//NCK1//RAPGEF2//PTPRG//SPOCK1//TSC1//DPYSL2//WNT7A//DLL1//NTF3//DDX6//GATA2//VWC2//NEUROD1//NEUROG1//ULK2//EFNA5//PPARG//FMR1//HECW2//BMPR2//POU3F2//SLIT2//MYB//CPEB3//IL2//DLG5//LRP8//LDLR//IL1RAP//PLAA//GPR183//NOS1//POU4F1// |
| GO:0051246 | regulation of protein metabolic process | Biological process | 124 | 3033 | 574 | 17653 | 1.25734918222434 | 0.00317831515297845 | 0.111952840518715 | 2.49780304154856 | 0.21602787456446 | GADD45A//DIRAS3//TNF//CRKL//MAP3K1//MAP3K7CL//LAMTOR3//IGF1//NTF3//PIK3CB//PRKAA1//SOD1//C5AR1//SPRED1//DUSP22//SECISBP2L//IL2//LRP6//PPP1R15B//SLIT2//EFNA5//FMR1//NPTN//IFNG//EREG//CBL//SOCS5//MTPN//DDX6//TSC1//KBTBD8//EIF3B//FLRT1//NCK1//DBNDD2//ANP32B//EGLN3//PPARG//CASP3//MDFIC//SNIP1//CALM2//INHBB//BMP3//BMPR2//ACVR1//A2ML1//WFDC13//SPOCK1//CPEB3//PRR16//SOX4//CNOT7//SPOPL//JARID2//CDK6//RAP2C//PAK6//TGFBR2//EDN1//WNT7A//MTM1//RNF180//RNF125//LONRF3//LONRF1//PHACTR2//AGTR2//AKAP6//RPS6KA5//IFNA1//PRKAR2B//GATA2//NOS1//CNOT6//MDM4//ITM2A//ARL6IP1//AR//MET//PTPN22//UBXN2B//IRF1//NMI//CEBPB//GJA1//HECW2//CCNY//ADCYAP1//RAPGEF2//GRIK2//ZMYND11//IL5//YES1//LRP8//SAMSN1//SH3D19//LDLR//DCUN1D4//ARRDC3//MYB//STRAP//PMEPA1//LDLRAD4//POLR2K//CNOT6L//YTHDF2//QKI//CCR1//GPR183//NPNT//TNFAIP8L3//CCL2//CCL8//RFX4//ACSL1//PLAA//CPEB2//TMED10//NCOA7//RAB3GAP1//ESR1//CPEB1//ST18// |
| GO:0030166 | proteoglycan biosynthetic process | Biological process | 7 | 60 | 574 | 17653 | 3.5880081300813 | 0.00321699499011152 | 0.112728171596498 | 2.49254961543269 | 0.0121951219512195 | NDST3//HS3ST5//SLC35D1//CHST7//DSEL//IGF1//BMPR2// |
| GO:0044271 | cellular nitrogen compound biosynthetic process | Biological process | 204 | 5342 | 574 | 17653 | 1.17444561994425 | 0.0032718401220655 | 0.114059045080046 | 2.48520792621133 | 0.355400696864112 | NPAT//RRM2//TSHZ1//ZBTB18//CEBPB//ZNF268//TCERG1//STRAP//KLF12//ARX//EDN1//CC2D1B//EN1//ESR1//CPEB3//MYT1L//RCOR1//ZFPM2//GATA2//CNOT7//HOXC8//HSBP1//IFNG//IRF2//AR//JARID2//ZNF568//MAF//MDM4//MYB//NFIA//NR4A2//KLF3//POU4F1//PPARG//DUSP22//ZBTB4//RBBP8//TNF//ZNF217//LRP8//BHLHE41//TBL1XR1//ARID5B//BHLHE40//RUNX3//PHF14//SYCP1//WRNIP1//SECISBP2L//ST8SIA3//ELK3//TFEC//FOXJ3//FOXF2//PTF1A//HIVEP2//MACC1//IRF1//MEOX2//MYBL1//NEUROD1//NHLH2//OTX2//POLR2K//POU3F2//RFX4//BMPR2//SOX4//BTF3//CARF//FOSL1//KLF7//CBFB//NMI//SRSF2//SLC35D1//HPRT1//IMPDH1//CMPK1//DCTPP1//BAHD1//ZMYND11//KLF17//NCOA7//ZNF782//ZNF800//ZFP30//KDM2A//CNOT6L//EPC2//HBP1//ATAD2//MDFIC//HOXA3//HOXB6//HOXD1//ZNF680//NEUROG1//NFE2//COMMD2//VGLL1//ARID4B//BRWD1//PRKAA1//TRERF1//SLC2A4RG//ZNF630//CNOT6//ZBTB26//MIER1//RFX3//ATXN1//DMRTA1//SMARCD2//ZNF3//ZNF131//ZNF134//ZNF208//ZNF227//ZNF655//MED28//TSC22D4//PCGF5//LCOR//ST18//NRBF2//MLLT6//PAK6//BTG1//RHOXF2B//SNIP1//CBL//RPS6KA5//MED6//EMX2//BOLA3//NR3C2//RFX7//RHOXF2//BLZF1//CPEB2//MRPL35//CPEB1//METTL17//EIF3B//MTPN//DDX6//TSC1//KBTBD8//AIMP1//GGT6//NOS1//PTGES3//G3BP2//AK4//EREG//FMR1//PRR16//AGTR2//IGF1//IL5//LRP6//PPP3CB//WNT7A//ACVR1//RASD1//BTAF1//BMP3//SULF1//VWC2//CALM2//EGLN1//TNKS//DCP2//ATR//CCT6A//NCK1//MYOG//C5AR1//INTS7//NABP1//FZD6//UNG//GPD1//ADCYAP1//DLL1//IL2//MET//ARMCX3//TET2//YES1//NFATC2IP//SPHK2//TRIM8//IL1RAP//MYD88//YTHDF2//QKI//EGLN3//RAP2C//PPP1R15B//GJA1//NPNT// |
| GO:0009790 | embryo development | Biological process | 47 | 959 | 574 | 17653 | 1.50725203736471 | 0.00333118930215552 | 0.115194598796682 | 2.47740068686885 | 0.0818815331010453 | WNT7A//EDN1//ZFPM2//GJA1//AR//ZNF568//MBNL1//BTF3//TGFBR2//FOSL1//ACVR1//LRP6//BMPR2//DLL1//MEOX2//RBBP8//SOX4//TSC1//FZD3//FZD6//CEBPB//GATA2//NEUROG1//RAI2//CLASP2//KBTBD8//TDRD5//EN1//LRIG1//HSBP1//PIK3CB//OTX2//TSHZ1//NEUROD1//FOXF2//TNF//YTHDF2//HOXA3//HOXB6//SLC35D1//SULF1//HOXD1//SOD1//EGLN1//FRZB//IGF1//DLG2// |
| GO:0010605 | negative regulation of macromolecule metabolic process | Biological process | 117 | 2842 | 574 | 17653 | 1.26610118996535 | 0.00337980498403017 | 0.115194598796682 | 2.47110835794808 | 0.20383275261324 | TSHZ1//ZBTB18//CEBPB//ZNF268//TCERG1//STRAP//KLF12//ARX//EDN1//CC2D1B//EN1//ESR1//CPEB3//MYT1L//RCOR1//ZFPM2//GATA2//CNOT7//HOXC8//HSBP1//IFNG//IRF2//AR//JARID2//ZNF568//MAF//MDM4//MYB//NFIA//NR4A2//KLF3//POU4F1//PPARG//DUSP22//ZBTB4//RBBP8//TNF//ZNF217//LRP8//BHLHE41//TBL1XR1//ARID5B//BHLHE40//RUNX3//PHF14//DCP2//SPRED1//CNOT6L//PAN3//CNOT6//IL2//LRP6//PPP1R15B//SLIT2//CBL//SOCS5//BAHD1//GADD45A//FLRT1//NCK1//DBNDD2//ATR//SNIP1//CCR1//PTPN22//GJA1//LDLR//POU3F2//CALM2//A2ML1//WFDC13//SPOCK1//ELK3//EREG//FOXF2//MDFIC//IRF1//RASD1//RFX3//ATXN1//TSC22D4//BTAF1//RPS6KA5//FMR1//TSC1//SPOPL//SOX4//CDK6//ATAD2//MIER1//MTM1//PHACTR2//ITM2A//ARL6IP1//YTHDF2//DDX6//NMI//CASP3//ZMYND11//INHBB//NTF3//SAMSN1//PMEPA1//LDLRAD4//POLR2K//QKI//MYD88//PLAA//CPEB2//NCOA7//TFEC//NPAT//SRSF2//TNKS//BMPR2//PRKAR2B//CPEB1// |
| GO:0006139 | nucleobase-containing compound metabolic process | Biological process | 225 | 5967 | 574 | 17653 | 1.15966649323895 | 0.00338386368223204 | 0.115194598796682 | 2.47058714068232 | 0.39198606271777 | RAD9B//IGF1//NFIA//ATR//WRNIP1//RBBP8//RRM2//DTD1//NPAT//TSHZ1//ZBTB18//CEBPB//ZNF268//TCERG1//STRAP//KLF12//ARX//EDN1//CC2D1B//EN1//ESR1//CPEB3//MYT1L//RCOR1//ZFPM2//GATA2//CNOT7//HOXC8//HSBP1//IFNG//IRF2//AR//JARID2//ZNF568//MAF//MDM4//MYB//NR4A2//KLF3//POU4F1//PPARG//DUSP22//ZBTB4//TNF//ZNF217//LRP8//BHLHE41//TBL1XR1//ARID5B//BHLHE40//RUNX3//PHF14//DCP2//CNOT6L//PAN3//CNOT6//FMR1//MBNL1//SMU1//RBM25//SRSF2//POLR2K//RBM41//SNRPN//NOB1//SPO11//SYCP1//TEX11//PTGES3//NABP1//ELK3//TFEC//FOXJ3//FOXF2//PTF1A//HIVEP2//MACC1//IRF1//MEOX2//MYBL1//NEUROD1//NHLH2//OTX2//POU3F2//RFX4//BMPR2//SOX4//BTF3//CARF//FOSL1//KLF7//CBFB//NMI//SLC35D1//COX8C//GPD1//GPD2//HPRT1//AK4//CMPK1//IMPDH1//DCTPP1//GMNC//ESCO2//GADD45A//EPC2//UBE2W//USP28//UNG//KDM2A//MTRR//CASP3//BAHD1//ARID4B//DIRAS3//ZMYND11//KLF17//NCOA7//ZNF782//ZNF800//ZFP30//HBP1//ATAD2//MDFIC//HOXA3//HOXB6//HOXD1//ZNF680//NEUROG1//NFE2//COMMD2//VGLL1//BRWD1//PRKAA1//TRERF1//SLC2A4RG//ZNF630//ZBTB26//MIER1//RFX3//ATXN1//DMRTA1//SMARCD2//ZNF3//ZNF131//ZNF134//ZNF208//ZNF227//ZNF655//MED28//TSC22D4//PCGF5//LCOR//ST18//NRBF2//MLLT6//PAK6//BTG1//RHOXF2B//SNIP1//CBL//RPS6KA5//MED6//EMX2//BOLA3//NR3C2//RFX7//RHOXF2//BLZF1//ELAVL4//RTCA//SCAF8//CPEB1//QKI//AIMP1//MDH1//G3BP2//EREG//DDX6//GLRX//INTS7//AGTR2//IL5//LRP6//NOS1//PPP3CB//WNT7A//ACVR1//RASD1//BTAF1//BMP3//SULF1//VWC2//CALM2//EGLN1//TNKS//CCT6A//GPHN//MYOG//C5AR1//TDRD5//FZD6//FASTK//YTHDF2//ADCYAP1//DLL1//IL2//MET//NCK1//ARMCX3//TET2//YES1//NFATC2IP//DLG2//ENPP4//SULT6B1//TRIM8//MTPN//IL1RAP//MYD88//EGLN3//RAP2C//GJA1//NPNT//DPYSL2// |
| GO:0002683 | negative regulation of immune system process | Biological process | 25 | 426 | 574 | 17653 | 1.80483306342118 | 0.00338759416525823 | 0.115194598796682 | 2.47010862382716 | 0.0435540069686411 | PPP3CB//SLIT2//DUSP22//TNF//SAMSN1//ADCYAP1//CASP3//CD84//RNF125//CEBPB//DLG5//IRF1//SOCS5//CDK6//DLL1//GATA2//NMI//IL2//PTPN22//PPARG//CNOT7//TSC22D4//CCL28//LDLR//CCL2// |
| GO:0000290 | deadenylation-dependent decapping of nuclear-transcribed mRNA | Biological process | 3 | 10 | 574 | 17653 | 9.22630662020906 | 0.00345906980944755 | 0.115194598796682 | 2.46104067315421 | 0.00522648083623693 | DCP2//PAN3//CNOT7// |
| GO:0003183 | mitral valve morphogenesis | Biological process | 3 | 10 | 574 | 17653 | 9.22630662020906 | 0.00345906980944755 | 0.115194598796682 | 2.46104067315421 | 0.00522648083623693 | BMPR2//SOX4//ACVR1// |
| GO:0098598 | learned vocalization behavior or vocal learning | Biological process | 3 | 10 | 574 | 17653 | 9.22630662020906 | 0.00345906980944755 | 0.115194598796682 | 2.46104067315421 | 0.00522648083623693 | CNTNAP2//NRXN1//NEUROG1// |
| GO:0030154 | cell differentiation | Biological process | 162 | 4124 | 574 | 17653 | 1.20810028489643 | 0.00347483646417018 | 0.115194598796682 | 2.45906562974429 | 0.282229965156794 | EREG//YTHDF2//ZFPM2//TGFBR2//QKI//GJA1//RDH14//BMP3//CBFB//AGFG1//GATA2//DLL1//ACVR1//DCX//LRP12//NR4A2//SPOCK1//FZD3//RAPGEF2//FOXF2//DLG5//MET//WNT7A//RUNX3//SULF1//BMPR2//ESR1//ESCO2//GPR183//ITM2A//SOX4//RFX3//CDK6//NEUROD1//AR//FRMD6//TDRD5//SPO11//STRBP//OGN//POU4F1//SLIT2//ARX//DOK1//DPYSL2//EFNA5//OTX2//PIK3CB//ENAH//KLF7//RPS6KA5//NRXN1//NOS1//ADAM12//CNTNAP2//GSTM3//CLASP2//STRAP//LDLRAD4//FRZB//PPARG//MYOG//BHLHE41//SNAP25//STMN2//ADCYAP1//NPTN//NCK1//PTPRG//TSC1//POU3F2//LRP6//KBTBD8//EDN1//IGF1//FLRT1//MTPN//HPRT1//KCNJ10//TET2//CEBPB//EMX2//MYT1L//HOXC8//HOXD1//NEUROG1//CASP3//IFNA1//PTPN22//IL2//PPP3CB//TMOD1//TNF//GSTA2//IFNG//UBE4B//DBNDD2//PSD//DSC2//KRT27//KRTAP4-11//SOD1//MAF//MGST1//SYCP1//SYCP3//PTF1A//HSBP1//IRF1//APCDD1//CCL2//CD8A//INHBB//ARID5B//MBNL1//ANP32B//ZNF268//BTC//BTG1//REG3G//MYB//SOCS5//IL5//NFE2//NTF3//DDX6//VWC2//SMOC1//NPNT//CCR1//PAQR5//EN1//FMR1//ULK2//BNIP3//NYAP1//HECW2//JARID2//TBL1XR1//MED28//SPRED1//CPEB3//LRP8//AGTR2//AKAP6//LDLR//RAP2C//ARID4B//IL1RAP//CRKL//NLN//PLAA//ELK3//FOXJ3//RAI14//MYBL1//NHLH2//YES1//ZNF3//SPATA9//ZIC5//ADAM18// |
| GO:0001504 | neurotransmitter uptake | Biological process | 5 | 32 | 574 | 17653 | 4.80536803135888 | 0.0034757706743173 | 0.115194598796682 | 2.45894888532004 | 0.00871080139372822 | ARL6IP1//NOS1//KCNJ10//SLC1A1//SNAP25// |
| GO:0021549 | cerebellum development | Biological process | 9 | 94 | 574 | 17653 | 2.94456594261991 | 0.00349177772487356 | 0.115194598796682 | 2.45695340985 | 0.0156794425087108 | LRP6//DLL1//AGTR2//RFX4//MTPN//WNT7A//EN1//PTF1A//NEUROD1// |
| GO:0030901 | midbrain development | Biological process | 9 | 94 | 574 | 17653 | 2.94456594261991 | 0.00349177772487356 | 0.115194598796682 | 2.45695340985 | 0.0156794425087108 | CALM2//FZD3//FZD6//LRP6//NR4A2//EN1//OTX2//RFX4//DLG5// |
| GO:0060393 | regulation of pathway-restricted SMAD protein phosphorylation | Biological process | 7 | 61 | 574 | 17653 | 3.52918832467013 | 0.00353442405881603 | 0.116035484998897 | 2.45168134522109 | 0.0121951219512195 | INHBB//BMP3//BMPR2//ACVR1//STRAP//PMEPA1//LDLRAD4// |
| GO:0061061 | muscle structure development | Biological process | 34 | 640 | 574 | 17653 | 1.63382513066202 | 0.00355665989309802 | 0.116201405106386 | 2.44895766239724 | 0.0592334494773519 | ZFPM2//UBE4B//POU4F1//IGF1//ZBTB18//MEOX2//MYOG//NOS1//ADAM12//BHLHE41//MTPN//TMOD1//QKI//MBNL1//DLL1//TNF//BTG1//GJA1//MTM1//NEUROG1//PRKAA1//ARID5B//ACVR1//EREG//MED28//TSC1//EGLN1//JARID2//TGFBR2//EDN1//AGTR2//AKAP6//NLN//NPNT// |
| GO:0051338 | regulation of transferase activity | Biological process | 48 | 988 | 574 | 17653 | 1.4941387239205 | 0.00357799680719967 | 0.11633650195717 | 2.4463600513081 | 0.0836236933797909 | GADD45A//DIRAS3//TNF//CRKL//MAP3K1//MAP3K7CL//LAMTOR3//IGF1//NTF3//PIK3CB//PRKAA1//SOD1//C5AR1//SPRED1//DUSP22//EREG//CBL//SOCS5//FLRT1//LRP6//NCK1//DBNDD2//EFNA5//MDFIC//CDK6//PAK6//TGFBR2//MET//PRKAR2B//EDN1//PTPN22//NRBF2//TNFAIP8L3//CASP3//CCNY//TSC1//ADCYAP1//RAPGEF2//DCUN1D4//ARRDC3//PTGES3//TNKS//PPARG//RAP2C//LRP8//ACSL1//IFNG//CALM2// |
| GO:0071156 | regulation of cell cycle arrest | Biological process | 10 | 113 | 574 | 17653 | 2.72162437174309 | 0.00378397233374808 | 0.1224449994887 | 2.42205204757269 | 0.0174216027874564 | GADD45A//CNOT6L//CNOT7//MDM4//CNOT6//SOX4//ZNF268//MYOG//POU4F1//NEUROD1// |
| GO:0051179 | localization | Biological process | 242 | 6497 | 574 | 17653 | 1.1455370181574 | 0.00412192372159245 | 0.13199325389049 | 2.38490004933458 | 0.421602787456446 | IFNG//TNF//SNAP25//ACVR1//DCX//GATA2//GJA1//LRP12//NR4A2//SPOCK1//FZD3//RAPGEF2//ADCYAP1//EDN1//AGTR2//NOS1//ARL6IP1//SLIT2//FMR1//NTF3//STIM2//CCR1//GPR183//CCL2//CCL8//MMRN1//IGF1//NHLRC2//SOD1//VEGFB//PHACTR2//SLC6A14//SLC6A6//RTN3//ARAP2//APPL1//AGFG1//HEATR5A//STX6//SYT6//STX12//CLASP2//ZFAND6//SRSF2//TSC1//PAN3//AKAP6//MTX3//IMMP1L//SLC17A5//GLRB//PLP2//TMEM63B//SLC1A1//KCNA4//KCNJ3//KCNJ10//KCNS2//SLC10A2//SLC4A4//TRPC3//CLCN6//SLC30A5//SV2C//SLC25A53//MICU3//MID1IP1//TMED10//RAB12//SNX5//ARFIP1//RAB30//GRIK2//RAB9B//RAB6B//KIF13A//SNX2//EVI5//RAB28//USP6NL//DCTN6//DYNLL2//DYNC1LI2//DDHD2//IER3IP1//KIF4B//KLC1//RP2//STEAP2//EPS15//DENND1B//DPYSL2//LDLR//LRP8//TGFBR2//ARHGAP12//NEUROD1//CD2AP//NCK1//G3BP2//WNT7A//NRXN1//RAPGEF4//PPP3CB//RFX3//MTM1//SLC35D1//BMPR2//STRAP//PTPRG//C5AR1//ARID5B//C5ORF30//CALM2//PPARG//VPS37A//CHMP3//ATG16L1//ZMAT3//BTF3//PLEKHF2//TNKS//SLC41A1//MMGT1//CA1//SLC2A5//SERINC3//SLC44A1//TNFAIP8L3//ARHGAP1//GNPTG//RASGEF1A//PIK3CB//PAK6//BTG1//YES1//PEX5L//INHBB//SYBU//POU4F1//POU3F2//ARX//EMX2//AKAP4//ZNF268//FAM110C//PTP4A1//SULF1//RAP2C//LDLRAD4//DLG5//SOX4//SLC30A7//SYCP1//MZT1//CLCN5//HTR3D//MTBP//SPO11//FRMD6//ARMCX3//RRAGD//LAMTOR3//KCNK10//CACNA2D3//MPP4//MDFIC//C16ORF70//BLZF1//DLG2//PXK//CBL//CD84//IQGAP2//ENPP4//OSTF1//IMPDH1//MGST1//NEU1//CPPED1//KCMF1//APCDD1//ACSL1//CLIP1//NPNT//DLL1//ANP32B//BNIP3//PLA2G12A//PROCR//TREM1//AIMP1//IL2//IL5//QKI//ATXN1//SKP1//GJB2//BCL2A1//CCL28//PRKAA1//EFNA5//RAB3GAP1//ATR//KLHL3//ESCO2//ESR1//CNTNAP2//GPHN//IL1RAP//EFR3A//LRP6//SPRED1//MEOX2//MMRN2//MIEF2//PRKAR2B//FAM155A//DSC2//PTPN22//TRIM8//COX7A2//COX8C//TMCO3//NPTN//AR//ARL5A//PLAA//SNIP1//CCT6A//DCP2//SESTD1//NEUROG1//CDK6//EREG//MEMO1//BTC//GCSAML//HECW2//GLRX//MYB//MET// |
| GO:0006875 | cellular metal ion homeostasis | Biological process | 30 | 550 | 574 | 17653 | 1.67751029458347 | 0.00413357676949065 | 0.13199325389049 | 2.38367399217034 | 0.0522648083623693 | CCR1//GRIK2//PIK3CB//STIM2//CCL8//CALCB//EGLN1//SKP1//SOD1//SLC30A5//AGTR2//ADCYAP1//EDN1//ESR1//GATA2//GJA1//NPTN//IL2//LRP6//CCL28//PTGFR//C5AR1//NOS1//TRPC3//CALM2//AKAP6//SLC41A1//SLC30A7//BNIP3//FAM155A// |
| GO:0035265 | organ growth | Biological process | 13 | 172 | 574 | 17653 | 2.32445709423872 | 0.00415711416215798 | 0.13199325389049 | 2.38120804878836 | 0.0226480836236934 | TGFBR2//SOD1//ARX//GJA1//JARID2//ZFPM2//ESR1//AR//AGTR2//EDN1//IGF1//AKAP6//DLL1// |
| GO:0050796 | regulation of insulin secretion | Biological process | 13 | 172 | 574 | 17653 | 2.32445709423872 | 0.00415711416215798 | 0.13199325389049 | 2.38120804878836 | 0.0226480836236934 | GJA1//SOX4//PPP3CB//SYBU//INHBB//EFNA5//NRXN1//RAPGEF4//IFNG//NEUROD1//RFX3//SNAP25//TNF// |
| GO:1901701 | cellular response to oxygen-containing compound | Biological process | 50 | 1048 | 574 | 17653 | 1.46728794850653 | 0.00423520440153288 | 0.132586732372108 | 2.37312562469204 | 0.0871080139372822 | CHRM2//APPL1//SOD1//CEBPB//CPEB3//RRAGD//CPEB1//TNF//LAMTOR3//PTPN22//CCL2//CPEB2//INHBB//PPARG//PPP3CB//SYBU//AGTR2//NCK1//TSC1//IGF1//EFNA5//NRXN1//MYB//PRKAA1//BNIP3//CDK19//PLAA//MYOG//YES1//GPD1//AKAP6//RAPGEF2//SLC2A5//NEUROD1//SOX4//EDN1//NR4A2//GJB2//PRKAR2B//ESR1//POU4F1//AR//LRP6//LRP8//LDLR//MGST1//SLIT2//PTGFR//MET//KLF3// |
| GO:0051960 | regulation of nervous system development | Biological process | 41 | 820 | 574 | 17653 | 1.53771777003484 | 0.00423656815475805 | 0.132586732372108 | 2.37298580259423 | 0.0714285714285714 | FZD3//SNAP25//STMN2//ADCYAP1//NPTN//NCK1//RAPGEF2//PTPRG//SPOCK1//TSC1//RFX4//DPYSL2//WNT7A//DLL1//NTF3//DDX6//GATA2//VWC2//NEUROD1//NEUROG1//ULK2//EFNA5//PPARG//FMR1//HECW2//BMPR2//NOS1//POU4F1//POU3F2//SLIT2//FLRT1//IL1RAP//DLG5//NRXN1//MYB//CPEB3//IL2//LRP8//LDLR//PLAA//GPR183// |
| GO:0003181 | atrioventricular valve morphogenesis | Biological process | 4 | 21 | 574 | 17653 | 5.85797245727559 | 0.00426070704742582 | 0.132586732372108 | 2.37051832547103 | 0.00696864111498258 | BMPR2//SOX4//ACVR1//TGFBR2// |
| GO:0007130 | synaptonemal complex assembly | Biological process | 4 | 21 | 574 | 17653 | 5.85797245727559 | 0.00426070704742582 | 0.132586732372108 | 2.37051832547103 | 0.00696864111498258 | SYCP1//SPO11//TEX11//SYCE1// |
| GO:0072503 | cellular divalent inorganic cation homeostasis | Biological process | 26 | 457 | 574 | 17653 | 1.74970074489741 | 0.00427382931496667 | 0.132586732372108 | 2.36918282635733 | 0.0452961672473868 | CCR1//GRIK2//PIK3CB//STIM2//CCL8//CALCB//SLC30A5//ADCYAP1//EDN1//ESR1//GATA2//GJA1//NPTN//IL2//LRP6//CCL28//PTGFR//C5AR1//NOS1//TRPC3//CALM2//AKAP6//SLC41A1//SLC30A7//BNIP3//FAM155A// |
| GO:0048754 | branching morphogenesis of an epithelial tube | Biological process | 12 | 153 | 574 | 17653 | 2.41210630593701 | 0.00430696761050757 | 0.133004666437729 | 2.36582839423014 | 0.0209059233449477 | EDN1//TGFBR2//ACVR1//NPNT//DLG5//LRP6//AR//ESR1//TNF//AGTR2//MET//SLIT2// |
| GO:0045859 | regulation of protein kinase activity | Biological process | 40 | 797 | 574 | 17653 | 1.54350591722443 | 0.00441466133738436 | 0.135710702839684 | 2.35510260691008 | 0.0696864111498258 | GADD45A//DIRAS3//TNF//CRKL//MAP3K1//MAP3K7CL//LAMTOR3//IGF1//NTF3//PIK3CB//PRKAA1//SOD1//C5AR1//SPRED1//DUSP22//EREG//CBL//SOCS5//FLRT1//LRP6//NCK1//DBNDD2//EFNA5//MDFIC//CDK6//PAK6//TGFBR2//PRKAR2B//EDN1//PTPN22//CASP3//CCNY//ADCYAP1//RAPGEF2//RAP2C//LRP8//ACSL1//IFNG//CALM2//TSC1// |
| GO:0048869 | cellular developmental process | Biological process | 168 | 4323 | 574 | 17653 | 1.19517272896532 | 0.00446258528399645 | 0.136001219742163 | 2.35041347104443 | 0.292682926829268 | EREG//YTHDF2//ZFPM2//TGFBR2//QKI//GJA1//RDH14//BMP3//CBFB//AGFG1//GATA2//DLL1//ACVR1//DCX//LRP12//NR4A2//SPOCK1//FZD3//RAPGEF2//FOXF2//DLG5//MET//WNT7A//RUNX3//SULF1//BMPR2//ESR1//ESCO2//GPR183//ITM2A//SOX4//RFX3//CDK6//NEUROD1//AR//FRMD6//TDRD5//SPO11//STRBP//OGN//POU4F1//SLIT2//ARX//DOK1//DPYSL2//EFNA5//OTX2//PIK3CB//ENAH//KLF7//RPS6KA5//NRXN1//NOS1//ADAM12//SOD1//CNTNAP2//GSTM3//CFDP1//BRWD1//CCL2//CLASP2//STRAP//LDLRAD4//FRZB//PPARG//MYOG//BHLHE41//SNAP25//STMN2//ADCYAP1//NPTN//NCK1//PTPRG//TSC1//POU3F2//LRP6//KBTBD8//EDN1//IGF1//FLRT1//MTPN//HPRT1//KCNJ10//SH3D19//TET2//ZNF268//ELK3//FOXJ3//RAI14//INHBB//MYB//MYBL1//NHLH2//SMOC1//YES1//ZNF3//SPATA9//ZIC5//ADAM18//CEBPB//EMX2//MYT1L//HOXC8//HOXD1//NEUROG1//CASP3//IFNA1//PTPN22//IL2//PPP3CB//TMOD1//TNF//GSTA2//IFNG//UBE4B//DBNDD2//PSD//DSC2//KRT27//KRTAP4-11//MAF//MGST1//SYCP1//SYCP3//PTF1A//HSBP1//IRF1//APCDD1//CD8A//ARID5B//MBNL1//ANP32B//BTC//BTG1//REG3G//SOCS5//IL5//NFE2//NTF3//DDX6//VWC2//NPNT//CCR1//PAQR5//EN1//FMR1//ULK2//BNIP3//NYAP1//HECW2//JARID2//TBL1XR1//MED28//SPRED1//CPEB3//LRP8//AGTR2//AKAP6//LDLR//MTM1//SNX2//ATR//RAP2C//ARID4B//IL1RAP//CRKL//NLN//PLAA// |
| GO:0032502 | developmental process | Biological process | 234 | 6267 | 574 | 17653 | 1.14831963679002 | 0.00446433103397313 | 0.136001219742163 | 2.35024360956834 | 0.407665505226481 | WNT7A//HAPLN1//EN1//FRZB//IGF1//BMP3//SOX4//ELK3//EREG//HOXA3//MEOX2//CCL2//VEGFB//MMRN2//AIMP1//CEBPB//ADCYAP1//SPO11//INHBB//DMRTA1//SOD1//ESR1//YTHDF2//CRKL//SPHK2//TGFBR2//RAPGEF2//EDN1//ACVR1//ZFPM2//QKI//GJA1//RDH14//CBFB//SMOC1//GATA2//NPNT//SLIT2//AGFG1//AR//ZNF568//MBNL1//BTF3//FOSL1//LRP6//BMPR2//DLL1//FZD3//DCX//LRP12//NR4A2//SPOCK1//TMED10//SULF1//TSC1//ARID5B//RBBP8//FOXF2//DLG5//FZD6//MET//AK4//JARID2//PPARG//APCDD1//ANP32B//PPP3CB//ARHGAP12//RUNX3//ESCO2//GPR183//ITM2A//UBE4B//POU4F1//RFX3//CDK6//NEUROD1//FRMD6//TSHZ1//CFDP1//WDR47//WDFY3//LCLAT1//HIVEP2//SHISA2//NFE2//KLF3//ATR//PAQR5//STRBP//BTBD7//TRERF1//DUSP22//ZNF3//ZNF217//PTP4A1//EVI5//SPATA9//ADAM18//TDRD5//FOXJ3//DLG2//NEUROG1//BHLHE41//PTF1A//FUT9//CHRM2//DPYSL2//EFNA5//MYT1L//FMR1//GLRB//NTF3//POU3F2//ZIC5//OGN//ARX//DOK1//OTX2//PIK3CB//ENAH//KLF7//RPS6KA5//NRXN1//PCDHB10//NHLH2//HPCAL4//AGTR2//RAB3GAP1//CNTNAP2//PTPRG//ATXN1//CASP3//ZBTB18//MYOG//NOS1//ADAM12//GJB2//GSTM3//BRWD1//RNF38//TEX11//CBL//RAI2//EMX2//HOXB6//HOXC8//CLASP2//BNIP3//STRAP//LDLRAD4//SNAP25//STMN2//NPTN//NCK1//KBTBD8//MTPN//FLRT1//UNG//SPRED1//DDX6//MED28//TRIM8//RFX4//C5AR1//LRP8//HPRT1//CALM2//TACC2//KCNJ10//SH3D19//TET2//ZNF268//RAI14//MYB//MYBL1//YES1//HOXD1//IFNA1//PTPN22//IL2//TMOD1//TNF//HSD11B1//CCR1//GSTA2//IFNG//KRT27//DBNDD2//PSD//DSC2//KRTAP4-11//MAF//LRIG1//MGST1//POLR2K//SYCP1//SYCP3//GPD2//TBL1XR1//HSBP1//SBF2//IRF1//ARRDC3//CD8A//BTC//BTG1//REG3G//SOCS5//IL5//VWC2//EGLN1//PHF14//MTM1//PRKAA1//ULK2//SLC35D1//NYAP1//HECW2//IL1RAP//NFIA//SYBU//CPEB3//AKAP6//LDLR//KLHL3//SNX2//MIEF2//DDHD2//RAP2C//ARID4B//NLN//PLAA//RBM41// |
| GO:0019221 | cytokine-mediated signaling pathway | Biological process | 40 | 798 | 574 | 17653 | 1.54157169928305 | 0.00450855076741456 | 0.136732416322981 | 2.34596303569642 | 0.0696864111498258 | CBFB//TNF//ST18//ACSL1//GSTA2//IFNG//SOD1//CA1//IL2//BRWD1//IL1RAP//IRF1//IRF2//TRIM8//NMI//PPARG//IFNA1//MYD88//XAF1//CNOT7//CCR1//CCL2//CCL8//SLIT2//EDN1//CBL//SKP1//RPS6KA5//CRKL//CSF2RA//EREG//FLRT1//IL5//PIK3CB//PLP2//IL20RA//IL36G//LRP8//CASP3//SOCS5// |
| GO:0009792 | embryo development ending in birth or egg hatching | Biological process | 31 | 578 | 574 | 17653 | 1.64945504744222 | 0.00458223704483336 | 0.138135293486492 | 2.33892244797066 | 0.054006968641115 | EDN1//ZFPM2//GJA1//AR//ZNF568//MBNL1//BTF3//TGFBR2//FOSL1//ACVR1//DLL1//MEOX2//RBBP8//SOX4//LRP6//TSC1//FZD3//FZD6//CEBPB//GATA2//KBTBD8//EN1//HOXA3//HOXB6//SLC35D1//SULF1//HOXD1//EGLN1//BMPR2//IGF1//RAI2// |
| GO:0003174 | mitral valve development | Biological process | 3 | 11 | 574 | 17653 | 8.38755147291733 | 0.00464214430413216 | 0.138135293486492 | 2.33328136333848 | 0.00522648083623693 | BMPR2//SOX4//ACVR1// |
| GO:0060394 | negative regulation of pathway-restricted SMAD protein phosphorylation | Biological process | 3 | 11 | 574 | 17653 | 8.38755147291733 | 0.00464214430413216 | 0.138135293486492 | 2.33328136333848 | 0.00522648083623693 | STRAP//PMEPA1//LDLRAD4// |
| GO:0098712 | L-glutamate import across plasma membrane | Biological process | 3 | 11 | 574 | 17653 | 8.38755147291733 | 0.00464214430413216 | 0.138135293486492 | 2.33328136333848 | 0.00522648083623693 | ARL6IP1//KCNJ10//SLC1A1// |
| GO:0043065 | positive regulation of apoptotic process | Biological process | 33 | 627 | 574 | 17653 | 1.6186502842472 | 0.00465693433608165 | 0.138135293486492 | 2.331899885683 | 0.0574912891986063 | ANP32B//EGLN3//PPARG//TNF//CASP3//BNIP3//PIK3CB//IFNG//GRIK2//MYB//DYNLL2//SOD1//SERINC3//NCK1//BTG1//RAPGEF2//INHBB//AGTR2//IL20RA//ST18//ZNF268//GADD45A//CDK19//FRZB//NEUROD1//ARHGEF4//POU4F1//BCL2A1//ZMAT3//SOS2//SOX4//FOSL1//SLIT2// |
| GO:0006793 | phosphorus metabolic process | Biological process | 134 | 3352 | 574 | 17653 | 1.22944022186557 | 0.00473114295915658 | 0.139723667392035 | 2.32503392884114 | 0.233449477351916 | GADD45A//DIRAS3//CSF2RA//SPRED1//EREG//RASGEF1A//IL2//IL5//MAP3K1//MET//MAP3K7CL//CCL2//BTC//TNF//LAMTOR3//RAPGEF2//CRKL//IGF1//NTF3//PIK3CB//PRKAA1//SOD1//C5AR1//DUSP22//LRP6//PPP1R15B//SLIT2//EFNA5//FMR1//NPTN//IFNG//CBL//SOCS5//SLC35D1//COX8C//GPD1//GPD2//HPRT1//AK4//CMPK1//IMPDH1//DCTPP1//CDK6//FASTK//PDIK1L//CDK19//PAN3//PPP3CB//CCL8//TGFBR2//RUNX3//ACVR1//RPS6KA5//FLRT1//NCK1//DBNDD2//PTPN22//MTM1//CPPED1//PIGP//PIGA//TNFAIP8L3//PLAA//DDHD2//LCLAT1//PLA2G12A//CDS1//ABHD3//SLC44A1//SERINC3//MTMR9//SPHK2//MDH1//GPHN//MDFIC//DGKE//RRM2//NPNT//CALM2//INHBB//BMP3//BMPR2//LDLR//DLG2//PLEKHF2//PIGS//DGKH//ATR//STK33//TNKS//PAK6//TBL1XR1//EGLN1//NOS1//RAP2C//PHACTR2//AGTR2//AKAP6//IFNA1//PRKAR2B//PTPRG//PTP4A1//CDC14A//ACSL1//ELOVL7//YES1//PTGES3//AR//CNOT7//EDN1//NRBF2//UBXN2B//TSC1//UNG//CASP3//CCNY//MYOG//ADCYAP1//GRIK2//ZMYND11//WNT7A//ULK2//GNPTG//ESR1//EFR3A//SULT6B1//LRP8//SAMSN1//SKP1//STRAP//PMEPA1//LDLRAD4//CCR1//GPR183// |
| GO:0071345 | cellular response to cytokine stimulus | Biological process | 51 | 1081 | 574 | 17653 | 1.45094553694315 | 0.0048316915836788 | 0.142072739914868 | 2.31590079536815 | 0.0888501742160279 | CBFB//TNF//CCR1//CRKL//CSF2RA//EREG//FLRT1//IFNA1//IL1RAP//IL2//IL5//PIK3CB//PLP2//IL20RA//IL36G//CCL2//LRP8//CASP3//CBL//SOCS5//ST18//ACSL1//IRF1//GSTA2//IFNG//SOD1//CA1//BRWD1//IRF2//TRIM8//NMI//PPARG//MYD88//XAF1//CNOT7//CCL8//SLIT2//EDN1//SKP1//RPS6KA5//RAB12//CEBPB//NPNT//GPD1//MYOG//ZFAND6//JARID2//PIGA//ARID5B//POU4F1//AKAP6// |
| GO:0061157 | mRNA destabilization | Biological process | 4 | 22 | 574 | 17653 | 5.59170098194488 | 0.0050755241372372 | 0.148596405801451 | 2.29451910254656 | 0.00696864111498258 | CPEB3//QKI//CNOT6L//YTHDF2// |
| GO:0034097 | response to cytokine | Biological process | 54 | 1164 | 574 | 17653 | 1.42674844642408 | 0.00529264588938131 | 0.154285190301232 | 2.27632716201331 | 0.0940766550522648 | CBFB//TNF//CCR1//CRKL//CSF2RA//EREG//FLRT1//IFNA1//IL1RAP//IL2//IL5//PIK3CB//PLP2//IL20RA//IL36G//CCL2//LRP8//CASP3//CBL//SOCS5//ST18//ACSL1//CALCOCO2//XAF1//IRF1//GSTA2//IFNG//SOD1//CA1//BRWD1//IRF2//TRIM8//NMI//PPARG//MYD88//CNOT7//CCL8//SLIT2//EDN1//SKP1//RPS6KA5//POU4F1//AKAP6//RAB12//CEBPB//NPNT//GPD1//MYOG//ZFAND6//JARID2//PIGA//ARID5B//PPP3CB//FOSL1// |
| GO:0043009 | chordate embryonic development | Biological process | 30 | 560 | 574 | 17653 | 1.64755475360876 | 0.00532454998598961 | 0.154549062468874 | 2.27371709152494 | 0.0522648083623693 | EDN1//ZFPM2//GJA1//AR//ZNF568//MBNL1//BTF3//TGFBR2//FOSL1//ACVR1//DLL1//MEOX2//RBBP8//SOX4//LRP6//TSC1//FZD3//FZD6//CEBPB//GATA2//KBTBD8//EN1//HOXA3//HOXB6//SLC35D1//SULF1//HOXD1//EGLN1//BMPR2//IGF1// |
| GO:0043068 | positive regulation of programmed cell death | Biological process | 33 | 633 | 574 | 17653 | 1.60330762752448 | 0.00536192972846779 | 0.154968934844563 | 2.27067888202391 | 0.0574912891986063 | ANP32B//EGLN3//PPARG//TNF//CASP3//BNIP3//PIK3CB//IFNG//ZNF268//GADD45A//CDK19//FRZB//NEUROD1//ARHGEF4//POU4F1//BCL2A1//ZMAT3//SOD1//SOS2//SOX4//FOSL1//SLIT2//GRIK2//MYB//DYNLL2//SERINC3//NCK1//BTG1//RAPGEF2//INHBB//AGTR2//IL20RA//ST18// |
| GO:0048589 | developmental growth | Biological process | 32 | 609 | 574 | 17653 | 1.61599240200706 | 0.00540744134797042 | 0.155311918644515 | 2.26700818245585 | 0.0557491289198606 | ESR1//EREG//TGFBR2//IGF1//WNT7A//DPYSL2//EN1//GPD2//AR//TBL1XR1//ARID5B//DLL1//SOD1//POU3F2//MTPN//GJA1//MYOG//ARX//MTM1//EFNA5//ULK2//PPP3CB//BMPR2//SLIT2//JARID2//ZFPM2//AGTR2//EDN1//AKAP6//LRP6//PLAA//FLRT1// |
| GO:0001763 | morphogenesis of a branching structure | Biological process | 14 | 198 | 574 | 17653 | 2.17455038186745 | 0.00541972686679072 | 0.155311918644515 | 2.26602259966725 | 0.024390243902439 | EDN1//TGFBR2//ACVR1//NPNT//MET//SLIT2//DLG5//LRP6//AR//ESR1//SULF1//BTBD7//TNF//AGTR2// |
| GO:0051239 | regulation of multicellular organismal process | Biological process | 120 | 2969 | 574 | 17653 | 1.24301874303928 | 0.00547708098746304 | 0.156067018533309 | 2.26145083728041 | 0.209059233449477 | RPS6KA5//EREG//SOD1//TNF//AGTR2//CALM2//FZD3//TGFBR2//EDN1//GJA1//BMPR2//CHRM2//PROCR//HS3ST5//NPNT//SLC1A1//CLASP2//SULF1//C5AR1//WNT7A//SLIT2//MTPN//IGF1//JARID2//STRAP//PTPRG//IFNG//LDLRAD4//GATA2//SNAP25//STMN2//ADCYAP1//NPTN//NCK1//RAPGEF2//SPOCK1//TSC1//LDLR//MYOG//DLL1//SPRED1//PPARG//INHBB//RFX4//ENPP4//LRP6//ACVR1//CCR1//DPYSL2//ARRDC3//MAF//RNF125//PTPN22//IRF1//MYD88//POLR2K//CD84//SOCS5//IL2//IL1RAP//CEBPB//CD2AP//POU3F2//OTX2//NR4A2//IL20RA//NMI//CBFB//BTG1//REG3G//MYB//CDK6//IL5//NFE2//NTF3//DDX6//VWC2//NEUROD1//NEUROG1//SMOC1//POU4F1//EGLN1//VEGFB//ADAM12//UNG//ARX//MTM1//PRKAA1//ULK2//EFNA5//FMR1//HECW2//NOS1//FLRT1//DLG5//NRXN1//ZFPM2//FZD6//AR//ESR1//BTBD7//CPEB3//LRP8//FRZB//AKAP6//PTF1A//CACNA2D3//DSC2//KCNJ3//MEOX2//MMRN2//C5ORF30//YTHDF2//NLN//TRPC3//PLAA//RFX3//GPR183//PHF14//MET// |
| GO:1902117 | positive regulation of organelle assembly | Biological process | 7 | 66 | 574 | 17653 | 3.26182557280118 | 0.00549222984044472 | 0.156067018533309 | 2.26025129657217 | 0.0121951219512195 | CNOT6L//PAN3//CNOT6//EDN1//IL5//TNF//RAB3GAP1// |
| GO:0048598 | embryonic morphogenesis | Biological process | 31 | 586 | 574 | 17653 | 1.62693688979796 | 0.00557298432195381 | 0.15769913376307 | 2.25391217870919 | 0.054006968641115 | LRP6//ACVR1//BMPR2//SOX4//TSC1//FZD3//FZD6//GJA1//DLL1//TGFBR2//CLASP2//MBNL1//LRIG1//EN1//WNT7A//HSBP1//GATA2//NEUROG1//TSHZ1//EDN1//NEUROD1//FOXF2//HOXA3//HOXB6//SOD1//AR//ZNF568//SULF1//OTX2//FRZB//YTHDF2// |
| GO:0035303 | regulation of dephosphorylation | Biological process | 14 | 199 | 574 | 17653 | 2.16362299301385 | 0.00566168599929958 | 0.159541593388596 | 2.24705422055074 | 0.024390243902439 | TNF//NPNT//MTMR9//SPRED1//DLG2//PLEKHF2//PHACTR2//AGTR2//CALM2//PPP1R15B//AKAP6//UBXN2B//TSC1//IFNG// |
| GO:1901698 | response to nitrogen compound | Biological process | 50 | 1065 | 574 | 17653 | 1.44386645073694 | 0.00575127846040043 | 0.161393760280864 | 2.24023560460056 | 0.0871080139372822 | HPRT1//NR4A2//SOD1//CHRM2//APPL1//SNIP1//MGST1//CEBPB//CPEB3//RRAGD//CPEB1//TNF//LAMTOR3//UBE4B//PPARG//PRKAA1//C5AR1//IGF1//TSC1//CPEB2//INHBB//TRPC3//AGTR2//EDN1//GLRB//CASP3//TMED10//IFNA1//REG3G//EREG//GJA1//LRP6//BTG1//NCK1//FOSL1//GSTM3//PTPN22//GPD1//AKAP6//RAPGEF2//GJB2//PRKAR2B//LDLR//SOCS5//SLIT2//EGLN1//FMR1//MYD88//KLF3//ESR1// |
| GO:0006468 | protein phosphorylation | Biological process | 83 | 1946 | 574 | 17653 | 1.31172224987556 | 0.00584465302375958 | 0.163336315701182 | 2.23324126625397 | 0.144599303135889 | GADD45A//DIRAS3//CSF2RA//SPRED1//EREG//RASGEF1A//IL2//IL5//MAP3K1//MET//MAP3K7CL//CCL2//BTC//TNF//LAMTOR3//RAPGEF2//CRKL//IGF1//NTF3//PIK3CB//PRKAA1//SOD1//C5AR1//DUSP22//LRP6//PPP1R15B//SLIT2//EFNA5//FMR1//NPTN//IFNG//CBL//SOCS5//FLRT1//NCK1//DBNDD2//TGFBR2//MDFIC//CALM2//INHBB//BMP3//BMPR2//ACVR1//RPS6KA5//ATR//STK33//TNKS//PAK6//CDK6//RAP2C//IFNA1//PRKAR2B//YES1//CNOT7//EDN1//AR//PTPN22//CASP3//CCNY//TSC1//ADCYAP1//GRIK2//ZMYND11//WNT7A//ULK2//LRP8//SAMSN1//SKP1//STRAP//PMEPA1//LDLRAD4//CCR1//GPR183//NPNT//TNFAIP8L3//CCL8//ACSL1//FASTK//PDIK1L//CDK19//PAN3//PPP3CB//RUNX3// |
| GO:0007169 | transmembrane receptor protein tyrosine kinase signaling pathway | Biological process | 36 | 711 | 574 | 17653 | 1.55718255193402 | 0.00588621947473052 | 0.163336993336141 | 2.23016354874552 | 0.0627177700348432 | EREG//CBL//SOCS5//EFNA5//EPS15//BTC//CAMLG//RPS6KA5//APPL1//SPRED1//FLRT1//POLR2K//MMRN2//RAPGEF2//SULF1//OTX2//SHISA2//IGF1//AR//DOK1//NPTN//NCK1//TSC1//ARID5B//PIK3CB//BMPR2//VEGFB//CASP3//MET//YES1//AGTR2//DLL1//PHF14//NTF3//PTPRG//CD8A// |
| GO:0071229 | cellular response to acid chemical | Biological process | 14 | 200 | 574 | 17653 | 2.15280487804878 | 0.00591225057374973 | 0.163336993336141 | 2.22824716789965 | 0.024390243902439 | CEBPB//CPEB3//RRAGD//CPEB1//TNF//LAMTOR3//MYB//MYOG//PPARG//YES1//PRKAA1//EDN1//LDLR//PTGFR// |
| GO:0040008 | regulation of growth | Biological process | 34 | 662 | 574 | 17653 | 1.57952882722612 | 0.00594019389872567 | 0.163336993336141 | 2.22619937862618 | 0.0592334494773519 | SPOCK1//BLZF1//MTPN//IL2//H3F3C//AKAP6//AGTR2//FRZB//GJA1//PPARG//BMPR2//BTG1//SLIT2//EI24//DPYSL2//TNF//IGF1//SOD1//POU3F2//TGFBR2//ARX//DLL1//MTM1//AR//ULK2//EFNA5//JARID2//ZFPM2//EDN1//PLAA//LRP12//IFNG//ZMAT3//SOCS5// |
| GO:0051726 | regulation of cell cycle | Biological process | 55 | 1197 | 574 | 17653 | 1.41310739100946 | 0.00594128350742137 | 0.163336993336141 | 2.22611972337302 | 0.0958188153310105 | RAD9B//INTS7//ATR//USP28//GADD45A//DIRAS3//CNOT6L//CNOT7//MDM4//CNOT6//SOX4//CDK6//HBP1//MTBP//IFNG//IRF1//PRKAA1//RRAGD//TSC1//LAMTOR3//CDC27//NABP1//CDC14A//ZNF268//CDK19//MAP3K1//PAK6//PRKAR2B//CHMP3//SKP1//HECW2//NEUROG1//RBBP8//KIF13A//CALM2//CASP3//CCNY//ADCYAP1//RUNX3//LRP6//FOSL1//EDN1//EREG//IGF1//BTC//BTG1//TNF//FZD3//RRM2//NEUROD1//MYOG//POU4F1//YTHDF2//TNKS//ZNF655// |
| GO:2000679 | positive regulation of transcription regulatory region DNA binding | Biological process | 4 | 23 | 574 | 17653 | 5.34858354794728 | 0.00598849464111635 | 0.163362463835717 | 2.22268233474261 | 0.00696864111498258 | IFNG//IGF1//NEUROD1//POU4F1// |
| GO:0021761 | limbic system development | Biological process | 9 | 102 | 574 | 17653 | 2.71361959417913 | 0.00599052063156261 | 0.163362463835717 | 2.22253543176944 | 0.0156794425087108 | LRP8//CDK6//EMX2//NEUROD1//TSC1//CASP3//RAB3GAP1//POU3F2//CNTNAP2// |
| GO:0034392 | negative regulation of smooth muscle cell apoptotic process | Biological process | 3 | 12 | 574 | 17653 | 7.68858885017422 | 0.0060413237350758 | 0.163429889681271 | 2.21886789120483 | 0.00522648083623693 | IGF1//EDN1//LRP6// |
| GO:0051938 | L-glutamate import | Biological process | 3 | 12 | 574 | 17653 | 7.68858885017422 | 0.0060413237350758 | 0.163429889681271 | 2.21886789120483 | 0.00522648083623693 | ARL6IP1//KCNJ10//SLC1A1// |
| GO:0030072 | peptide hormone secretion | Biological process | 16 | 243 | 574 | 17653 | 2.02497813337922 | 0.0060862112991985 | 0.163988235125416 | 2.21565297401483 | 0.0278745644599303 | RAPGEF4//IFNG//NEUROD1//PPP3CB//RFX3//SNAP25//TNF//ADCYAP1//GJA1//SOX4//SYBU//INHBB//EFNA5//NRXN1//AIMP1//EDN1// |
| GO:0043549 | regulation of kinase activity | Biological process | 43 | 889 | 574 | 17653 | 1.48755599800896 | 0.00614091228455599 | 0.164805515001794 | 2.21176710594064 | 0.0749128919860627 | GADD45A//DIRAS3//TNF//CRKL//MAP3K1//MAP3K7CL//LAMTOR3//IGF1//NTF3//PIK3CB//PRKAA1//SOD1//C5AR1//SPRED1//DUSP22//EREG//CBL//SOCS5//FLRT1//LRP6//NCK1//DBNDD2//EFNA5//MDFIC//CDK6//PAK6//TGFBR2//MET//PRKAR2B//EDN1//PTPN22//NRBF2//TNFAIP8L3//CASP3//CCNY//TSC1//ADCYAP1//RAPGEF2//RAP2C//LRP8//ACSL1//IFNG//CALM2// |
| GO:0019725 | cellular homeostasis | Biological process | 44 | 916 | 574 | 17653 | 1.47728344719505 | 0.00632048355076146 | 0.166972781339381 | 2.19924969459831 | 0.0766550522648084 | DNAJC16//GLRX//NHLRC2//NOS1//CCR1//GRIK2//PIK3CB//STIM2//CCL8//CALCB//EGLN1//SKP1//SOD1//SLC30A5//AGTR2//CLCN6//ADCYAP1//EDN1//ESR1//GATA2//GJA1//NPTN//IL2//LRP6//CCL28//PTGFR//C5AR1//TRPC3//CALM2//AKAP6//SLC41A1//PLAA//SLC30A7//PPP3CB//SYBU//MTM1//SLC4A4//BNIP3//EFNA5//NRXN1//NEUROD1//SOX4//FAM155A//CCL2// |
| GO:0010506 | regulation of autophagy | Biological process | 19 | 310 | 574 | 17653 | 1.88494436326852 | 0.00635179262654634 | 0.166972781339381 | 2.19710368920451 | 0.0331010452961672 | PTPN22//MET//MTMR9//IFNG//PIK3CB//PRKAA1//BNIP3//TRIM8//RAB12//TSC1//SNX5//RRAGD//CASP3//LAMTOR3//CALCOCO2//MTM1//RAB3GAP1//DRAM1//ULK2// |
| GO:0022037 | metencephalon development | Biological process | 9 | 103 | 574 | 17653 | 2.68727377287643 | 0.00638060283423952 | 0.166972781339381 | 2.19513828754598 | 0.0156794425087108 | EN1//PTF1A//NEUROD1//LRP6//DLL1//AGTR2//RFX4//MTPN//WNT7A// |
| GO:0050777 | negative regulation of immune response | Biological process | 11 | 141 | 574 | 17653 | 2.39927595324585 | 0.00638298682110525 | 0.166972781339381 | 2.19497605225313 | 0.0191637630662021 | PPP3CB//DUSP22//TNF//SAMSN1//ADCYAP1//CD84//SOCS5//NMI//PPARG//CNOT7//IL2// |
| GO:0006928 | movement of cell or subcellular component | Biological process | 88 | 2090 | 574 | 17653 | 1.29492022739776 | 0.00638906991073755 | 0.166972781339381 | 2.19456235968733 | 0.153310104529617 | ACVR1//DCX//GATA2//GJA1//LRP12//NR4A2//SPOCK1//FZD3//RAPGEF2//SLIT2//CCR1//GPR183//CCL2//CCL8//FRMD6//CD2AP//NCK1//DYNC1LI2//KIF4B//KLC1//KIF13A//ARX//DOK1//DPYSL2//EFNA5//OTX2//PIK3CB//ENAH//KLF7//RPS6KA5//NRXN1//FMR1//SOD1//EDN1//BMPR2//WNT7A//STRAP//PTPRG//CLASP2//IFNG//TGFBR2//C5AR1//ARID5B//C5ORF30//DYNLL2//IGF1//RASGEF1A//PAK6//BTG1//YES1//SYBU//POU4F1//POU3F2//EMX2//TMOD1//AKAP4//ZNF268//NTF3//FAM110C//PTP4A1//SULF1//RAP2C//LDLRAD4//DLG5//AGTR2//PPARG//APCDD1//PROCR//TREM1//CD84//AIMP1//TNF//RFX3//CCL28//VEGFB//SPRED1//MEOX2//MMRN2//DSC2//KCNJ3//CDK6//EREG//MEMO1//BTC//GCSAML//MET//PLAA//AKAP11// |
| GO:0048562 | embryonic organ morphogenesis | Biological process | 18 | 288 | 574 | 17653 | 1.92214721254355 | 0.00640195189083667 | 0.166972781339381 | 2.19368759380055 | 0.0313588850174216 | GJA1//DLL1//TGFBR2//ACVR1//LRIG1//GATA2//NEUROG1//FZD3//FZD6//TSHZ1//EDN1//FOXF2//LRP6//HOXA3//HOXB6//SOD1//FRZB//NEUROD1// |
| GO:0030073 | insulin secretion | Biological process | 14 | 202 | 574 | 17653 | 2.13148997826612 | 0.00644005795568943 | 0.166972781339381 | 2.19111022429813 | 0.024390243902439 | RAPGEF4//IFNG//NEUROD1//PPP3CB//RFX3//SNAP25//TNF//GJA1//SOX4//SYBU//INHBB//EFNA5//NRXN1//ADCYAP1// |
| GO:0003002 | regionalization | Biological process | 20 | 333 | 574 | 17653 | 1.84710843247429 | 0.00646225634435287 | 0.166972781339381 | 2.18961581841219 | 0.0348432055749129 | DLL1//MEOX2//NEUROG1//TSHZ1//CRKL//EMX2//HOXA3//HOXB6//HOXC8//LRP6//NEUROD1//BMPR2//EDN1//EN1//WNT7A//RFX4//TDRD5//AR//OTX2//ACVR1// |
| GO:0006807 | nitrogen compound metabolic process | Biological process | 367 | 10390 | 574 | 17653 | 1.08631842464444 | 0.00646383536734736 | 0.166972781339381 | 2.18950971349014 | 0.639372822299652 | RAD9B//IGF1//NFIA//ATR//WRNIP1//RBBP8//RRM2//DTD1//GADD45A//DIRAS3//NPAT//MTRR//TSHZ1//ZBTB18//CEBPB//ZNF268//TCERG1//STRAP//KLF12//ARX//EDN1//CC2D1B//EN1//ESR1//CPEB3//MYT1L//RCOR1//ZFPM2//GATA2//CNOT7//HOXC8//HSBP1//IFNG//IRF2//AR//JARID2//ZNF568//MAF//MDM4//MYB//NR4A2//KLF3//POU4F1//PPARG//DUSP22//ZBTB4//TNF//ZNF217//LRP8//BHLHE41//TBL1XR1//ARID5B//BHLHE40//RUNX3//PHF14//CSF2RA//SPRED1//EREG//RASGEF1A//IL2//IL5//MAP3K1//MET//MAP3K7CL//CCL2//BTC//LAMTOR3//RAPGEF2//DCP2//CRKL//NTF3//PIK3CB//PRKAA1//SOD1//C5AR1//UBE4B//RNF145//ZNRF2//RNF180//RNF125//UBE2W//HECW2//SKP1//THOP1//UBE2G1//LONRF3//CBFB//TNKS//CBL//UBE3B//LONRF1//CNOT6L//PAN3//CNOT6//FMR1//MBNL1//SMU1//RBM25//SRSF2//POLR2K//RBM41//SNRPN//NOB1//SPO11//SYCP1//TEX11//PTGES3//NABP1//SECISBP2L//ST8SIA3//LRP6//PPP1R15B//SLIT2//EFNA5//NPTN//ELK3//TFEC//FOXJ3//FOXF2//PTF1A//HIVEP2//MACC1//IRF1//MEOX2//MYBL1//NEUROD1//NHLH2//OTX2//POU3F2//RFX4//BMPR2//SOX4//BTF3//CARF//FOSL1//KLF7//NMI//SOCS5//RNF38//KLHL3//SPOPL//DCAF8//TRIM8//KBTBD8//HS3ST5//IDS//SLC35D1//COX8C//GPD1//GPD2//GSTA2//GSTM3//MGST1//DPYSL2//HPRT1//AK4//CMPK1//IMPDH1//TET2//DCTPP1//GMNC//ESCO2//EPC2//USP28//UNG//KDM2A//CASP3//BAHD1//ARID4B//ZMYND11//KLF17//NCOA7//ZNF782//ZNF800//ZFP30//HBP1//ATAD2//MDFIC//HOXA3//HOXB6//HOXD1//ZNF680//NEUROG1//NFE2//COMMD2//VGLL1//BRWD1//TRERF1//SLC2A4RG//ZNF630//ZBTB26//MIER1//RFX3//ATXN1//DMRTA1//SMARCD2//ZNF3//ZNF131//ZNF134//ZNF208//ZNF227//ZNF655//MED28//TSC22D4//PCGF5//LCOR//ST18//NRBF2//MLLT6//PAK6//BTG1//RHOXF2B//SNIP1//RPS6KA5//MED6//EMX2//BOLA3//NR3C2//RFX7//RHOXF2//BLZF1//ELAVL4//RTCA//SCAF8//CPEB1//QKI//CPEB2//MRPL35//METTL17//EIF3B//MTPN//DDX6//TSC1//AIMP1//YES1//IMMP1L//CDK6//FASTK//PDIK1L//CDK19//PPP3CB//CCL8//TGFBR2//ACVR1//FLRT1//NCK1//DBNDD2//PTPN22//MTM1//CPPED1//FUT9//FUT2//ST8SIA5//MAN1A1//CCDC126//VEGFB//PIGP//PIGA//GGT6//PRSS55//MEP1A//DESI2//PGC//NLN//NPEPL1//ADAM12//ADAM18//LMLN//SLC6A14//SLC6A6//NOS1//GLUD2//LGSN//SERINC3//ABHD3//SLC44A1//CDS1//SPHK2//ARSK//NEU1//MDH1//GPHN//ANP32B//EGLN3//VSIG4//DLL1//G3BP2//CALM2//INHBB//BMP3//LDLR//A2ML1//WFDC13//SPOCK1//NDST3//GLRX//PRR16//INTS7//PIGS//GNPTG//AGTR2//WNT7A//RASD1//BTAF1//OTUD3//WDR20//NFATC2IP//VKORC1L1//STK33//OGN//CHST1//EGLN1//DCUN1D4//MSRB3//ELOVL7//SULF1//DSEL//CHST7//VWC2//CDC27//RAP2C//CCT6A//PHACTR2//AKAP6//IFNA1//PRKAR2B//PTPRG//PTP4A1//CDC14A//BNIP3//PLA2G12A//IL1RAP//MYOG//ITM2A//TDRD5//ARL6IP1//UBXN2B//CD2AP//PLAA//VPS37A//FZD6//YTHDF2//RAB12//GJA1//CCNY//ADCYAP1//ARMCX3//DLG2//ENPP4//GRIK2//ULK2//SULT6B1//SAMSN1//SH3D19//MYD88//ARRDC3//PMEPA1//LDLRAD4//ATG16L1//CCR1//GPR183//NPNT//TNFAIP8L3//UBA5//ACSL1//TMED10//RAB3GAP1// |
| GO:0009247 | glycolipid biosynthetic process | Biological process | 7 | 68 | 574 | 17653 | 3.16588952654232 | 0.00646855962012683 | 0.166972781339381 | 2.18919241464387 | 0.0121951219512195 | ST8SIA3//PIGP//PIGA//ST8SIA5//PIGS//PRKAA1//GLT6D1// |
| GO:0021587 | cerebellum morphogenesis | Biological process | 5 | 37 | 574 | 17653 | 4.15599397306714 | 0.00658700750228711 | 0.16874216567412 | 2.18131184191752 | 0.00871080139372822 | DLL1//RFX4//MTPN//WNT7A//LRP6// |
| GO:0090503 | RNA phosphodiester bond hydrolysis, exonucleolytic | Biological process | 5 | 37 | 574 | 17653 | 4.15599397306714 | 0.00658700750228711 | 0.16874216567412 | 2.18131184191752 | 0.00871080139372822 | DCP2//CNOT6L//PAN3//CNOT7//CNOT6// |
| GO:0006836 | neurotransmitter transport | Biological process | 16 | 246 | 574 | 17653 | 2.00028327809411 | 0.00682265077186313 | 0.174119196868341 | 2.16604685819897 | 0.0278745644599303 | SNAP25//ADCYAP1//EDN1//ARL6IP1//SLC1A1//WNT7A//NRXN1//SLC44A1//FMR1//SYT6//NOS1//KCNJ10//RAB3GAP1//SLC6A14//SV2C//SLC6A6// |
| GO:0072507 | divalent inorganic cation homeostasis | Biological process | 26 | 475 | 574 | 17653 | 1.68339629561709 | 0.00698437588531735 | 0.174727125804458 | 2.15587239580216 | 0.0452961672473868 | CCR1//GRIK2//PIK3CB//STIM2//CCL8//CALCB//SLC30A5//ADCYAP1//EDN1//ESR1//GATA2//GJA1//NPTN//IL2//LRP6//CCL28//PTGFR//C5AR1//NOS1//TRPC3//CALM2//AKAP6//SLC41A1//SLC30A7//BNIP3//FAM155A// |
| GO:0055021 | regulation of cardiac muscle tissue growth | Biological process | 7 | 69 | 574 | 17653 | 3.12000706963591 | 0.00700251332529019 | 0.174727125804458 | 2.15474605607176 | 0.0121951219512195 | GJA1//JARID2//TGFBR2//ZFPM2//EDN1//IGF1//AKAP6// |
| GO:0003171 | atrioventricular valve development | Biological process | 4 | 24 | 574 | 17653 | 5.12572590011614 | 0.00700446561761129 | 0.174727125804458 | 2.15462499216625 | 0.00696864111498258 | TGFBR2//ACVR1//BMPR2//SOX4// |
| GO:0045672 | positive regulation of osteoclast differentiation | Biological process | 4 | 24 | 574 | 17653 | 5.12572590011614 | 0.00700446561761129 | 0.174727125804458 | 2.15462499216625 | 0.00696864111498258 | CCR1//IFNG//POU4F1//TNF// |
| GO:0050779 | RNA destabilization | Biological process | 4 | 24 | 574 | 17653 | 5.12572590011614 | 0.00700446561761129 | 0.174727125804458 | 2.15462499216625 | 0.00696864111498258 | CNOT6L//YTHDF2//CPEB3//QKI// |
| GO:0070193 | synaptonemal complex organization | Biological process | 4 | 24 | 574 | 17653 | 5.12572590011614 | 0.00700446561761129 | 0.174727125804458 | 2.15462499216625 | 0.00696864111498258 | SPO11//TEX11//SYCP1//SYCE1// |
| GO:0048568 | embryonic organ development | Biological process | 24 | 428 | 574 | 17653 | 1.72454329349702 | 0.0070273219309201 | 0.174727125804458 | 2.15321015040193 | 0.0418118466898955 | CEBPB//GATA2//GJA1//DLL1//TGFBR2//ACVR1//LRIG1//NEUROG1//FZD3//FZD6//TSHZ1//EDN1//NEUROD1//FOXF2//TNF//LRP6//HOXA3//HOXB6//SOD1//ZNF568//EGLN1//FRZB//EN1//ZFPM2// |
| GO:0051128 | regulation of cellular component organization | Biological process | 99 | 2403 | 574 | 17653 | 1.26703336856804 | 0.00707818764462807 | 0.17534719062498 | 2.15007792828422 | 0.172473867595819 | SPOCK1//BLZF1//FMR1//NTF3//STMN2//CLASP2//MID1IP1//CDC27//CDC14A//CFDP1//BRWD1//CCL2//SLIT2//CNOT6L//PAN3//CNOT6//BNIP3//CHMP3//SNAP25//ADCYAP1//NPTN//NCK1//RAPGEF2//PTPRG//TSC1//PMEPA1//LDLRAD4//SH3D19//EFNA5//HECW2//MTPN//IL2//H3F3C//AKAP6//AGTR2//FRZB//GJA1//PPARG//BMPR2//BTG1//EI24//DPYSL2//JARID2//MET//CLIP1//WNT7A//IFNG//TNF//EVI5//USP6NL//NEUROG1//ATAD2//MIER1//TNKS//DCP2//ATR//CCT6A//MIEF2//RPS6KA5//IQGAP2//ARFIP1//GATA2//NOS1//MTM1//AR//DLL1//LDLR//MTBP//EDN1//EREG//IGF1//BTC//ESR1//CD2AP//CBL//ULK2//POU3F2//MYB//TMOD1//FLRT1//IL1RAP//DLG5//NRXN1//FAM110C//CPEB3//LRP8//IL5//DDHD2//CRKL//DYNLL2//CALCOCO2//MYOG//RAB3GAP1//PLAA//PRKAA1//SNIP1//FOSL1//PIK3CB//CBFB// |
| GO:0009653 | anatomical structure morphogenesis | Biological process | 108 | 2656 | 574 | 17653 | 1.25055360816087 | 0.00718753329765772 | 0.176594289009636 | 2.14342013034329 | 0.18815331010453 | WNT7A//ELK3//EREG//HOXA3//MEOX2//CCL2//VEGFB//MMRN2//AIMP1//EDN1//TGFBR2//ACVR1//ZFPM2//QKI//NPNT//AGFG1//LRP6//BMPR2//FZD3//DLL1//SOX4//TSC1//FZD6//MET//PPP3CB//GJA1//ARHGAP12//SLIT2//UBE4B//POU4F1//AR//FRMD6//CRKL//GATA2//BHLHE41//OGN//ARX//DOK1//DPYSL2//EFNA5//OTX2//PIK3CB//ENAH//KLF7//RPS6KA5//NRXN1//NOS1//ADAM12//CFDP1//BRWD1//CLASP2//BNIP3//PTF1A//KBTBD8//SPRED1//SULF1//PPARG//NEUROG1//RFX4//MTPN//NR4A2//SH3D19//CASP3//TMOD1//MBNL1//DLG5//KRT27//LRIG1//EN1//AGTR2//HSBP1//FOXF2//TSHZ1//EGLN1//BTG1//C5AR1//NEUROD1//YTHDF2//ARID5B//ULK2//HOXB6//HOXC8//NYAP1//CNTNAP2//NTF3//HPRT1//FMR1//HECW2//POU3F2//RAPGEF2//FRZB//SOD1//RAB3GAP1//ESR1//TNF//ZNF568//BTBD7//LRP8//MTM1//KLHL3//CEBPB//SNX2//MIEF2//DDHD2//MYOG//PLAA//FLRT1//FOXJ3// |
| GO:0010508 | positive regulation of autophagy | Biological process | 9 | 105 | 574 | 17653 | 2.63608760577402 | 0.00721906712684799 | 0.176594289009636 | 2.14151891985945 | 0.0156794425087108 | RAB12//BNIP3//TSC1//CALCOCO2//RAB3GAP1//IFNG//PIK3CB//PRKAA1//TRIM8// |
| GO:0046620 | regulation of organ growth | Biological process | 9 | 105 | 574 | 17653 | 2.63608760577402 | 0.00721906712684799 | 0.176594289009636 | 2.14151891985945 | 0.0156794425087108 | ARX//GJA1//JARID2//TGFBR2//ZFPM2//EDN1//IGF1//AKAP6//SOD1// |
| GO:0048146 | positive regulation of fibroblast proliferation | Biological process | 6 | 53 | 574 | 17653 | 3.48162513970153 | 0.00723297620222818 | 0.176594289009636 | 2.14068296380624 | 0.0104529616724739 | MYB//CDK6//EREG//ESR1//IGF1//BTC// |
| GO:0048145 | regulation of fibroblast proliferation | Biological process | 8 | 87 | 574 | 17653 | 2.82798670351236 | 0.00732433515742192 | 0.177542934299801 | 2.13523179084651 | 0.0139372822299652 | CDK6//EREG//ESR1//IGF1//BTC//AGTR2//MYB//PPARG// |
| GO:0060021 | roof of mouth development | Biological process | 8 | 87 | 574 | 17653 | 2.82798670351236 | 0.00732433515742192 | 0.177542934299801 | 2.13523179084651 | 0.0139372822299652 | TSHZ1//ANP32B//FOXF2//LRP6//MEOX2//TGFBR2//WNT7A//ARID5B// |
| GO:0033993 | response to lipid | Biological process | 43 | 900 | 574 | 17653 | 1.46937475803329 | 0.00755522917766224 | 0.181722222050822 | 2.12175235738922 | 0.0749128919860627 | ESR1//AR//PMEPA1//PTPN22//CCL2//TNF//GJB2//PTGFR//RBBP8//WNT7A//CASP3//EDN1//GJA1//MGST1//NOS1//C5AR1//PPARG//FOSL1//UBA5//CBFB//CBL//ACSL1//NR3C2//NR4A2//PAQR5//TGFBR2//SLIT2//CEBPB//CDK19//PLAA//MYB//MYOG//YES1//PRKAA1//ADCYAP1//POU4F1//LRP6//LRP8//LDLR//FIBIN//AGTR2//EFNA5//GATA2// |
| GO:0045597 | positive regulation of cell differentiation | Biological process | 44 | 926 | 574 | 17653 | 1.46133006223614 | 0.00760655260119167 | 0.181722222050822 | 2.11881212713236 | 0.0766550522648084 | FZD3//TGFBR2//MYOG//STMN2//ADCYAP1//NPTN//NCK1//RAPGEF2//IFNG//IL2//CEBPB//FRZB//PPARG//BTG1//MYB//SOCS5//IL5//GATA2//VWC2//NEUROD1//NEUROG1//NPNT//GJA1//IGF1//BMPR2//ACVR1//CCR1//POU4F1//TNF//EFNA5//SLIT2//EDN1//CPEB3//FMR1//DLG5//LRP8//AKAP6//IL1RAP//CRKL//PLAA//RFX3//ZNF268//AR//BTC// |
| GO:0055065 | metal ion homeostasis | Biological process | 32 | 624 | 574 | 17653 | 1.57714643080497 | 0.0076392648796244 | 0.181722222050822 | 2.11694843122577 | 0.0557491289198606 | AGTR2//EDN1//CCR1//GRIK2//PIK3CB//STIM2//CCL8//CALCB//EGLN1//SKP1//SOD1//SLC30A5//ADCYAP1//ESR1//GATA2//GJA1//NPTN//IL2//LRP6//CCL28//PTGFR//C5AR1//NOS1//TRPC3//CALM2//AKAP6//SLC41A1//SLC30A7//BNIP3//STEAP2//KCNJ10//FAM155A// |
| GO:0031223 | auditory behavior | Biological process | 3 | 13 | 574 | 17653 | 7.09715893862235 | 0.00766599305447094 | 0.181722222050822 | 2.11543157856018 | 0.00522648083623693 | CNTNAP2//NRXN1//NEUROG1// |
| GO:0061051 | positive regulation of cell growth involved in cardiac muscle cell development | Biological process | 3 | 13 | 574 | 17653 | 7.09715893862235 | 0.00766599305447094 | 0.181722222050822 | 2.11543157856018 | 0.00522648083623693 | EDN1//IGF1//AKAP6// |
| GO:1904861 | excitatory synapse assembly | Biological process | 3 | 13 | 574 | 17653 | 7.09715893862235 | 0.00766599305447094 | 0.181722222050822 | 2.11543157856018 | 0.00522648083623693 | NRXN1//WNT7A//NPTN// |
| GO:0071363 | cellular response to growth factor stimulus | Biological process | 34 | 674 | 574 | 17653 | 1.55140665226067 | 0.0077290591112805 | 0.181722222050822 | 2.11187337123686 | 0.0592334494773519 | DUSP22//TGFBR2//CBL//ACVR1//SPRED1//FLRT1//POLR2K//BMP3//BMPR2//NPNT//STRAP//PMEPA1//LDLRAD4//SULF1//VWC2//MMRN2//YES1//NTF3//RAPGEF2//OTX2//SHISA2//CCL2//NPTN//CASP3//AGTR2//EDN1//WNT7A//SLIT2//DLL1//STMN2//DYNC1LI2//MYOG//NOS1//LRP8// |
| GO:0060562 | epithelial tube morphogenesis | Biological process | 19 | 316 | 574 | 17653 | 1.84915428042165 | 0.00773470530572373 | 0.181722222050822 | 2.11155622838169 | 0.0331010452961672 | EDN1//TGFBR2//ACVR1//NPNT//SOX4//LRP6//TSC1//FZD3//FZD6//GJA1//DLL1//MET//SLIT2//DLG5//AR//ESR1//TNF//KLHL3//AGTR2// |
| GO:0045664 | regulation of neuron differentiation | Biological process | 31 | 600 | 574 | 17653 | 1.588975029036 | 0.00773857754704076 | 0.181722222050822 | 2.11133886105406 | 0.054006968641115 | SNAP25//STMN2//ADCYAP1//NPTN//NCK1//RAPGEF2//PTPRG//SPOCK1//TSC1//DPYSL2//WNT7A//DLL1//DDX6//GATA2//VWC2//NEUROD1//NEUROG1//ULK2//EFNA5//FMR1//HECW2//BMPR2//POU3F2//SLIT2//CPEB3//IL2//DLG5//LRP8//IL1RAP//PLAA//NTF3// |
| GO:0006650 | glycerophospholipid metabolic process | Biological process | 21 | 362 | 574 | 17653 | 1.78409244037192 | 0.0078256473010252 | 0.182072257659866 | 2.1064797294829 | 0.0365853658536585 | PIGP//PIGA//DDHD2//LCLAT1//GPD1//PLA2G12A//CDS1//ABHD3//SLC44A1//SERINC3//MTM1//PIK3CB//MTMR9//LDLR//PIGS//DGKH//EREG//ESR1//EFR3A//MET//BTC// |
| GO:0048144 | fibroblast proliferation | Biological process | 8 | 88 | 574 | 17653 | 2.79585049097244 | 0.00783424914668356 | 0.182072257659866 | 2.10600262103052 | 0.0139372822299652 | CDK6//EREG//ESR1//IGF1//BTC//AGTR2//MYB//PPARG// |
| GO:0071158 | positive regulation of cell cycle arrest | Biological process | 8 | 88 | 574 | 17653 | 2.79585049097244 | 0.00783424914668356 | 0.182072257659866 | 2.10600262103052 | 0.0139372822299652 | GADD45A//CNOT6L//CNOT7//MDM4//CNOT6//SOX4//MYOG//POU4F1// |
| GO:0051241 | negative regulation of multicellular organismal process | Biological process | 53 | 1162 | 574 | 17653 | 1.40273738058256 | 0.00790201792199346 | 0.182331373823128 | 2.10226198966165 | 0.0923344947735192 | RPS6KA5//TNF//BMPR2//PROCR//HS3ST5//AGTR2//SLIT2//JARID2//STRAP//PTPRG//LDLRAD4//STMN2//SPOCK1//TSC1//SPRED1//SULF1//PPARG//INHBB//RFX4//EDN1//CCR1//ARRDC3//RNF125//CD84//DLL1//IFNG//SOCS5//PTPN22//CD2AP//NMI//IRF1//REG3G//CDK6//GATA2//DDX6//IL2//FZD3//ULK2//WNT7A//RAPGEF2//GJA1//TGFBR2//SOD1//ADCYAP1//IGF1//FRZB//LDLR//MEOX2//MMRN2//C5ORF30//NEUROG1//ZFPM2//PHF14// |
| GO:0001885 | endothelial cell development | Biological process | 6 | 54 | 574 | 17653 | 3.41715060007743 | 0.00791917121937085 | 0.182331373823128 | 2.10132026710846 | 0.0104529616724739 | MET//GSTM3//WNT7A//RAPGEF2//RAP2C//TNF// |
| GO:0040007 | growth | Biological process | 45 | 954 | 574 | 17653 | 1.4506771415423 | 0.00792627885612888 | 0.182331373823128 | 2.10093065272506 | 0.078397212543554 | ESR1//EREG//AR//SOCS5//SPOCK1//BLZF1//TGFBR2//IGF1//WNT7A//MTPN//IL2//H3F3C//AKAP6//AGTR2//FRZB//GJA1//PPARG//BMPR2//BTG1//SLIT2//EI24//DPYSL2//TNF//EN1//GPD2//TBL1XR1//ARID5B//DLL1//LRP12//IFNG//ZMAT3//SOD1//POU3F2//MYOG//ARX//MTM1//EFNA5//ULK2//PPP3CB//JARID2//ZFPM2//EDN1//LRP6//PLAA//FLRT1// |
| GO:0070848 | response to growth factor | Biological process | 35 | 701 | 574 | 17653 | 1.53552416408615 | 0.00807866118001505 | 0.185083796214751 | 2.09266060584772 | 0.0609756097560976 | DUSP22//TGFBR2//CBL//ACVR1//SPRED1//FLRT1//POLR2K//BMP3//BMPR2//NPNT//STRAP//PMEPA1//LDLRAD4//SULF1//VWC2//MMRN2//YES1//NTF3//RAPGEF2//OTX2//SHISA2//CCL2//NPTN//CASP3//AGTR2//MYOG//NOS1//LRP8//RUNX3//EDN1//WNT7A//SLIT2//DLL1//STMN2//DYNC1LI2// |
| GO:0007263 | nitric oxide mediated signal transduction | Biological process | 4 | 25 | 574 | 17653 | 4.9206968641115 | 0.00812803304388304 | 0.185083796214751 | 2.0900145394663 | 0.00696864111498258 | AGTR2//NEUROD1//NOS1//RASD1// |
| GO:0055094 | response to lipoprotein particle | Biological process | 4 | 25 | 574 | 17653 | 4.9206968641115 | 0.00812803304388304 | 0.185083796214751 | 2.0900145394663 | 0.00696864111498258 | LDLR//PPARG//SOCS5//MYD88// |
| GO:0061014 | positive regulation of mRNA catabolic process | Biological process | 5 | 39 | 574 | 17653 | 3.94286607701242 | 0.00825129501573708 | 0.186667911888099 | 2.08347788489839 | 0.00871080139372822 | CPEB3//CNOT7//CNOT6L//YTHDF2//QKI// |
| GO:0043583 | ear development | Biological process | 14 | 208 | 574 | 17653 | 2.07000469043152 | 0.0082528028470415 | 0.186667911888099 | 2.08339852971977 | 0.024390243902439 | LRIG1//GATA2//NEUROG1//FZD3//FZD6//TSHZ1//EDN1//DLL1//ANP32B//GJB2//MAF//NEUROD1//SOD1//FRZB// |
| GO:0032879 | regulation of localization | Biological process | 109 | 2700 | 574 | 17653 | 1.24156471802813 | 0.00854886567807388 | 0.192719928602712 | 2.0680915066092 | 0.189895470383275 | AGTR2//NOS1//ARL6IP1//FMR1//NTF3//CCL8//SLIT2//TNF//EDN1//ADCYAP1//RAPGEF4//IFNG//NEUROD1//PPP3CB//RFX3//SNAP25//SLC30A5//TRPC3//BMPR2//WNT7A//STRAP//PTPRG//CLASP2//TGFBR2//C5AR1//C5ORF30//CALM2//AKAP6//PPARG//IGF1//INHBB//SYT6//GJA1//NCK1//ZNF268//CCR1//FAM110C//PTP4A1//ACVR1//SULF1//RAP2C//LDLRAD4//DLG5//EVI5//USP6NL//SOX4//STIM2//STX6//SYCP1//KCNA4//KCNJ3//KCNJ10//KCNK10//CACNA2D3//SYBU//MDFIC//PXK//CD84//GATA2//DLL1//LDLR//APPL1//ANP32B//CD2AP//CBL//ARFIP1//IL2//IL5//PRKAA1//VEGFB//SLC30A7//EFNA5//NRXN1//RAB3GAP1//AIMP1//SPRED1//MEOX2//MMRN2//CCL2//MIEF2//DSC2//PTPN22//TRIM8//DYNLL2//KCNS2//NPTN//AR//CCL28//PLAA//SNIP1//CCT6A//DCP2//SESTD1//NEUROG1//CDK6//EREG//MEMO1//BTC//PIK3CB//GCSAML//GPR183//CHMP3//HECW2//GLRX//IL1RAP//MYB//MET//ARHGAP1//RAPGEF2// |
| GO:0044272 | sulfur compound biosynthetic process | Biological process | 13 | 188 | 574 | 17653 | 2.12663095855883 | 0.00863284005014946 | 0.19373038162022 | 2.06384630565215 | 0.0226480836236934 | GGT6//MTRR//OGN//CHST1//SLC35D1//CHST7//DSEL//NDST3//ACSL1//ELOVL7//GSTA2//GSTM3//MGST1// |
| GO:0060688 | regulation of morphogenesis of a branching structure | Biological process | 6 | 55 | 574 | 17653 | 3.35502058916693 | 0.00865097963171765 | 0.19373038162022 | 2.06293471050518 | 0.0104529616724739 | AR//SULF1//ESR1//BTBD7//TNF//AGTR2// |
| GO:0071900 | regulation of protein serine/threonine kinase activity | Biological process | 27 | 508 | 574 | 17653 | 1.6345818815331 | 0.00878216238276984 | 0.196019023744793 | 2.05639853702337 | 0.0470383275261324 | GADD45A//DIRAS3//TNF//CRKL//MAP3K1//MAP3K7CL//LAMTOR3//IGF1//NTF3//PIK3CB//PRKAA1//SOD1//C5AR1//SPRED1//DUSP22//MDFIC//CDK6//EDN1//PTPN22//CASP3//CCNY//LRP6//ACSL1//IFNG//CALM2//PRKAR2B//RAPGEF2// |
| GO:0043200 | response to amino acid | Biological process | 9 | 109 | 574 | 17653 | 2.5393504459291 | 0.00914610106076761 | 0.202311484920746 | 2.03876400414807 | 0.0156794425087108 | CEBPB//CPEB3//RRAGD//CPEB1//TNF//LAMTOR3//EDN1//GLRB//CASP3// |
| GO:0000289 | nuclear-transcribed mRNA poly(A) tail shortening | Biological process | 5 | 40 | 574 | 17653 | 3.84429442508711 | 0.00918373885415776 | 0.202311484920746 | 2.03698047422863 | 0.00871080139372822 | CPEB3//CNOT7//CNOT6L//PAN3//CNOT6// |
| GO:0010613 | positive regulation of cardiac muscle hypertrophy | Biological process | 5 | 40 | 574 | 17653 | 3.84429442508711 | 0.00918373885415776 | 0.202311484920746 | 2.03698047422863 | 0.00871080139372822 | EDN1//IGF1//AKAP6//TRPC3//MTPN// |
| GO:0014742 | positive regulation of muscle hypertrophy | Biological process | 5 | 40 | 574 | 17653 | 3.84429442508711 | 0.00918373885415776 | 0.202311484920746 | 2.03698047422863 | 0.00871080139372822 | MTPN//EDN1//IGF1//AKAP6//TRPC3// |
| GO:0006725 | cellular aromatic compound metabolic process | Biological process | 228 | 6178 | 574 | 17653 | 1.13499401608269 | 0.00936320829796783 | 0.204937057036335 | 2.02857531500529 | 0.397212543554007 | RAD9B//IGF1//NFIA//ATR//WRNIP1//RBBP8//RRM2//DTD1//NPAT//TSHZ1//ZBTB18//CEBPB//ZNF268//TCERG1//STRAP//KLF12//ARX//EDN1//CC2D1B//EN1//ESR1//CPEB3//MYT1L//RCOR1//ZFPM2//GATA2//CNOT7//HOXC8//HSBP1//IFNG//IRF2//AR//JARID2//ZNF568//MAF//MDM4//MYB//NR4A2//KLF3//POU4F1//PPARG//DUSP22//ZBTB4//TNF//ZNF217//LRP8//BHLHE41//TBL1XR1//ARID5B//BHLHE40//RUNX3//PHF14//DCP2//CNOT6L//PAN3//CNOT6//FMR1//MBNL1//SMU1//RBM25//SRSF2//POLR2K//RBM41//SNRPN//NOB1//SPO11//SYCP1//TEX11//PTGES3//NABP1//ELK3//TFEC//FOXJ3//FOXF2//PTF1A//HIVEP2//MACC1//IRF1//MEOX2//MYBL1//NEUROD1//NHLH2//OTX2//POU3F2//RFX4//BMPR2//SOX4//BTF3//CARF//FOSL1//KLF7//CBFB//NMI//SLC35D1//COX8C//GPD1//GPD2//DPYSL2//HPRT1//AK4//CMPK1//IMPDH1//TET2//DCTPP1//GMNC//ESCO2//GADD45A//EPC2//UBE2W//USP28//UNG//KDM2A//MTRR//CASP3//BAHD1//ARID4B//DIRAS3//ZMYND11//KLF17//NCOA7//ZNF782//ZNF800//ZFP30//HBP1//ATAD2//MDFIC//HOXA3//HOXB6//HOXD1//ZNF680//NEUROG1//NFE2//COMMD2//VGLL1//BRWD1//PRKAA1//TRERF1//SLC2A4RG//ZNF630//ZBTB26//MIER1//RFX3//ATXN1//DMRTA1//SMARCD2//ZNF3//ZNF131//ZNF134//ZNF208//ZNF227//ZNF655//MED28//TSC22D4//PCGF5//LCOR//ST18//NRBF2//MLLT6//PAK6//BTG1//RHOXF2B//SNIP1//CBL//RPS6KA5//MED6//EMX2//BOLA3//NR3C2//RFX7//RHOXF2//BLZF1//ELAVL4//RTCA//SCAF8//CPEB1//QKI//AIMP1//MTPN//MDH1//G3BP2//EREG//DDX6//GLRX//INTS7//AGTR2//IL5//LRP6//NOS1//PPP3CB//WNT7A//ACVR1//RASD1//BTAF1//GSTM3//BMP3//SULF1//VWC2//CALM2//EGLN1//TNKS//CCT6A//GPHN//RNF180//MYOG//C5AR1//TDRD5//FZD6//FASTK//YTHDF2//ADCYAP1//DLL1//IL2//MET//NCK1//ARMCX3//YES1//NFATC2IP//DLG2//ENPP4//RAPGEF2//SULT6B1//TRIM8//IL1RAP//MYD88//EGLN3//RAP2C//GJA1//NPNT// |
| GO:0035883 | enteroendocrine cell differentiation | Biological process | 4 | 26 | 574 | 17653 | 4.7314392924149 | 0.00936352959104355 | 0.204937057036335 | 2.02856041269708 | 0.00696864111498258 | RFX3//CDK6//DLL1//NEUROD1// |
| GO:0050890 | cognition | Biological process | 17 | 277 | 574 | 17653 | 1.8874514144832 | 0.0094037312017972 | 0.205140404706784 | 2.02669979333321 | 0.029616724738676 | CASP3//CNTNAP2//PPP3CB//PRKAR2B//ATXN1//FOSL1//NRXN1//CEBPB//NTF3//KCNK10//CPEB3//LDLR//SNAP25//DDHD2//NPTN//EN1//NEUROG1// |
| GO:0010893 | positive regulation of steroid biosynthetic process | Biological process | 3 | 14 | 574 | 17653 | 6.59021901443504 | 0.00952390507243538 | 0.205140404706784 | 2.02118494198649 | 0.00522648083623693 | PRKAA1//IFNG//TNF// |
| GO:0034349 | glial cell apoptotic process | Biological process | 3 | 14 | 574 | 17653 | 6.59021901443504 | 0.00952390507243538 | 0.205140404706784 | 2.02118494198649 | 0.00522648083623693 | DLL1//CCL2//CASP3// |
| GO:0035112 | genitalia morphogenesis | Biological process | 3 | 14 | 574 | 17653 | 6.59021901443504 | 0.00952390507243538 | 0.205140404706784 | 2.02118494198649 | 0.00522648083623693 | LRP6//AR//NEUROG1// |
| GO:0055024 | regulation of cardiac muscle tissue development | Biological process | 8 | 91 | 574 | 17653 | 2.70367959566566 | 0.00952448426407365 | 0.205140404706784 | 2.02115853138263 | 0.0139372822299652 | GJA1//JARID2//TGFBR2//ZFPM2//EDN1//IGF1//AKAP6//DLL1// |
| GO:0051090 | regulation of DNA binding transcription factor activity | Biological process | 23 | 416 | 574 | 17653 | 1.70036099571161 | 0.00963098216195005 | 0.20677565828974 | 2.01632942151877 | 0.0400696864111498 | G3BP2//LRP8//RPS6KA5//ESR1//EGLN1//FZD6//BHLHE40//EDN1//IL5//LRP6//NEUROD1//NEUROG1//NHLH2//PPARG//TNF//FOSL1//TRIM8//ARID5B//MTPN//IL1RAP//AR//MYD88//POU4F1// |
| GO:0051093 | negative regulation of developmental process | Biological process | 45 | 968 | 574 | 17653 | 1.42969627379273 | 0.0101310312113192 | 0.214677068458779 | 1.99434634668393 | 0.078397212543554 | BMPR2//BNIP3//STRAP//LDLRAD4//FRZB//PPARG//BHLHE41//STMN2//PTPRG//SPOCK1//TSC1//SPRED1//SULF1//RFX4//CCR1//DLL1//IFNG//IRF1//CDK6//ANP32B//ZFPM2//GATA2//TNF//REG3G//SOCS5//DDX6//FZD3//ULK2//WNT7A//RAPGEF2//EREG//MED28//GJA1//JARID2//TGFBR2//ADCYAP1//IGF1//LDLR//MEOX2//MMRN2//SLIT2//EFNA5//CLASP2//IL2//PHF14// |
| GO:0048513 | animal organ development | Biological process | 134 | 3428 | 574 | 17653 | 1.20218308742514 | 0.0101345619965247 | 0.214677068458779 | 1.99419501624605 | 0.233449477351916 | WNT7A//CEBPB//ADCYAP1//SPO11//INHBB//DMRTA1//SOD1//ESR1//EREG//SMOC1//NPNT//SLIT2//ACVR1//TMED10//SULF1//TSC1//ARID5B//FOXF2//DLG5//AK4//JARID2//MET//PPARG//GATA2//BMPR2//APCDD1//FZD3//FZD6//GJA1//DLL1//TGFBR2//RUNX3//ESCO2//GPR183//ITM2A//SOX4//ZFPM2//UBE4B//POU4F1//RFX3//CDK6//NEUROD1//LRP6//CRKL//CCL2//BHLHE41//DPYSL2//AGTR2//RAB3GAP1//CNTNAP2//SPHK2//PTPRG//ATXN1//EDN1//PPP3CB//CASP3//IGF1//ZBTB18//MEOX2//MYOG//RNF38//TEX11//CBL//HOXA3//CLASP2//STRAP//LDLRAD4//PTF1A//KBTBD8//FRZB//MTPN//RFX4//C5AR1//LRP8//EMX2//EN1//ANP32B//RAPGEF2//HPRT1//ARX//CALM2//POU3F2//NR4A2//TACC2//BNIP3//CBFB//TET2//IFNA1//PTPN22//IL2//TNF//HSD11B1//CCR1//AR//OTX2//KRT27//DSC2//KRTAP4-11//MAF//LRIG1//IFNG//MGST1//NEUROG1//TSHZ1//GPD2//IRF1//ARRDC3//CD8A//REG3G//MYB//SOCS5//IL5//NFE2//GJB2//PHF14//MTM1//PRKAA1//HOXB6//HOXC8//BMP3//EGLN1//TBL1XR1//ZNF568//FOSL1//BTBD7//VEGFB//SPRED1//AKAP6//TMOD1//KLHL3//NFIA//ARID4B//YTHDF2//NLN// |
| GO:0046128 | purine ribonucleoside metabolic process | Biological process | 7 | 74 | 574 | 17653 | 2.909195781147 | 0.0101741820782601 | 0.214677068458779 | 1.99250049445422 | 0.0121951219512195 | HPRT1//IMPDH1//GPHN//MTRR//DLG2//AK4//ENPP4// |
| GO:0021575 | hindbrain morphogenesis | Biological process | 5 | 41 | 574 | 17653 | 3.75053114642645 | 0.010186317302024 | 0.214677068458779 | 1.99198279975106 | 0.00871080139372822 | LRP6//DLL1//RFX4//MTPN//WNT7A// |
| GO:0060999 | positive regulation of dendritic spine development | Biological process | 5 | 41 | 574 | 17653 | 3.75053114642645 | 0.010186317302024 | 0.214677068458779 | 1.99198279975106 | 0.00871080139372822 | LRP8//CPEB3//FMR1//IL2//DLG5// |
| GO:0021953 | central nervous system neuron differentiation | Biological process | 12 | 171 | 574 | 17653 | 2.15820037899627 | 0.0101894631044312 | 0.214677068458779 | 1.99184869891073 | 0.0209059233449477 | GATA2//SOX4//MTPN//WNT7A//ARX//SLIT2//RAPGEF2//HPRT1//NR4A2//LRP6//POU4F1//SPOCK1// |
| GO:0031329 | regulation of cellular catabolic process | Biological process | 39 | 814 | 574 | 17653 | 1.47348877226926 | 0.0102301044255899 | 0.214863963448026 | 1.99011993312792 | 0.0679442508710801 | DRAM1//RRAGD//ULK2//PTPN22//MET//MTMR9//IFNG//PIK3CB//PRKAA1//BNIP3//TRIM8//LDLR//RAB12//TSC1//SNX5//CASP3//LAMTOR3//EGLN1//MTM1//RNF180//RNF125//LONRF3//LONRF1//SOCS5//DCP2//FMR1//YTHDF2//MYOG//GPD1//IGF1//SH3D19//TNF//CPEB3//CNOT7//CNOT6L//QKI//MYD88//CALCOCO2//RAB3GAP1// |
| GO:0032990 | cell part morphogenesis | Biological process | 33 | 663 | 574 | 17653 | 1.53075977107541 | 0.0103749519158467 | 0.217231578349447 | 1.9840139074092 | 0.0574912891986063 | OGN//POU4F1//WNT7A//SLIT2//ARX//DOK1//DPYSL2//EFNA5//OTX2//PIK3CB//ENAH//KLF7//RPS6KA5//NRXN1//NR4A2//FZD3//ULK2//PPP3CB//NYAP1//CNTNAP2//GJA1//NTF3//HPRT1//FMR1//HECW2//BMPR2//POU3F2//RAPGEF2//LRP8//MTM1//SNX2//PLAA//FLRT1// |
| GO:0048729 | tissue morphogenesis | Biological process | 32 | 639 | 574 | 17653 | 1.5401242141194 | 0.010588677088945 | 0.220931844530838 | 1.97515829568664 | 0.0557491289198606 | EDN1//TGFBR2//ACVR1//NPNT//BMPR2//FZD3//SOX4//LRP6//TSC1//FZD6//GJA1//DLL1//ARHGAP12//ZFPM2//UBE4B//POU4F1//DLG5//KRT27//WNT7A//FOXF2//MET//SLIT2//EGLN1//FRZB//AR//ESR1//SULF1//BTBD7//TNF//KLHL3//AGTR2//CLASP2// |
| GO:0006874 | cellular calcium ion homeostasis | Biological process | 23 | 420 | 574 | 17653 | 1.68416708146673 | 0.0107145304308294 | 0.220931844530838 | 1.97002685735834 | 0.0400696864111498 | ADCYAP1//CCR1//EDN1//ESR1//GATA2//GJA1//NPTN//IL2//LRP6//CCL28//PTGFR//C5AR1//NOS1//TRPC3//CALM2//AKAP6//BNIP3//FAM155A//GRIK2//PIK3CB//STIM2//CCL8//CALCB// |
| GO:0021846 | cell proliferation in forebrain | Biological process | 4 | 27 | 574 | 17653 | 4.55620080010324 | 0.0107150147872416 | 0.220931844530838 | 1.97000722527423 | 0.00696864111498258 | POU3F2//ARX//EMX2//WNT7A// |
| GO:0048643 | positive regulation of skeletal muscle tissue development | Biological process | 4 | 27 | 574 | 17653 | 4.55620080010324 | 0.0107150147872416 | 0.220931844530838 | 1.97000722527423 | 0.00696864111498258 | DLL1//MTM1//MYOG//PRKAA1// |
| GO:0071402 | cellular response to lipoprotein particle stimulus | Biological process | 4 | 27 | 574 | 17653 | 4.55620080010324 | 0.0107150147872416 | 0.220931844530838 | 1.97000722527423 | 0.00696864111498258 | LDLR//PPARG//SOCS5//MYD88// |
| GO:0046483 | heterocycle metabolic process | Biological process | 226 | 6136 | 574 | 17653 | 1.13273864415865 | 0.0107596024356791 | 0.221176873168686 | 1.96820377543363 | 0.393728222996516 | RAD9B//IGF1//NFIA//ATR//WRNIP1//RBBP8//RRM2//DTD1//NPAT//TSHZ1//ZBTB18//CEBPB//ZNF268//TCERG1//STRAP//KLF12//ARX//EDN1//CC2D1B//EN1//ESR1//CPEB3//MYT1L//RCOR1//ZFPM2//GATA2//CNOT7//HOXC8//HSBP1//IFNG//IRF2//AR//JARID2//ZNF568//MAF//MDM4//MYB//NR4A2//KLF3//POU4F1//PPARG//DUSP22//ZBTB4//TNF//ZNF217//LRP8//BHLHE41//TBL1XR1//ARID5B//BHLHE40//RUNX3//PHF14//DCP2//CNOT6L//PAN3//CNOT6//FMR1//MBNL1//SMU1//RBM25//SRSF2//POLR2K//RBM41//SNRPN//NOB1//SPO11//SYCP1//TEX11//PTGES3//NABP1//ELK3//TFEC//FOXJ3//FOXF2//PTF1A//HIVEP2//MACC1//IRF1//MEOX2//MYBL1//NEUROD1//NHLH2//OTX2//POU3F2//RFX4//BMPR2//SOX4//BTF3//CARF//FOSL1//KLF7//CBFB//NMI//SLC35D1//COX8C//GPD1//GPD2//DPYSL2//HPRT1//AK4//CMPK1//IMPDH1//TET2//DCTPP1//GMNC//ESCO2//GADD45A//EPC2//UBE2W//USP28//UNG//KDM2A//MTRR//CASP3//BAHD1//ARID4B//DIRAS3//ZMYND11//KLF17//NCOA7//ZNF782//ZNF800//ZFP30//HBP1//ATAD2//MDFIC//HOXA3//HOXB6//HOXD1//ZNF680//NEUROG1//NFE2//COMMD2//VGLL1//BRWD1//PRKAA1//TRERF1//SLC2A4RG//ZNF630//ZBTB26//MIER1//RFX3//ATXN1//DMRTA1//SMARCD2//ZNF3//ZNF131//ZNF134//ZNF208//ZNF227//ZNF655//MED28//TSC22D4//PCGF5//LCOR//ST18//NRBF2//MLLT6//PAK6//BTG1//RHOXF2B//SNIP1//CBL//RPS6KA5//MED6//EMX2//BOLA3//NR3C2//RFX7//RHOXF2//BLZF1//ELAVL4//RTCA//SCAF8//CPEB1//QKI//AIMP1//MDH1//GPHN//G3BP2//EREG//DDX6//GLRX//INTS7//AGTR2//IL5//LRP6//NOS1//PPP3CB//WNT7A//ACVR1//RASD1//BTAF1//BMP3//SULF1//VWC2//CALM2//EGLN1//TNKS//CCT6A//RNF180//MYOG//C5AR1//TDRD5//FZD6//FASTK//YTHDF2//ADCYAP1//DLL1//IL2//MET//NCK1//ARMCX3//YES1//NFATC2IP//DLG2//ENPP4//SULT6B1//TRIM8//MTPN//IL1RAP//MYD88//EGLN3//RAP2C//GJA1//NPNT// |
| GO:1903038 | negative regulation of leukocyte cell-cell adhesion | Biological process | 9 | 112 | 574 | 17653 | 2.47133213041314 | 0.01082940563017 | 0.22193718265709 | 1.96539537888908 | 0.0156794425087108 | CEBPB//DLG5//IRF1//SOCS5//CASP3//PTPN22//DUSP22//CCL28//IL2// |
| GO:0060420 | regulation of heart growth | Biological process | 7 | 75 | 574 | 17653 | 2.87040650406504 | 0.0109165415006706 | 0.222668249191206 | 1.96191492986176 | 0.0121951219512195 | GJA1//JARID2//TGFBR2//ZFPM2//EDN1//IGF1//AKAP6// |
| GO:0021537 | telencephalon development | Biological process | 15 | 237 | 574 | 17653 | 1.94647818991752 | 0.0109309269157889 | 0.222668249191206 | 1.96134300938204 | 0.0261324041811847 | LRP8//CDK6//EMX2//NEUROD1//CNTNAP2//HPRT1//TSC1//CASP3//POU3F2//ARX//SLIT2//TACC2//LRP6//BNIP3//RFX4// |
| GO:0009888 | tissue development | Biological process | 82 | 1969 | 574 | 17653 | 1.28078067184212 | 0.011006374737282 | 0.222958667959499 | 1.95835570472101 | 0.142857142857143 | WNT7A//EDN1//TGFBR2//ACVR1//NPNT//SLIT2//BMPR2//FZD3//DLL1//MEOX2//FOXF2//DLG5//SOX4//LRP6//TSC1//FZD6//MET//APCDD1//GJA1//ARHGAP12//RUNX3//SULF1//ESR1//ZFPM2//UBE4B//POU4F1//RFX3//CDK6//NEUROD1//AR//FRMD6//ZBTB18//MYOG//GSTM3//POU3F2//CLASP2//STRAP//LDLRAD4//KBTBD8//FRZB//IGF1//MTPN//EREG//CASP3//CCR1//GSTA2//PPARG//IFNG//EN1//KRT27//DSC2//KRTAP4-11//MAF//CEBPB//HSBP1//BTG1//CBFB//REG3G//GJB2//MTM1//PRKAA1//HOXA3//BMP3//TNF//EGLN1//JARID2//SOD1//ARID5B//ARRDC3//TBL1XR1//BTBD7//RAPGEF2//AGTR2//AKAP6//TMOD1//ARX//KLHL3//RAP2C//ARID4B//NLN//PTF1A//GATA2// |
| GO:0040012 | regulation of locomotion | Biological process | 45 | 973 | 574 | 17653 | 1.42234942757591 | 0.0110329639460625 | 0.222958667959499 | 1.957307801023 | 0.078397212543554 | CCL8//SLIT2//EDN1//BMPR2//WNT7A//STRAP//PTPRG//CLASP2//IFNG//TGFBR2//C5AR1//C5ORF30//IGF1//NCK1//ZNF268//CCR1//NTF3//FAM110C//PTP4A1//ACVR1//SULF1//RAP2C//LDLRAD4//DLG5//AGTR2//PPARG//VEGFB//TNF//SPRED1//MEOX2//MMRN2//ARRDC3//CHMP3//TRIM8//CCL28//CDK6//EREG//MEMO1//BTC//GCSAML//GPR183//CCL2//MET//PLAA//RAPGEF2// |
| GO:0042060 | wound healing | Biological process | 28 | 542 | 574 | 17653 | 1.58878588785888 | 0.0110440860219477 | 0.222958667959499 | 1.95687021931712 | 0.0487804878048781 | PROCR//ENPP4//MMRN1//RCOR1//ZFPM2//GATA2//IFNA1//IRF1//IRF2//NFE2//PRKAR2B//IGF1//WNT7A//DGKH//PIK3CB//TRPC3//FZD6//DGKE//EDN1//MTPN//GJA1//MYOG//TGFBR2//REG3G//CLASP2//ELK3//EREG//CASP3// |
| GO:0097755 | positive regulation of blood vessel diameter | Biological process | 6 | 58 | 574 | 17653 | 3.1814850414514 | 0.0111358306401325 | 0.224141733985762 | 1.95327738267382 | 0.0104529616724739 | AGTR2//NOS1//BMPR2//SOD1//ADCYAP1//GJA1// |
| GO:0046473 | phosphatidic acid metabolic process | Biological process | 5 | 42 | 574 | 17653 | 3.66123278579725 | 0.0112613900779053 | 0.224870176910892 | 1.94840799794805 | 0.00871080139372822 | DDHD2//LCLAT1//GPD1//PLA2G12A//DGKH// |
| GO:0045165 | cell fate commitment | Biological process | 16 | 260 | 574 | 17653 | 1.89257571696596 | 0.0112623522735044 | 0.224870176910892 | 1.94837089254763 | 0.0278745644599303 | GATA2//DLL1//NEUROD1//MYOG//WNT7A//MYT1L//PTF1A//POU3F2//POU4F1//AR//CEBPB//PPARG//ACVR1//ARX//NEUROG1//CASP3// |
| GO:0120039 | plasma membrane bounded cell projection morphogenesis | Biological process | 32 | 642 | 574 | 17653 | 1.53292737199735 | 0.0112783264632027 | 0.224870176910892 | 1.94775533844819 | 0.0557491289198606 | OGN//POU4F1//WNT7A//SLIT2//ARX//DOK1//DPYSL2//EFNA5//OTX2//PIK3CB//ENAH//KLF7//RPS6KA5//NRXN1//NR4A2//FZD3//ULK2//PPP3CB//NYAP1//CNTNAP2//GJA1//NTF3//HPRT1//FMR1//HECW2//BMPR2//POU3F2//RAPGEF2//LRP8//SNX2//PLAA//FLRT1// |
| GO:0006644 | phospholipid metabolic process | Biological process | 25 | 470 | 574 | 17653 | 1.63586996812217 | 0.0113050214623249 | 0.224870176910892 | 1.94672860881969 | 0.0435540069686411 | PIGP//PIGA//DDHD2//LCLAT1//GPD1//PLA2G12A//CDS1//ABHD3//SLC44A1//SERINC3//MTM1//PIK3CB//MTMR9//SPHK2//DGKE//LDLR//PIGS//TNFAIP8L3//DGKH//EREG//ESR1//EFR3A//MET//BTC//PLAA// |
| GO:0044238 | primary metabolic process | Biological process | 381 | 10905 | 574 | 17653 | 1.07449879941912 | 0.0113642616016816 | 0.225385634053292 | 1.94445877751342 | 0.663763066202091 | RAD9B//IGF1//NFIA//ATR//WRNIP1//RBBP8//RRM2//DTD1//GADD45A//DIRAS3//NPAT//MTRR//TSHZ1//ZBTB18//CEBPB//ZNF268//TCERG1//STRAP//KLF12//ARX//EDN1//CC2D1B//EN1//ESR1//CPEB3//MYT1L//RCOR1//ZFPM2//GATA2//CNOT7//HOXC8//HSBP1//IFNG//IRF2//AR//JARID2//ZNF568//MAF//MDM4//MYB//NR4A2//KLF3//POU4F1//PPARG//DUSP22//ZBTB4//TNF//ZNF217//LRP8//BHLHE41//TBL1XR1//ARID5B//BHLHE40//RUNX3//PHF14//CSF2RA//SPRED1//EREG//RASGEF1A//IL2//IL5//MAP3K1//MET//MAP3K7CL//CCL2//BTC//LAMTOR3//RAPGEF2//DCP2//CRKL//NTF3//PIK3CB//PRKAA1//SOD1//C5AR1//UBE4B//RNF145//ZNRF2//RNF180//RNF125//UBE2W//HECW2//SKP1//THOP1//UBE2G1//LONRF3//CBFB//TNKS//CBL//UBE3B//LONRF1//CNOT6L//PAN3//CNOT6//FMR1//MBNL1//SMU1//RBM25//SRSF2//POLR2K//RBM41//SNRPN//NOB1//SPO11//SYCP1//TEX11//PTGES3//NABP1//SECISBP2L//MGST1//ADH4//ST8SIA3//ACSL1//LRP6//PPP1R15B//SLIT2//EFNA5//NPTN//ELK3//TFEC//FOXJ3//FOXF2//PTF1A//HIVEP2//MACC1//IRF1//MEOX2//MYBL1//NEUROD1//NHLH2//OTX2//POU3F2//RFX4//BMPR2//SOX4//BTF3//CARF//FOSL1//KLF7//NMI//SOCS5//RNF38//KLHL3//SPOPL//DCAF8//TRIM8//KBTBD8//FUT9//FUT2//ST8SIA5//GLT6D1//SLC2A5//CHST7//CHST1//AIMP1//SLC35D1//GPD1//GPD2//MDH1//NLN//COX8C//DPYSL2//HPRT1//AK4//CMPK1//IMPDH1//DCTPP1//GMNC//ESCO2//EPC2//USP28//UNG//KDM2A//CASP3//BAHD1//ARID4B//ZMYND11//KLF17//NCOA7//ZNF782//ZNF800//ZFP30//HBP1//ATAD2//MDFIC//HOXA3//HOXB6//HOXD1//ZNF680//NEUROG1//NFE2//COMMD2//VGLL1//BRWD1//TRERF1//SLC2A4RG//ZNF630//ZBTB26//MIER1//RFX3//ATXN1//DMRTA1//SMARCD2//ZNF3//ZNF131//ZNF134//ZNF208//ZNF227//ZNF655//MED28//TSC22D4//PCGF5//LCOR//ST18//NRBF2//MLLT6//PAK6//BTG1//RHOXF2B//SNIP1//RPS6KA5//MED6//EMX2//BOLA3//NR3C2//RFX7//RHOXF2//BLZF1//ELAVL4//RTCA//SCAF8//CPEB1//QKI//CPEB2//MRPL35//METTL17//EIF3B//MTPN//DDX6//TSC1//YES1//IMMP1L//CDK6//FASTK//PDIK1L//CDK19//PPP3CB//CCL8//TGFBR2//ACVR1//FLRT1//NCK1//DBNDD2//PTPN22//MTM1//CPPED1//HS3ST5//MAN1A1//CCDC126//TET2//VEGFB//PIGP//PIGA//GGT6//PRSS55//MEP1A//DESI2//PGC//NPEPL1//ADAM12//ADAM18//LMLN//SLC6A14//SLC6A6//NOS1//GLUD2//LGSN//SERINC3//LDLR//MID1IP1//MSMO1//PRKAR2B//TNFAIP8L3//PLAA//DDHD2//LCLAT1//PLA2G12A//CDS1//ABHD3//SLC44A1//MTMR9//SPHK2//ARSK//NEU1//ACBD3//HSD11B1//GPHN//ANP32B//EGLN3//VSIG4//DLL1//G3BP2//DGKE//ACBD5//CALM2//INHBB//BMP3//A2ML1//WFDC13//SPOCK1//NDST3//GLRX//IAH1//PRR16//INTS7//PIGS//GNPTG//AGTR2//WNT7A//RASD1//BTAF1//OTUD3//WDR20//NFATC2IP//VKORC1L1//STK33//EGLN1//ELOVL7//DCUN1D4//MSRB3//SULF1//DSEL//IDS//VWC2//CDC27//RAP2C//CCT6A//PHACTR2//AKAP6//IFNA1//PTPRG//PTP4A1//CDC14A//BNIP3//IL1RAP//RDH14//MYOG//ITM2A//TDRD5//ARL6IP1//UBXN2B//CD2AP//VPS37A//FZD6//YTHDF2//RAB12//GJA1//CCNY//ADCYAP1//ARMCX3//DLG2//ENPP4//GRIK2//DGKH//TMEM86A//ULK2//EFR3A//SULT6B1//SAMSN1//SH3D19//MYD88//ARRDC3//PMEPA1//LDLRAD4//ATG16L1//CCR1//GPR183//NPNT//UBA5//TMED10//RAB3GAP1// |
| GO:0055017 | cardiac muscle tissue growth | Biological process | 8 | 94 | 574 | 17653 | 2.61739194899548 | 0.0114722297378787 | 0.225847312574398 | 1.94035216459695 | 0.0139372822299652 | GJA1//JARID2//TGFBR2//ZFPM2//AGTR2//EDN1//IGF1//AKAP6// |
| GO:0007167 | enzyme linked receptor protein signaling pathway | Biological process | 47 | 1028 | 574 | 17653 | 1.40608434224976 | 0.0115545173009225 | 0.225847312574398 | 1.93724819280058 | 0.0818815331010453 | DOK1//NTF3//PIK3CB//PTPRG//YES1//CD8A//BMPR2//AKAP4//EREG//CBL//SOCS5//EFNA5//EPS15//BTC//CAMLG//RPS6KA5//DUSP22//TGFBR2//ACVR1//APPL1//SPRED1//FLRT1//POLR2K//INHBB//BMP3//PMEPA1//LDLRAD4//NPNT//STRAP//SULF1//VWC2//MMRN2//RAPGEF2//OTX2//SHISA2//IGF1//AR//NPTN//NCK1//TSC1//ARID5B//VEGFB//CASP3//MET//AGTR2//DLL1//PHF14// |
| GO:0046879 | hormone secretion | Biological process | 18 | 306 | 574 | 17653 | 1.80907972945276 | 0.0115628600380965 | 0.225847312574398 | 1.93693473121514 | 0.0313588850174216 | AGTR2//RAPGEF4//IFNG//NEUROD1//PPP3CB//RFX3//SNAP25//TNF//INHBB//EDN1//ADCYAP1//GJA1//SOX4//SYBU//EFNA5//NRXN1//AIMP1//MYB// |
| GO:0001101 | response to acid chemical | Biological process | 19 | 329 | 574 | 17653 | 1.77608739396122 | 0.0115712944977604 | 0.225847312574398 | 1.93661805316698 | 0.0331010452961672 | EDN1//CEBPB//CPEB3//RRAGD//CPEB1//TNF//LAMTOR3//GJA1//GJB2//PPARG//ACSL1//GLRB//CASP3//MYB//MYOG//YES1//PRKAA1//LDLR//PTGFR// |
| GO:0021542 | dentate gyrus development | Biological process | 3 | 15 | 574 | 17653 | 6.15087108013937 | 0.0116213019038726 | 0.225847312574398 | 1.93474521635223 | 0.00522648083623693 | CDK6//EMX2//NEUROD1// |
| GO:0051044 | positive regulation of membrane protein ectodomain proteolysis | Biological process | 3 | 15 | 574 | 17653 | 6.15087108013937 | 0.0116213019038726 | 0.225847312574398 | 1.93474521635223 | 0.00522648083623693 | SH3D19//IFNG//TNF// |
| GO:0090141 | positive regulation of mitochondrial fission | Biological process | 3 | 15 | 574 | 17653 | 6.15087108013937 | 0.0116213019038726 | 0.225847312574398 | 1.93474521635223 | 0.00522648083623693 | MIEF2//DDHD2//BNIP3// |
| GO:0071396 | cellular response to lipid | Biological process | 29 | 569 | 574 | 17653 | 1.56744517859439 | 0.0116572158024677 | 0.225896133157848 | 1.93340516364311 | 0.0505226480836237 | ESR1//AR//PMEPA1//PTPN22//CCL2//TNF//UBA5//CBFB//NR3C2//NR4A2//PPARG//PAQR5//CEBPB//CDK19//PLAA//MYB//MYOG//YES1//PRKAA1//ADCYAP1//EDN1//POU4F1//LRP6//LRP8//LDLR//AGTR2//GJB2//PTGFR//EFNA5// |
| GO:0048858 | cell projection morphogenesis | Biological process | 32 | 644 | 574 | 17653 | 1.52816672798494 | 0.0117581894896985 | 0.227201815768088 | 1.92965954519581 | 0.0557491289198606 | OGN//POU4F1//WNT7A//SLIT2//ARX//DOK1//DPYSL2//EFNA5//OTX2//PIK3CB//ENAH//KLF7//RPS6KA5//NRXN1//NR4A2//FZD3//ULK2//PPP3CB//NYAP1//CNTNAP2//GJA1//NTF3//HPRT1//FMR1//HECW2//BMPR2//POU3F2//RAPGEF2//LRP8//SNX2//PLAA//FLRT1// |
| GO:0009725 | response to hormone | Biological process | 45 | 977 | 574 | 17653 | 1.41652609317437 | 0.0118015902474222 | 0.227390754539363 | 1.92805946818988 | 0.078397212543554 | CHRM2//APPL1//PPARG//ESR1//AR//PMEPA1//GJB2//PTGFR//RBBP8//WNT7A//CASP3//FOSL1//IGF1//TSC1//CPEB2//INHBB//CPEB1//SLIT2//UBA5//CBFB//ACSL1//EDN1//CBL//AGTR2//NR3C2//NR4A2//PAQR5//REG3G//EREG//GJA1//LRP6//BTG1//NCK1//TGFBR2//TNF//EFNA5//PRKAR2B//PRKAA1//ADCYAP1//MYOG//POU4F1//FIBIN//STEAP2//NOS1//SNRPN// |
| GO:0007389 | pattern specification process | Biological process | 23 | 424 | 574 | 17653 | 1.66827871277365 | 0.0118944632257182 | 0.228529132941853 | 1.92465515206039 | 0.0400696864111498 | WNT7A//DLL1//MEOX2//GJA1//TGFBR2//LRP6//NEUROG1//ACVR1//TSHZ1//CRKL//EMX2//HOXA3//HOXB6//HOXC8//NEUROD1//BMPR2//EDN1//EN1//RFX4//TDRD5//RFX3//AR//OTX2// |
| GO:0002695 | negative regulation of leukocyte activation | Biological process | 11 | 154 | 574 | 17653 | 2.19673967147835 | 0.0119989175937388 | 0.229882945287409 | 1.92085792930809 | 0.0191637630662021 | CASP3//CD84//CEBPB//DLG5//IRF1//SOCS5//IL2//PTPN22//DUSP22//SAMSN1//LDLR// |
| GO:0045667 | regulation of osteoblast differentiation | Biological process | 9 | 114 | 574 | 17653 | 2.4279754263708 | 0.0120741248519512 | 0.230670356987983 | 1.91814433765655 | 0.0156794425087108 | CDK6//TNF//CEBPB//NPNT//GJA1//IGF1//BMPR2//ACVR1//SMOC1// |
| GO:0016310 | phosphorylation | Biological process | 96 | 2367 | 574 | 17653 | 1.24732493386857 | 0.0121201117771617 | 0.230896664644914 | 1.9164933748908 | 0.167247386759582 | GADD45A//DIRAS3//CSF2RA//SPRED1//EREG//RASGEF1A//IL2//IL5//MAP3K1//MET//MAP3K7CL//CCL2//BTC//TNF//LAMTOR3//RAPGEF2//CRKL//IGF1//NTF3//PIK3CB//PRKAA1//SOD1//C5AR1//DUSP22//LRP6//PPP1R15B//SLIT2//EFNA5//FMR1//NPTN//IFNG//CBL//SOCS5//COX8C//AK4//CMPK1//CDK6//FASTK//PDIK1L//CDK19//PAN3//PPP3CB//CCL8//TGFBR2//RUNX3//ACVR1//RPS6KA5//FLRT1//NCK1//DBNDD2//MDFIC//CALM2//INHBB//BMP3//BMPR2//ATR//STK33//TNKS//PAK6//RAP2C//IFNA1//PRKAR2B//YES1//PTGES3//AR//CNOT7//EDN1//PTPN22//NRBF2//TNFAIP8L3//CASP3//CCNY//MYOG//GPD1//TSC1//ADCYAP1//GRIK2//ZMYND11//WNT7A//ULK2//SPHK2//DGKE//GNPTG//ESR1//EFR3A//LRP8//SAMSN1//SKP1//STRAP//PMEPA1//LDLRAD4//CCR1//GPR183//NPNT//ACSL1//DGKH// |
| GO:0060548 | negative regulation of cell death | Biological process | 46 | 1005 | 574 | 17653 | 1.40766203824085 | 0.0121734871747011 | 0.231262061130628 | 1.91458499746461 | 0.0801393728222996 | IL2//ZNF268//ARL6IP1//IGF1//MDM4//MET//MYD88//ZFAND6//PRKAA1//TEX11//SPHK2//PTGFR//BCL2A1//BNIP3//BTC//UNG//WNT7A//CASP3//CBL//ADCYAP1//DLL1//CCL2//EDN1//LRP6//POU4F1//CEBPB//EN1//GRIK2//NTF3//NR4A2//SOD1//C5AR1//TSC22D4//FZD3//NCOA7//TSC1//CFDP1//GATA2//NEUROD1//ZMYND11//AR//ACVR1//TNF//ZFPM2//NCK1//SOX4// |
| GO:0055082 | cellular chemical homeostasis | Biological process | 37 | 773 | 574 | 17653 | 1.47207134518213 | 0.0123572647970327 | 0.234095747401491 | 1.90807764696655 | 0.0644599303135888 | CCR1//GRIK2//PIK3CB//STIM2//CCL8//CALCB//EGLN1//SKP1//SOD1//SLC30A5//AGTR2//ADCYAP1//EDN1//ESR1//GATA2//GJA1//NPTN//IL2//LRP6//CCL28//PTGFR//C5AR1//NOS1//TRPC3//CALM2//AKAP6//SLC41A1//SLC30A7//PPP3CB//SYBU//SLC4A4//BNIP3//EFNA5//NRXN1//NEUROD1//SOX4//FAM155A// |
| GO:0008610 | lipid biosynthetic process | Biological process | 34 | 697 | 574 | 17653 | 1.50021245857058 | 0.0124147293793283 | 0.234527415621222 | 1.9060627429113 | 0.0592334494773519 | PTGES3//EDN1//MGST1//ST8SIA3//PIGP//PIGA//PRKAA1//DDHD2//LCLAT1//GPD1//PLA2G12A//CDS1//ABHD3//SLC44A1//MTM1//PIK3CB//MTMR9//SPHK2//ST8SIA5//TRERF1//ACBD3//MSMO1//HSD11B1//DGKE//GLT6D1//LDLR//PIGS//ELOVL7//QKI//SOD1//MID1IP1//IFNG//TNF//ACSL1// |
| GO:0010975 | regulation of neuron projection development | Biological process | 24 | 450 | 574 | 17653 | 1.64023228803717 | 0.0125167299070007 | 0.235162555698035 | 1.90250911911646 | 0.0418118466898955 | STMN2//ADCYAP1//NPTN//NCK1//RAPGEF2//PTPRG//SPOCK1//TSC1//DPYSL2//WNT7A//ULK2//EFNA5//FMR1//HECW2//BMPR2//POU3F2//SLIT2//CPEB3//IL2//DLG5//LRP8//IL1RAP//PLAA//SNAP25// |
| GO:0042278 | purine nucleoside metabolic process | Biological process | 7 | 77 | 574 | 17653 | 2.79585049097244 | 0.0125178944331351 | 0.235162555698035 | 1.90246871529273 | 0.0121951219512195 | HPRT1//IMPDH1//GPHN//MTRR//DLG2//AK4//ENPP4// |
| GO:0014706 | striated muscle tissue development | Biological process | 21 | 379 | 574 | 17653 | 1.70406718579059 | 0.012706475561686 | 0.23804402832045 | 1.89597489447919 | 0.0365853658536585 | ZFPM2//UBE4B//POU4F1//ZBTB18//MEOX2//MYOG//MTPN//GJA1//DLL1//MTM1//PRKAA1//TSC1//EGLN1//JARID2//TGFBR2//ACVR1//AGTR2//EDN1//IGF1//AKAP6//NLN// |
| GO:2000145 | regulation of cell motility | Biological process | 42 | 904 | 574 | 17653 | 1.42885279516512 | 0.0128603878137489 | 0.240261886144707 | 1.89074593476859 | 0.0731707317073171 | CCL8//SLIT2//EDN1//BMPR2//WNT7A//STRAP//PTPRG//CLASP2//IFNG//TGFBR2//C5AR1//C5ORF30//IGF1//NCK1//ZNF268//CCR1//NTF3//FAM110C//PTP4A1//ACVR1//SULF1//RAP2C//LDLRAD4//DLG5//AGTR2//PPARG//VEGFB//TNF//SPRED1//MEOX2//MMRN2//CCL28//GCSAML//GPR183//CCL2//MET//PLAA//RAPGEF2//CDK6//EREG//MEMO1//BTC// |
| GO:0045669 | positive regulation of osteoblast differentiation | Biological process | 6 | 60 | 574 | 17653 | 3.07543554006969 | 0.0130489133100383 | 0.243112398666085 | 1.88442565408085 | 0.0104529616724739 | CEBPB//NPNT//GJA1//IGF1//BMPR2//ACVR1// |
| GO:0031400 | negative regulation of protein modification process | Biological process | 30 | 600 | 574 | 17653 | 1.53771777003484 | 0.0133265123182606 | 0.246843256510256 | 1.87528349493125 | 0.0522648083623693 | SPRED1//DUSP22//IL2//LRP6//PPP1R15B//SLIT2//CBL//SOCS5//GADD45A//FLRT1//NCK1//DBNDD2//CALM2//JARID2//SPOPL//SOX4//CDK6//PHACTR2//TNF//PTPN22//CASP3//ZMYND11//NTF3//SAMSN1//STRAP//PMEPA1//LDLRAD4//PLAA//NCOA7//PRKAR2B// |
| GO:0034502 | protein localization to chromosome | Biological process | 7 | 78 | 574 | 17653 | 2.76000625390869 | 0.0133789932829324 | 0.246843256510256 | 1.87357656430483 | 0.0121951219512195 | MTBP//ATR//TNKS//ESCO2//ESR1//CCT6A//SPO11// |
| GO:0071260 | cellular response to mechanical stimulus | Biological process | 7 | 78 | 574 | 17653 | 2.76000625390869 | 0.0133789932829324 | 0.246843256510256 | 1.87357656430483 | 0.0121951219512195 | MTPN//GADD45A//GJA1//IRF1//MAP3K1//MYD88//BNIP3// |
| GO:0016049 | cell growth | Biological process | 25 | 477 | 574 | 17653 | 1.61186349060256 | 0.0133963708846263 | 0.246843256510256 | 1.87301283732529 | 0.0435540069686411 | SPOCK1//BLZF1//TGFBR2//MTPN//IL2//H3F3C//AKAP6//AGTR2//FRZB//GJA1//PPARG//BMPR2//BTG1//SLIT2//EI24//DPYSL2//EFNA5//ULK2//PPP3CB//EDN1//IGF1//PLAA//FLRT1//AR//SOCS5// |
| GO:0007612 | learning | Biological process | 10 | 136 | 574 | 17653 | 2.26134966181595 | 0.0134316602684865 | 0.246843256510256 | 1.8718703014879 | 0.0174216027874564 | SNAP25//DDHD2//NPTN//CNTNAP2//NRXN1//EN1//PPP3CB//PRKAR2B//ATXN1//FOSL1// |
| GO:0048738 | cardiac muscle tissue development | Biological process | 14 | 221 | 574 | 17653 | 1.94823970864143 | 0.0135597113337399 | 0.247979588445584 | 1.86774955585969 | 0.024390243902439 | ZFPM2//UBE4B//POU4F1//TSC1//EGLN1//GJA1//JARID2//TGFBR2//ACVR1//AGTR2//EDN1//IGF1//AKAP6//DLL1// |
| GO:0031018 | endocrine pancreas development | Biological process | 5 | 44 | 574 | 17653 | 3.49481311371555 | 0.0136380538755636 | 0.247979588445584 | 1.86524759825759 | 0.00871080139372822 | RFX3//CDK6//DLL1//NEUROD1//SOX4// |
| GO:0048599 | oocyte development | Biological process | 5 | 44 | 574 | 17653 | 3.49481311371555 | 0.0136380538755636 | 0.247979588445584 | 1.86524759825759 | 0.00871080139372822 | EREG//YTHDF2//TDRD5//IGF1//INHBB// |
| GO:0048639 | positive regulation of developmental growth | Biological process | 12 | 178 | 574 | 17653 | 2.07332733038406 | 0.0136908508223031 | 0.247979588445584 | 1.86356956165787 | 0.0209059233449477 | POU3F2//TGFBR2//ARX//DLL1//MTM1//EFNA5//BMPR2//ZFPM2//EDN1//IGF1//AKAP6//PLAA// |
| GO:0007205 | protein kinase C-activating G-protein coupled receptor signaling pathway | Biological process | 4 | 29 | 574 | 17653 | 4.24198005526853 | 0.0137807796066017 | 0.247979588445584 | 1.86072621281533 | 0.00696864111498258 | DGKH//EDN1//IL2//DGKE// |
| GO:0045589 | regulation of regulatory T cell differentiation | Biological process | 4 | 29 | 574 | 17653 | 4.24198005526853 | 0.0137807796066017 | 0.247979588445584 | 1.86072621281533 | 0.00696864111498258 | IFNG//IRF1//IL2//CBFB// |
| GO:2000516 | positive regulation of CD4-positive, alpha-beta T cell activation | Biological process | 4 | 29 | 574 | 17653 | 4.24198005526853 | 0.0137807796066017 | 0.247979588445584 | 1.86072621281533 | 0.00696864111498258 | IFNG//MYB//SOCS5//TGFBR2// |
| GO:0009628 | response to abiotic stimulus | Biological process | 53 | 1198 | 574 | 17653 | 1.36058500520612 | 0.0137956122620897 | 0.247979588445584 | 1.86025902036447 | 0.0923344947735192 | EGLN3//MYB//NOS1//NR4A2//EGLN1//PRKAA1//BNIP3//TGFBR2//VEGFB//CASP3//CLCN6//PPARG//FOSL1//CDS1//TRPC3//STRBP//DDHD2//NPTN//DNAJB4//IGF1//SOD1//UBE4B//MYOG//TNF//USP28//NABP1//CBL//MTPN//GADD45A//GJA1//IRF1//MAP3K1//MYD88//NEUROG1//CETN1//TFEC//FMR1//ATR//EDN1//CNTNAP2//NRXN1//BHLHE40//KCNJ3//FZD3//HSBP1//CPEB2//MDM4//CPEB1//RAD9B//INTS7//TSC1//PTGES3//HSPB8// |
| GO:0043410 | positive regulation of MAPK cascade | Biological process | 28 | 552 | 574 | 17653 | 1.56000353481796 | 0.0138234962064151 | 0.247979588445584 | 1.85938210232184 | 0.0487804878048781 | GADD45A//TNF//CRKL//MAP3K1//MAP3K7CL//LAMTOR3//IGF1//NTF3//PIK3CB//PRKAA1//SOD1//C5AR1//MDFIC//EDN1//DUSP22//WNT7A//ADCYAP1//CCR1//GPR183//NPNT//PTPN22//NPTN//TNFAIP8L3//MET//CCL2//CCL8//RAPGEF2//AR// |
| GO:0060413 | atrial septum morphogenesis | Biological process | 3 | 16 | 574 | 17653 | 5.76644163763066 | 0.0139630294494345 | 0.249160865874738 | 1.85502034600823 | 0.00522648083623693 | SOX4//ACVR1//BMPR2// |
| GO:0071786 | endoplasmic reticulum tubular network organization | Biological process | 3 | 16 | 574 | 17653 | 5.76644163763066 | 0.0139630294494345 | 0.249160865874738 | 1.85502034600823 | 0.00522648083623693 | RTN3//ARL6IP1//RAB3GAP1// |
| GO:0043085 | positive regulation of catalytic activity | Biological process | 61 | 1414 | 574 | 17653 | 1.32674376198197 | 0.0140309256535486 | 0.249210844201674 | 1.85291367658623 | 0.106271777003484 | GADD45A//TNF//CRKL//MAP3K1//MAP3K7CL//LAMTOR3//IGF1//NTF3//PIK3CB//PRKAA1//SOD1//C5AR1//EREG//ANP32B//EGLN3//PPARG//CASP3//EFNA5//MDFIC//NPNT//ESR1//MTMR9//NOS1//PAK6//TGFBR2//PLAA//AGTR2//CALM2//PPP1R15B//AKAP6//DNAJB4//ADCYAP1//ARAP2//DENND1B//RAB3GAP1//AGFG1//ARHGAP1//RP2//CCL2//CCL8//EVI5//ARHGAP12//RAPGEF2//USP6NL//MET//PRKAR2B//EDN1//TNFAIP8L3//CCNY//MID1IP1//DCUN1D4//ARRDC3//PTGES3//TNKS//IFNG//LRP8//ACSL1//TSC1//DYNLL2//ST18//NR4A2// |
| GO:0002009 | morphogenesis of an epithelium | Biological process | 27 | 528 | 574 | 17653 | 1.572665901172 | 0.014039528558456 | 0.249210844201674 | 1.85264747538988 | 0.0470383275261324 | EDN1//TGFBR2//ACVR1//NPNT//FZD3//SOX4//LRP6//TSC1//FZD6//GJA1//DLL1//ARHGAP12//DLG5//KRT27//WNT7A//FOXF2//MET//SLIT2//FRZB//AR//ESR1//SULF1//BTBD7//TNF//KLHL3//AGTR2//CLASP2// |
| GO:0002292 | T cell differentiation involved in immune response | Biological process | 6 | 61 | 574 | 17653 | 3.02501856400297 | 0.0140868575919898 | 0.249396381923107 | 1.851185875781 | 0.0104529616724739 | IFNG//TSC1//MYB//SOCS5//GPR183//IL2// |
| GO:0042471 | ear morphogenesis | Biological process | 9 | 117 | 574 | 17653 | 2.36571964620745 | 0.0141371542137608 | 0.249597834616175 | 1.84963800451686 | 0.0156794425087108 | LRIG1//GATA2//NEUROG1//FZD3//FZD6//TSHZ1//EDN1//SOD1//FRZB// |
| GO:0048812 | neuron projection morphogenesis | Biological process | 31 | 628 | 574 | 17653 | 1.51812900863313 | 0.0141754672143091 | 0.249597834616175 | 1.84846261813154 | 0.054006968641115 | OGN//POU4F1//WNT7A//SLIT2//ARX//DOK1//DPYSL2//EFNA5//OTX2//PIK3CB//ENAH//KLF7//RPS6KA5//NRXN1//NR4A2//FZD3//ULK2//PPP3CB//HPRT1//FMR1//HECW2//BMPR2//POU3F2//RAPGEF2//LRP8//PLAA//FLRT1//NYAP1//CNTNAP2//GJA1//NTF3// |
| GO:0055074 | calcium ion homeostasis | Biological process | 23 | 431 | 574 | 17653 | 1.64118369887709 | 0.0142089555415093 | 0.249597834616175 | 1.84743784460808 | 0.0400696864111498 | CCR1//GRIK2//PIK3CB//STIM2//CCL8//CALCB//ADCYAP1//EDN1//ESR1//GATA2//GJA1//NPTN//IL2//LRP6//CCL28//PTGFR//C5AR1//NOS1//TRPC3//CALM2//AKAP6//BNIP3//FAM155A// |
| GO:0010243 | response to organonitrogen compound | Biological process | 43 | 937 | 574 | 17653 | 1.41135248903945 | 0.0144650491609087 | 0.252350756858078 | 1.83968008602049 | 0.0749128919860627 | HPRT1//NR4A2//SOD1//APPL1//CEBPB//CPEB3//RRAGD//CPEB1//TNF//LAMTOR3//UBE4B//PPARG//PRKAA1//C5AR1//IGF1//TSC1//CPEB2//INHBB//TRPC3//AGTR2//EDN1//GLRB//CASP3//TMED10//REG3G//EREG//GJA1//LRP6//BTG1//NCK1//FOSL1//PTPN22//GPD1//AKAP6//RAPGEF2//GJB2//PRKAR2B//LDLR//SOCS5//SLIT2//MYD88//KLF3//MGST1// |
| GO:0006464 | cellular protein modification process | Biological process | 159 | 4192 | 574 | 17653 | 1.16649391906269 | 0.0144776125478241 | 0.252350756858078 | 1.83930305021024 | 0.277003484320557 | GADD45A//DIRAS3//CSF2RA//SPRED1//EREG//RASGEF1A//IL2//IL5//MAP3K1//MET//MAP3K7CL//CCL2//BTC//TNF//LAMTOR3//RAPGEF2//CRKL//IGF1//NTF3//PIK3CB//PRKAA1//SOD1//C5AR1//DUSP22//UBE4B//RNF145//ZNRF2//RNF180//RNF125//UBE2W//HECW2//SKP1//THOP1//UBE2G1//LONRF3//CBFB//TNKS//CBL//UBE3B//LONRF1//LRP6//PPP1R15B//SLIT2//EFNA5//FMR1//NPTN//IFNG//SOCS5//RNF38//KLHL3//SPOPL//MDM4//DCAF8//TRIM8//KBTBD8//CDK6//FASTK//PDIK1L//CDK19//PAN3//PPP3CB//CCL8//TGFBR2//RUNX3//ACVR1//RPS6KA5//FLRT1//NCK1//DBNDD2//PTPN22//MTM1//CPPED1//HS3ST5//BTG1//FUT9//FUT2//ST8SIA5//MAN1A1//ST8SIA3//CCDC126//TET2//VEGFB//PIGP//PIGA//MDFIC//CALM2//INHBB//BMP3//BMPR2//PIGS//EPC2//ARID4B//MIER1//TBL1XR1//ARID5B//ESR1//OTUD3//AR//USP28//WDR20//NFATC2IP//VKORC1L1//ATR//STK33//NOS1//EGLN3//GPHN//EGLN1//DCUN1D4//PAK6//JARID2//SOX4//RAP2C//PHACTR2//AGTR2//AKAP6//IFNA1//PRKAR2B//ESCO2//GATA2//PTPRG//PTP4A1//CDC14A//PCGF5//YES1//CNOT7//EDN1//UBXN2B//TSC1//ARSK//CASP3//CCNY//ADCYAP1//GRIK2//ZMYND11//WNT7A//ULK2//LRP8//SAMSN1//ARRDC3//MYB//STRAP//PMEPA1//LDLRAD4//ATG16L1//CCR1//GPR183//NPNT//TNFAIP8L3//KDM2A//RCOR1//CDC27//UBA5//ACSL1//GLRX//PLAA//NCOA7//NMI//RAB3GAP1// |
| GO:0036211 | protein modification process | Biological process | 159 | 4192 | 574 | 17653 | 1.16649391906269 | 0.0144776125478241 | 0.252350756858078 | 1.83930305021024 | 0.277003484320557 | GADD45A//DIRAS3//CSF2RA//SPRED1//EREG//RASGEF1A//IL2//IL5//MAP3K1//MET//MAP3K7CL//CCL2//BTC//TNF//LAMTOR3//RAPGEF2//CRKL//IGF1//NTF3//PIK3CB//PRKAA1//SOD1//C5AR1//DUSP22//UBE4B//RNF145//ZNRF2//RNF180//RNF125//UBE2W//HECW2//SKP1//THOP1//UBE2G1//LONRF3//CBFB//TNKS//CBL//UBE3B//LONRF1//LRP6//PPP1R15B//SLIT2//EFNA5//FMR1//NPTN//IFNG//SOCS5//RNF38//KLHL3//SPOPL//MDM4//DCAF8//TRIM8//KBTBD8//YES1//CDK6//FASTK//PDIK1L//CDK19//PAN3//PPP3CB//CCL8//TGFBR2//RUNX3//ACVR1//RPS6KA5//FLRT1//NCK1//DBNDD2//PTPN22//MTM1//CPPED1//HS3ST5//BTG1//FUT9//FUT2//ST8SIA5//MAN1A1//ST8SIA3//CCDC126//TET2//VEGFB//PIGP//PIGA//MDFIC//CALM2//INHBB//BMP3//BMPR2//PIGS//EPC2//ARID4B//MIER1//TBL1XR1//ARID5B//ESR1//OTUD3//AR//USP28//WDR20//NFATC2IP//VKORC1L1//ATR//STK33//NOS1//EGLN3//GPHN//EGLN1//DCUN1D4//PAK6//JARID2//SOX4//RAP2C//PHACTR2//AGTR2//AKAP6//IFNA1//PRKAR2B//ESCO2//GATA2//PTPRG//PTP4A1//CDC14A//PCGF5//CNOT7//EDN1//UBXN2B//TSC1//ARSK//CASP3//CCNY//ADCYAP1//GRIK2//ZMYND11//WNT7A//ULK2//LRP8//SAMSN1//ARRDC3//MYB//STRAP//PMEPA1//LDLRAD4//ATG16L1//CCR1//GPR183//NPNT//TNFAIP8L3//KDM2A//RCOR1//CDC27//UBA5//ACSL1//GLRX//PLAA//NCOA7//NMI//RAB3GAP1// |
| GO:0009611 | response to wounding | Biological process | 32 | 655 | 574 | 17653 | 1.50250285927069 | 0.0147041544465031 | 0.255640608024937 | 1.83255994428007 | 0.0557491289198606 | PROCR//ENPP4//MMRN1//RCOR1//ZFPM2//GATA2//IFNA1//IRF1//IRF2//NFE2//PRKAR2B//IGF1//WNT7A//DGKH//PIK3CB//TRPC3//FZD6//DGKE//EDN1//ELK3//EREG//TGFBR2//CASP3//MTPN//GJA1//MYOG//SOD1//REG3G//CLASP2//CCR1//INHBB//AIMP1// |
| GO:1903532 | positive regulation of secretion by cell | Biological process | 20 | 361 | 574 | 17653 | 1.70384240447074 | 0.0148036128918283 | 0.256286924933408 | 1.82963228004416 | 0.0348432055749129 | INHBB//EDN1//GJA1//SOX4//PPP3CB//SYBU//GATA2//CLASP2//IGF1//AGTR2//TNF//IL2//IL5//ADCYAP1//RAB3GAP1//AIMP1//PTPN22//IFNG//IL1RAP//MYB// |
| GO:0010942 | positive regulation of cell death | Biological process | 33 | 681 | 574 | 17653 | 1.49029916038619 | 0.0149052817218851 | 0.256286924933408 | 1.82665981103666 | 0.0574912891986063 | ANP32B//EGLN3//PPARG//TNF//CASP3//BNIP3//PIK3CB//IFNG//ZNF268//GADD45A//CDK19//FRZB//NEUROD1//ARHGEF4//POU4F1//BCL2A1//ZMAT3//SOD1//SOS2//SOX4//FOSL1//SLIT2//GRIK2//MYB//DYNLL2//SERINC3//NCK1//BTG1//RAPGEF2//INHBB//AGTR2//IL20RA//ST18// |
| GO:0000381 | regulation of alternative mRNA splicing, via spliceosome | Biological process | 5 | 45 | 574 | 17653 | 3.41715060007743 | 0.014943945850958 | 0.256286924933408 | 1.82553471476714 | 0.00871080139372822 | FMR1//MBNL1//SMU1//RBM25//SRSF2// |
| GO:2000514 | regulation of CD4-positive, alpha-beta T cell activation | Biological process | 5 | 45 | 574 | 17653 | 3.41715060007743 | 0.014943945850958 | 0.256286924933408 | 1.82553471476714 | 0.00871080139372822 | IFNG//MYB//SOCS5//IL2//TGFBR2// |
| GO:0000077 | DNA damage checkpoint | Biological process | 11 | 159 | 574 | 17653 | 2.12765980759538 | 0.0149518390355088 | 0.256286924933408 | 1.8253053870149 | 0.0191637630662021 | GADD45A//CNOT6L//CNOT7//MDM4//CNOT6//SOX4//RBBP8//RAD9B//INTS7//ATR//USP28// |
| GO:0035239 | tube morphogenesis | Biological process | 41 | 887 | 574 | 17653 | 1.42156546947979 | 0.0149951416925778 | 0.256286924933408 | 1.8240494261356 | 0.0714285714285714 | ELK3//EREG//HOXA3//MEOX2//CCL2//VEGFB//WNT7A//MMRN2//AIMP1//EDN1//TGFBR2//ACVR1//ZFPM2//QKI//NPNT//SOX4//LRP6//TSC1//FZD3//FZD6//GJA1//DLL1//SLIT2//SPRED1//SULF1//PPARG//EGLN1//GATA2//BTG1//C5AR1//ADAM12//MET//PIK3CB//DLG5//AR//ESR1//TNF//BMPR2//KLHL3//AGTR2//RAPGEF2// |
| GO:0030003 | cellular cation homeostasis | Biological process | 31 | 631 | 574 | 17653 | 1.5109112795905 | 0.0150672439826446 | 0.256286924933408 | 1.82196617917774 | 0.054006968641115 | CCR1//GRIK2//PIK3CB//STIM2//CCL8//CALCB//EGLN1//SKP1//SOD1//SLC30A5//AGTR2//ADCYAP1//EDN1//ESR1//GATA2//GJA1//NPTN//IL2//LRP6//CCL28//PTGFR//C5AR1//NOS1//TRPC3//CALM2//AKAP6//SLC41A1//SLC30A7//SLC4A4//BNIP3//FAM155A// |
| GO:0042493 | response to drug | Biological process | 44 | 966 | 574 | 17653 | 1.40081950065286 | 0.0151512886383857 | 0.256286924933408 | 1.81955042823495 | 0.0766550522648084 | HPRT1//NR4A2//SOD1//ARL6IP1//SLC1A1//CHRM2//EDN1//GJA1//PPARG//PRKAA1//GJB2//FOSL1//TRPC3//CASP3//PPP3CB//CD69//PPP1R15B//NOS1//ADCYAP1//IL2//CBL//SLIT2//KCNJ10//MYB//BNIP3//PTPN22//TNF//FIBIN//AGTR2//EGLN1//FMR1//PRKAR2B//MET//EFNA5//EMX2//AK4//ACSL1//MGST1//NEUROD1//ATR//TGFBR2//VEGFB//LRP8//FZD3// |
| GO:0033036 | macromolecule localization | Biological process | 116 | 2955 | 574 | 17653 | 1.20727757241314 | 0.0151710464970322 | 0.256286924933408 | 1.81898446059687 | 0.202090592334495 | IFNG//TNF//AGTR2//HEATR5A//ZFAND6//SRSF2//AGFG1//TSC1//PAN3//AKAP6//ARL6IP1//MTX3//IMMP1L//MID1IP1//STX6//TMED10//RAB12//STX12//SNX5//ARFIP1//RAB30//GRIK2//RAB9B//RAB6B//KIF13A//SNX2//EVI5//RAB28//USP6NL//G3BP2//RAPGEF4//NEUROD1//PPP3CB//RFX3//SNAP25//PPARG//VPS37A//DENND1B//EPS15//MTM1//CHMP3//ATG16L1//RP2//ZMAT3//BTF3//PLEKHF2//TNKS//C5ORF30//SLC10A2//TNFAIP8L3//LDLR//GNPTG//PEX5L//ADCYAP1//GJA1//SOX4//EDN1//SYCP1//IGF1//MZT1//ARHGAP1//MTBP//SPO11//FRMD6//ARMCX3//RRAGD//LAMTOR3//MPP4//SYBU//MDFIC//C16ORF70//BLZF1//DLG2//ACSL1//CLIP1//WNT7A//DLG5//NPNT//AKAP4//INHBB//ANP32B//PLA2G12A//IL2//IL5//FMR1//QKI//SKP1//EFNA5//NRXN1//ATR//ESCO2//ESR1//CNTNAP2//GPHN//IL1RAP//CLASP2//EFR3A//LRP6//RAPGEF2//MIEF2//RAB3GAP1//GLRB//PTPN22//CD2AP//TRIM8//DYNLL2//APPL1//AR//ARL5A//NPTN//PRKAA1//SNIP1//CCT6A//DCP2//TGFBR2//MYB// |
| GO:0006977 | DNA damage response, signal transduction by p53 class mediator resulting in cell cycle arrest | Biological process | 6 | 62 | 574 | 17653 | 2.97622794200292 | 0.0151809707550474 | 0.256286924933408 | 1.81870045636672 | 0.0104529616724739 | GADD45A//CNOT6L//CNOT7//MDM4//CNOT6//SOX4// |
| GO:0000288 | nuclear-transcribed mRNA catabolic process, deadenylation-dependent decay | Biological process | 7 | 80 | 574 | 17653 | 2.69100609756098 | 0.0152271956025091 | 0.256286924933408 | 1.81738007345333 | 0.0121951219512195 | CNOT6L//PAN3//CNOT7//CNOT6//DCP2//DDX6//CPEB3// |
| GO:0048477 | oogenesis | Biological process | 7 | 80 | 574 | 17653 | 2.69100609756098 | 0.0152271956025091 | 0.256286924933408 | 1.81738007345333 | 0.0121951219512195 | EREG//YTHDF2//TDRD5//INHBB//IGF1//SPO11//PAQR5// |
| GO:0009894 | regulation of catabolic process | Biological process | 42 | 914 | 574 | 17653 | 1.41321983241714 | 0.0152339706969141 | 0.256286924933408 | 1.81718688412275 | 0.0731707317073171 | DRAM1//RRAGD//ULK2//PTPN22//MET//MTMR9//IFNG//PIK3CB//PRKAA1//BNIP3//TRIM8//LDLR//RAB12//TSC1//SNX5//CASP3//LAMTOR3//EGLN1//MTM1//RNF180//RNF125//LONRF3//LONRF1//SOCS5//MDM4//DCP2//FMR1//YTHDF2//GJA1//HECW2//TNF//MYOG//GPD1//IGF1//SH3D19//CPEB3//CNOT7//CNOT6L//QKI//MYD88//CALCOCO2//RAB3GAP1// |
| GO:0002521 | leukocyte differentiation | Biological process | 25 | 483 | 574 | 17653 | 1.59184034165098 | 0.0154264433290114 | 0.258409173130128 | 1.81173419193002 | 0.0435540069686411 | GPR183//DLL1//ITM2A//CBFB//IFNA1//PTPN22//IL2//PPP3CB//SOX4//PPARG//TNF//IFNG//SOD1//GATA2//TGFBR2//IRF1//TSC1//CD8A//MYB//SOCS5//IL5//CDK6//CEBPB//CCR1//POU4F1// |
| GO:1901360 | organic cyclic compound metabolic process | Biological process | 233 | 6394 | 574 | 17653 | 1.12070140887744 | 0.0154365379187597 | 0.258409173130128 | 1.81145009592867 | 0.405923344947735 | RAD9B//IGF1//NFIA//ATR//WRNIP1//RBBP8//RRM2//DTD1//NPAT//TSHZ1//ZBTB18//CEBPB//ZNF268//TCERG1//STRAP//KLF12//ARX//EDN1//CC2D1B//EN1//ESR1//CPEB3//MYT1L//RCOR1//ZFPM2//GATA2//CNOT7//HOXC8//HSBP1//IFNG//IRF2//AR//JARID2//ZNF568//MAF//MDM4//MYB//NR4A2//KLF3//POU4F1//PPARG//DUSP22//ZBTB4//TNF//ZNF217//LRP8//BHLHE41//TBL1XR1//ARID5B//BHLHE40//RUNX3//PHF14//DCP2//CNOT6L//PAN3//CNOT6//FMR1//MBNL1//SMU1//RBM25//SRSF2//POLR2K//RBM41//SNRPN//NOB1//SPO11//SYCP1//TEX11//PTGES3//NABP1//ELK3//TFEC//FOXJ3//FOXF2//PTF1A//HIVEP2//MACC1//IRF1//MEOX2//MYBL1//NEUROD1//NHLH2//OTX2//POU3F2//RFX4//BMPR2//SOX4//BTF3//CARF//FOSL1//KLF7//CBFB//NMI//SLC35D1//COX8C//GPD1//GPD2//DPYSL2//HPRT1//AK4//CMPK1//IMPDH1//TET2//DCTPP1//GMNC//ESCO2//GADD45A//EPC2//UBE2W//USP28//UNG//KDM2A//MTRR//CASP3//BAHD1//ARID4B//DIRAS3//ZMYND11//KLF17//NCOA7//ZNF782//ZNF800//ZFP30//HBP1//ATAD2//MDFIC//HOXA3//HOXB6//HOXD1//ZNF680//NEUROG1//NFE2//COMMD2//VGLL1//BRWD1//PRKAA1//TRERF1//SLC2A4RG//ZNF630//ZBTB26//MIER1//RFX3//ATXN1//DMRTA1//SMARCD2//ZNF3//ZNF131//ZNF134//ZNF208//ZNF227//ZNF655//MED28//TSC22D4//PCGF5//LCOR//ST18//NRBF2//MLLT6//PAK6//BTG1//RHOXF2B//SNIP1//CBL//RPS6KA5//MED6//EMX2//BOLA3//NR3C2//RFX7//RHOXF2//BLZF1//ELAVL4//RTCA//SCAF8//CPEB1//QKI//AIMP1//MTPN//ACBD3//MSMO1//HSD11B1//MDH1//GPHN//G3BP2//LDLR//EREG//DDX6//GLRX//INTS7//AGTR2//IL5//LRP6//NOS1//PPP3CB//WNT7A//ACVR1//RASD1//BTAF1//GSTM3//BMP3//SULF1//VWC2//CALM2//EGLN1//TNKS//CCT6A//RNF180//MYOG//C5AR1//TDRD5//FZD6//FASTK//YTHDF2//SOD1//ADCYAP1//DLL1//IL2//MET//NCK1//ARMCX3//YES1//NFATC2IP//DLG2//ENPP4//RAPGEF2//SULT6B1//TRIM8//IL1RAP//MYD88//EGLN3//RAP2C//GJA1//NPNT// |
| GO:0021696 | cerebellar cortex morphogenesis | Biological process | 4 | 30 | 574 | 17653 | 4.10058072009291 | 0.015501753499338 | 0.25852380374734 | 1.80961917331268 | 0.00696864111498258 | DLL1//MTPN//WNT7A//RFX4// |
| GO:0009605 | response to external stimulus | Biological process | 93 | 2306 | 574 | 17653 | 1.24031008337589 | 0.0155524281304342 | 0.25852380374734 | 1.80820179690559 | 0.162020905923345 | GLRB//TBL1XR1//CCR1//GPR183//CCL2//CCL8//PPARG//SLIT2//PGC//ADCYAP1//TNF//REG3G//CEBPB//CDK6//IFI44//CHRM2//IFNG//FOSL1//PIK3CB//PLP2//CCL28//C5AR1//AIMP1//CLCN6//TGFBR2//ARX//DOK1//DPYSL2//EFNA5//OTX2//ENAH//KLF7//RPS6KA5//NRXN1//ACSL1//CDS1//TRPC3//STRBP//MYD88//DSC2//INHBB//RRAGD//BMPR2//PLEKHF2//SERINC3//NCK1//MTPN//GADD45A//GJA1//IRF1//MAP3K1//BNIP3//ENPP4//EDN1//NEUROG1//PTPN22//IGF1//NR4A2//PRKAA1//GJB2//MGST1//NOS1//PTGFR//CASP3//SRSF2//FZD3//RNF125//DCTPP1//CNTNAP2//CBL//ULK2//BHLHE40//ESR1//IL2//SOCS5//CDK19//LDLR//MET//NTF3//VEGFB//ZMYND11//CNOT7//IFNA1//IRF2//PLAA//SOD1//TSC1//NPNT//FMR1//CALCOCO2//ATG16L1//C5ORF30//CLASP2// |
| GO:0030855 | epithelial cell differentiation | Biological process | 36 | 760 | 574 | 17653 | 1.45678525582248 | 0.0156038962695906 | 0.25852380374734 | 1.80676694542088 | 0.0627177700348432 | MET//ESR1//GJA1//RFX3//CDK6//DLL1//NEUROD1//AR//FRMD6//GSTM3//WNT7A//EREG//CASP3//IFNG//DLG5//DSC2//KRT27//KRTAP4-11//BTG1//CBFB//REG3G//SOD1//SOX4//CEBPB//BMPR2//RAPGEF2//ACVR1//MAF//TMOD1//FRZB//ARX//RAP2C//ARID4B//TNF//GSTA2//PPARG// |
| GO:0070997 | neuron death | Biological process | 18 | 316 | 574 | 17653 | 1.75183037092577 | 0.0156101904328121 | 0.25852380374734 | 1.80659179883284 | 0.0313588850174216 | EGLN3//CEBPB//EN1//GRIK2//NTF3//NR4A2//POU4F1//CCL2//SOD1//C5AR1//MYB//CASP3//BNIP3//EGLN1//TSC1//CBL//NCOA7//MEOX2// |
| GO:0030217 | T cell differentiation | Biological process | 14 | 225 | 574 | 17653 | 1.91360433604336 | 0.0156345165951001 | 0.25852380374734 | 1.80591554220014 | 0.024390243902439 | IFNG//SOD1//IRF1//TSC1//CD8A//IL2//CBFB//MYB//SOCS5//TGFBR2//GPR183//PTPN22//PPP3CB//SOX4// |
| GO:0001933 | negative regulation of protein phosphorylation | Biological process | 23 | 435 | 574 | 17653 | 1.6260923545196 | 0.0156844809555548 | 0.258717426103456 | 1.80452984877805 | 0.0400696864111498 | SPRED1//DUSP22//CBL//SOCS5//GADD45A//FLRT1//LRP6//NCK1//DBNDD2//CALM2//CDK6//PTPN22//CASP3//ZMYND11//NTF3//SAMSN1//STRAP//PMEPA1//LDLRAD4//PPP1R15B//PRKAR2B//IL2//SLIT2// |
| GO:0042127 | regulation of cell proliferation | Biological process | 70 | 1668 | 574 | 17653 | 1.29065040650406 | 0.0157242218225479 | 0.258741878797789 | 1.80343083819022 | 0.121951219512195 | SULF1//GJA1//TNF//AIMP1//BMPR2//VEGFB//FZD3//TGFBR2//ZNF268//ADCYAP1//REG3G//CRKL//AGTR2//EDN1//EREG//CNOT6L//DLL1//CNOT7//HOXA3//IFNG//IGF1//IL2//AR//NTF3//POU3F2//PRKAA1//SPHK2//PTGFR//CNOT6//SOX4//BTC//FOSL1//CDK6//FRZB//TES//MTBP//IRF1//MDM4//MYOG//BTG1//TSC1//DLG5//RAPGEF2//ST18//CASP3//GPR183//IL5//PTPN22//NCK1//CEBPB//LRP6//ESR1//MYB//OGN//PPARG//C5AR1//STRAP//RUNX3//JARID2//ZFPM2//WNT7A//GATA2//CCL8//PHF14//CFDP1//EGLN3//ARX//EPS15//DUSP22//YES1// |
| GO:0034641 | cellular nitrogen compound metabolic process | Biological process | 248 | 6851 | 574 | 17653 | 1.11327983350939 | 0.0160142374813096 | 0.262782957494122 | 1.79549373546057 | 0.43205574912892 | RAD9B//IGF1//NFIA//ATR//WRNIP1//RBBP8//RRM2//DTD1//NPAT//TSHZ1//ZBTB18//CEBPB//ZNF268//TCERG1//STRAP//KLF12//ARX//EDN1//CC2D1B//EN1//ESR1//CPEB3//MYT1L//RCOR1//ZFPM2//GATA2//CNOT7//HOXC8//HSBP1//IFNG//IRF2//AR//JARID2//ZNF568//MAF//MDM4//MYB//NR4A2//KLF3//POU4F1//PPARG//DUSP22//ZBTB4//TNF//ZNF217//LRP8//BHLHE41//TBL1XR1//ARID5B//BHLHE40//RUNX3//PHF14//DCP2//CNOT6L//PAN3//CNOT6//FMR1//MBNL1//SMU1//RBM25//SRSF2//POLR2K//RBM41//SNRPN//NOB1//SPO11//SYCP1//TEX11//PTGES3//NABP1//SECISBP2L//ST8SIA3//ELK3//TFEC//FOXJ3//FOXF2//PTF1A//HIVEP2//MACC1//IRF1//MEOX2//MYBL1//NEUROD1//NHLH2//OTX2//POU3F2//RFX4//BMPR2//SOX4//BTF3//CARF//FOSL1//KLF7//CBFB//NMI//SLC35D1//COX8C//GPD1//GPD2//GSTA2//GSTM3//MGST1//SOD1//DPYSL2//HPRT1//AK4//CMPK1//IMPDH1//TET2//DCTPP1//GMNC//ESCO2//GADD45A//EPC2//UBE2W//USP28//UNG//KDM2A//MTRR//CASP3//BAHD1//ARID4B//DIRAS3//ZMYND11//KLF17//NCOA7//ZNF782//ZNF800//ZFP30//HBP1//ATAD2//MDFIC//HOXA3//HOXB6//HOXD1//ZNF680//NEUROG1//NFE2//COMMD2//VGLL1//BRWD1//PRKAA1//TRERF1//SLC2A4RG//ZNF630//ZBTB26//MIER1//RFX3//ATXN1//DMRTA1//SMARCD2//ZNF3//ZNF131//ZNF134//ZNF208//ZNF227//ZNF655//MED28//TSC22D4//PCGF5//LCOR//ST18//NRBF2//MLLT6//PAK6//BTG1//RHOXF2B//SNIP1//CBL//RPS6KA5//MED6//EMX2//BOLA3//NR3C2//RFX7//RHOXF2//BLZF1//ELAVL4//RTCA//SCAF8//CPEB1//QKI//CPEB2//MRPL35//METTL17//EIF3B//MTPN//DDX6//TSC1//KBTBD8//AIMP1//IMMP1L//NLN//THOP1//NEU1//MDH1//GGT6//NOS1//G3BP2//EREG//GLRX//PRR16//INTS7//AGTR2//IL5//LRP6//PPP3CB//WNT7A//ACVR1//RASD1//BTAF1//BMP3//SULF1//VWC2//CALM2//EGLN1//TNKS//CCT6A//GPHN//NCK1//SLC44A1//RNF180//MYOG//C5AR1//TDRD5//FZD6//FASTK//YTHDF2//ADCYAP1//DLL1//IL2//MET//ARMCX3//YES1//NFATC2IP//DLG2//ENPP4//SPHK2//SULT6B1//TRIM8//IL1RAP//MYD88//EGLN3//TMED10//RAP2C//PPP1R15B//GJA1//NPNT// |
| GO:0048839 | inner ear development | Biological process | 12 | 182 | 574 | 17653 | 2.02775969674924 | 0.0160663108852654 | 0.262782957494122 | 1.79408383363497 | 0.0209059233449477 | LRIG1//GATA2//NEUROG1//FZD3//FZD6//DLL1//SOD1//FRZB//ANP32B//GJB2//MAF//NEUROD1// |
| GO:1901888 | regulation of cell junction assembly | Biological process | 7 | 81 | 574 | 17653 | 2.65778380006022 | 0.0162163153731057 | 0.262782957494122 | 1.79004781812192 | 0.0121951219512195 | EFNA5//TSC1//CLASP2//TNF//GJA1//CBFB//RAPGEF2// |
| GO:0050868 | negative regulation of T cell activation | Biological process | 8 | 100 | 574 | 17653 | 2.46034843205575 | 0.0162254344815445 | 0.262782957494122 | 1.7898036649183 | 0.0139372822299652 | CEBPB//DLG5//IRF1//SOCS5//CASP3//IL2//PTPN22//DUSP22// |
| GO:0048806 | genitalia development | Biological process | 5 | 46 | 574 | 17653 | 3.34286471746705 | 0.0163309251641215 | 0.262782957494122 | 1.78698921132631 | 0.00871080139372822 | NEUROG1//LRP6//AR//ESR1//FOXF2// |
| GO:0042246 | tissue regeneration | Biological process | 6 | 63 | 574 | 17653 | 2.9289862286378 | 0.0163326317212644 | 0.262782957494122 | 1.78694383057653 | 0.0104529616724739 | IGF1//WNT7A//MTPN//GJA1//MYOG//TGFBR2// |
| GO:0071230 | cellular response to amino acid stimulus | Biological process | 6 | 63 | 574 | 17653 | 2.9289862286378 | 0.0163326317212644 | 0.262782957494122 | 1.78694383057653 | 0.0104529616724739 | RRAGD//CEBPB//CPEB3//CPEB1//TNF//LAMTOR3// |
| GO:0072431 | signal transduction involved in mitotic G1 DNA damage checkpoint | Biological process | 6 | 63 | 574 | 17653 | 2.9289862286378 | 0.0163326317212644 | 0.262782957494122 | 1.78694383057653 | 0.0104529616724739 | GADD45A//CNOT6L//CNOT7//MDM4//CNOT6//SOX4// |
| GO:1902400 | intracellular signal transduction involved in G1 DNA damage checkpoint | Biological process | 6 | 63 | 574 | 17653 | 2.9289862286378 | 0.0163326317212644 | 0.262782957494122 | 1.78694383057653 | 0.0104529616724739 | GADD45A//CNOT6L//CNOT7//MDM4//CNOT6//SOX4// |
| GO:0045727 | positive regulation of translation | Biological process | 9 | 120 | 574 | 17653 | 2.30657665505226 | 0.0164497675960507 | 0.262782957494122 | 1.78384023342621 | 0.0156794425087108 | NCK1//TNF//PPARG//YTHDF2//ESR1//CPEB3//FMR1//PRR16//SOX4// |
| GO:0009914 | hormone transport | Biological process | 18 | 318 | 574 | 17653 | 1.74081256985077 | 0.0165384524588395 | 0.262782957494122 | 1.78150513081914 | 0.0313588850174216 | AGTR2//RAPGEF4//IFNG//NEUROD1//PPP3CB//RFX3//SNAP25//TNF//INHBB//EDN1//ADCYAP1//GJA1//SOX4//SYBU//EFNA5//NRXN1//AIMP1//MYB// |
| GO:0006349 | regulation of gene expression by genetic imprinting | Biological process | 3 | 17 | 574 | 17653 | 5.42723918835827 | 0.0165526452598693 | 0.262782957494122 | 1.78113259222279 | 0.00522648083623693 | PCGF5//ARID4B//DIRAS3// |
| GO:0051023 | regulation of immunoglobulin secretion | Biological process | 3 | 17 | 574 | 17653 | 5.42723918835827 | 0.0165526452598693 | 0.262782957494122 | 1.78113259222279 | 0.00522648083623693 | IL2//IL5//TNF// |
| GO:1902914 | regulation of protein polyubiquitination | Biological process | 3 | 17 | 574 | 17653 | 5.42723918835827 | 0.0165526452598693 | 0.262782957494122 | 1.78113259222279 | 0.00522648083623693 | PLAA//PTPN22//NMI// |
| GO:2000269 | regulation of fibroblast apoptotic process | Biological process | 3 | 17 | 574 | 17653 | 5.42723918835827 | 0.0165526452598693 | 0.262782957494122 | 1.78113259222279 | 0.00522648083623693 | CFDP1//BTG1//IER3IP1// |
| GO:0007204 | positive regulation of cytosolic calcium ion concentration | Biological process | 17 | 295 | 574 | 17653 | 1.77228488749779 | 0.0166531613067152 | 0.263336061714408 | 1.77850331124236 | 0.029616724738676 | NOS1//TRPC3//CALM2//AKAP6//EDN1//FAM155A//ADCYAP1//CCR1//ESR1//GATA2//GJA1//NPTN//IL2//LRP6//CCL28//PTGFR//C5AR1// |
| GO:0044089 | positive regulation of cellular component biogenesis | Biological process | 26 | 511 | 574 | 17653 | 1.56480086187499 | 0.0166653606999507 | 0.263336061714408 | 1.77818528216529 | 0.0452961672473868 | CNOT6L//PAN3//CNOT6//NCK1//MET//CLIP1//IFNG//TNF//MIEF2//IQGAP2//ARFIP1//ESR1//FMR1//TSC1//EFNA5//FLRT1//IL1RAP//WNT7A//DLG5//NRXN1//EDN1//IL5//CLASP2//ATR//FOSL1//RAB3GAP1// |
| GO:0023014 | signal transduction by protein phosphorylation | Biological process | 44 | 973 | 574 | 17653 | 1.39074166251867 | 0.0169543961969028 | 0.266104858308459 | 1.77071767229832 | 0.0766550522648084 | CSF2RA//SPRED1//EREG//RASGEF1A//IL2//IL5//MAP3K1//MET//MAP3K7CL//CCL2//BTC//TNF//LAMTOR3//RAPGEF2//GADD45A//CRKL//IGF1//NTF3//PIK3CB//PRKAA1//SOD1//C5AR1//DUSP22//MDFIC//EDN1//INHBB//PAK6//BMP3//AR//PTPN22//GRIK2//ZMYND11//WNT7A//SKP1//ADCYAP1//CCR1//GPR183//NPNT//NPTN//TNFAIP8L3//CCL8//BMPR2//TGFBR2//ACVR1// |
| GO:1901699 | cellular response to nitrogen compound | Biological process | 31 | 637 | 574 | 17653 | 1.49667977617206 | 0.0169863155063608 | 0.266104858308459 | 1.76990081352578 | 0.054006968641115 | CHRM2//APPL1//SNIP1//CEBPB//CPEB3//RRAGD//CPEB1//TNF//LAMTOR3//CPEB2//INHBB//PPARG//AGTR2//NCK1//TSC1//IGF1//PTPN22//SOD1//GPD1//AKAP6//RAPGEF2//EDN1//NR4A2//GJB2//PRKAR2B//PRKAA1//SLIT2//FMR1//CASP3//KLF3//ESR1// |
| GO:0071417 | cellular response to organonitrogen compound | Biological process | 27 | 537 | 574 | 17653 | 1.54630837210208 | 0.0171138104898107 | 0.266104858308459 | 1.76665328149042 | 0.0470383275261324 | APPL1//CEBPB//CPEB3//RRAGD//CPEB1//TNF//LAMTOR3//CPEB2//INHBB//PPARG//AGTR2//NCK1//TSC1//IGF1//PTPN22//SOD1//GPD1//AKAP6//RAPGEF2//EDN1//NR4A2//GJB2//PRKAR2B//SLIT2//CASP3//KLF3//PRKAA1// |
| GO:0060419 | heart growth | Biological process | 8 | 101 | 574 | 17653 | 2.43598854658985 | 0.0171378094399039 | 0.266104858308459 | 1.76604469052019 | 0.0139372822299652 | GJA1//JARID2//TGFBR2//ZFPM2//AGTR2//EDN1//IGF1//AKAP6// |
| GO:2000278 | regulation of DNA biosynthetic process | Biological process | 8 | 101 | 574 | 17653 | 2.43598854658985 | 0.0171378094399039 | 0.266104858308459 | 1.76604469052019 | 0.0139372822299652 | TNKS//DCP2//ATR//CCT6A//PTGES3//PPARG//GJA1//BMPR2// |
| GO:0042147 | retrograde transport, endosome to Golgi | Biological process | 7 | 82 | 574 | 17653 | 2.62537180249851 | 0.0172500952635516 | 0.266104858308459 | 1.76320850219531 | 0.0121951219512195 | SNX2//STX6//HEATR5A//SNX5//RAB9B//RAB6B//EVI5// |
| GO:0045666 | positive regulation of neuron differentiation | Biological process | 19 | 343 | 574 | 17653 | 1.70359403094239 | 0.0172566501139719 | 0.266104858308459 | 1.76304350631579 | 0.0331010452961672 | STMN2//ADCYAP1//NPTN//NCK1//RAPGEF2//EFNA5//BMPR2//SLIT2//CPEB3//FMR1//IL2//DLG5//LRP8//IL1RAP//PLAA//GATA2//VWC2//NEUROD1//NEUROG1// |
| GO:0003298 | physiological muscle hypertrophy | Biological process | 4 | 31 | 574 | 17653 | 3.96830392267056 | 0.0173520985530135 | 0.266104858308459 | 1.76064799436705 | 0.00696864111498258 | AGTR2//EDN1//IGF1//AKAP6// |
| GO:0003301 | physiological cardiac muscle hypertrophy | Biological process | 4 | 31 | 574 | 17653 | 3.96830392267056 | 0.0173520985530135 | 0.266104858308459 | 1.76064799436705 | 0.00696864111498258 | AGTR2//EDN1//IGF1//AKAP6// |
| GO:0014002 | astrocyte development | Biological process | 4 | 31 | 574 | 17653 | 3.96830392267056 | 0.0173520985530135 | 0.266104858308459 | 1.76064799436705 | 0.00696864111498258 | LDLR//CDK6//DLL1//POU3F2// |
| GO:0034694 | response to prostaglandin | Biological process | 4 | 31 | 574 | 17653 | 3.96830392267056 | 0.0173520985530135 | 0.266104858308459 | 1.76064799436705 | 0.00696864111498258 | EDN1//PPARG//PRKAA1//PTGFR// |
| GO:0045066 | regulatory T cell differentiation | Biological process | 4 | 31 | 574 | 17653 | 3.96830392267056 | 0.0173520985530135 | 0.266104858308459 | 1.76064799436705 | 0.00696864111498258 | IFNG//IL2//CBFB//IRF1// |
| GO:0061049 | cell growth involved in cardiac muscle cell development | Biological process | 4 | 31 | 574 | 17653 | 3.96830392267056 | 0.0173520985530135 | 0.266104858308459 | 1.76064799436705 | 0.00696864111498258 | EDN1//IGF1//AKAP6//AGTR2// |
| GO:0042221 | response to chemical | Biological process | 174 | 4655 | 574 | 17653 | 1.14957203860822 | 0.0174733809702774 | 0.266616906151804 | 1.75762305410868 | 0.303135888501742 | SOD1//CBFB//HPRT1//NR4A2//ARL6IP1//CCR1//GPR183//CCL2//CCL8//SLIT2//DNAJB4//CALM2//PTGES3//MGST1//NAT1//SLC1A1//PIK3CB//PLP2//CCL28//C5AR1//FOSL1//AIMP1//DUSP22//TGFBR2//CBL//ACVR1//CHRM2//ARX//DOK1//DPYSL2//EFNA5//OTX2//ENAH//KLF7//RPS6KA5//NRXN1//ACSL1//PPARG//APPL1//SPRED1//FLRT1//POLR2K//EMX2//AK4//NEUROD1//ATR//VEGFB//LRP8//FZD3//SLC6A14//GPHN//STEAP2//NOS1//SNRPN//GJA1//IGF1//CASP3//SLC2A5//FIBIN//SLC30A7//SLC30A5//EDN1//SNIP1//TNF//TET2//GJB2//CRKL//CSF2RA//EREG//IFNA1//IL1RAP//IL2//IL5//IL20RA//IL36G//SOCS5//CEBPB//CPEB3//RRAGD//CPEB1//LAMTOR3//UBE4B//BMP3//BMPR2//NPNT//STRAP//PMEPA1//LDLRAD4//SULF1//VWC2//ESR1//AR//MMRN2//PRKAA1//PTPN22//PTGFR//RBBP8//WNT7A//TSC1//CPEB2//INHBB//UBA5//SRSF2//TRPC3//ST18//GATA2//PPP3CB//CALCOCO2//VKORC1L1//XAF1//IRF1//CD69//GSTA2//IFNG//CA1//SYBU//CARF//YES1//BRWD1//AGTR2//NTF3//RAPGEF2//SHISA2//GSTM3//PPP1R15B//GLRB//TMED10//NR3C2//PAQR5//REG3G//LRP6//BTG1//ADCYAP1//NPTN//NCK1//OR13A1//OR6A2//MET//KCNJ10//IRF2//TRIM8//NMI//MYD88//CNOT7//EGLN3//EGLN1//MYB//BNIP3//SKP1//UBE2W//CDK19//PLAA//MYOG//SLC41A1//POU3F2//GPD1//AKAP6//SOX4//POU4F1//RAB12//ZFAND6//PRKAR2B//LDLR//MDM4//RUNX3//FMR1//DLL1//KLF3//NCOA7//STMN2//DYNC1LI2//JARID2//PIGA//ARID5B// |
| GO:0008104 | protein localization | Biological process | 104 | 2628 | 574 | 17653 | 1.21706733701388 | 0.0174797278173717 | 0.266616906151804 | 1.75746533419211 | 0.181184668989547 | IFNG//TNF//AGTR2//ZFAND6//AGFG1//SRSF2//TSC1//PAN3//AKAP6//ARL6IP1//MTX3//IMMP1L//STX6//TMED10//RAB12//STX12//SNX5//ARFIP1//RAB30//GRIK2//RAB9B//RAB6B//KIF13A//SNX2//EVI5//RAB28//USP6NL//G3BP2//RAPGEF4//NEUROD1//PPP3CB//RFX3//SNAP25//VPS37A//DENND1B//EPS15//MTM1//CHMP3//ATG16L1//RP2//ZMAT3//BTF3//PLEKHF2//TNKS//C5ORF30//GNPTG//PEX5L//ADCYAP1//GJA1//SOX4//SYCP1//IGF1//MZT1//ARHGAP1//MTBP//SPO11//FRMD6//ARMCX3//RRAGD//LAMTOR3//MPP4//SYBU//MDFIC//PPARG//C16ORF70//BLZF1//DLG2//CLIP1//WNT7A//DLG5//NPNT//AKAP4//INHBB//ANP32B//IL2//IL5//SKP1//EFNA5//NRXN1//ATR//ESCO2//ESR1//CNTNAP2//GPHN//IL1RAP//CLASP2//EFR3A//LRP6//RAPGEF2//MIEF2//RAB3GAP1//GLRB//PTPN22//CD2AP//TRIM8//DYNLL2//APPL1//AR//ARL5A//NPTN//PRKAA1//SNIP1//CCT6A//HEATR5A// |
| GO:0051347 | positive regulation of transferase activity | Biological process | 32 | 664 | 574 | 17653 | 1.48213760967214 | 0.0175349566388716 | 0.266616906151804 | 1.75609530372158 | 0.0557491289198606 | GADD45A//TNF//CRKL//MAP3K1//MAP3K7CL//LAMTOR3//IGF1//NTF3//PIK3CB//PRKAA1//SOD1//C5AR1//EREG//EFNA5//MDFIC//PAK6//TGFBR2//MET//PRKAR2B//EDN1//TNFAIP8L3//CCNY//ADCYAP1//RAPGEF2//DCUN1D4//ARRDC3//PTGES3//TNKS//LRP8//ACSL1//IFNG//CALM2// |
| GO:0009880 | embryonic pattern specification | Biological process | 6 | 64 | 574 | 17653 | 2.88322081881533 | 0.0175431795412617 | 0.266616906151804 | 1.75589169192303 | 0.0104529616724739 | WNT7A//DLL1//MEOX2//NEUROG1//TDRD5//LRP6// |
| GO:0021695 | cerebellar cortex development | Biological process | 5 | 47 | 574 | 17653 | 3.27173993624435 | 0.0178009063441355 | 0.269323332450533 | 1.74955788475651 | 0.00871080139372822 | DLL1//RFX4//MTPN//WNT7A//AGTR2// |
| GO:0034260 | negative regulation of GTPase activity | Biological process | 5 | 47 | 574 | 17653 | 3.27173993624435 | 0.0178009063441355 | 0.269323332450533 | 1.74955788475651 | 0.00871080139372822 | IQGAP2//ADCYAP1//CPEB2//TSC1//SLIT2// |
| GO:0010660 | regulation of muscle cell apoptotic process | Biological process | 7 | 83 | 574 | 17653 | 2.59374081692624 | 0.0183294966149752 | 0.276701753587226 | 1.73684946195216 | 0.0121951219512195 | ADCYAP1//BNIP3//EDN1//IGF1//LRP6//IFNG//PPARG// |
| GO:0016241 | regulation of macroautophagy | Biological process | 11 | 164 | 574 | 17653 | 2.06279213053455 | 0.0184211087264397 | 0.277465386006485 | 1.73468423411291 | 0.0191637630662021 | RAB12//BNIP3//TSC1//CALCOCO2//MTM1//RAB3GAP1//SNX5//PRKAA1//RRAGD//CASP3//LAMTOR3// |
| GO:0001764 | neuron migration | Biological process | 10 | 143 | 574 | 17653 | 2.15065422382496 | 0.0184997960103948 | 0.278031378707333 | 1.73283306035636 | 0.0174216027874564 | ARX//PLAA//RAPGEF2//DCX//GATA2//GJA1//LRP12//NR4A2//SPOCK1//FZD3// |
| GO:0072413 | signal transduction involved in mitotic cell cycle checkpoint | Biological process | 6 | 65 | 574 | 17653 | 2.83886357544894 | 0.0188139111564825 | 0.280879649340599 | 1.72552091114241 | 0.0104529616724739 | GADD45A//CNOT6L//CNOT7//MDM4//CNOT6//SOX4// |
| GO:1902402 | signal transduction involved in mitotic DNA damage checkpoint | Biological process | 6 | 65 | 574 | 17653 | 2.83886357544894 | 0.0188139111564825 | 0.280879649340599 | 1.72552091114241 | 0.0104529616724739 | GADD45A//CNOT6L//CNOT7//MDM4//CNOT6//SOX4// |
| GO:1902403 | signal transduction involved in mitotic DNA integrity checkpoint | Biological process | 6 | 65 | 574 | 17653 | 2.83886357544894 | 0.0188139111564825 | 0.280879649340599 | 1.72552091114241 | 0.0104529616724739 | GADD45A//CNOT6L//CNOT7//MDM4//CNOT6//SOX4// |
| GO:0048638 | regulation of developmental growth | Biological process | 18 | 323 | 574 | 17653 | 1.71386500684998 | 0.0190473386334092 | 0.283485278688585 | 1.72016569702608 | 0.0313588850174216 | DPYSL2//IGF1//SOD1//POU3F2//TGFBR2//ARX//DLL1//MTM1//ULK2//EFNA5//BMPR2//GJA1//JARID2//ZFPM2//EDN1//AKAP6//PLAA//AR// |
| GO:0009119 | ribonucleoside metabolic process | Biological process | 8 | 103 | 574 | 17653 | 2.38868779811238 | 0.0190722758839725 | 0.283485278688585 | 1.719597479745 | 0.0139372822299652 | HPRT1//IMPDH1//CMPK1//GPHN//MTRR//DLG2//AK4//ENPP4// |
| GO:1903580 | positive regulation of ATP metabolic process | Biological process | 4 | 32 | 574 | 17653 | 3.84429442508711 | 0.019334431107113 | 0.285112203371264 | 1.71366860200707 | 0.00696864111498258 | GPD1//IGF1//PRKAA1//MYOG// |
| GO:0051962 | positive regulation of nervous system development | Biological process | 25 | 493 | 574 | 17653 | 1.55955149090755 | 0.0193473059731501 | 0.285112203371264 | 1.71337950002233 | 0.0435540069686411 | FZD3//STMN2//ADCYAP1//NPTN//NCK1//RAPGEF2//GATA2//VWC2//NEUROD1//NEUROG1//EFNA5//PPARG//BMPR2//SLIT2//FLRT1//IL1RAP//WNT7A//DLG5//NRXN1//MYB//CPEB3//FMR1//IL2//LRP8//PLAA// |
| GO:0002313 | mature B cell differentiation involved in immune response | Biological process | 3 | 18 | 574 | 17653 | 5.12572590011614 | 0.0193925201169276 | 0.285112203371264 | 1.71236574936599 | 0.00522648083623693 | DLL1//ITM2A//GPR183// |
| GO:0045821 | positive regulation of glycolytic process | Biological process | 3 | 18 | 574 | 17653 | 5.12572590011614 | 0.0193925201169276 | 0.285112203371264 | 1.71236574936599 | 0.00522648083623693 | GPD1//IGF1//PRKAA1// |
| GO:0060074 | synapse maturation | Biological process | 3 | 18 | 574 | 17653 | 5.12572590011614 | 0.0193925201169276 | 0.285112203371264 | 1.71236574936599 | 0.00522648083623693 | NRXN1//NFIA//SYBU// |
| GO:0010469 | regulation of signaling receptor activity | Biological process | 27 | 543 | 574 | 17653 | 1.52922209174736 | 0.0194449131563926 | 0.28526235938543 | 1.71119399212998 | 0.0470383275261324 | EREG//CBL//SOCS5//PATE1//ADCYAP1//EDN1//C1QTNF9//IFNA1//IFNG//MACC1//IGF1//IL2//IL5//INHBB//NTF3//OGN//IL36G//CCL28//CCL2//CCL8//BMP3//BTC//TNF//VEGFB//WNT7A//CALCB//AIMP1// |
| GO:0060537 | muscle tissue development | Biological process | 21 | 396 | 574 | 17653 | 1.63091278640059 | 0.0197550851399437 | 0.289185369700085 | 1.70432109426762 | 0.0365853658536585 | ZFPM2//UBE4B//POU4F1//ZBTB18//MEOX2//MYOG//MTPN//GJA1//DLL1//MTM1//PRKAA1//TSC1//EGLN1//JARID2//TGFBR2//ACVR1//AGTR2//EDN1//IGF1//AKAP6//NLN// |
| GO:0006873 | cellular ion homeostasis | Biological process | 31 | 645 | 574 | 17653 | 1.47811630608 | 0.0198441966099568 | 0.289862422620168 | 1.70236647876363 | 0.054006968641115 | CCR1//GRIK2//PIK3CB//STIM2//CCL8//CALCB//EGLN1//SKP1//SOD1//SLC30A5//AGTR2//ADCYAP1//EDN1//ESR1//GATA2//GJA1//NPTN//IL2//LRP6//CCL28//PTGFR//C5AR1//NOS1//TRPC3//CALM2//AKAP6//SLC41A1//SLC30A7//SLC4A4//BNIP3//FAM155A// |
| GO:0070661 | leukocyte proliferation | Biological process | 16 | 278 | 574 | 17653 | 1.77003484320558 | 0.0200046097708177 | 0.291575810086293 | 1.69886991596874 | 0.0278745644599303 | CASP3//GPR183//IL2//IL5//PTPN22//PPP3CB//IFNA1//NCK1//IGF1//CEBPB//DLG5//HPRT1//IMPDH1//CCL8//TGFBR2//IRF1// |
| GO:0006513 | protein monoubiquitination | Biological process | 6 | 66 | 574 | 17653 | 2.79585049097244 | 0.0201460792880863 | 0.291859264428456 | 1.69580946114706 | 0.0104529616724739 | SKP1//PCGF5//UBE4B//UBE2W//KBTBD8//CBL// |
| GO:0010611 | regulation of cardiac muscle hypertrophy | Biological process | 6 | 66 | 574 | 17653 | 2.79585049097244 | 0.0201460792880863 | 0.291859264428456 | 1.69580946114706 | 0.0104529616724739 | MTPN//EDN1//IGF1//JARID2//AKAP6//TRPC3// |
| GO:0046467 | membrane lipid biosynthetic process | Biological process | 10 | 145 | 574 | 17653 | 2.12099002763427 | 0.0201765475631852 | 0.291859264428456 | 1.69515314446831 | 0.0174216027874564 | ST8SIA3//PIGP//PIGA//SPHK2//ST8SIA5//GLT6D1//PIGS//ELOVL7//PRKAA1//TNF// |
| GO:1901215 | negative regulation of neuron death | Biological process | 12 | 188 | 574 | 17653 | 1.96304396174661 | 0.0201966783605674 | 0.291859264428456 | 1.69472005076604 | 0.0209059233449477 | CEBPB//EN1//GRIK2//NTF3//NR4A2//POU4F1//CCL2//SOD1//C5AR1//NCOA7//TSC1//CBL// |
| GO:0008152 | metabolic process | Biological process | 405 | 11746 | 574 | 17653 | 1.06040472818681 | 0.0203975795067158 | 0.293916320889684 | 1.69042136538025 | 0.705574912891986 | UBXN2B//STX12//ATG16L1//ULK2//RAD9B//IGF1//NFIA//ATR//WRNIP1//RBBP8//RRM2//DTD1//GADD45A//DIRAS3//NPAT//MTRR//TSHZ1//ZBTB18//CEBPB//ZNF268//TCERG1//STRAP//KLF12//ARX//EDN1//CC2D1B//EN1//ESR1//CPEB3//MYT1L//RCOR1//ZFPM2//GATA2//CNOT7//HOXC8//HSBP1//IFNG//IRF2//AR//JARID2//ZNF568//MAF//MDM4//MYB//NR4A2//KLF3//POU4F1//PPARG//DUSP22//ZBTB4//TNF//ZNF217//LRP8//BHLHE41//TBL1XR1//ARID5B//BHLHE40//RUNX3//PHF14//CSF2RA//SPRED1//EREG//RASGEF1A//IL2//IL5//MAP3K1//MET//MAP3K7CL//CCL2//BTC//LAMTOR3//RAPGEF2//DCP2//CRKL//NTF3//PIK3CB//PRKAA1//SOD1//C5AR1//UBE4B//RNF145//ZNRF2//RNF180//RNF125//UBE2W//HECW2//SKP1//THOP1//UBE2G1//LONRF3//CBFB//TNKS//CBL//UBE3B//LONRF1//CNOT6L//PAN3//CNOT6//FMR1//MBNL1//SMU1//RBM25//SRSF2//POLR2K//RBM41//SNRPN//BNIP3//NOB1//SPO11//SYCP1//TEX11//PTGES3//NABP1//NOS1//SECISBP2L//MGST1//ADH4//CHST7//CHST1//ST8SIA3//ACSL1//CAMLG//EPS15//LRP6//PPP1R15B//SLIT2//EFNA5//NPTN//ELK3//TFEC//FOXJ3//FOXF2//PTF1A//HIVEP2//MACC1//IRF1//MEOX2//MYBL1//NEUROD1//NHLH2//OTX2//POU3F2//RFX4//BMPR2//SOX4//BTF3//CARF//FOSL1//KLF7//NMI//SOCS5//RNF38//KLHL3//SPOPL//DCAF8//TRIM8//KBTBD8//FUT9//FUT2//ST8SIA5//GLT6D1//SLC2A5//AIMP1//HS3ST5//IDS//SLC35D1//GPD1//GPD2//MDH1//NLN//GPHN//EGLN3//VKORC1L1//KDM2A//MSRB3//STEAP2//GLUD2//HSD11B1//IMPDH1//EGLN1//TET2//RDH14//MSMO1//COX8C//COX7A2//GLRX//GSTA2//GSTM3//DPYSL2//HPRT1//AK4//CMPK1//DCTPP1//GMNC//ESCO2//EPC2//USP28//UNG//CASP3//BAHD1//ARID4B//ZMYND11//KLF17//NCOA7//ZNF782//ZNF800//ZFP30//HBP1//ATAD2//MDFIC//HOXA3//HOXB6//HOXD1//ZNF680//NEUROG1//NFE2//COMMD2//VGLL1//BRWD1//TRERF1//SLC2A4RG//ZNF630//ZBTB26//MIER1//RFX3//ATXN1//DMRTA1//SMARCD2//ZNF3//ZNF131//ZNF134//ZNF208//ZNF227//ZNF655//MED28//TSC22D4//PCGF5//LCOR//ST18//NRBF2//MLLT6//PAK6//BTG1//RHOXF2B//SNIP1//RPS6KA5//MED6//EMX2//BOLA3//NR3C2//RFX7//RHOXF2//BLZF1//ELAVL4//RTCA//SCAF8//CPEB1//QKI//AGFG1//CPEB2//MRPL35//METTL17//EIF3B//MTPN//DDX6//TSC1//YES1//IMMP1L//CDK6//FASTK//PDIK1L//CDK19//PPP3CB//CCL8//TGFBR2//ACVR1//FLRT1//NCK1//DBNDD2//PTPN22//MTM1//CPPED1//MAN1A1//CCDC126//VEGFB//PIGP//PIGA//GGT6//PRSS55//MEP1A//DESI2//PGC//NPEPL1//ADAM12//ADAM18//LMLN//SLC6A14//SLC6A6//LGSN//SERINC3//LDLR//MID1IP1//PRKAR2B//TNFAIP8L3//PLAA//DDHD2//LCLAT1//PLA2G12A//CDS1//ABHD3//SLC44A1//MTMR9//SPHK2//ARSK//NEU1//ACBD3//CA1//NAT1//RAB12//DRAM1//ANP32B//VSIG4//DLL1//G3BP2//DGKE//ACBD5//RRAGD//RAB3GAP1//GJA1//MYD88//PTGFR//CCR1//NPNT//CALM2//INHBB//BMP3//DLG2//PLEKHF2//A2ML1//WFDC13//SPOCK1//NDST3//IAH1//PRR16//INTS7//VPS37A//DYNLL2//CHMP3//EI24//SNX5//PIGS//GNPTG//DGKH//AGTR2//WNT7A//RASD1//BTAF1//OTUD3//WDR20//NFATC2IP//STK33//OGN//ELOVL7//DCUN1D4//SULF1//DSEL//VWC2//CDC27//RAP2C//CCT6A//PHACTR2//AKAP6//IFNA1//PTPRG//PTP4A1//CDC14A//WDFY3//IL1RAP//MYOG//ITM2A//TDRD5//ARL6IP1//CD2AP//FZD6//YTHDF2//CCNY//ADCYAP1//ARMCX3//ENPP4//GRIK2//TMEM86A//EFR3A//SULT6B1//SAMSN1//SH3D19//ARRDC3//PMEPA1//LDLRAD4//GPR183//UBA5//CALCOCO2//SNAP25//TMED10//ENPP5//ABHD11// |
| GO:1903037 | regulation of leukocyte cell-cell adhesion | Biological process | 17 | 302 | 574 | 17653 | 1.73120543646307 | 0.0204259457072529 | 0.293916320889684 | 1.68981782679391 | 0.029616724738676 | YES1//IFNG//IL2//NCK1//IGF1//CEBPB//DLG5//IRF1//MYB//SOCS5//CASP3//PTPN22//DUSP22//CCL2//TGFBR2//CCL28//TNF// |
| GO:1902105 | regulation of leukocyte differentiation | Biological process | 15 | 256 | 574 | 17653 | 1.80201301175958 | 0.0206495135480889 | 0.296502463111943 | 1.68509017480027 | 0.0261324041811847 | IFNG//SOD1//IL2//CBFB//IRF1//MYB//SOCS5//IL5//GATA2//CDK6//CEBPB//CCR1//POU4F1//TNF//TGFBR2// |
| GO:0051716 | cellular response to stimulus | Biological process | 270 | 7556 | 574 | 17653 | 1.09895129144893 | 0.0208276727049515 | 0.296702733483842 | 1.68135925563387 | 0.470383275261324 | INTS7//ATR//USP28//CSF2RA//SPRED1//EREG//RASGEF1A//IL2//IL5//MAP3K1//MET//MAP3K7CL//CCL2//BTC//TNF//LAMTOR3//RAPGEF2//GADD45A//CRKL//IGF1//NTF3//PIK3CB//PRKAA1//SOD1//C5AR1//DUSP22//SPO11//SYCP1//RBBP8//NABP1//WRNIP1//BCL2A1//CBFB//SKP1//RPS6KA5//MYD88//CCR1//GPR183//CCL8//SLIT2//REG3G//SPHK2//APCDD1//CCNY//HBP1//LRP6//WNT7A//TNKS//PPP3CB//FZD3//FZD6//CDK6//CDS1//PTGES3//IQGAP2//ARAP2//CLCN6//DOK1//DPYSL2//ELK3//HTR3D//ESR1//EVI2A//CD2AP//APPL1//OSTF1//GJA1//GLRB//CNOT7//LRP12//HIVEP2//AR//NR3C2//NR4A2//HPCAL4//RASD1//KCNK10//PPARG//PSD//ARRDC3//MIER1//SMOC1//SMOC2//SPOCK1//ZNF217//LRP8//CALCB//RERGL//CAMLG//OR6A2//CBL//AKAP4//DLG5//AIMP1//PLAA//ARHGAP12//CD69//ULK2//PTPRG//YES1//CD8A//AKAP11//DGKH//DCX//AGTR2//ARHGEF4//TREM1//PRKAR2B//STK33//DGKE//AKAP6//BMPR2//SOCS5//RAD9B//EPC2//UBE2W//UNG//POLR2K//ESCO2//KDM2A//MGST1//NAT1//EGLN3//FMR1//ZBTB4//ZMAT3//CASP3//CNOT6L//MDM4//CNOT6//SOX4//PPP1R15B//EDN1//IFNG//EFNA5//NCK1//EPS15//TGFBR2//ACVR1//RAPGEF4//CHRM2//ADCYAP1//FRZB//CCL28//PTGFR//SOS2//OR13A1//CALM2//GRIK2//DLL1//SNIP1//MDFIC//NMI//NEUROD1//NOS1//ARHGAP1//RALGPS2//DIRAS3//G3BP2//PAK6//TRPC3//FLRT1//OTX2//UBE4B//BNIP3//DSC2//INHBB//RRAGD//PLEKHF2//SAMSN1//SH3BGR//SH3BGRL//C1QTNF9//IFNA1//MACC1//OGN//IL36G//BMP3//VEGFB//PMEPA1//LDLRAD4//NYAP1//IL1RAP//PLP2//IL20RA//TNFAIP8L3//RFX4//CEBPB//CPEB3//CPEB1//DENND1B//PTPN22//MTPN//IRF1//SULF1//SHISA2//NPNT//STRAP//VWC2//MMRN2//FOSL1//MTM1//TSC1//AGFG1//RAB12//RAB30//RAB9B//RAB6B//RAB28//RAP2C//CPEB2//UBA5//ST18//ACSL1//VKORC1L1//GJB2//CETN1//TFEC//NRBF2//GSTA2//CA1//SYBU//CARF//BRWD1//RNF125//GSTM3//DCTPP1//ZFAND6//TRIM8//ZMYND11//PAQR5//PEX5L//NPTN//GATA2//YTHDF2//ARID5B//PTF1A//GCSAML//FAM110C//OTUD3//RAB3GAP1//IRF2//XAF1//NRXN1//EGLN1//MYB//HSBP1//FIBIN//CDK19//MYOG//SLC41A1//POU3F2//GPD1//SLC2A5//POU4F1//LDLR//TBL1XR1//FASTK//SIVA1//PATE1//HSPB8//DYNLL2//KLF3//SERINC3//NCOA7//STMN2//DYNC1LI2//JARID2//PIGA//PHF14// |
| GO:0033002 | muscle cell proliferation | Biological process | 13 | 211 | 574 | 17653 | 1.8948181052562 | 0.0208396230101613 | 0.296702733483842 | 1.68111014171069 | 0.0226480836236934 | MYOG//EDN1//EREG//IGF1//MYB//TGFBR2//TNF//IFNG//OGN//PPARG//GJA1//JARID2//ZFPM2// |
| GO:0048588 | developmental cell growth | Biological process | 13 | 211 | 574 | 17653 | 1.8948181052562 | 0.0208396230101613 | 0.296702733483842 | 1.68111014171069 | 0.0226480836236934 | TGFBR2//DPYSL2//EFNA5//ULK2//PPP3CB//BMPR2//SLIT2//AGTR2//EDN1//IGF1//AKAP6//PLAA//FLRT1// |
| GO:0009994 | oocyte differentiation | Biological process | 5 | 49 | 574 | 17653 | 3.13819953068335 | 0.0209970249230176 | 0.296702733483842 | 1.67784223626839 | 0.00871080139372822 | EREG//YTHDF2//TDRD5//INHBB//IGF1// |
| GO:0006470 | protein dephosphorylation | Biological process | 17 | 303 | 574 | 17653 | 1.72549188716781 | 0.0210143363660986 | 0.296702733483842 | 1.67748432048568 | 0.029616724738676 | PHACTR2//AGTR2//CALM2//PPP1R15B//AKAP6//PTPN22//MTM1//DUSP22//PTPRG//PTP4A1//CDC14A//TNF//UBXN2B//TSC1//NCK1//CPPED1//PPP3CB// |
| GO:0016482 | cytosolic transport | Biological process | 10 | 146 | 574 | 17653 | 2.10646269867787 | 0.0210553612566424 | 0.296702733483842 | 1.67663730278704 | 0.0174216027874564 | EPS15//SNX2//STX6//HEATR5A//SNX5//RAB9B//RAB6B//EVI5//SYBU//CHMP3// |
| GO:0045860 | positive regulation of protein kinase activity | Biological process | 26 | 522 | 574 | 17653 | 1.53182613106919 | 0.0211543845671815 | 0.296702733483842 | 1.67459960483824 | 0.0452961672473868 | GADD45A//TNF//CRKL//MAP3K1//MAP3K7CL//LAMTOR3//IGF1//NTF3//PIK3CB//PRKAA1//SOD1//C5AR1//EREG//EFNA5//MDFIC//PAK6//TGFBR2//PRKAR2B//EDN1//CCNY//LRP8//ACSL1//IFNG//CALM2//RAPGEF2//ADCYAP1// |
| GO:0043244 | regulation of protein complex disassembly | Biological process | 8 | 105 | 574 | 17653 | 2.34318898291024 | 0.0211578047361011 | 0.296702733483842 | 1.67452939525955 | 0.0139372822299652 | STMN2//CLASP2//MID1IP1//TNF//BNIP3//TMOD1//CALCOCO2//MTPN// |
| GO:0044255 | cellular lipid metabolic process | Biological process | 48 | 1094 | 574 | 17653 | 1.34936842708725 | 0.0212331403158261 | 0.296702733483842 | 1.67298577027219 | 0.0836236933797909 | PTGES3//EDN1//MGST1//ADH4//LRP8//ST8SIA3//ACSL1//PIGP//PIGA//PRKAR2B//MSMO1//PRKAA1//TNFAIP8L3//PLAA//DDHD2//LCLAT1//GPD1//PLA2G12A//CDS1//ABHD3//SLC44A1//SERINC3//MTM1//PIK3CB//MTMR9//SPHK2//ARSK//NEU1//ST8SIA5//DGKE//ACBD5//GLT6D1//LDLR//PIGS//ELOVL7//PPARG//RDH14//QKI//MID1IP1//DGKH//TMEM86A//EREG//ESR1//EFR3A//MET//BTC//TBL1XR1//TNF// |
| GO:0050896 | response to stimulus | Biological process | 321 | 9123 | 574 | 17653 | 1.08211641824221 | 0.0212647973312203 | 0.296702733483842 | 1.67233875206833 | 0.559233449477352 | INTS7//ATR//USP28//CSF2RA//SPRED1//EREG//RASGEF1A//IL2//IL5//MAP3K1//MET//MAP3K7CL//CCL2//BTC//TNF//LAMTOR3//RAPGEF2//GADD45A//CRKL//IGF1//NTF3//PIK3CB//PRKAA1//SOD1//C5AR1//DUSP22//SPO11//SYCP1//RBBP8//NABP1//WRNIP1//ADCYAP1//GRIK2//EGLN3//MYB//NOS1//NR4A2//EGLN1//BNIP3//TGFBR2//VEGFB//CASP3//BCL2A1//RAET1E//PPP3CB//CBFB//GLRB//HPRT1//TBL1XR1//ARL6IP1//SKP1//RPS6KA5//MYD88//GPR183//IFNA1//IFNG//RNF125//TSC1//CD84//DLL1//ITM2A//CCR1//CD8A//ACVR1//GJA1//CCL8//PPARG//SLIT2//REG3G//PGC//IRF1//SAMSN1//GJB2//SPHK2//DNAJB4//INHBB//CAMLG//CEBPB//CDK6//IFI44//CHRM2//FOSL1//PROCR//ENPP4//MMRN1//RCOR1//ZFPM2//GATA2//IRF2//NFE2//PRKAR2B//APCDD1//CCNY//HBP1//LRP6//WNT7A//TNKS//FZD3//FZD6//CDS1//PTGES3//IQGAP2//ARAP2//CLCN6//DOK1//DPYSL2//ELK3//HTR3D//ESR1//EVI2A//CD2AP//APPL1//OSTF1//CNOT7//LRP12//HIVEP2//AR//NR3C2//HPCAL4//RASD1//KCNK10//PSD//ARRDC3//MIER1//SMOC1//SMOC2//SPOCK1//ZNF217//LRP8//CALCB//RERGL//OR6A2//CBL//AKAP4//DLG5//AIMP1//PLAA//ARHGAP12//CD69//ULK2//PTPRG//YES1//AKAP11//DGKH//DCX//AGTR2//ARHGEF4//TREM1//STK33//DGKE//AKAP6//BMPR2//SOCS5//CALM2//RAD9B//EPC2//UBE2W//UNG//POLR2K//ESCO2//KDM2A//MGST1//NAT1//SLC1A1//PLP2//CCL28//IL1RAP//PXK//IL36G//PTGFR//CHST1//NMI//VSIG4//YTHDF2//FMR1//ZBTB4//ZMAT3//CNOT6L//MDM4//CNOT6//SOX4//MSRB3//BTG1//PPP1R15B//EDN1//EFNA5//NCK1//EPS15//RAPGEF4//FRZB//SOS2//OR13A1//SNIP1//MDFIC//NEUROD1//ARHGAP1//RALGPS2//DIRAS3//G3BP2//PAK6//ARX//OTX2//ENAH//KLF7//NRXN1//ACSL1//TRPC3//STRBP//DDHD2//NPTN//FLRT1//UBE4B//DSC2//RRAGD//PLEKHF2//EMX2//AK4//SLC6A14//GPHN//SERINC3//MYOG//STEAP2//SNRPN//SLC2A5//SH3BGR//SH3BGRL//FIBIN//SLC30A7//SLC30A5//C1QTNF9//MACC1//OGN//BMP3//MTPN//JARID2//PMEPA1//LDLRAD4//NYAP1//TET2//IL20RA//TNFAIP8L3//RFX4//CPEB3//CPEB1//DENND1B//PTPN22//SULF1//SHISA2//NPNT//STRAP//VWC2//MMRN2//NEUROG1//MTM1//AGFG1//RAB12//RAB30//RAB9B//RAB6B//RAB28//RAP2C//CPEB2//UBA5//SRSF2//ST18//CALCOCO2//VKORC1L1//CETN1//TFEC//NRBF2//XAF1//GSTA2//CA1//SYBU//CARF//BRWD1//GSTM3//DCTPP1//CNTNAP2//ZFAND6//TRIM8//ZMYND11//BHLHE40//TMED10//IMPDH1//NEU1//CPPED1//KCMF1//SNAP25//PAQR5//PEX5L//ARID5B//PTF1A//CDK19//LDLR//GCSAML//KCNJ3//FAM110C//OTUD3//KCNJ10//RAB3GAP1//HSBP1//SLC41A1//POU3F2//GPD1//POU4F1//RUNX3//FASTK//SIVA1//ATG16L1//PATE1//C5ORF30//HSPB8//DYNLL2//KLF3//NCOA7//CLASP2//STMN2//DYNC1LI2//PIGA//PHF14// |
| GO:0031334 | positive regulation of protein complex assembly | Biological process | 15 | 257 | 574 | 17653 | 1.79500128797842 | 0.0212997889234439 | 0.296702733483842 | 1.67162470030952 | 0.0261324041811847 | CNOT6L//PAN3//CNOT6//NCK1//MET//CLIP1//MIEF2//IQGAP2//ARFIP1//ESR1//IL5//TNF//ATR//FOSL1//IFNG// |
| GO:0015949 | nucleobase-containing small molecule interconversion | Biological process | 4 | 33 | 574 | 17653 | 3.72780065462992 | 0.0214510749943212 | 0.296702733483842 | 1.66855093879844 | 0.00696864111498258 | AK4//GLRX//CMPK1//RRM2// |
| GO:0043928 | exonucleolytic nuclear-transcribed mRNA catabolic process involved in deadenylation-dependent decay | Biological process | 4 | 33 | 574 | 17653 | 3.72780065462992 | 0.0214510749943212 | 0.296702733483842 | 1.66855093879844 | 0.00696864111498258 | DDX6//DCP2//CNOT7//CNOT6// |
| GO:0048665 | neuron fate specification | Biological process | 4 | 33 | 574 | 17653 | 3.72780065462992 | 0.0214510749943212 | 0.296702733483842 | 1.66855093879844 | 0.00696864111498258 | MYT1L//DLL1//POU3F2//POU4F1// |
| GO:0097120 | receptor localization to synapse | Biological process | 4 | 33 | 574 | 17653 | 3.72780065462992 | 0.0214510749943212 | 0.296702733483842 | 1.66855093879844 | 0.00696864111498258 | SNAP25//GPHN//NPTN//DLG2// |
| GO:0042326 | negative regulation of phosphorylation | Biological process | 24 | 473 | 574 | 17653 | 1.56047469263578 | 0.0214937138419457 | 0.296702733483842 | 1.66768853743032 | 0.0418118466898955 | SPRED1//DUSP22//IL2//LRP6//PPP1R15B//SLIT2//CBL//SOCS5//GADD45A//FLRT1//NCK1//DBNDD2//CALM2//CDK6//PTPN22//CASP3//MYOG//ZMYND11//NTF3//SAMSN1//STRAP//PMEPA1//LDLRAD4//PRKAR2B// |
| GO:0003281 | ventricular septum development | Biological process | 6 | 67 | 574 | 17653 | 2.75412137916688 | 0.0215408904540243 | 0.296702733483842 | 1.66673634786831 | 0.0104529616724739 | TGFBR2//ZFPM2//EGLN1//BMPR2//SOX4//ACVR1// |
| GO:0048663 | neuron fate commitment | Biological process | 6 | 67 | 574 | 17653 | 2.75412137916688 | 0.0215408904540243 | 0.296702733483842 | 1.66673634786831 | 0.0104529616724739 | GATA2//MYT1L//DLL1//POU3F2//POU4F1//PTF1A// |
| GO:0051705 | multi-organism behavior | Biological process | 6 | 67 | 574 | 17653 | 2.75412137916688 | 0.0215408904540243 | 0.296702733483842 | 1.66673634786831 | 0.0104529616724739 | POU4F1//EN1//CNTNAP2//PPP3CB//ATXN1//NRXN1// |
| GO:0051965 | positive regulation of synapse assembly | Biological process | 6 | 67 | 574 | 17653 | 2.75412137916688 | 0.0215408904540243 | 0.296702733483842 | 1.66673634786831 | 0.0104529616724739 | WNT7A//EFNA5//FLRT1//IL1RAP//DLG5//NRXN1// |
| GO:0071704 | organic substance metabolic process | Biological process | 389 | 11249 | 574 | 17653 | 1.06351180112642 | 0.0217568047295554 | 0.297099644789858 | 1.66240488609782 | 0.677700348432056 | RAD9B//IGF1//NFIA//ATR//WRNIP1//RBBP8//RRM2//DTD1//GADD45A//DIRAS3//NPAT//MTRR//TSHZ1//ZBTB18//CEBPB//ZNF268//TCERG1//STRAP//KLF12//ARX//EDN1//CC2D1B//EN1//ESR1//CPEB3//MYT1L//RCOR1//ZFPM2//GATA2//CNOT7//HOXC8//HSBP1//IFNG//IRF2//AR//JARID2//ZNF568//MAF//MDM4//MYB//NR4A2//KLF3//POU4F1//PPARG//DUSP22//ZBTB4//TNF//ZNF217//LRP8//BHLHE41//TBL1XR1//ARID5B//BHLHE40//RUNX3//PHF14//CSF2RA//SPRED1//EREG//RASGEF1A//IL2//IL5//MAP3K1//MET//MAP3K7CL//CCL2//BTC//LAMTOR3//RAPGEF2//DCP2//CRKL//NTF3//PIK3CB//PRKAA1//SOD1//C5AR1//UBE4B//RNF145//ZNRF2//RNF180//RNF125//UBE2W//HECW2//SKP1//THOP1//UBE2G1//LONRF3//CBFB//TNKS//CBL//UBE3B//LONRF1//CNOT6L//PAN3//CNOT6//FMR1//MBNL1//SMU1//RBM25//SRSF2//POLR2K//RBM41//SNRPN//NOB1//SPO11//SYCP1//TEX11//PTGES3//NABP1//SECISBP2L//MGST1//ADH4//ST8SIA3//ACSL1//CAMLG//EPS15//LRP6//PPP1R15B//SLIT2//EFNA5//NPTN//ELK3//TFEC//FOXJ3//FOXF2//PTF1A//HIVEP2//MACC1//IRF1//MEOX2//MYBL1//NEUROD1//NHLH2//OTX2//POU3F2//RFX4//BMPR2//SOX4//BTF3//CARF//FOSL1//KLF7//NMI//SOCS5//RNF38//KLHL3//SPOPL//DCAF8//TRIM8//KBTBD8//FUT9//FUT2//ST8SIA5//GLT6D1//SLC2A5//CHST7//CHST1//AIMP1//HS3ST5//IDS//SLC35D1//GPD1//GPD2//MDH1//NLN//COX8C//GSTA2//GSTM3//DPYSL2//HPRT1//AK4//CMPK1//IMPDH1//TET2//DCTPP1//GMNC//ESCO2//EPC2//USP28//UNG//KDM2A//CASP3//BAHD1//ARID4B//ZMYND11//KLF17//NCOA7//ZNF782//ZNF800//ZFP30//HBP1//ATAD2//MDFIC//HOXA3//HOXB6//HOXD1//ZNF680//NEUROG1//NFE2//COMMD2//VGLL1//BRWD1//TRERF1//SLC2A4RG//ZNF630//ZBTB26//MIER1//RFX3//ATXN1//DMRTA1//SMARCD2//ZNF3//ZNF131//ZNF134//ZNF208//ZNF227//ZNF655//MED28//TSC22D4//PCGF5//LCOR//ST18//NRBF2//MLLT6//PAK6//BTG1//RHOXF2B//SNIP1//RPS6KA5//MED6//EMX2//BOLA3//NR3C2//RFX7//RHOXF2//BLZF1//ELAVL4//RTCA//SCAF8//CPEB1//QKI//AGFG1//CPEB2//MRPL35//METTL17//EIF3B//MTPN//DDX6//TSC1//YES1//IMMP1L//CDK6//FASTK//PDIK1L//CDK19//PPP3CB//CCL8//TGFBR2//ACVR1//FLRT1//NCK1//DBNDD2//PTPN22//MTM1//CPPED1//MAN1A1//CCDC126//VEGFB//PIGP//PIGA//GGT6//PRSS55//MEP1A//DESI2//PGC//NPEPL1//ADAM12//ADAM18//LMLN//SLC6A14//SLC6A6//NOS1//GLUD2//LGSN//SERINC3//LDLR//MID1IP1//MSMO1//PRKAR2B//TNFAIP8L3//PLAA//DDHD2//LCLAT1//PLA2G12A//CDS1//ABHD3//SLC44A1//MTMR9//SPHK2//ARSK//NEU1//ACBD3//HSD11B1//GPHN//ANP32B//EGLN3//VSIG4//DLL1//G3BP2//DGKE//ACBD5//RAB3GAP1//GJA1//MYD88//PTGFR//CCR1//CALM2//INHBB//BMP3//A2ML1//WFDC13//SPOCK1//NDST3//GLRX//IAH1//PRR16//INTS7//PIGS//GNPTG//AGTR2//WNT7A//RASD1//BTAF1//OTUD3//WDR20//NFATC2IP//VKORC1L1//STK33//OGN//EGLN1//ELOVL7//DCUN1D4//MSRB3//SULF1//DSEL//VWC2//CDC27//RAP2C//CCT6A//PHACTR2//AKAP6//IFNA1//PTPRG//PTP4A1//CDC14A//BNIP3//IL1RAP//RDH14//MYOG//ITM2A//TDRD5//ARL6IP1//UBXN2B//CD2AP//VPS37A//FZD6//YTHDF2//RAB12//CCNY//ADCYAP1//ARMCX3//DLG2//ENPP4//GRIK2//DGKH//TMEM86A//ULK2//EFR3A//SULT6B1//SAMSN1//SH3D19//ARRDC3//PMEPA1//LDLRAD4//ATG16L1//GPR183//NPNT//UBA5//SNAP25//TMED10// |
| GO:0007154 | cell communication | Biological process | 243 | 6744 | 574 | 17653 | 1.10814180936675 | 0.0217733082481698 | 0.297099644789858 | 1.66207557900811 | 0.423344947735192 | CSF2RA//SPRED1//EREG//RASGEF1A//IL2//IL5//MAP3K1//MET//MAP3K7CL//CCL2//BTC//TNF//LAMTOR3//RAPGEF2//GADD45A//CRKL//IGF1//NTF3//PIK3CB//PRKAA1//SOD1//C5AR1//DUSP22//BCL2A1//CBFB//AGTR2//SKP1//RPS6KA5//MYD88//REG3G//ADCYAP1//CCR1//EDN1//GJA1//GJB2//AR//PCDHB10//IL36G//CCL8//BMP3//AIMP1//SPHK2//APCDD1//CCNY//HBP1//LRP6//WNT7A//TNKS//PPP3CB//FZD3//FZD6//CDK6//CDS1//PTGES3//IQGAP2//ARAP2//CLCN6//DOK1//DPYSL2//ELK3//HTR3D//ESR1//EVI2A//CD2AP//APPL1//OSTF1//GLRB//CNOT7//LRP12//HIVEP2//NR3C2//NR4A2//HPCAL4//RASD1//KCNK10//PPARG//PSD//ARRDC3//MIER1//SMOC1//SMOC2//SPOCK1//ZNF217//LRP8//CALCB//RERGL//CAMLG//OR6A2//CBL//AKAP4//DLG5//PLAA//ARHGAP12//CD69//ULK2//PTPRG//YES1//CD8A//AKAP11//DGKH//DCX//ARHGEF4//TREM1//PRKAR2B//STK33//DGKE//AKAP6//BMPR2//SOCS5//CNOT6L//MDM4//CNOT6//SOX4//PPP1R15B//IFNG//EFNA5//NCK1//EPS15//TGFBR2//ACVR1//RAPGEF4//CHRM2//GPR183//FRZB//CCL28//PTGFR//SOS2//OR13A1//CALM2//FMR1//GRIK2//DLL1//SNIP1//MDFIC//NMI//NEUROD1//NOS1//ARHGAP1//RALGPS2//DIRAS3//USP28//G3BP2//PAK6//DLG2//SV2C//SLC1A1//SNAP25//NRXN1//TRPC3//RFX3//FLRT1//POLR2K//OTX2//UBE4B//BNIP3//CASP3//DSC2//INHBB//RRAGD//PLEKHF2//SAMSN1//SH3BGR//SH3BGRL//C1QTNF9//IFNA1//MACC1//OGN//VEGFB//PMEPA1//LDLRAD4//NYAP1//IL1RAP//PLP2//IL20RA//CNTNAP2//TNFAIP8L3//RFX4//DENND1B//PTPN22//SULF1//SHISA2//NPNT//STRAP//VWC2//MMRN2//FOSL1//MTM1//TSC1//AGFG1//RAB12//RAB30//RAB9B//RAB6B//RAB28//RAP2C//UBA5//ST18//ACSL1//IRF1//SLIT2//GSTA2//CA1//SYBU//BRWD1//RNF125//DCTPP1//ZFAND6//TRIM8//ZMYND11//PAQR5//ATR//PEX5L//NPTN//GATA2//YTHDF2//ARID5B//CPEB3//KCNJ10//RAB3GAP1//PTF1A//SYT6//PXK//GCSAML//FAM110C//OTUD3//IRF2//XAF1//CEBPB//FIBIN//POU4F1//ZMAT3//TBL1XR1//FASTK//SIVA1//KCNJ3//PATE1//DYNLL2//RAD9B//RBBP8//SERINC3//PHF14//MYB//ENPP5// |
| GO:1901214 | regulation of neuron death | Biological process | 16 | 281 | 574 | 17653 | 1.75113767406103 | 0.0218621829062662 | 0.297099644789858 | 1.66030647656678 | 0.0278745644599303 | EGLN3//CEBPB//EN1//GRIK2//NTF3//NR4A2//POU4F1//CCL2//SOD1//C5AR1//MYB//CASP3//CBL//NCOA7//TSC1//EGLN1// |
| GO:0006664 | glycolipid metabolic process | Biological process | 9 | 126 | 574 | 17653 | 2.19673967147835 | 0.0218870425902592 | 0.297099644789858 | 1.65981291698398 | 0.0156794425087108 | ST8SIA3//PIGP//PIGA//ARSK//NEU1//ST8SIA5//GLT6D1//PIGS//PRKAA1// |
| GO:0046887 | positive regulation of hormone secretion | Biological process | 9 | 126 | 574 | 17653 | 2.19673967147835 | 0.0218870425902592 | 0.297099644789858 | 1.65981291698398 | 0.0156794425087108 | INHBB//GJA1//SOX4//PPP3CB//SYBU//ADCYAP1//AIMP1//MYB//EDN1// |
| GO:1903509 | liposaccharide metabolic process | Biological process | 9 | 126 | 574 | 17653 | 2.19673967147835 | 0.0218870425902592 | 0.297099644789858 | 1.65981291698398 | 0.0156794425087108 | ST8SIA3//PIGP//PIGA//ARSK//NEU1//ST8SIA5//GLT6D1//PIGS//PRKAA1// |
| GO:0014070 | response to organic cyclic compound | Biological process | 42 | 937 | 574 | 17653 | 1.37853033813156 | 0.0220605403265979 | 0.297099644789858 | 1.65638385463445 | 0.0731707317073171 | SNIP1//ESR1//AR//PMEPA1//PPARG//PRKAA1//GJB2//PTGFR//RBBP8//WNT7A//CASP3//FOSL1//UBA5//CBFB//SRSF2//TRPC3//EDN1//CBL//IFNA1//NR3C2//NR4A2//PAQR5//TGFBR2//TNF//SLIT2//SOD1//GPD1//AKAP6//RAPGEF2//ADCYAP1//MYOG//POU4F1//LRP6//LRP8//CEBPB//CCL2//FIBIN//AGTR2//PRKAR2B//EFNA5//ACSL1//TET2// |
| GO:0043066 | negative regulation of apoptotic process | Biological process | 41 | 911 | 574 | 17653 | 1.38411478751764 | 0.0222000258004665 | 0.297099644789858 | 1.65364652081991 | 0.0714285714285714 | IL2//ADCYAP1//DLL1//CCL2//EDN1//IGF1//LRP6//ARL6IP1//CEBPB//EN1//GRIK2//NTF3//NR4A2//POU4F1//SOD1//C5AR1//TSC22D4//FZD3//CFDP1//GATA2//NEUROD1//ZMYND11//AR//ACVR1//TNF//BCL2A1//ZNF268//MDM4//MET//MYD88//ZFAND6//PRKAA1//TEX11//SPHK2//PTGFR//BNIP3//BTC//UNG//WNT7A//CASP3//CBL// |
| GO:0046486 | glycerolipid metabolic process | Biological process | 23 | 450 | 574 | 17653 | 1.57188927603562 | 0.0223270902733891 | 0.297099644789858 | 1.65116787166884 | 0.0400696864111498 | PIGP//PIGA//ACSL1//DDHD2//LCLAT1//GPD1//PLA2G12A//CDS1//ABHD3//SLC44A1//SERINC3//MTM1//PIK3CB//MTMR9//LDLR//PIGS//DGKH//EREG//ESR1//EFR3A//MET//BTC//TBL1XR1// |
| GO:0031570 | DNA integrity checkpoint | Biological process | 11 | 169 | 574 | 17653 | 2.00176277756015 | 0.0224554252673114 | 0.297099644789858 | 1.6486787157294 | 0.0191637630662021 | RAD9B//INTS7//ATR//USP28//GADD45A//CNOT6L//CNOT7//MDM4//CNOT6//SOX4//RBBP8// |
| GO:0048870 | cell motility | Biological process | 67 | 1615 | 574 | 17653 | 1.27587728287721 | 0.0224733198083163 | 0.297099644789858 | 1.64833276790413 | 0.116724738675958 | ACVR1//DCX//GATA2//GJA1//LRP12//NR4A2//SPOCK1//FZD3//RAPGEF2//SLIT2//CCR1//GPR183//CCL2//CCL8//CD2AP//NCK1//EDN1//BMPR2//WNT7A//STRAP//PTPRG//CLASP2//IFNG//TGFBR2//C5AR1//ARID5B//C5ORF30//IGF1//RASGEF1A//PIK3CB//PAK6//BTG1//YES1//POU4F1//POU3F2//ARX//EMX2//AKAP4//ZNF268//NTF3//FAM110C//PTP4A1//SULF1//RAP2C//LDLRAD4//DLG5//AGTR2//PPARG//APCDD1//PROCR//TREM1//CD84//AIMP1//TNF//RFX3//CCL28//VEGFB//SPRED1//MEOX2//MMRN2//CDK6//EREG//MEMO1//BTC//GCSAML//MET//PLAA// |
| GO:0051674 | localization of cell | Biological process | 67 | 1615 | 574 | 17653 | 1.27587728287721 | 0.0224733198083163 | 0.297099644789858 | 1.64833276790413 | 0.116724738675958 | ACVR1//DCX//GATA2//GJA1//LRP12//NR4A2//SPOCK1//FZD3//RAPGEF2//SLIT2//CCR1//GPR183//CCL2//CCL8//CD2AP//NCK1//EDN1//BMPR2//WNT7A//STRAP//PTPRG//CLASP2//IFNG//TGFBR2//C5AR1//ARID5B//C5ORF30//IGF1//RASGEF1A//PIK3CB//PAK6//BTG1//YES1//POU4F1//POU3F2//ARX//EMX2//AKAP4//ZNF268//NTF3//FAM110C//PTP4A1//SULF1//RAP2C//LDLRAD4//DLG5//AGTR2//PPARG//APCDD1//PROCR//TREM1//CD84//AIMP1//TNF//RFX3//CCL28//VEGFB//SPRED1//MEOX2//MMRN2//CDK6//EREG//MEMO1//BTC//GCSAML//MET//PLAA// |
| GO:0003323 | type B pancreatic cell development | Biological process | 3 | 19 | 574 | 17653 | 4.85595085274161 | 0.0224839336485946 | 0.297099644789858 | 1.64812770500567 | 0.00522648083623693 | RFX3//CDK6//DLL1// |
| GO:0021533 | cell differentiation in hindbrain | Biological process | 3 | 19 | 574 | 17653 | 4.85595085274161 | 0.0224839336485946 | 0.297099644789858 | 1.64812770500567 | 0.00522648083623693 | MTPN//WNT7A//GATA2// |
| GO:0051197 | positive regulation of coenzyme metabolic process | Biological process | 3 | 19 | 574 | 17653 | 4.85595085274161 | 0.0224839336485946 | 0.297099644789858 | 1.64812770500567 | 0.00522648083623693 | GPD1//IGF1//PRKAA1// |
| GO:0060004 | reflex | Biological process | 3 | 19 | 574 | 17653 | 4.85595085274161 | 0.0224839336485946 | 0.297099644789858 | 1.64812770500567 | 0.00522648083623693 | GLRB//GJA1//NPNT// |
| GO:0061050 | regulation of cell growth involved in cardiac muscle cell development | Biological process | 3 | 19 | 574 | 17653 | 4.85595085274161 | 0.0224839336485946 | 0.297099644789858 | 1.64812770500567 | 0.00522648083623693 | EDN1//IGF1//AKAP6// |
| GO:0071625 | vocalization behavior | Biological process | 3 | 19 | 574 | 17653 | 4.85595085274161 | 0.0224839336485946 | 0.297099644789858 | 1.64812770500567 | 0.00522648083623693 | NEUROG1//CNTNAP2//NRXN1// |
| GO:0089718 | amino acid import across plasma membrane | Biological process | 3 | 19 | 574 | 17653 | 4.85595085274161 | 0.0224839336485946 | 0.297099644789858 | 1.64812770500567 | 0.00522648083623693 | ARL6IP1//KCNJ10//SLC1A1// |
| GO:1905331 | negative regulation of morphogenesis of an epithelium | Biological process | 3 | 19 | 574 | 17653 | 4.85595085274161 | 0.0224839336485946 | 0.297099644789858 | 1.64812770500567 | 0.00522648083623693 | SULF1//TNF//CLASP2// |
| GO:0040013 | negative regulation of locomotion | Biological process | 19 | 353 | 574 | 17653 | 1.65533357680805 | 0.0225035170332678 | 0.297099644789858 | 1.64774960149863 | 0.0331010452961672 | SLIT2//STRAP//PTPRG//C5ORF30//SULF1//RAP2C//LDLRAD4//DLG5//AGTR2//PPARG//SPRED1//MEOX2//MMRN2//ARRDC3//CHMP3//TRIM8//CCL28//CLASP2//CCL2// |
| GO:0007626 | locomotory behavior | Biological process | 12 | 191 | 574 | 17653 | 1.93221081051499 | 0.022536170009936 | 0.297099644789858 | 1.64711988974883 | 0.0209059233449477 | GLRB//KCNJ10//EN1//NR4A2//TMOD1//TSC1//PPP3CB//ARRDC3//DDHD2//HPRT1//SNAP25//SOD1// |
| GO:0043113 | receptor clustering | Biological process | 5 | 50 | 574 | 17653 | 3.07543554006969 | 0.0227264753183677 | 0.29844495646237 | 1.64346791440615 | 0.00871080139372822 | GPHN//GLRB//NRXN1//DLG2//GRIK2// |
| GO:1990090 | cellular response to nerve growth factor stimulus | Biological process | 5 | 50 | 574 | 17653 | 3.07543554006969 | 0.0227264753183677 | 0.29844495646237 | 1.64346791440615 | 0.00871080139372822 | NTF3//RAPGEF2//STMN2//DYNC1LI2//CBL// |
| GO:0014743 | regulation of muscle hypertrophy | Biological process | 6 | 68 | 574 | 17653 | 2.71361959417914 | 0.0229995031153693 | 0.301445037924889 | 1.63828154644259 | 0.0104529616724739 | MTPN//EDN1//IGF1//JARID2//AKAP6//TRPC3// |
| GO:0010657 | muscle cell apoptotic process | Biological process | 7 | 87 | 574 | 17653 | 2.47448836557331 | 0.0231217763132768 | 0.30246145687948 | 1.63597880461912 | 0.0121951219512195 | ADCYAP1//BNIP3//EDN1//IGF1//LRP6//IFNG//PPARG// |
| GO:0050730 | regulation of peptidyl-tyrosine phosphorylation | Biological process | 14 | 237 | 574 | 17653 | 1.81671297725635 | 0.0233325467384081 | 0.303019276596449 | 1.63203785554514 | 0.024390243902439 | EREG//CBL//SOCS5//CNOT7//IFNG//IGF1//IL2//EFNA5//IL5//NTF3//YES1//LRP8//SAMSN1//RAP2C// |
| GO:0010976 | positive regulation of neuron projection development | Biological process | 15 | 260 | 574 | 17653 | 1.77428973465559 | 0.0233436408556484 | 0.303019276596449 | 1.63183140706517 | 0.0261324041811847 | EFNA5//BMPR2//SLIT2//CPEB3//FMR1//IL2//DLG5//LRP8//IL1RAP//PLAA//STMN2//ADCYAP1//NPTN//NCK1//RAPGEF2// |
| GO:0045927 | positive regulation of growth | Biological process | 15 | 260 | 574 | 17653 | 1.77428973465559 | 0.0233436408556484 | 0.303019276596449 | 1.63183140706517 | 0.0261324041811847 | MTPN//IL2//H3F3C//AKAP6//POU3F2//TGFBR2//ARX//DLL1//MTM1//EFNA5//BMPR2//ZFPM2//EDN1//IGF1//PLAA// |
| GO:0046651 | lymphocyte proliferation | Biological process | 15 | 260 | 574 | 17653 | 1.77428973465559 | 0.0233436408556484 | 0.303019276596449 | 1.63183140706517 | 0.0261324041811847 | CASP3//GPR183//IL2//IL5//PTPN22//PPP3CB//IFNA1//NCK1//IGF1//CEBPB//DLG5//TGFBR2//IRF1//HPRT1//IMPDH1// |
| GO:0032989 | cellular component morphogenesis | Biological process | 48 | 1101 | 574 | 17653 | 1.34078933627016 | 0.0234715484619159 | 0.304095943003711 | 1.62945825823769 | 0.0836236933797909 | AGFG1//MET//AR//FRMD6//OGN//POU4F1//WNT7A//SLIT2//ARX//DOK1//DPYSL2//EFNA5//OTX2//PIK3CB//ENAH//KLF7//RPS6KA5//NRXN1//CFDP1//BRWD1//CCL2//NR4A2//SH3D19//CASP3//TMOD1//FZD3//ULK2//PPP3CB//NYAP1//CNTNAP2//GJA1//NTF3//HPRT1//FMR1//HECW2//BMPR2//POU3F2//RAPGEF2//SOD1//EDN1//LRP8//MTM1//SNX2//YTHDF2//CRKL//PLAA//CLASP2//FLRT1// |
| GO:0071902 | positive regulation of protein serine/threonine kinase activity | Biological process | 19 | 355 | 574 | 17653 | 1.64600775384011 | 0.0236857372844166 | 0.305761176957062 | 1.62551309210258 | 0.0331010452961672 | GADD45A//TNF//CRKL//MAP3K1//MAP3K7CL//LAMTOR3//IGF1//NTF3//PIK3CB//PRKAA1//SOD1//C5AR1//MDFIC//EDN1//CCNY//RAPGEF2//ACSL1//IFNG//CALM2// |
| GO:0043243 | positive regulation of protein complex disassembly | Biological process | 4 | 34 | 574 | 17653 | 3.61815945890551 | 0.0237040634225046 | 0.305761176957062 | 1.62517719952974 | 0.00696864111498258 | STMN2//CALCOCO2//BNIP3//TNF// |
| GO:0007264 | small GTPase mediated signal transduction | Biological process | 28 | 578 | 574 | 17653 | 1.48983036543168 | 0.0237357116519973 | 0.305761176957062 | 1.62459774258888 | 0.0487804878048781 | CRKL//DOK1//IGF1//USP28//G3BP2//ARHGAP1//PAK6//ARAP2//APPL1//AGFG1//PSD//RAB12//RAB30//RAB9B//RAB6B//RAB28//RALGPS2//RAP2C//RAPGEF2//ARHGEF4//SOS2//MET//RASGEF1A//ARHGAP12//CD2AP//SLIT2//RAPGEF4//DIRAS3// |
| GO:0060429 | epithelium development | Biological process | 54 | 1264 | 574 | 17653 | 1.31387277819433 | 0.0238323506824711 | 0.306422410010555 | 1.6228331192953 | 0.0940766550522648 | EDN1//TGFBR2//ACVR1//NPNT//SLIT2//FZD3//DLL1//MEOX2//SOX4//LRP6//TSC1//FZD6//MET//APCDD1//GJA1//ARHGAP12//ESR1//RFX3//CDK6//NEUROD1//AR//FRMD6//GSTM3//WNT7A//EREG//CASP3//GSTA2//PPARG//IFNG//DLG5//EN1//KRT27//DSC2//KRTAP4-11//CEBPB//FOXF2//BTG1//CBFB//REG3G//TNF//FRZB//SOD1//SULF1//BTBD7//BMPR2//RAPGEF2//MAF//TMOD1//ARX//KLHL3//AGTR2//RAP2C//ARID4B//CLASP2// |
| GO:0055080 | cation homeostasis | Biological process | 33 | 707 | 574 | 17653 | 1.43549325066902 | 0.024140647952369 | 0.309797347441882 | 1.6172510773057 | 0.0574912891986063 | AGTR2//EDN1//CCR1//GRIK2//PIK3CB//STIM2//CCL8//CALCB//EGLN1//SKP1//SOD1//SLC30A5//ADCYAP1//ESR1//GATA2//GJA1//NPTN//IL2//LRP6//CCL28//PTGFR//C5AR1//NOS1//TRPC3//CALM2//AKAP6//SLC41A1//SLC30A7//SLC4A4//BNIP3//STEAP2//KCNJ10//FAM155A// |
| GO:0021954 | central nervous system neuron development | Biological process | 6 | 69 | 574 | 17653 | 2.67429177397364 | 0.0245230259525197 | 0.310864319022006 | 1.61042594225313 | 0.0104529616724739 | ARX//SLIT2//RAPGEF2//NR4A2//GATA2//HPRT1// |
| GO:0043242 | negative regulation of protein complex disassembly | Biological process | 6 | 69 | 574 | 17653 | 2.67429177397364 | 0.0245230259525197 | 0.310864319022006 | 1.61042594225313 | 0.0104529616724739 | STMN2//CLASP2//MID1IP1//TMOD1//MTPN//TNF// |
| GO:0060411 | cardiac septum morphogenesis | Biological process | 6 | 69 | 574 | 17653 | 2.67429177397364 | 0.0245230259525197 | 0.310864319022006 | 1.61042594225313 | 0.0104529616724739 | ZFPM2//BMPR2//TGFBR2//SOX4//ACVR1//EGLN1// |
| GO:1900006 | positive regulation of dendrite development | Biological process | 6 | 69 | 574 | 17653 | 2.67429177397364 | 0.0245230259525197 | 0.310864319022006 | 1.61042594225313 | 0.0104529616724739 | CPEB3//FMR1//IL2//DLG5//LRP8//IL1RAP// |
| GO:0002763 | positive regulation of myeloid leukocyte differentiation | Biological process | 5 | 51 | 574 | 17653 | 3.01513288242126 | 0.0245455487738801 | 0.310864319022006 | 1.61002725377378 | 0.00871080139372822 | IL5//CCR1//IFNG//POU4F1//TNF// |
| GO:0035176 | social behavior | Biological process | 5 | 51 | 574 | 17653 | 3.01513288242126 | 0.0245455487738801 | 0.310864319022006 | 1.61002725377378 | 0.00871080139372822 | EN1//CNTNAP2//PPP3CB//ATXN1//NRXN1// |
| GO:0051703 | intraspecies interaction between organisms | Biological process | 5 | 51 | 574 | 17653 | 3.01513288242126 | 0.0245455487738801 | 0.310864319022006 | 1.61002725377378 | 0.00871080139372822 | EN1//CNTNAP2//PPP3CB//ATXN1//NRXN1// |
| GO:0032943 | mononuclear cell proliferation | Biological process | 15 | 262 | 574 | 17653 | 1.76074553820784 | 0.0247858139832191 | 0.311808742362849 | 1.60579681399443 | 0.0261324041811847 | CASP3//GPR183//IL2//IL5//PTPN22//PPP3CB//IFNA1//NCK1//IGF1//CEBPB//DLG5//HPRT1//IMPDH1//TGFBR2//IRF1// |
| GO:0050769 | positive regulation of neurogenesis | Biological process | 22 | 430 | 574 | 17653 | 1.57347864840775 | 0.0248103167047669 | 0.311808742362849 | 1.60536769190454 | 0.0383275261324042 | FZD3//STMN2//ADCYAP1//NPTN//NCK1//RAPGEF2//GATA2//VWC2//NEUROD1//NEUROG1//EFNA5//PPARG//BMPR2//SLIT2//MYB//CPEB3//FMR1//IL2//DLG5//LRP8//IL1RAP//PLAA// |
| GO:0015849 | organic acid transport | Biological process | 17 | 309 | 574 | 17653 | 1.6919871903296 | 0.0248205678733739 | 0.311808742362849 | 1.60518828643715 | 0.029616724738676 | ARL6IP1//SLC6A14//SLC6A6//SLC1A1//MID1IP1//SLC17A5//SNAP25//SLC10A2//SLC35D1//SERINC3//PPARG//AGTR2//EDN1//ACSL1//PLA2G12A//KCNJ10//RAB3GAP1// |
| GO:0046942 | carboxylic acid transport | Biological process | 17 | 309 | 574 | 17653 | 1.6919871903296 | 0.0248205678733739 | 0.311808742362849 | 1.60518828643715 | 0.029616724738676 | ARL6IP1//SLC6A14//SLC6A6//SLC1A1//MID1IP1//SLC17A5//SNAP25//SLC10A2//SLC35D1//SERINC3//PPARG//AGTR2//EDN1//ACSL1//PLA2G12A//KCNJ10//RAB3GAP1// |
| GO:0001890 | placenta development | Biological process | 10 | 150 | 574 | 17653 | 2.05029036004646 | 0.0248506449997894 | 0.311808742362849 | 1.60466233464839 | 0.0174216027874564 | CEBPB//GATA2//BMPR2//GJA1//GJB2//ZNF568//FOSL1//EGLN1//PPARG//SOD1// |
| GO:0043523 | regulation of neuron apoptotic process | Biological process | 12 | 194 | 574 | 17653 | 1.90233126189878 | 0.0250700019673579 | 0.313978561676373 | 1.6008456319607 | 0.0209059233449477 | CEBPB//EN1//GRIK2//NTF3//NR4A2//POU4F1//CCL2//SOD1//C5AR1//MYB//CASP3//EGLN3// |
| GO:0009615 | response to virus | Biological process | 17 | 310 | 574 | 17653 | 1.68652916713499 | 0.0255024689167358 | 0.31700383309804 | 1.59341777309503 | 0.029616724738676 | SERINC3//RNF125//ZMYND11//CNOT7//IFNA1//IFNG//IRF1//IRF2//BNIP3//AIMP1//FMR1//CDK6//IFI44//CHRM2//CCL8//TNF//FOSL1// |
| GO:0061448 | connective tissue development | Biological process | 14 | 240 | 574 | 17653 | 1.79400406504065 | 0.0256367949428729 | 0.31700383309804 | 1.59113627032442 | 0.024390243902439 | WNT7A//RUNX3//SULF1//BMPR2//TGFBR2//MAF//EDN1//HOXA3//BMP3//LRP6//ARID5B//ARRDC3//TBL1XR1//FRZB// |
| GO:0043666 | regulation of phosphoprotein phosphatase activity | Biological process | 8 | 109 | 574 | 17653 | 2.25720039638142 | 0.0258040140182527 | 0.31700383309804 | 1.58831273084371 | 0.0139372822299652 | PHACTR2//AGTR2//CALM2//PPP1R15B//AKAP6//TNF//UBXN2B//TSC1// |
| GO:0003283 | atrial septum development | Biological process | 3 | 20 | 574 | 17653 | 4.61315331010453 | 0.0258271642816827 | 0.31700383309804 | 1.58792327498905 | 0.00522648083623693 | SOX4//ACVR1//BMPR2// |
| GO:0006491 | N-glycan processing | Biological process | 3 | 20 | 574 | 17653 | 4.61315331010453 | 0.0258271642816827 | 0.31700383309804 | 1.58792327498905 | 0.00522648083623693 | GNPTG//MAN1A1//ST8SIA3// |
| GO:0021697 | cerebellar cortex formation | Biological process | 3 | 20 | 574 | 17653 | 4.61315331010453 | 0.0258271642816827 | 0.31700383309804 | 1.58792327498905 | 0.00522648083623693 | DLL1//MTPN//WNT7A// |
| GO:0044346 | fibroblast apoptotic process | Biological process | 3 | 20 | 574 | 17653 | 4.61315331010453 | 0.0258271642816827 | 0.31700383309804 | 1.58792327498905 | 0.00522648083623693 | IER3IP1//CFDP1//BTG1// |
| GO:0048305 | immunoglobulin secretion | Biological process | 3 | 20 | 574 | 17653 | 4.61315331010453 | 0.0258271642816827 | 0.31700383309804 | 1.58792327498905 | 0.00522648083623693 | TNF//IL2//IL5// |
| GO:0060044 | negative regulation of cardiac muscle cell proliferation | Biological process | 3 | 20 | 574 | 17653 | 4.61315331010453 | 0.0258271642816827 | 0.31700383309804 | 1.58792327498905 | 0.00522648083623693 | GJA1//JARID2//TGFBR2// |
| GO:0071404 | cellular response to low-density lipoprotein particle stimulus | Biological process | 3 | 20 | 574 | 17653 | 4.61315331010453 | 0.0258271642816827 | 0.31700383309804 | 1.58792327498905 | 0.00522648083623693 | LDLR//PPARG//SOCS5// |
| GO:2000810 | regulation of bicellular tight junction assembly | Biological process | 3 | 20 | 574 | 17653 | 4.61315331010453 | 0.0258271642816827 | 0.31700383309804 | 1.58792327498905 | 0.00522648083623693 | TNF//GJA1//CBFB// |
| GO:0000291 | nuclear-transcribed mRNA catabolic process, exonucleolytic | Biological process | 4 | 35 | 574 | 17653 | 3.51478347436536 | 0.0260951419506539 | 0.318304634342675 | 1.58344033635525 | 0.00696864111498258 | DDX6//DCP2//CNOT7//CNOT6// |
| GO:0032456 | endocytic recycling | Biological process | 4 | 35 | 574 | 17653 | 3.51478347436536 | 0.0260951419506539 | 0.318304634342675 | 1.58344033635525 | 0.00696864111498258 | ARHGAP1//STX6//DENND1B//EPS15// |
| GO:0040036 | regulation of fibroblast growth factor receptor signaling pathway | Biological process | 4 | 35 | 574 | 17653 | 3.51478347436536 | 0.0260951419506539 | 0.318304634342675 | 1.58344033635525 | 0.00696864111498258 | SULF1//SHISA2//NPTN//OTX2// |
| GO:0050866 | negative regulation of cell activation | Biological process | 11 | 173 | 574 | 17653 | 1.95547924513101 | 0.0261214064853149 | 0.318304634342675 | 1.58300344254375 | 0.0191637630662021 | CASP3//CD84//CEBPB//DLG5//IRF1//SOCS5//IL2//PTPN22//DUSP22//SAMSN1//LDLR// |
| GO:0045840 | positive regulation of mitotic nuclear division | Biological process | 5 | 52 | 574 | 17653 | 2.95714955775931 | 0.0264556293335782 | 0.321219786684002 | 1.57748190289589 | 0.00871080139372822 | NEUROG1//EDN1//EREG//IGF1//BTC// |
| GO:0048641 | regulation of skeletal muscle tissue development | Biological process | 5 | 52 | 574 | 17653 | 2.95714955775931 | 0.0264556293335782 | 0.321219786684002 | 1.57748190289589 | 0.00871080139372822 | DLL1//MTM1//PRKAA1//MYOG//NLN// |
| GO:0065008 | regulation of biological quality | Biological process | 142 | 3770 | 574 | 17653 | 1.15838686124641 | 0.0265269186822794 | 0.321508156000458 | 1.57631319393028 | 0.247386759581882 | PTGES3//SNAP25//NOS1//AKAP6//ARRDC3//CASP3//SOD1//AGTR2//CALM2//ARL6IP1//SLC2A5//AR//EDN1//BMPR2//PRKAR2B//BTC//GJA1//PROCR//ENPP4//MMRN1//RCOR1//ZFPM2//GATA2//IFNA1//IRF1//IRF2//NFE2//DNAJC16//GLRX//NHLRC2//HSD11B1//CCR1//GRIK2//PIK3CB//STIM2//CCL8//CALCB//EGLN1//SKP1//SLC30A5//CLCN6//ADCYAP1//ESR1//NPTN//IL2//LRP6//CCL28//PTGFR//C5AR1//G3BP2//WNT7A//NRXN1//RAPGEF4//IFNG//NEUROD1//PPP3CB//RFX3//TNF//DLL1//PPARG//CFDP1//BRWD1//CCL2//MTPN//SLC1A1//TRPC3//BNIP3//SLC41A1//PLAA//INHBB//OTUD3//STX12//IGF1//MDM4//MTMR9//SOX4//TSC1//CCT6A//DGKH//FZD6//DGKE//STRAP//KCNK10//DPYSL2//SLIT2//NCK1//USP28//SLC30A7//TNKS//DCP2//ATR//HOXB6//IQGAP2//ARFIP1//SYBU//GPR183//HTR3D//GLRB//PXK//SLC44A1//RNF180//ADH4//RDH14//TRERF1//PRKAA1//LDLR//YES1//FMR1//YTHDF2//IL20RA//CDK6//PRR16//MTM1//KLHL3//CPEB3//RAPGEF2//KCNJ10//RAB3GAP1//ULK2//EFNA5//SYT6//SLC4A4//TMOD1//FLRT1//IL1RAP//DLG5//STEAP2//LRP8//CNOT6L//QKI//MET//AIMP1//TSC22D4//MYD88//DSC2//KCNJ3//FAM155A//GPHN//DTD1//DYNLL2//NEUROG1//MYB// |
| GO:0090288 | negative regulation of cellular response to growth factor stimulus | Biological process | 10 | 152 | 574 | 17653 | 2.023312855309 | 0.0269223862337344 | 0.325717527904733 | 1.56988644956637 | 0.0174216027874564 | STRAP//PMEPA1//TGFBR2//LDLRAD4//VWC2//MMRN2//SULF1//SHISA2//AGTR2//SLIT2// |
| GO:0030330 | DNA damage response, signal transduction by p53 class mediator | Biological process | 8 | 110 | 574 | 17653 | 2.23668039277795 | 0.0270683669334651 | 0.326898867091115 | 1.56753794493798 | 0.0139372822299652 | GADD45A//CNOT6L//CNOT7//MDM4//CNOT6//SOX4//SPRED1//ATR// |
| GO:0043069 | negative regulation of programmed cell death | Biological process | 41 | 924 | 574 | 17653 | 1.36464131106988 | 0.0271248497848128 | 0.326997075035096 | 1.56663265822388 | 0.0714285714285714 | IL2//ZNF268//ARL6IP1//IGF1//MDM4//MET//MYD88//ZFAND6//PRKAA1//TEX11//SPHK2//PTGFR//BCL2A1//BNIP3//BTC//UNG//WNT7A//CASP3//CBL//ADCYAP1//DLL1//CCL2//EDN1//LRP6//CEBPB//EN1//GRIK2//NTF3//NR4A2//POU4F1//SOD1//C5AR1//TSC22D4//FZD3//CFDP1//GATA2//NEUROD1//ZMYND11//AR//ACVR1//TNF// |
| GO:0051402 | neuron apoptotic process | Biological process | 13 | 219 | 574 | 17653 | 1.82560100552082 | 0.0272210283996021 | 0.327507231503464 | 1.56509547135033 | 0.0226480836236934 | EGLN3//CEBPB//EN1//GRIK2//NTF3//NR4A2//POU4F1//CCL2//SOD1//C5AR1//MYB//CASP3//BNIP3// |
| GO:0090257 | regulation of muscle system process | Biological process | 14 | 242 | 574 | 17653 | 1.77917758516428 | 0.0272640206027577 | 0.327507231503464 | 1.56441009872749 | 0.024390243902439 | CHRM2//EDN1//NPNT//MTPN//IGF1//JARID2//CALM2//MYOG//NOS1//SOD1//AKAP6//DSC2//NEUROG1//TRPC3// |
| GO:0045807 | positive regulation of endocytosis | Biological process | 9 | 131 | 574 | 17653 | 2.1128946458494 | 0.0273167972944809 | 0.32755939734499 | 1.56357022002962 | 0.0156794425087108 | FMR1//NTF3//CBL//GATA2//TNF//PPARG//CCL2//DLL1//LDLR// |
| GO:0023061 | signal release | Biological process | 22 | 435 | 574 | 17653 | 1.5553926869318 | 0.0277625170875659 | 0.330368769673528 | 1.55654116113925 | 0.0383275261324042 | AGTR2//SNAP25//WNT7A//NRXN1//RAPGEF4//IFNG//NEUROD1//PPP3CB//RFX3//TNF//INHBB//EDN1//ADCYAP1//GJA1//SOX4//SYBU//FMR1//SYT6//EFNA5//RAB3GAP1//AIMP1//MYB// |
| GO:0031571 | mitotic G1 DNA damage checkpoint | Biological process | 6 | 71 | 574 | 17653 | 2.5989596113265 | 0.0277689785400141 | 0.330368769673528 | 1.5564400951361 | 0.0104529616724739 | GADD45A//CNOT6L//CNOT7//MDM4//CNOT6//SOX4// |
| GO:0044819 | mitotic G1/S transition checkpoint | Biological process | 6 | 71 | 574 | 17653 | 2.5989596113265 | 0.0277689785400141 | 0.330368769673528 | 1.5564400951361 | 0.0104529616724739 | GADD45A//CNOT6L//CNOT7//MDM4//CNOT6//SOX4// |
| GO:0044248 | cellular catabolic process | Biological process | 89 | 2250 | 574 | 17653 | 1.21650561362756 | 0.0277812982144661 | 0.330368769673528 | 1.5562474636306 | 0.155052264808362 | UBXN2B//STX12//ATG16L1//ULK2//DCP2//CNOT6L//PAN3//CNOT7//CNOT6//BNIP3//SPO11//TET2//DCTPP1//CASP3//UBE4B//KLHL3//RNF125//USP28//UBE2G1//CBL//UBE2W//NOS1//GLUD2//NEU1//GGT6//RAB12//PTPN22//NRBF2//PGC//PIK3CB//DRAM1//ACBD5//RRAGD//MET//MTMR9//IFNG//PRKAA1//TRIM8//LDLR//VPS37A//DYNLL2//CHMP3//PLEKHF2//PLAA//EI24//TSC1//SNX5//LAMTOR3//DDHD2//GPD2//IDS//EGLN1//CDC27//SKP1//MTM1//RNF180//LONRF3//LONRF1//SOCS5//WDFY3//ACSL1//GSTM3//OGN//SLC44A1//RNF38//RNF145//ZNRF2//CD2AP//SPOPL//HECW2//TBL1XR1//MTRR//FMR1//YTHDF2//DDX6//UNG//MYOG//GPD1//IGF1//HPRT1//ENPP4//SH3D19//TNF//ABHD3//CPEB3//QKI//MYD88//CALCOCO2//RAB3GAP1// |
| GO:0002699 | positive regulation of immune effector process | Biological process | 11 | 175 | 574 | 17653 | 1.93313091090095 | 0.0281085472854548 | 0.330368769673528 | 1.55116159915292 | 0.0191637630662021 | PGC//TNF//IFNG//CD84//GATA2//MYB//SOCS5//UNG//RAET1E//IL2//IL5// |
| GO:0010633 | negative regulation of epithelial cell migration | Biological process | 8 | 111 | 574 | 17653 | 2.21653011896914 | 0.0283750818838431 | 0.330368769673528 | 1.54706287652482 | 0.0139372822299652 | SLIT2//AGTR2//PPARG//SPRED1//MEOX2//MMRN2//STRAP//PTPRG// |
| GO:0032675 | regulation of interleukin-6 production | Biological process | 8 | 111 | 574 | 17653 | 2.21653011896914 | 0.0283750818838431 | 0.330368769673528 | 1.54706287652482 | 0.0139372822299652 | TNF//SOCS5//ADCYAP1//MYD88//CEBPB//EREG//PTPN22//IL1RAP// |
| GO:0010467 | gene expression | Biological process | 209 | 5761 | 574 | 17653 | 1.11571954152849 | 0.0284212696332424 | 0.330368769673528 | 1.54635652519831 | 0.36411149825784 | NPAT//RRM2//TSHZ1//ZBTB18//CEBPB//ZNF268//TCERG1//STRAP//KLF12//ARX//EDN1//CC2D1B//EN1//ESR1//CPEB3//MYT1L//RCOR1//ZFPM2//GATA2//CNOT7//HOXC8//HSBP1//IFNG//IRF2//AR//JARID2//ZNF568//MAF//MDM4//MYB//NFIA//NR4A2//KLF3//POU4F1//PPARG//DUSP22//ZBTB4//RBBP8//TNF//ZNF217//LRP8//BHLHE41//TBL1XR1//ARID5B//BHLHE40//RUNX3//PHF14//DCP2//CNOT6L//PAN3//CNOT6//FMR1//MBNL1//SMU1//RBM25//SRSF2//POLR2K//RBM41//SNRPN//NOB1//SECISBP2L//ELK3//TFEC//FOXJ3//FOXF2//PTF1A//HIVEP2//MACC1//IRF1//MEOX2//MYBL1//NEUROD1//NHLH2//OTX2//POU3F2//RFX4//BMPR2//SOX4//BTF3//CARF//FOSL1//KLF7//CBFB//NMI//BAHD1//ARID4B//DIRAS3//ZMYND11//KLF17//NCOA7//ZNF782//ZNF800//ZFP30//KDM2A//EPC2//HBP1//ATAD2//MDFIC//HOXA3//HOXB6//HOXD1//ZNF680//NEUROG1//NFE2//COMMD2//VGLL1//BRWD1//PRKAA1//TRERF1//SLC2A4RG//ZNF630//ZBTB26//MIER1//RFX3//ATXN1//DMRTA1//SMARCD2//ZNF3//ZNF131//ZNF134//ZNF208//ZNF227//ZNF655//MED28//TSC22D4//PCGF5//LCOR//ST18//NRBF2//MLLT6//PAK6//BTG1//RHOXF2B//SNIP1//CBL//RPS6KA5//MED6//EMX2//BOLA3//NR3C2//RFX7//RHOXF2//BLZF1//ELAVL4//RTCA//SCAF8//CPEB1//QKI//AGFG1//CPEB2//MRPL35//METTL17//EIF3B//MTPN//DDX6//TSC1//KBTBD8//AIMP1//IMMP1L//CASP3//G3BP2//EREG//CDK6//IGF1//TGFBR2//RAB3GAP1//PTPN22//GJA1//LDLR//MYD88//PIK3CB//PTGFR//CCR1//PRR16//INTS7//AGTR2//IL5//LRP6//NOS1//PPP3CB//WNT7A//ACVR1//RASD1//BTAF1//C5AR1//BMP3//SULF1//VWC2//NCK1//MYOG//NABP1//EGLN1//FZD6//FASTK//YTHDF2//ADCYAP1//DLL1//IL2//MET//ARMCX3//TET2//YES1//NFATC2IP//TNKS//TRIM8//IL1RAP//EGLN3//PPP1R15B//NPNT// |
| GO:0016311 | dephosphorylation | Biological process | 23 | 461 | 574 | 17653 | 1.53438215665082 | 0.0284506636882508 | 0.330368769673528 | 1.54590759806388 | 0.0400696864111498 | PTPN22//MTM1//CPPED1//PPP3CB//DUSP22//TNF//NPNT//MTMR9//SPRED1//DLG2//PLEKHF2//PHACTR2//AGTR2//CALM2//PPP1R15B//AKAP6//PTPRG//PTP4A1//CDC14A//UBXN2B//TSC1//IFNG//NCK1// |
| GO:0006584 | catecholamine metabolic process | Biological process | 5 | 53 | 574 | 17653 | 2.90135428308461 | 0.0284579890347605 | 0.330368769673528 | 1.54579579230386 | 0.00871080139372822 | NR4A2//RNF180//AGTR2//HPRT1//MTPN// |
| GO:0009712 | catechol-containing compound metabolic process | Biological process | 5 | 53 | 574 | 17653 | 2.90135428308461 | 0.0284579890347605 | 0.330368769673528 | 1.54579579230386 | 0.00871080139372822 | MTPN//NR4A2//RNF180//AGTR2//HPRT1// |
| GO:0046637 | regulation of alpha-beta T cell differentiation | Biological process | 5 | 53 | 574 | 17653 | 2.90135428308461 | 0.0284579890347605 | 0.330368769673528 | 1.54579579230386 | 0.00871080139372822 | IFNG//MYB//SOCS5//TGFBR2//IL2// |
| GO:0071398 | cellular response to fatty acid | Biological process | 5 | 53 | 574 | 17653 | 2.90135428308461 | 0.0284579890347605 | 0.330368769673528 | 1.54579579230386 | 0.00871080139372822 | PPARG//PRKAA1//PTGFR//EDN1//LDLR// |
| GO:1901068 | guanosine-containing compound metabolic process | Biological process | 5 | 53 | 574 | 17653 | 2.90135428308461 | 0.0284579890347605 | 0.330368769673528 | 1.54579579230386 | 0.00871080139372822 | IMPDH1//HPRT1//GPHN//DLG2//AK4// |
| GO:1990089 | response to nerve growth factor | Biological process | 5 | 53 | 574 | 17653 | 2.90135428308461 | 0.0284579890347605 | 0.330368769673528 | 1.54579579230386 | 0.00871080139372822 | NTF3//RAPGEF2//STMN2//DYNC1LI2//CBL// |
| GO:0051250 | negative regulation of lymphocyte activation | Biological process | 9 | 132 | 574 | 17653 | 2.09688786822933 | 0.0285070986988085 | 0.330368769673528 | 1.54504698062862 | 0.0156794425087108 | CASP3//CEBPB//DLG5//IRF1//SOCS5//IL2//PTPN22//DUSP22//SAMSN1// |
| GO:0002755 | MyD88-dependent toll-like receptor signaling pathway | Biological process | 4 | 36 | 574 | 17653 | 3.41715060007743 | 0.0286257724425089 | 0.330368769673528 | 1.54324278542086 | 0.00696864111498258 | IRF1//REG3G//MAP3K1//MYD88// |
| GO:0003179 | heart valve morphogenesis | Biological process | 4 | 36 | 574 | 17653 | 3.41715060007743 | 0.0286257724425089 | 0.330368769673528 | 1.54324278542086 | 0.00696864111498258 | TGFBR2//ACVR1//BMPR2//SOX4// |
| GO:0006692 | prostanoid metabolic process | Biological process | 4 | 36 | 574 | 17653 | 3.41715060007743 | 0.0286257724425089 | 0.330368769673528 | 1.54324278542086 | 0.00696864111498258 | PTGES3//EDN1//MGST1//PLAA// |
| GO:0006693 | prostaglandin metabolic process | Biological process | 4 | 36 | 574 | 17653 | 3.41715060007743 | 0.0286257724425089 | 0.330368769673528 | 1.54324278542086 | 0.00696864111498258 | PTGES3//EDN1//MGST1//PLAA// |
| GO:0035418 | protein localization to synapse | Biological process | 4 | 36 | 574 | 17653 | 3.41715060007743 | 0.0286257724425089 | 0.330368769673528 | 1.54324278542086 | 0.00696864111498258 | NRXN1//SNAP25//WNT7A//MPP4// |
| GO:2000279 | negative regulation of DNA biosynthetic process | Biological process | 4 | 36 | 574 | 17653 | 3.41715060007743 | 0.0286257724425089 | 0.330368769673528 | 1.54324278542086 | 0.00696864111498258 | DCP2//PPARG//GJA1//BMPR2// |
| GO:0046632 | alpha-beta T cell differentiation | Biological process | 7 | 91 | 574 | 17653 | 2.36571964620745 | 0.0287148879469811 | 0.330832686857637 | 1.5418928741104 | 0.0121951219512195 | IFNG//IRF1//MYB//SOCS5//TGFBR2//GPR183//IL2// |
| GO:0045936 | negative regulation of phosphate metabolic process | Biological process | 29 | 614 | 574 | 17653 | 1.45256727462575 | 0.0289635290743098 | 0.331957372225219 | 1.53814852244307 | 0.0505226480836237 | SPRED1//DUSP22//IL2//LRP6//PPP1R15B//SLIT2//CBL//SOCS5//GADD45A//FLRT1//NCK1//DBNDD2//TNF//CALM2//DLG2//PLEKHF2//EGLN1//CDK6//PHACTR2//PTPN22//CASP3//MYOG//ZMYND11//NTF3//SAMSN1//STRAP//PMEPA1//LDLRAD4//PRKAR2B// |
| GO:0006643 | membrane lipid metabolic process | Biological process | 13 | 221 | 574 | 17653 | 1.80907972945276 | 0.0290187214435608 | 0.331957372225219 | 1.5373217263655 | 0.0226480836236934 | ST8SIA3//PIGP//PIGA//SERINC3//SPHK2//ARSK//NEU1//ST8SIA5//GLT6D1//PIGS//ELOVL7//PRKAA1//TNF// |
| GO:0046488 | phosphatidylinositol metabolic process | Biological process | 13 | 221 | 574 | 17653 | 1.80907972945276 | 0.0290187214435608 | 0.331957372225219 | 1.5373217263655 | 0.0226480836236934 | PIGP//PIGA//CDS1//MTM1//PIK3CB//MTMR9//PIGS//PLA2G12A//EREG//ESR1//EFR3A//MET//BTC// |
| GO:0051047 | positive regulation of secretion | Biological process | 20 | 388 | 574 | 17653 | 1.58527605158231 | 0.029277217185907 | 0.331957372225219 | 1.533470205559 | 0.0348432055749129 | AGTR2//EDN1//INHBB//GJA1//SOX4//PPP3CB//SYBU//GATA2//CLASP2//IGF1//TNF//IL2//IL5//ADCYAP1//RAB3GAP1//AIMP1//PTPN22//IFNG//IL1RAP//MYB// |
| GO:0033043 | regulation of organelle organization | Biological process | 53 | 1253 | 574 | 17653 | 1.30086259875254 | 0.0294141497437671 | 0.331957372225219 | 1.53144370097115 | 0.0923344947735192 | STMN2//CLASP2//MID1IP1//CDC27//CDC14A//CNOT6L//PAN3//CNOT6//BNIP3//CHMP3//HECW2//SLIT2//NCK1//JARID2//MET//CLIP1//EVI5//USP6NL//NEUROG1//ATAD2//MIER1//TNKS//DCP2//ATR//CCT6A//EFNA5//TSC1//FMR1//RPS6KA5//IQGAP2//ARFIP1//GATA2//NOS1//MTM1//MTBP//EDN1//EREG//IGF1//BTC//MYB//TMOD1//IL5//TNF//MIEF2//DDHD2//DYNLL2//CALCOCO2//RAB3GAP1//PRKAA1//SNIP1//CD2AP//NTF3//MTPN// |
| GO:0007289 | spermatid nucleus differentiation | Biological process | 3 | 21 | 574 | 17653 | 4.3934793429567 | 0.0294215738207254 | 0.331957372225219 | 1.53133409967867 | 0.00522648083623693 | SYCP1//SYCP3//AGFG1// |
| GO:0030813 | positive regulation of nucleotide catabolic process | Biological process | 3 | 21 | 574 | 17653 | 4.3934793429567 | 0.0294215738207254 | 0.331957372225219 | 1.53133409967867 | 0.00522648083623693 | GPD1//IGF1//PRKAA1// |
| GO:0036499 | PERK-mediated unfolded protein response | Biological process | 3 | 21 | 574 | 17653 | 4.3934793429567 | 0.0294215738207254 | 0.331957372225219 | 1.53133409967867 | 0.00522648083623693 | NCK1//PPP1R15B//CCL2// |
| GO:0043950 | positive regulation of cAMP-mediated signaling | Biological process | 3 | 21 | 574 | 17653 | 4.3934793429567 | 0.0294215738207254 | 0.331957372225219 | 1.53133409967867 | 0.00522648083623693 | NOS1//ADCYAP1//RAPGEF2// |
| GO:0046037 | GMP metabolic process | Biological process | 3 | 21 | 574 | 17653 | 4.3934793429567 | 0.0294215738207254 | 0.331957372225219 | 1.53133409967867 | 0.00522648083623693 | IMPDH1//HPRT1//DLG2// |
| GO:0050855 | regulation of B cell receptor signaling pathway | Biological process | 3 | 21 | 574 | 17653 | 4.3934793429567 | 0.0294215738207254 | 0.331957372225219 | 1.53133409967867 | 0.00522648083623693 | GCSAML//PTPN22//CBFB// |
| GO:1902932 | positive regulation of alcohol biosynthetic process | Biological process | 3 | 21 | 574 | 17653 | 4.3934793429567 | 0.0294215738207254 | 0.331957372225219 | 1.53133409967867 | 0.00522648083623693 | PRKAA1//IFNG//TNF// |
| GO:0044783 | G1 DNA damage checkpoint | Biological process | 6 | 72 | 574 | 17653 | 2.56286295005807 | 0.0294933630531149 | 0.331957372225219 | 1.53027570313029 | 0.0104529616724739 | GADD45A//CNOT6L//CNOT7//MDM4//CNOT6//SOX4// |
| GO:0010563 | negative regulation of phosphorus metabolic process | Biological process | 29 | 615 | 574 | 17653 | 1.45020537661823 | 0.0294996866342387 | 0.331957372225219 | 1.53018259736908 | 0.0505226480836237 | SPRED1//DUSP22//IL2//LRP6//PPP1R15B//SLIT2//CBL//SOCS5//GADD45A//FLRT1//NCK1//DBNDD2//TNF//CALM2//DLG2//PLEKHF2//EGLN1//CDK6//PHACTR2//PTPN22//CASP3//MYOG//ZMYND11//NTF3//SAMSN1//STRAP//PMEPA1//LDLRAD4//PRKAR2B// |
| GO:0051704 | multi-organism process | Biological process | 97 | 2484 | 574 | 17653 | 1.20095510220113 | 0.0296183945938336 | 0.332374439423402 | 1.52843848524542 | 0.168989547038328 | EREG//YTHDF2//AGFG1//BMPR2//POU4F1//PGC//REG3G//CEBPB//CDK6//IFI44//CHRM2//IFNG//CCL8//TNF//FOSL1//TEX11//SPO11//MYBL1//SYCP3//ACVR1//DDX6//RAI14//AR//ARID4B//SOD1//SYCP1//BTG1//SPATA9//ADAM18//TDRD5//STRBP//QKI//TRPC3//AKAP4//CRISP1//GLRB//ADCYAP1//SPHK2//TGFBR2//PTGFR//NHLH2//NFIA//CCL2//MYD88//C5AR1//CALCOCO2//RTN3//ZMYND11//RCOR1//FMR1//MDFIC//KLC1//BNIP3//UNG//CAMLG//SERINC3//NCK1//VPS37A//CHMP3//SIVA1//EPS15//LDLR//USP6NL//PTPN22//EDN1//GJA1//GJB2//MGST1//NOS1//CASP3//TRIM8//EN1//CNTNAP2//PPP3CB//ATXN1//NRXN1//MET//CBL//RNF125//ATG16L1//INHBB//CNOT7//HS3ST5//PAQR5//POLR2K//IFNA1//IRF1//IRF2//AIMP1//DMRTA1//IGF1//SYT6//ESR1//CDK19//PLAA//ULK2//EIF3B// |
| GO:0007265 | Ras protein signal transduction | Biological process | 23 | 463 | 574 | 17653 | 1.52775415597414 | 0.0296896417553605 | 0.332374439423402 | 1.52739504270738 | 0.0400696864111498 | ARHGAP1//PAK6//ARAP2//APPL1//AGFG1//PSD//RAB12//RAB30//RAB9B//RAB6B//RAB28//RALGPS2//RAP2C//RAPGEF2//ARHGEF4//SOS2//MET//RASGEF1A//IGF1//CRKL//DOK1//USP28//G3BP2// |
| GO:0042110 | T cell activation | Biological process | 23 | 463 | 574 | 17653 | 1.52775415597414 | 0.0296896417553605 | 0.332374439423402 | 1.52739504270738 | 0.0400696864111498 | IFNA1//PTPN22//IL2//PPP3CB//SOX4//YES1//IFNG//SOD1//NCK1//IGF1//CEBPB//DLG5//IRF1//TSC1//CD8A//CBFB//MYB//SOCS5//CASP3//DUSP22//CCL2//TGFBR2//GPR183// |
| GO:1902107 | positive regulation of leukocyte differentiation | Biological process | 9 | 133 | 574 | 17653 | 2.08112179403212 | 0.0297333337056274 | 0.332374439423402 | 1.52675639490569 | 0.0156794425087108 | IFNG//IL2//MYB//SOCS5//IL5//CCR1//POU4F1//TNF//TGFBR2// |
| GO:0022412 | cellular process involved in reproduction in multicellular organism | Biological process | 18 | 340 | 574 | 17653 | 1.62817175650748 | 0.0298149898415743 | 0.332737254618097 | 1.52556533428903 | 0.0313588850174216 | EREG//YTHDF2//AGFG1//TEX11//SPO11//MYBL1//SYCP3//ACVR1//TDRD5//STRBP//QKI//FOSL1//CRISP1//SYCP1//PAQR5//INHBB//IGF1//SLIT2// |
| GO:0033674 | positive regulation of kinase activity | Biological process | 28 | 590 | 574 | 17653 | 1.45952873088053 | 0.0298685190525589 | 0.332785493167143 | 1.524786310146 | 0.0487804878048781 | GADD45A//TNF//CRKL//MAP3K1//MAP3K7CL//LAMTOR3//IGF1//NTF3//PIK3CB//PRKAA1//SOD1//C5AR1//EREG//EFNA5//MDFIC//PAK6//TGFBR2//PRKAR2B//EDN1//TNFAIP8L3//CCNY//ADCYAP1//RAPGEF2//LRP8//ACSL1//IFNG//CALM2//MET// |
| GO:0016477 | cell migration | Biological process | 61 | 1474 | 574 | 17653 | 1.27273791006954 | 0.0300052515877507 | 0.333759073170984 | 1.52280272741434 | 0.106271777003484 | ACVR1//DCX//GATA2//GJA1//LRP12//NR4A2//SPOCK1//FZD3//RAPGEF2//SLIT2//CCR1//GPR183//CCL2//CCL8//CD2AP//NCK1//EDN1//BMPR2//WNT7A//STRAP//PTPRG//CLASP2//IFNG//TGFBR2//C5AR1//ARID5B//C5ORF30//IGF1//POU4F1//POU3F2//ARX//EMX2//ZNF268//NTF3//FAM110C//PTP4A1//SULF1//RAP2C//LDLRAD4//DLG5//AGTR2//PPARG//APCDD1//PROCR//PIK3CB//TREM1//YES1//CD84//AIMP1//TNF//CCL28//VEGFB//SPRED1//MEOX2//MMRN2//GCSAML//MET//PLAA//RASGEF1A//PAK6//BTG1// |
| GO:0035295 | tube development | Biological process | 46 | 1065 | 574 | 17653 | 1.32835713467799 | 0.0300951186106383 | 0.334209010121095 | 1.52150394069792 | 0.0801393728222996 | ELK3//EREG//HOXA3//MEOX2//CCL2//VEGFB//WNT7A//MMRN2//AIMP1//EDN1//TGFBR2//ACVR1//ZFPM2//QKI//NPNT//SLIT2//SOX4//LRP6//TSC1//FZD3//FZD6//GJA1//DLL1//SPRED1//SULF1//PPARG//HSD11B1//EN1//EGLN1//GATA2//BTG1//C5AR1//ADAM12//BMPR2//PHF14//FOXF2//TNF//MET//PIK3CB//DLG5//AR//ESR1//KLHL3//NFIA//AGTR2//RAPGEF2// |
| GO:0098771 | inorganic ion homeostasis | Biological process | 33 | 720 | 574 | 17653 | 1.40957462253194 | 0.0301995108091095 | 0.334765737132398 | 1.52000009196421 | 0.0574912891986063 | AGTR2//EDN1//CCR1//GRIK2//PIK3CB//STIM2//CCL8//CALCB//EGLN1//SKP1//SOD1//SLC30A5//ADCYAP1//ESR1//GATA2//GJA1//NPTN//IL2//LRP6//CCL28//PTGFR//C5AR1//NOS1//TRPC3//CALM2//AKAP6//SLC41A1//SLC30A7//SLC4A4//BNIP3//STEAP2//KCNJ10//FAM155A// |
| GO:2000379 | positive regulation of reactive oxygen species metabolic process | Biological process | 7 | 92 | 574 | 17653 | 2.34000530222694 | 0.0302442503900481 | 0.334765737132398 | 1.51935717510109 | 0.0121951219512195 | SOD1//AGTR2//EDN1//IFNG//TNF//GADD45A//TGFBR2// |
| GO:0051270 | regulation of cellular component movement | Biological process | 43 | 985 | 574 | 17653 | 1.34257592104565 | 0.0303513548525452 | 0.334958120906907 | 1.5178219177073 | 0.0749128919860627 | CCL8//SLIT2//EDN1//BMPR2//WNT7A//STRAP//PTPRG//CLASP2//IFNG//TGFBR2//C5AR1//C5ORF30//IGF1//NCK1//ZNF268//CCR1//NTF3//FAM110C//PTP4A1//ACVR1//SULF1//RAP2C//LDLRAD4//DLG5//AGTR2//PPARG//VEGFB//TNF//SPRED1//MEOX2//MMRN2//DSC2//CCL28//CDK6//EREG//MEMO1//BTC//GCSAML//GPR183//CCL2//MET//PLAA//RAPGEF2// |
| GO:0010632 | regulation of epithelial cell migration | Biological process | 15 | 269 | 574 | 17653 | 1.71492688107975 | 0.0303606872861059 | 0.334958120906907 | 1.51768840138048 | 0.0261324041811847 | EDN1//BMPR2//WNT7A//SLIT2//STRAP//PTPRG//CLASP2//IFNG//TGFBR2//AGTR2//PPARG//SPRED1//MEOX2//MMRN2//MET// |
| GO:0002294 | CD4-positive, alpha-beta T cell differentiation involved in immune response | Biological process | 5 | 54 | 574 | 17653 | 2.84762550006452 | 0.0305537867710247 | 0.336539511290619 | 1.51493495655584 | 0.00871080139372822 | IFNG//MYB//SOCS5//GPR183//IL2// |
| GO:0016197 | endosomal transport | Biological process | 12 | 200 | 574 | 17653 | 1.84526132404181 | 0.0307537490754357 | 0.338191227637677 | 1.51210193343244 | 0.0209059233449477 | STX6//DENND1B//EPS15//VPS37A//SNX2//HEATR5A//SNX5//RAB9B//RAB6B//EVI5//CHMP3//ARHGAP1// |
| GO:0030334 | regulation of cell migration | Biological process | 38 | 853 | 574 | 17653 | 1.37006507060549 | 0.0309150696439665 | 0.339202794148678 | 1.5098297708065 | 0.0662020905923345 | CCL8//SLIT2//EDN1//BMPR2//WNT7A//STRAP//PTPRG//CLASP2//IFNG//TGFBR2//C5AR1//C5ORF30//IGF1//ZNF268//CCR1//NTF3//FAM110C//PTP4A1//ACVR1//SULF1//RAP2C//LDLRAD4//DLG5//AGTR2//PPARG//VEGFB//TNF//SPRED1//MEOX2//MMRN2//CCL28//GCSAML//GPR183//CCL2//MET//PLAA//RAPGEF2//NCK1// |
| GO:0030030 | cell projection organization | Biological process | 61 | 1477 | 574 | 17653 | 1.27015279583108 | 0.0310803507074372 | 0.339202794148678 | 1.50751408930982 | 0.106271777003484 | CD2AP//NCK1//OGN//POU4F1//WNT7A//SLIT2//ARX//DOK1//DPYSL2//EFNA5//OTX2//PIK3CB//ENAH//KLF7//RPS6KA5//NRXN1//DYNLL2//RFX3//RFX4//GMNC//CDC14A//C5ORF30//SNAP25//STMN2//ADCYAP1//NPTN//RAPGEF2//PTPRG//SPOCK1//TSC1//FLRT1//NR4A2//ARHGEF4//UBE4B//CNTNAP2//LRP12//DBNDD2//PSD//FZD3//AKAP4//ULK2//PPP3CB//NYAP1//GJA1//NTF3//HPRT1//FMR1//HECW2//BMPR2//POU3F2//SOD1//FAM110C//CPEB3//IL2//DLG5//LRP8//SNX2//PRKAR2B//IL1RAP//PLAA//FAM92B// |
| GO:0035710 | CD4-positive, alpha-beta T cell activation | Biological process | 6 | 73 | 574 | 17653 | 2.52775523841344 | 0.0312865647268516 | 0.339202794148678 | 1.50464211988595 | 0.0104529616724739 | IFNG//MYB//SOCS5//GPR183//IL2//TGFBR2// |
| GO:0007259 | JAK-STAT cascade | Biological process | 11 | 178 | 574 | 17653 | 1.90055005285205 | 0.0312897309980034 | 0.339202794148678 | 1.50459817052542 | 0.0191637630662021 | IFNA1//IFNG//CNOT7//IGF1//IL2//FLRT1//NEUROD1//SOCS5//IL5//CCL2//NMI// |
| GO:0019068 | virion assembly | Biological process | 4 | 37 | 574 | 17653 | 3.32479517845371 | 0.0312971378898085 | 0.339202794148678 | 1.50449537668668 | 0.00696864111498258 | DDX6//VPS37A//CHMP3//USP6NL// |
| GO:0038179 | neurotrophin signaling pathway | Biological process | 4 | 37 | 574 | 17653 | 3.32479517845371 | 0.0312971378898085 | 0.339202794148678 | 1.50449537668668 | 0.00696864111498258 | NTF3//RAPGEF2//CASP3//AGTR2// |
| GO:0043370 | regulation of CD4-positive, alpha-beta T cell differentiation | Biological process | 4 | 37 | 574 | 17653 | 3.32479517845371 | 0.0312971378898085 | 0.339202794148678 | 1.50449537668668 | 0.00696864111498258 | IFNG//MYB//SOCS5//IL2// |
| GO:0055026 | negative regulation of cardiac muscle tissue development | Biological process | 4 | 37 | 574 | 17653 | 3.32479517845371 | 0.0312971378898085 | 0.339202794148678 | 1.50449537668668 | 0.00696864111498258 | GJA1//JARID2//TGFBR2//DLL1// |
| GO:0071542 | dopaminergic neuron differentiation | Biological process | 4 | 37 | 574 | 17653 | 3.32479517845371 | 0.0312971378898085 | 0.339202794148678 | 1.50449537668668 | 0.00696864111498258 | LRP6//NR4A2//EN1//OTX2// |
| GO:0050790 | regulation of catalytic activity | Biological process | 90 | 2292 | 574 | 17653 | 1.20763175657187 | 0.0316902667234711 | 0.342914038161336 | 1.49907410552273 | 0.156794425087108 | GADD45A//DIRAS3//TNF//CRKL//MAP3K1//MAP3K7CL//LAMTOR3//IGF1//NTF3//PIK3CB//PRKAA1//SOD1//C5AR1//SPRED1//DUSP22//EREG//CBL//SOCS5//FLRT1//LRP6//NCK1//DBNDD2//ANP32B//EGLN3//PPARG//CASP3//EFNA5//ADCYAP1//MDFIC//NPNT//ESR1//MTMR9//DLG2//PLEKHF2//A2ML1//WFDC13//SPOCK1//IQGAP2//CPEB2//TSC1//SLIT2//NOS1//CDK6//PAK6//TGFBR2//PLAA//PHACTR2//AGTR2//CALM2//PPP1R15B//AKAP6//PXK//DNAJB4//RAB3GAP1//ARHGAP1//SBF2//ARAP2//DENND1B//AGFG1//RP2//CCL2//CCL8//EVI5//ARHGAP12//RAPGEF2//USP6NL//MET//PRKAR2B//NR4A2//ARL6IP1//EDN1//PTPN22//NRBF2//TNFAIP8L3//UBXN2B//CCNY//EGLN1//MID1IP1//DCUN1D4//ARRDC3//PTGES3//TNKS//IFNG//RAP2C//LRP8//ACSL1//MTRR//DYNLL2//ST18//AR// |
| GO:0002761 | regulation of myeloid leukocyte differentiation | Biological process | 8 | 114 | 574 | 17653 | 2.15820037899627 | 0.0325552102644464 | 0.351149740061325 | 1.48737949532879 | 0.0139372822299652 | IL5//GATA2//CDK6//CEBPB//CCR1//IFNG//POU4F1//TNF// |
| GO:0046834 | lipid phosphorylation | Biological process | 8 | 114 | 574 | 17653 | 2.15820037899627 | 0.0325552102644464 | 0.351149740061325 | 1.48737949532879 | 0.0139372822299652 | EREG//ESR1//EFR3A//MET//PIK3CB//BTC//SPHK2//DGKE// |
| GO:0002287 | alpha-beta T cell activation involved in immune response | Biological process | 5 | 55 | 574 | 17653 | 2.79585049097244 | 0.0327440674849555 | 0.351504965715483 | 1.48486737328847 | 0.00871080139372822 | IFNG//MYB//SOCS5//GPR183//IL2// |
| GO:0002293 | alpha-beta T cell differentiation involved in immune response | Biological process | 5 | 55 | 574 | 17653 | 2.79585049097244 | 0.0327440674849555 | 0.351504965715483 | 1.48486737328847 | 0.00871080139372822 | IFNG//MYB//SOCS5//GPR183//IL2// |
| GO:0043388 | positive regulation of DNA binding | Biological process | 5 | 55 | 574 | 17653 | 2.79585049097244 | 0.0327440674849555 | 0.351504965715483 | 1.48486737328847 | 0.00871080139372822 | IFNG//IGF1//NEUROD1//POU4F1//PPARG// |
| GO:0090287 | regulation of cellular response to growth factor stimulus | Biological process | 15 | 272 | 574 | 17653 | 1.69601224636196 | 0.0330129202235596 | 0.351524751091288 | 1.48131605758777 | 0.0261324041811847 | NPNT//STRAP//PMEPA1//TGFBR2//LDLRAD4//SULF1//BMPR2//VWC2//MMRN2//OTX2//SHISA2//NPTN//AGTR2//SLIT2//DLL1// |
| GO:0046634 | regulation of alpha-beta T cell activation | Biological process | 6 | 74 | 574 | 17653 | 2.49359638384029 | 0.0331494220193743 | 0.351524751091288 | 1.47952403938544 | 0.0104529616724739 | IFNG//MYB//SOCS5//TGFBR2//IL2//IRF1// |
| GO:0030336 | negative regulation of cell migration | Biological process | 16 | 296 | 574 | 17653 | 1.66239758922686 | 0.0331683501875617 | 0.351524751091288 | 1.47927612999567 | 0.0278745644599303 | SLIT2//STRAP//PTPRG//C5ORF30//AGTR2//PPARG//SPRED1//MEOX2//MMRN2//CCL28//CLASP2//CCL2//SULF1//RAP2C//LDLRAD4//DLG5// |
| GO:0002335 | mature B cell differentiation | Biological process | 3 | 22 | 574 | 17653 | 4.19377573645866 | 0.0332656869286447 | 0.351524751091288 | 1.47800350404599 | 0.00522648083623693 | GPR183//DLL1//ITM2A// |
| GO:0043090 | amino acid import | Biological process | 3 | 22 | 574 | 17653 | 4.19377573645866 | 0.0332656869286447 | 0.351524751091288 | 1.47800350404599 | 0.00522648083623693 | ARL6IP1//KCNJ10//SLC1A1// |
| GO:0051194 | positive regulation of cofactor metabolic process | Biological process | 3 | 22 | 574 | 17653 | 4.19377573645866 | 0.0332656869286447 | 0.351524751091288 | 1.47800350404599 | 0.00522648083623693 | GPD1//IGF1//PRKAA1// |
| GO:0070723 | response to cholesterol | Biological process | 3 | 22 | 574 | 17653 | 4.19377573645866 | 0.0332656869286447 | 0.351524751091288 | 1.47800350404599 | 0.00522648083623693 | LRP6//LRP8//TGFBR2// |
| GO:0071379 | cellular response to prostaglandin stimulus | Biological process | 3 | 22 | 574 | 17653 | 4.19377573645866 | 0.0332656869286447 | 0.351524751091288 | 1.47800350404599 | 0.00522648083623693 | PPARG//PRKAA1//PTGFR// |
| GO:1901685 | glutathione derivative metabolic process | Biological process | 3 | 22 | 574 | 17653 | 4.19377573645866 | 0.0332656869286447 | 0.351524751091288 | 1.47800350404599 | 0.00522648083623693 | GSTA2//GSTM3//MGST1// |
| GO:1901687 | glutathione derivative biosynthetic process | Biological process | 3 | 22 | 574 | 17653 | 4.19377573645866 | 0.0332656869286447 | 0.351524751091288 | 1.47800350404599 | 0.00522648083623693 | GSTA2//GSTM3//MGST1// |
| GO:0021782 | glial cell development | Biological process | 7 | 94 | 574 | 17653 | 2.29021795537104 | 0.0334652676761296 | 0.352825281302141 | 1.47540569688785 | 0.0121951219512195 | CDK6//DLL1//POU3F2//KCNJ10//SOD1//LDLR//SOX4// |
| GO:0097696 | STAT cascade | Biological process | 11 | 180 | 574 | 17653 | 1.87943283004259 | 0.0335480281167142 | 0.352825281302141 | 1.47433300167077 | 0.0191637630662021 | CCL2//NMI//SOCS5//IFNA1//IFNG//CNOT7//IGF1//IL2//FLRT1//NEUROD1//IL5// |
| GO:0050801 | ion homeostasis | Biological process | 35 | 779 | 574 | 17653 | 1.38177463289395 | 0.0335844583200613 | 0.352825281302141 | 1.47386165199549 | 0.0609756097560976 | AGTR2//EDN1//CCR1//GRIK2//PIK3CB//STIM2//CCL8//CALCB//EGLN1//SKP1//SOD1//SLC30A5//ADCYAP1//ESR1//GATA2//GJA1//NPTN//IL2//LRP6//CCL28//PTGFR//C5AR1//NOS1//TRPC3//CALM2//AKAP6//SLC41A1//SLC30A7//SLC4A4//BNIP3//STEAP2//KCNJ10//PRKAA1//FAM155A//KLHL3// |
| GO:0034613 | cellular protein localization | Biological process | 71 | 1762 | 574 | 17653 | 1.23925041625964 | 0.0335974391776695 | 0.352825281302141 | 1.47369382360393 | 0.123693379790941 | IFNG//TNF//AGTR2//ZFAND6//AGFG1//SRSF2//TSC1//PAN3//AKAP6//ARL6IP1//MTX3//IMMP1L//STX6//TMED10//RAB12//STX12//SNX5//ARFIP1//RAB30//GRIK2//RAB9B//RAB6B//KIF13A//SNX2//EVI5//RAB28//USP6NL//GNPTG//PEX5L//IGF1//MZT1//ATG16L1//MTBP//SPO11//MDFIC//C16ORF70//BLZF1//DLG2//CLIP1//SOX4//ANP32B//SKP1//ATR//TNKS//ESCO2//ESR1//CNTNAP2//GPHN//CLASP2//EFR3A//LRP6//RAPGEF2//MIEF2//RAB3GAP1//GLRB//NRXN1//SNAP25//CD2AP//TRIM8//DYNLL2//APPL1//AR//ARL5A//NPTN//PRKAA1//SNIP1//CCT6A//FRMD6//ARMCX3//RRAGD//LAMTOR3// |
| GO:0043412 | macromolecule modification | Biological process | 162 | 4389 | 574 | 17653 | 1.13515734219934 | 0.0338107226378773 | 0.353814311283425 | 1.47094554694234 | 0.282229965156794 | GADD45A//DIRAS3//CSF2RA//SPRED1//EREG//RASGEF1A//IL2//IL5//MAP3K1//MET//MAP3K7CL//CCL2//BTC//TNF//LAMTOR3//RAPGEF2//CRKL//IGF1//NTF3//PIK3CB//PRKAA1//SOD1//C5AR1//DUSP22//UBE4B//RNF145//ZNRF2//RNF180//RNF125//UBE2W//HECW2//SKP1//THOP1//UBE2G1//LONRF3//CBFB//TNKS//CBL//UBE3B//LONRF1//LRP6//PPP1R15B//SLIT2//EFNA5//FMR1//NPTN//IFNG//SOCS5//RNF38//KLHL3//SPOPL//MDM4//DCAF8//TRIM8//KBTBD8//MTRR//YES1//CDK6//FASTK//PDIK1L//CDK19//PAN3//PPP3CB//CCL8//TGFBR2//RUNX3//ACVR1//RPS6KA5//FLRT1//NCK1//DBNDD2//PTPN22//MTM1//CPPED1//HS3ST5//BTG1//FUT9//FUT2//ST8SIA5//MAN1A1//ST8SIA3//CCDC126//TET2//VEGFB//PIGP//PIGA//MDFIC//CALM2//INHBB//BMP3//BMPR2//PIGS//EPC2//ARID4B//MIER1//TBL1XR1//ARID5B//ESR1//OTUD3//AR//USP28//WDR20//NFATC2IP//VKORC1L1//ATR//STK33//NOS1//EGLN3//GPHN//EGLN1//DCUN1D4//PAK6//JARID2//SOX4//RAP2C//PHACTR2//AGTR2//AKAP6//IFNA1//PRKAR2B//ESCO2//GATA2//PTPRG//PTP4A1//CDC14A//PCGF5//CNOT7//TDRD5//EDN1//UBXN2B//TSC1//ARSK//UNG//CASP3//CCNY//ADCYAP1//GRIK2//ZMYND11//WNT7A//ULK2//LRP8//SAMSN1//ARRDC3//MYB//STRAP//PMEPA1//LDLRAD4//ATG16L1//CCR1//GPR183//NPNT//TNFAIP8L3//KDM2A//RCOR1//CDC27//UBA5//ACSL1//GLRX//PLAA//NCOA7//NMI//RAB3GAP1// |
| GO:0050680 | negative regulation of epithelial cell proliferation | Biological process | 10 | 158 | 574 | 17653 | 1.94647818991752 | 0.0338735617529475 | 0.353814311283425 | 1.47013913555721 | 0.0174216027874564 | SULF1//GJA1//TNF//AIMP1//PPARG//CDK6//STRAP//EREG//AR//RUNX3// |
| GO:0050863 | regulation of T cell activation | Biological process | 17 | 321 | 574 | 17653 | 1.62873533274719 | 0.0339638230225825 | 0.353814311283425 | 1.46898343079074 | 0.029616724738676 | YES1//IFNG//SOD1//IL2//NCK1//IGF1//CEBPB//DLG5//CBFB//IRF1//MYB//SOCS5//CASP3//PTPN22//DUSP22//CCL2//TGFBR2// |
| GO:0000904 | cell morphogenesis involved in differentiation | Biological process | 32 | 701 | 574 | 17653 | 1.40390780716448 | 0.0340250819914786 | 0.353814311283425 | 1.46820081958815 | 0.0557491289198606 | MET//AR//FRMD6//OGN//POU4F1//WNT7A//SLIT2//ARX//DOK1//DPYSL2//EFNA5//OTX2//PIK3CB//ENAH//KLF7//RPS6KA5//NRXN1//NR4A2//CASP3//FZD3//ULK2//PPP3CB//HPRT1//FMR1//HECW2//BMPR2//POU3F2//RAPGEF2//SOD1//LRP8//YTHDF2//CRKL// |
| GO:0002820 | negative regulation of adaptive immune response | Biological process | 4 | 38 | 574 | 17653 | 3.23730056849441 | 0.0341101480048489 | 0.353814311283425 | 1.46711639617309 | 0.00696864111498258 | PPP3CB//DUSP22//IL2//SAMSN1// |
| GO:0035307 | positive regulation of protein dephosphorylation | Biological process | 4 | 38 | 574 | 17653 | 3.23730056849441 | 0.0341101480048489 | 0.353814311283425 | 1.46711639617309 | 0.00696864111498258 | AGTR2//CALM2//PPP1R15B//AKAP6// |
| GO:0040019 | positive regulation of embryonic development | Biological process | 4 | 38 | 574 | 17653 | 3.23730056849441 | 0.0341101480048489 | 0.353814311283425 | 1.46711639617309 | 0.00696864111498258 | AR//IGF1//CLASP2//OTX2// |
| GO:0071675 | regulation of mononuclear cell migration | Biological process | 4 | 38 | 574 | 17653 | 3.23730056849441 | 0.0341101480048489 | 0.353814311283425 | 1.46711639617309 | 0.00696864111498258 | C5AR1//SLIT2//TNF//CCR1// |
| GO:0007165 | signal transduction | Biological process | 223 | 6210 | 574 | 17653 | 1.10438345480763 | 0.0343988480681936 | 0.356075843029101 | 1.46345610062625 | 0.388501742160279 | CSF2RA//SPRED1//EREG//RASGEF1A//IL2//IL5//MAP3K1//MET//MAP3K7CL//CCL2//BTC//TNF//LAMTOR3//RAPGEF2//GADD45A//CRKL//IGF1//NTF3//PIK3CB//PRKAA1//SOD1//C5AR1//DUSP22//BCL2A1//CBFB//SKP1//RPS6KA5//MYD88//REG3G//SPHK2//APCDD1//CCNY//HBP1//LRP6//WNT7A//TNKS//PPP3CB//FZD3//FZD6//DOK1//PTPRG//YES1//CD8A//AKAP11//DGKH//DCX//AGTR2//ARHGEF4//TREM1//PRKAR2B//STK33//DGKE//DLG5//AKAP6//BMPR2//AKAP4//CBL//SOCS5//CNOT6L//CNOT7//MDM4//CNOT6//SOX4//PPP1R15B//CCR1//EDN1//IFNG//EFNA5//NCK1//EPS15//CAMLG//TGFBR2//ACVR1//RAPGEF4//CHRM2//ADCYAP1//GPR183//FRZB//RASD1//PPARG//CCL28//PTGFR//CCL8//SOS2//OR13A1//CALCB//CALM2//OR6A2//ESR1//FMR1//GRIK2//GLRB//DLL1//SNIP1//MDFIC//NMI//NEUROD1//NOS1//ARHGAP1//RALGPS2//DIRAS3//USP28//G3BP2//PAK6//CDS1//TRPC3//APPL1//FLRT1//POLR2K//OTX2//UBE4B//BNIP3//CASP3//SAMSN1//SH3BGR//SH3BGRL//C1QTNF9//IFNA1//MACC1//INHBB//OGN//IL36G//BMP3//VEGFB//AIMP1//PMEPA1//LDLRAD4//NYAP1//IL1RAP//PLP2//IL20RA//LRP8//TNFAIP8L3//RFX4//DENND1B//PTPN22//SULF1//SHISA2//NPNT//STRAP//VWC2//AR//NR4A2//MMRN2//MTM1//TSC1//RRAGD//ARAP2//AGFG1//PSD//RAB12//RAB30//RAB9B//RAB6B//RAB28//RAP2C//UBA5//ST18//ACSL1//IRF1//SLIT2//GSTA2//CA1//BRWD1//RNF125//ZFAND6//GJA1//MIER1//TRIM8//ZMYND11//NR3C2//PAQR5//ATR//PEX5L//NPTN//GATA2//YTHDF2//ARID5B//PTF1A//GCSAML//ARHGAP12//CD2AP//FAM110C//OTUD3//RAB3GAP1//IRF2//XAF1//CEBPB//IQGAP2//FIBIN//ARRDC3//POU4F1//ZMAT3//TBL1XR1//FASTK//SIVA1//PATE1//DYNLL2//RAD9B//RBBP8//SERINC3//NRXN1//PHF14//CDK6//PTGES3//CLCN6//DPYSL2//ELK3//HTR3D//EVI2A//OSTF1//LRP12//HIVEP2//HPCAL4//KCNK10//SMOC1//SMOC2//SPOCK1//ZNF217//RERGL//PLAA//CD69//ULK2// |
| GO:0071407 | cellular response to organic cyclic compound | Biological process | 27 | 572 | 574 | 17653 | 1.45169160108185 | 0.034433476466218 | 0.356075843029101 | 1.46301912792339 | 0.0470383275261324 | SNIP1//ESR1//AR//PMEPA1//UBA5//CBFB//NR3C2//NR4A2//PPARG//PAQR5//SOD1//GPD1//AKAP6//RAPGEF2//ADCYAP1//EDN1//MYOG//POU4F1//LRP6//LRP8//AGTR2//GJB2//CASP3//EFNA5//CEBPB//CCL2//TNF// |
| GO:0007423 | sensory organ development | Biological process | 25 | 521 | 574 | 17653 | 1.47573682344995 | 0.0345526886779659 | 0.356763104624555 | 1.4615181528463 | 0.0435540069686411 | SMOC1//GJA1//TGFBR2//PTF1A//LRIG1//NEUROD1//GATA2//NEUROG1//FZD3//FZD6//TSHZ1//EDN1//RAB3GAP1//GPD2//DLL1//FOXF2//LRP6//ANP32B//GJB2//MAF//SOD1//BMPR2//TMOD1//WNT7A//FRZB// |
| GO:0023052 | signaling | Biological process | 240 | 6724 | 574 | 17653 | 1.09771643310042 | 0.0346558566877313 | 0.357282863992571 | 1.46022336098784 | 0.418118466898955 | CSF2RA//SPRED1//EREG//RASGEF1A//IL2//IL5//MAP3K1//MET//MAP3K7CL//CCL2//BTC//TNF//LAMTOR3//RAPGEF2//GADD45A//CRKL//IGF1//NTF3//PIK3CB//PRKAA1//SOD1//C5AR1//DUSP22//BCL2A1//EPS15//CBFB//AGTR2//SKP1//RPS6KA5//MYD88//REG3G//ADCYAP1//CCR1//EDN1//GJA1//GJB2//AR//PCDHB10//IL36G//CCL8//BMP3//AIMP1//SPHK2//APCDD1//CCNY//HBP1//LRP6//WNT7A//TNKS//PPP3CB//FZD3//FZD6//CDK6//CDS1//PTGES3//IQGAP2//ARAP2//CLCN6//DOK1//DPYSL2//ELK3//HTR3D//ESR1//EVI2A//CD2AP//APPL1//OSTF1//GLRB//CNOT7//LRP12//HIVEP2//NR3C2//NR4A2//HPCAL4//RASD1//KCNK10//PPARG//PSD//ARRDC3//MIER1//SMOC1//SMOC2//SPOCK1//ZNF217//LRP8//CALCB//RERGL//CAMLG//OR6A2//CBL//AKAP4//DLG5//PLAA//ARHGAP12//CD69//ULK2//PTPRG//YES1//CD8A//AKAP11//DGKH//DCX//ARHGEF4//TREM1//PRKAR2B//STK33//DGKE//AKAP6//BMPR2//SOCS5//CNOT6L//MDM4//CNOT6//SOX4//PPP1R15B//IFNG//EFNA5//NCK1//TGFBR2//ACVR1//RAPGEF4//CHRM2//GPR183//FRZB//CCL28//PTGFR//SOS2//OR13A1//CALM2//FMR1//GRIK2//DLL1//SNIP1//MDFIC//NMI//NEUROD1//NOS1//ARHGAP1//RALGPS2//DIRAS3//USP28//G3BP2//PAK6//DLG2//SV2C//SLC1A1//SNAP25//NRXN1//TRPC3//RFX3//FLRT1//POLR2K//OTX2//UBE4B//BNIP3//CASP3//SAMSN1//SH3BGR//SH3BGRL//C1QTNF9//IFNA1//MACC1//INHBB//OGN//VEGFB//PMEPA1//LDLRAD4//NYAP1//IL1RAP//PLP2//IL20RA//CNTNAP2//TNFAIP8L3//RFX4//DENND1B//PTPN22//SULF1//SHISA2//NPNT//STRAP//VWC2//MMRN2//MTM1//TSC1//RRAGD//AGFG1//RAB12//RAB30//RAB9B//RAB6B//RAB28//RAP2C//UBA5//ST18//ACSL1//IRF1//SLIT2//GSTA2//CA1//SYBU//BRWD1//RNF125//ZFAND6//TRIM8//ZMYND11//PAQR5//ATR//PEX5L//NPTN//GATA2//YTHDF2//ARID5B//CPEB3//KCNJ10//RAB3GAP1//PTF1A//SYT6//PXK//GCSAML//FAM110C//OTUD3//IRF2//XAF1//CACNA2D3//CEBPB//FIBIN//POU4F1//ZMAT3//DSC2//KCNJ3//TBL1XR1//FASTK//SIVA1//PATE1//DYNLL2//RAD9B//RBBP8//SERINC3//PHF14//MYB// |
| GO:0033554 | cellular response to stress | Biological process | 78 | 1962 | 574 | 17653 | 1.2226502147066 | 0.0347951720729157 | 0.358094965744778 | 1.45848101144277 | 0.13588850174216 | INTS7//ATR//USP28//SPO11//SYCP1//RBBP8//NABP1//WRNIP1//RAD9B//GADD45A//EPC2//UBE2W//UNG//POLR2K//ESCO2//KDM2A//EGLN3//FMR1//ZBTB4//ZMAT3//CASP3//CBL//CNOT6L//CNOT7//MDM4//CNOT6//SOX4//PPP1R15B//CRKL//TNF//MDFIC//BCL2A1//DSC2//INHBB//RRAGD//BMPR2//PLEKHF2//SOD1//UBE4B//MAP3K1//PAK6//CPEB2//VKORC1L1//GJB2//NR4A2//CETN1//TFEC//CEBPB//NRBF2//UBA5//NCK1//CCL2//PRKAA1//DCTPP1//EDN1//PTPN22//SPRED1//GRIK2//ZMYND11//DUSP22//WNT7A//SKP1//EGLN1//MYB//BNIP3//HSBP1//GSTM3//MGST1//PPARG//CPEB1//SOCS5//PTGES3//HSPB8//MET//SERINC3//NCOA7//TSC1//CDK6// |
| GO:0001837 | epithelial to mesenchymal transition | Biological process | 9 | 137 | 574 | 17653 | 2.02035911391439 | 0.0350068205446534 | 0.358094965744778 | 1.45584733175471 | 0.0156794425087108 | CLASP2//TGFBR2//STRAP//LDLRAD4//LRP6//KBTBD8//ACVR1//FOXF2//DLG5// |
| GO:0006865 | amino acid transport | Biological process | 9 | 137 | 574 | 17653 | 2.02035911391439 | 0.0350068205446534 | 0.358094965744778 | 1.45584733175471 | 0.0156794425087108 | ARL6IP1//SLC6A14//SLC6A6//SLC1A1//SNAP25//SERINC3//KCNJ10//RAB3GAP1//SLC17A5// |
| GO:0008584 | male gonad development | Biological process | 9 | 137 | 574 | 17653 | 2.02035911391439 | 0.0350068205446534 | 0.358094965744778 | 1.45584733175471 | 0.0156794425087108 | AR//MGST1//ARID4B//ZFPM2//RNF38//ESR1//TEX11//ARID5B//CBL// |
| GO:1901185 | negative regulation of ERBB signaling pathway | Biological process | 5 | 56 | 574 | 17653 | 2.74592458934793 | 0.0350297616762115 | 0.358094965744778 | 1.45556281737995 | 0.00871080139372822 | CBL//SOCS5//EPS15//EREG//BTC// |
| GO:0021766 | hippocampus development | Biological process | 6 | 75 | 574 | 17653 | 2.46034843205575 | 0.0350827159775224 | 0.358094965744778 | 1.4549067924898 | 0.0104529616724739 | LRP8//CDK6//EMX2//NEUROD1//TSC1//CASP3// |
| GO:0022408 | negative regulation of cell-cell adhesion | Biological process | 10 | 159 | 574 | 17653 | 1.93423618872307 | 0.0351434760292856 | 0.358094965744778 | 1.45415528479641 | 0.0174216027874564 | CEBPB//DLG5//IRF1//SOCS5//CASP3//PTPN22//DUSP22//YTHDF2//CCL28//IL2// |
| GO:0043502 | regulation of muscle adaptation | Biological process | 7 | 95 | 574 | 17653 | 2.26611039794608 | 0.0351582222762875 | 0.358094965744778 | 1.45397309250376 | 0.0121951219512195 | MTPN//EDN1//IGF1//JARID2//MYOG//AKAP6//TRPC3// |
| GO:0050817 | coagulation | Biological process | 18 | 347 | 574 | 17653 | 1.59532679311972 | 0.0353738660169101 | 0.359749557702802 | 1.45131747346037 | 0.0313588850174216 | PROCR//ENPP4//MMRN1//RCOR1//ZFPM2//GATA2//IFNA1//IRF1//IRF2//NFE2//PRKAR2B//HS3ST5//DGKH//PIK3CB//TRPC3//FZD6//DGKE//EDN1// |
| GO:0008543 | fibroblast growth factor receptor signaling pathway | Biological process | 8 | 116 | 574 | 17653 | 2.12099002763427 | 0.0355640934043874 | 0.361141086627435 | 1.44898825780798 | 0.0139372822299652 | SULF1//OTX2//SHISA2//NPTN//SPRED1//FLRT1//POLR2K//CBL// |
| GO:0000902 | cell morphogenesis | Biological process | 43 | 997 | 574 | 17653 | 1.32641653182544 | 0.035928696294754 | 0.36332715534629 | 1.44455854127758 | 0.0749128919860627 | MET//AR//FRMD6//OGN//POU4F1//WNT7A//SLIT2//ARX//DOK1//DPYSL2//EFNA5//OTX2//PIK3CB//ENAH//KLF7//RPS6KA5//NRXN1//CFDP1//BRWD1//CCL2//NR4A2//SH3D19//CASP3//FZD3//ULK2//PPP3CB//NYAP1//CNTNAP2//GJA1//NTF3//HPRT1//FMR1//HECW2//BMPR2//POU3F2//RAPGEF2//SOD1//LRP8//SNX2//YTHDF2//CRKL//PLAA//FLRT1// |
| GO:0001558 | regulation of cell growth | Biological process | 20 | 397 | 574 | 17653 | 1.54933780356155 | 0.0359302518375878 | 0.36332715534629 | 1.44453973878501 | 0.0348432055749129 | MTPN//IL2//H3F3C//AKAP6//AGTR2//FRZB//GJA1//PPARG//BMPR2//BTG1//SLIT2//EI24//DPYSL2//ULK2//EFNA5//EDN1//IGF1//PLAA//SPOCK1//BLZF1// |
| GO:0016192 | vesicle-mediated transport | Biological process | 81 | 2050 | 574 | 17653 | 1.21517209144217 | 0.0359472652889544 | 0.36332715534629 | 1.44433414325306 | 0.141114982578397 | FMR1//NTF3//MMRN1//IGF1//NHLRC2//SOD1//VEGFB//PHACTR2//STX6//SYT6//STX12//SNAP25//CLASP2//CCR1//CCL8//DCTN6//TMED10//DYNLL2//DYNC1LI2//DDHD2//IER3IP1//KIF4B//KLC1//RAB6B//RP2//STEAP2//EPS15//DENND1B//DPYSL2//HEATR5A//SNX5//LRP12//LDLR//SNX2//LRP8//TGFBR2//GATA2//ARHGAP12//RAB12//MTM1//KIF13A//VPS37A//ARHGAP1//CHMP3//RAPGEF4//PPP3CB//EVI5//USP6NL//NCK1//PIK3CB//YES1//RAB9B//C16ORF70//BLZF1//CBL//CD84//IQGAP2//ENPP4//OSTF1//IMPDH1//MGST1//NEU1//CPPED1//KCMF1//RAP2C//SLC2A5//C5AR1//LAMTOR3//DLL1//CD2AP//TNF//PPARG//PRKAA1//LRP6//RAB3GAP1//IFNG//CCL2//RTN3//ARAP2//APPL1//AGFG1// |
| GO:0043408 | regulation of MAPK cascade | Biological process | 34 | 757 | 574 | 17653 | 1.38130526238269 | 0.0359942620260261 | 0.36332715534629 | 1.44376672615078 | 0.0592334494773519 | GADD45A//TNF//CRKL//MAP3K1//MAP3K7CL//LAMTOR3//IGF1//NTF3//PIK3CB//PRKAA1//SOD1//C5AR1//SPRED1//DUSP22//MDFIC//EDN1//AR//MET//PTPN22//GRIK2//ZMYND11//WNT7A//ADCYAP1//CCR1//GPR183//NPNT//NPTN//TNFAIP8L3//CCL2//CCL8//RAPGEF2//INHBB//PAK6//BMP3// |
| GO:0001649 | osteoblast differentiation | Biological process | 12 | 205 | 574 | 17653 | 1.80025495028469 | 0.0361549894250878 | 0.36404494007854 | 1.44183176115615 | 0.0209059233449477 | SMOC1//CDK6//TNF//CEBPB//NPNT//GJA1//IGF1//BMPR2//ACVR1//RDH14//BMP3//CBFB// |
| GO:0000165 | MAPK cascade | Biological process | 41 | 944 | 574 | 17653 | 1.3357294188862 | 0.0363340934226224 | 0.36404494007854 | 1.43968567148185 | 0.0714285714285714 | GADD45A//TNF//CRKL//MAP3K1//MAP3K7CL//LAMTOR3//IGF1//NTF3//PIK3CB//PRKAA1//SOD1//C5AR1//SPRED1//DUSP22//MDFIC//EDN1//INHBB//PAK6//BMP3//AR//MET//PTPN22//GRIK2//ZMYND11//WNT7A//SKP1//ADCYAP1//CCR1//GPR183//NPNT//NPTN//TNFAIP8L3//CCL2//CCL8//RAPGEF2//CSF2RA//EREG//RASGEF1A//IL2//IL5//BTC// |
| GO:0043524 | negative regulation of neuron apoptotic process | Biological process | 9 | 138 | 574 | 17653 | 2.00571883048023 | 0.0364195195331383 | 0.36404494007854 | 1.43866578796128 | 0.0156794425087108 | CEBPB//EN1//GRIK2//NTF3//NR4A2//POU4F1//CCL2//SOD1//C5AR1// |
| GO:0046546 | development of primary male sexual characteristics | Biological process | 9 | 138 | 574 | 17653 | 2.00571883048023 | 0.0364195195331383 | 0.36404494007854 | 1.43866578796128 | 0.0156794425087108 | RNF38//ESR1//TEX11//ARID5B//CBL//AR//MGST1//ARID4B//ZFPM2// |
| GO:0061351 | neural precursor cell proliferation | Biological process | 9 | 138 | 574 | 17653 | 2.00571883048023 | 0.0364195195331383 | 0.36404494007854 | 1.43866578796128 | 0.0156794425087108 | FZD3//C5AR1//ARX//EMX2//WNT7A//POU3F2//FZD6//ADCYAP1//GATA2// |
| GO:0007517 | muscle organ development | Biological process | 20 | 398 | 574 | 17653 | 1.54544499500989 | 0.0367333107984828 | 0.36404494007854 | 1.43493992663123 | 0.0348432055749129 | ZFPM2//UBE4B//POU4F1//ZBTB18//MEOX2//MYOG//MTPN//GJA1//DLL1//MTM1//NEUROG1//PRKAA1//ARID5B//EGLN1//JARID2//TGFBR2//EDN1//IGF1//AKAP6//NLN// |
| GO:0002286 | T cell activation involved in immune response | Biological process | 7 | 96 | 574 | 17653 | 2.24250508130081 | 0.0369069524579084 | 0.36404494007854 | 1.4328918146081 | 0.0121951219512195 | IFNG//TSC1//MYB//SOCS5//GPR183//IL2//IFNA1// |
| GO:0033146 | regulation of intracellular estrogen receptor signaling pathway | Biological process | 4 | 39 | 574 | 17653 | 3.15429286160993 | 0.0370654454893189 | 0.36404494007854 | 1.43103077572866 | 0.00696864111498258 | AR//ESR1//UBA5//CBFB// |
| GO:0043029 | T cell homeostasis | Biological process | 4 | 39 | 574 | 17653 | 3.15429286160993 | 0.0370654454893189 | 0.36404494007854 | 1.43103077572866 | 0.00696864111498258 | IL2//TSC22D4//PPP3CB//CASP3// |
| GO:0046638 | positive regulation of alpha-beta T cell differentiation | Biological process | 4 | 39 | 574 | 17653 | 3.15429286160993 | 0.0370654454893189 | 0.36404494007854 | 1.43103077572866 | 0.00696864111498258 | IFNG//MYB//SOCS5//TGFBR2// |
| GO:0048016 | inositol phosphate-mediated signaling | Biological process | 4 | 39 | 574 | 17653 | 3.15429286160993 | 0.0370654454893189 | 0.36404494007854 | 1.43103077572866 | 0.00696864111498258 | PPP3CB//IGF1//AKAP6//EDN1// |
| GO:0061028 | establishment of endothelial barrier | Biological process | 4 | 39 | 574 | 17653 | 3.15429286160993 | 0.0370654454893189 | 0.36404494007854 | 1.43103077572866 | 0.00696864111498258 | WNT7A//RAP2C//RAPGEF2//TNF// |
| GO:0031016 | pancreas development | Biological process | 6 | 76 | 574 | 17653 | 2.42797542637081 | 0.0370871694072215 | 0.36404494007854 | 1.43077631193252 | 0.0104529616724739 | RFX3//CDK6//DLL1//NEUROD1//PTF1A//SOX4// |
| GO:1901654 | response to ketone | Biological process | 11 | 183 | 574 | 17653 | 1.84862245577959 | 0.0371480427960316 | 0.36404494007854 | 1.4300640627991 | 0.0191637630662021 | GJB2//FOSL1//EDN1//CBL//SLIT2//PPARG//PRKAA1//AR//FIBIN//AGTR2//EFNA5// |
| GO:0070727 | cellular macromolecule localization | Biological process | 71 | 1772 | 574 | 17653 | 1.23225690375253 | 0.037310695617823 | 0.36404494007854 | 1.4281666539298 | 0.123693379790941 | IFNG//TNF//AGTR2//ZFAND6//AGFG1//SRSF2//TSC1//PAN3//AKAP6//ARL6IP1//MTX3//IMMP1L//STX6//TMED10//RAB12//STX12//SNX5//ARFIP1//RAB30//GRIK2//RAB9B//RAB6B//KIF13A//SNX2//EVI5//RAB28//USP6NL//GNPTG//PEX5L//IGF1//MZT1//ATG16L1//MTBP//SPO11//FRMD6//ARMCX3//RRAGD//LAMTOR3//MDFIC//C16ORF70//BLZF1//DLG2//CLIP1//SOX4//ANP32B//SKP1//ATR//TNKS//ESCO2//ESR1//CNTNAP2//GPHN//CLASP2//EFR3A//LRP6//RAPGEF2//MIEF2//RAB3GAP1//GLRB//NRXN1//SNAP25//CD2AP//TRIM8//DYNLL2//APPL1//AR//ARL5A//NPTN//PRKAA1//SNIP1//CCT6A// |
| GO:0001516 | prostaglandin biosynthetic process | Biological process | 3 | 23 | 574 | 17653 | 4.01143766096046 | 0.0373572657717739 | 0.36404494007854 | 1.4276249178725 | 0.00522648083623693 | PTGES3//EDN1//MGST1// |
| GO:0007026 | negative regulation of microtubule depolymerization | Biological process | 3 | 23 | 574 | 17653 | 4.01143766096046 | 0.0373572657717739 | 0.36404494007854 | 1.4276249178725 | 0.00522648083623693 | STMN2//CLASP2//MID1IP1// |
| GO:0007141 | male meiosis I | Biological process | 3 | 23 | 574 | 17653 | 4.01143766096046 | 0.0373572657717739 | 0.36404494007854 | 1.4276249178725 | 0.00522648083623693 | SPO11//MYBL1//SYCP3// |
| GO:0015813 | L-glutamate transmembrane transport | Biological process | 3 | 23 | 574 | 17653 | 4.01143766096046 | 0.0373572657717739 | 0.36404494007854 | 1.4276249178725 | 0.00522648083623693 | ARL6IP1//KCNJ10//SLC1A1// |
| GO:0032660 | regulation of interleukin-17 production | Biological process | 3 | 23 | 574 | 17653 | 4.01143766096046 | 0.0373572657717739 | 0.36404494007854 | 1.4276249178725 | 0.00522648083623693 | IFNG//IL2//MYD88// |
| GO:0045940 | positive regulation of steroid metabolic process | Biological process | 3 | 23 | 574 | 17653 | 4.01143766096046 | 0.0373572657717739 | 0.36404494007854 | 1.4276249178725 | 0.00522648083623693 | PRKAA1//IFNG//TNF// |
| GO:0046457 | prostanoid biosynthetic process | Biological process | 3 | 23 | 574 | 17653 | 4.01143766096046 | 0.0373572657717739 | 0.36404494007854 | 1.4276249178725 | 0.00522648083623693 | PTGES3//EDN1//MGST1// |
| GO:0051043 | regulation of membrane protein ectodomain proteolysis | Biological process | 3 | 23 | 574 | 17653 | 4.01143766096046 | 0.0373572657717739 | 0.36404494007854 | 1.4276249178725 | 0.00522648083623693 | SH3D19//IFNG//TNF// |
| GO:0090140 | regulation of mitochondrial fission | Biological process | 3 | 23 | 574 | 17653 | 4.01143766096046 | 0.0373572657717739 | 0.36404494007854 | 1.4276249178725 | 0.00522648083623693 | MIEF2//DDHD2//BNIP3// |
| GO:0000380 | alternative mRNA splicing, via spliceosome | Biological process | 5 | 57 | 574 | 17653 | 2.69775047374534 | 0.0374116852105636 | 0.364050686444664 | 1.42699272855227 | 0.00871080139372822 | FMR1//MBNL1//SMU1//RBM25//SRSF2// |
| GO:0051480 | regulation of cytosolic calcium ion concentration | Biological process | 17 | 325 | 574 | 17653 | 1.60868935942107 | 0.0375022198135805 | 0.36440734568857 | 1.42594302495878 | 0.029616724738676 | ADCYAP1//CCR1//EDN1//ESR1//GATA2//GJA1//NPTN//IL2//LRP6//CCL28//PTGFR//C5AR1//NOS1//TRPC3//CALM2//AKAP6//FAM155A// |
| GO:0007088 | regulation of mitotic nuclear division | Biological process | 10 | 161 | 574 | 17653 | 1.91020840998117 | 0.0377820385116028 | 0.366277890068319 | 1.42271461363344 | 0.0174216027874564 | CDC27//CDC14A//HECW2//NEUROG1//MTBP//EDN1//EREG//IGF1//BTC//TNKS// |
| GO:0009410 | response to xenobiotic stimulus | Biological process | 15 | 277 | 574 | 17653 | 1.66539830689694 | 0.0378030411455991 | 0.366277890068319 | 1.42247326101924 | 0.0261324041811847 | HPRT1//NR4A2//SOD1//PTGES3//MGST1//NAT1//PPARG//PRKAA1//ACSL1//GSTM3//EDN1//FIBIN//AGTR2//GJB2//PRKAR2B// |
| GO:0051050 | positive regulation of transport | Biological process | 41 | 947 | 574 | 17653 | 1.33149796349374 | 0.0379012963449825 | 0.366704531017334 | 1.42134593552416 | 0.0714285714285714 | ARL6IP1//FMR1//NTF3//AGTR2//EDN1//TRPC3//IGF1//INHBB//GJA1//SOX4//STIM2//IFNG//PPP3CB//SYBU//GATA2//DLL1//LDLR//CLASP2//ANP32B//CBL//TNF//IL2//IL5//AKAP6//CCR1//PPARG//ADCYAP1//CALM2//RAB3GAP1//AIMP1//CCL2//MIEF2//DYNLL2//NOS1//PTPN22//PLAA//PRKAA1//SNIP1//GLRX//IL1RAP//MYB// |
| GO:0045596 | negative regulation of cell differentiation | Biological process | 31 | 682 | 574 | 17653 | 1.39792524548622 | 0.0383284309682673 | 0.369803502055966 | 1.4164789588769 | 0.054006968641115 | STRAP//LDLRAD4//FRZB//PPARG//BHLHE41//STMN2//PTPRG//SPOCK1//TSC1//DLL1//IFNG//IRF1//ZFPM2//GATA2//TNF//REG3G//SOCS5//CDK6//DDX6//ULK2//WNT7A//RAPGEF2//EREG//MED28//ADCYAP1//IGF1//LDLR//SLIT2//EFNA5//IL2//ANP32B// |
| GO:0001944 | vasculature development | Biological process | 34 | 761 | 574 | 17653 | 1.37404478794178 | 0.0383309559280249 | 0.369803502055966 | 1.41645034982758 | 0.0592334494773519 | ELK3//EREG//HOXA3//MEOX2//CCL2//VEGFB//WNT7A//MMRN2//AIMP1//CRKL//SPHK2//TGFBR2//RAPGEF2//EDN1//ACVR1//ZFPM2//QKI//PPP3CB//BMPR2//SLIT2//SPRED1//SULF1//PPARG//SOX4//EGLN1//GATA2//BTG1//C5AR1//ADAM12//GJA1//PIK3CB//FOSL1//DLL1//ANP32B// |
| GO:0051216 | cartilage development | Biological process | 11 | 184 | 574 | 17653 | 1.83857559460688 | 0.0384059338853103 | 0.369999046818167 | 1.41560167004515 | 0.0191637630662021 | WNT7A//RUNX3//SULF1//BMPR2//TGFBR2//MAF//LRP6//FRZB//EDN1//HOXA3//BMP3// |
| GO:0051668 | localization within membrane | Biological process | 8 | 118 | 574 | 17653 | 2.08504104411504 | 0.0387554192855486 | 0.371131695950732 | 1.41166756022222 | 0.0139372822299652 | DLG2//GRIK2//CLIP1//GPHN//GLRB//NRXN1//SNAP25//NPTN// |
| GO:0009056 | catabolic process | Biological process | 98 | 2543 | 574 | 17653 | 1.18518554041223 | 0.0389406557840338 | 0.371131695950732 | 1.40959673897446 | 0.170731707317073 | UBXN2B//STX12//ATG16L1//ULK2//DCP2//CNOT6L//PAN3//CNOT7//CNOT6//BNIP3//SPO11//IDS//TET2//DCTPP1//CASP3//UBE4B//KLHL3//RNF125//USP28//UBE2G1//CBL//UBE2W//NOS1//GLUD2//NEU1//HSD11B1//TRERF1//GGT6//RAB12//PTPN22//NRBF2//PGC//PIK3CB//DRAM1//ACBD5//RRAGD//MET//MTMR9//IFNG//PRKAA1//TRIM8//LDLR//IAH1//TBL1XR1//PLA2G12A//VPS37A//DYNLL2//CHMP3//PLEKHF2//PLAA//EI24//TSC1//SNX5//LAMTOR3//DDHD2//GPD2//EGLN1//CDC27//SKP1//MTM1//RNF180//LONRF3//LONRF1//SOCS5//WDFY3//MDM4//ACSL1//GSTM3//OGN//FUT9//FUT2//SLC44A1//RNF38//RNF145//ZNRF2//CD2AP//SPOPL//HECW2//MTRR//FMR1//YTHDF2//DDX6//UNG//GJA1//TNF//MYOG//GPD1//IGF1//HPRT1//ENPP4//ADH4//SH3D19//ABHD3//CPEB3//QKI//MYD88//CALCOCO2//RAB3GAP1// |
| GO:0002285 | lymphocyte activation involved in immune response | Biological process | 10 | 162 | 574 | 17653 | 1.89841700004302 | 0.0391513637748603 | 0.371131695950732 | 1.40725310539197 | 0.0174216027874564 | IFNA1//GPR183//DLL1//ITM2A//IFNG//TSC1//MYB//SOCS5//UNG//IL2// |
| GO:0006446 | regulation of translational initiation | Biological process | 6 | 77 | 574 | 17653 | 2.39644327797638 | 0.039163446165595 | 0.371131695950732 | 1.40711909968679 | 0.0104529616724739 | FMR1//TNF//NCK1//YTHDF2//PPP1R15B//EIF3B// |
| GO:0007422 | peripheral nervous system development | Biological process | 6 | 77 | 574 | 17653 | 2.39644327797638 | 0.039163446165595 | 0.371131695950732 | 1.40711909968679 | 0.0104529616724739 | GSTM3//POU3F2//SOD1//POU4F1//RUNX3//NTF3// |
| GO:0021675 | nerve development | Biological process | 6 | 77 | 574 | 17653 | 2.39644327797638 | 0.039163446165595 | 0.371131695950732 | 1.40711909968679 | 0.0104529616724739 | NEUROG1//POU4F1//HOXA3//SULF1//LRIG1//NTF3// |
| GO:0034250 | positive regulation of cellular amide metabolic process | Biological process | 9 | 140 | 574 | 17653 | 1.97706570433051 | 0.039360987496084 | 0.371131695950732 | 1.40493401443987 | 0.0156794425087108 | CPEB3//FMR1//PRR16//SOX4//NCK1//TNF//PPARG//YTHDF2//ESR1// |
| GO:0030278 | regulation of ossification | Biological process | 11 | 185 | 574 | 17653 | 1.82863734814954 | 0.0396932477157816 | 0.371131695950732 | 1.40128336550859 | 0.0191637630662021 | GJA1//BMPR2//ACVR1//CCR1//SMOC1//CDK6//TNF//CEBPB//NPNT//IGF1//LRP6// |
| GO:0006749 | glutathione metabolic process | Biological process | 5 | 58 | 574 | 17653 | 2.65123753454283 | 0.0398905394153212 | 0.371131695950732 | 1.39913009095229 | 0.00871080139372822 | GGT6//GSTA2//GSTM3//MGST1//SOD1// |
| GO:0007028 | cytoplasm organization | Biological process | 2 | 10 | 574 | 17653 | 6.15087108013937 | 0.039950299371896 | 0.371131695950732 | 1.39847996190505 | 0.00348432055749129 | FOSL1//TDRD5// |
| GO:0009629 | response to gravity | Biological process | 2 | 10 | 574 | 17653 | 6.15087108013937 | 0.039950299371896 | 0.371131695950732 | 1.39847996190505 | 0.00348432055749129 | MYOG//FOSL1// |
| GO:0010998 | regulation of translational initiation by eIF2 alpha phosphorylation | Biological process | 2 | 10 | 574 | 17653 | 6.15087108013937 | 0.039950299371896 | 0.371131695950732 | 1.39847996190505 | 0.00348432055749129 | NCK1//PPP1R15B// |
| GO:0032025 | response to cobalt ion | Biological process | 2 | 10 | 574 | 17653 | 6.15087108013937 | 0.039950299371896 | 0.371131695950732 | 1.39847996190505 | 0.00348432055749129 | BNIP3//CASP3// |
| GO:0033327 | Leydig cell differentiation | Biological process | 2 | 10 | 574 | 17653 | 6.15087108013937 | 0.039950299371896 | 0.371131695950732 | 1.39847996190505 | 0.00348432055749129 | AR//MGST1// |
| GO:0046886 | positive regulation of hormone biosynthetic process | Biological process | 2 | 10 | 574 | 17653 | 6.15087108013937 | 0.039950299371896 | 0.371131695950732 | 1.39847996190505 | 0.00348432055749129 | IFNG//TNF// |
| GO:0051256 | mitotic spindle midzone assembly | Biological process | 2 | 10 | 574 | 17653 | 6.15087108013937 | 0.039950299371896 | 0.371131695950732 | 1.39847996190505 | 0.00348432055749129 | KIF4B//CDC14A// |
| GO:0052646 | alditol phosphate metabolic process | Biological process | 2 | 10 | 574 | 17653 | 6.15087108013937 | 0.039950299371896 | 0.371131695950732 | 1.39847996190505 | 0.00348432055749129 | GPD1//GPD2// |
| GO:0060281 | regulation of oocyte development | Biological process | 2 | 10 | 574 | 17653 | 6.15087108013937 | 0.039950299371896 | 0.371131695950732 | 1.39847996190505 | 0.00348432055749129 | IGF1//YTHDF2// |
| GO:0060513 | prostatic bud formation | Biological process | 2 | 10 | 574 | 17653 | 6.15087108013937 | 0.039950299371896 | 0.371131695950732 | 1.39847996190505 | 0.00348432055749129 | AR//SULF1// |
| GO:0061088 | regulation of sequestering of zinc ion | Biological process | 2 | 10 | 574 | 17653 | 6.15087108013937 | 0.039950299371896 | 0.371131695950732 | 1.39847996190505 | 0.00348432055749129 | SLC30A7//SLC30A5// |
| GO:0072578 | neurotransmitter-gated ion channel clustering | Biological process | 2 | 10 | 574 | 17653 | 6.15087108013937 | 0.039950299371896 | 0.371131695950732 | 1.39847996190505 | 0.00348432055749129 | GPHN//GLRB// |
| GO:0098792 | xenophagy | Biological process | 2 | 10 | 574 | 17653 | 6.15087108013937 | 0.039950299371896 | 0.371131695950732 | 1.39847996190505 | 0.00348432055749129 | CALCOCO2//ATG16L1// |
| GO:1902237 | positive regulation of endoplasmic reticulum stress-induced intrinsic apoptotic signaling pathway | Biological process | 2 | 10 | 574 | 17653 | 6.15087108013937 | 0.039950299371896 | 0.371131695950732 | 1.39847996190505 | 0.00348432055749129 | SERINC3//NCK1// |
| GO:1902916 | positive regulation of protein polyubiquitination | Biological process | 2 | 10 | 574 | 17653 | 6.15087108013937 | 0.039950299371896 | 0.371131695950732 | 1.39847996190505 | 0.00348432055749129 | PTPN22//NMI// |
| GO:1903897 | regulation of PERK-mediated unfolded protein response | Biological process | 2 | 10 | 574 | 17653 | 6.15087108013937 | 0.039950299371896 | 0.371131695950732 | 1.39847996190505 | 0.00348432055749129 | NCK1//PPP1R15B// |
| GO:1905879 | regulation of oogenesis | Biological process | 2 | 10 | 574 | 17653 | 6.15087108013937 | 0.039950299371896 | 0.371131695950732 | 1.39847996190505 | 0.00348432055749129 | IGF1//YTHDF2// |
| GO:0003170 | heart valve development | Biological process | 4 | 40 | 574 | 17653 | 3.07543554006969 | 0.0401634128932257 | 0.372116609505169 | 1.39616939004336 | 0.00696864111498258 | TGFBR2//ACVR1//BMPR2//SOX4// |
| GO:0120036 | plasma membrane bounded cell projection organization | Biological process | 59 | 1444 | 574 | 17653 | 1.25658377329717 | 0.0401663647698911 | 0.372116609505169 | 1.3961374720228 | 0.102787456445993 | CD2AP//NCK1//OGN//POU4F1//WNT7A//SLIT2//ARX//DOK1//DPYSL2//EFNA5//OTX2//PIK3CB//ENAH//KLF7//RPS6KA5//NRXN1//DYNLL2//RFX3//RFX4//GMNC//CDC14A//C5ORF30//SNAP25//STMN2//ADCYAP1//NPTN//RAPGEF2//PTPRG//SPOCK1//TSC1//FLRT1//NR4A2//ARHGEF4//UBE4B//CNTNAP2//LRP12//DBNDD2//PSD//FZD3//AKAP4//ULK2//PPP3CB//NYAP1//GJA1//NTF3//HPRT1//FMR1//HECW2//BMPR2//POU3F2//SOD1//CPEB3//IL2//DLG5//LRP8//SNX2//PRKAR2B//IL1RAP//PLAA// |
| GO:0010629 | negative regulation of gene expression | Biological process | 79 | 2005 | 574 | 17653 | 1.21176761927933 | 0.0402264342992364 | 0.372163303920295 | 1.39548846190541 | 0.137630662020906 | TSHZ1//ZBTB18//CEBPB//ZNF268//TCERG1//STRAP//KLF12//ARX//EDN1//CC2D1B//EN1//ESR1//CPEB3//MYT1L//RCOR1//ZFPM2//GATA2//CNOT7//HOXC8//HSBP1//IFNG//IRF2//AR//JARID2//ZNF568//MAF//MDM4//MYB//NFIA//NR4A2//KLF3//POU4F1//PPARG//DUSP22//ZBTB4//RBBP8//TNF//ZNF217//LRP8//BHLHE41//TBL1XR1//ARID5B//BHLHE40//RUNX3//PHF14//DCP2//CNOT6L//PAN3//CNOT6//BAHD1//SNIP1//ELK3//EREG//FOXF2//MDFIC//IRF1//RASD1//RFX3//ATXN1//TSC22D4//BTAF1//RPS6KA5//FMR1//TSC1//ATAD2//MIER1//YTHDF2//DDX6//POLR2K//QKI//MYD88//CPEB2//NCK1//CPEB1//CCR1//PTPN22//GJA1//LDLR//POU3F2// |
| GO:0032147 | activation of protein kinase activity | Biological process | 17 | 328 | 574 | 17653 | 1.59397573723124 | 0.0403269719783311 | 0.372583758865373 | 1.39440438653372 | 0.029616724738676 | GADD45A//TNF//CRKL//MAP3K1//MAP3K7CL//LAMTOR3//IGF1//NTF3//PIK3CB//PRKAA1//SOD1//C5AR1//EFNA5//MDFIC//PRKAR2B//PAK6//TGFBR2// |
| GO:0022612 | gland morphogenesis | Biological process | 8 | 119 | 574 | 17653 | 2.06751969080315 | 0.0404206622835418 | 0.37282848799515 | 1.39339657494414 | 0.0139372822299652 | TGFBR2//LRP6//AR//ESR1//TNF//SULF1//BTBD7//CEBPB// |
| GO:0022414 | reproductive process | Biological process | 58 | 1417 | 574 | 17653 | 1.25882329798195 | 0.0404978159552873 | 0.37282848799515 | 1.39256839762838 | 0.101045296167247 | SPO11//SYCP1//TEX11//CEBPB//ADCYAP1//INHBB//DMRTA1//SOD1//ESR1//EREG//YTHDF2//AGFG1//PPARG//GATA2//BMPR2//SYCE1//MYBL1//SYCP3//ACVR1//DDX6//RAI14//AR//ARID4B//BTG1//SPATA9//ADAM18//TDRD5//STRBP//QKI//FOSL1//TRPC3//AKAP4//CRISP1//GLRB//ZFPM2//WNT7A//SPHK2//TGFBR2//PTGFR//NHLH2//RNF38//ARID5B//CBL//MGST1//NEUROG1//LRP6//GJA1//GJB2//PAQR5//FOXF2//RBBP8//EDN1//IGF1//SYT6//ZNF568//SULF1//EGLN1//SLIT2// |
| GO:0003300 | cardiac muscle hypertrophy | Biological process | 7 | 98 | 574 | 17653 | 2.19673967147835 | 0.0405739711909553 | 0.37282848799515 | 1.39175248349817 | 0.0121951219512195 | MTPN//EDN1//IGF1//JARID2//AGTR2//AKAP6//TRPC3// |
| GO:0042472 | inner ear morphogenesis | Biological process | 7 | 98 | 574 | 17653 | 2.19673967147835 | 0.0405739711909553 | 0.37282848799515 | 1.39175248349817 | 0.0121951219512195 | LRIG1//SOD1//FRZB//NEUROG1//GATA2//FZD3//FZD6// |
| GO:0050808 | synapse organization | Biological process | 16 | 304 | 574 | 17653 | 1.6186502842472 | 0.0407143229753364 | 0.373610537696337 | 1.3902527825467 | 0.0278745644599303 | POU4F1//PCDHB10//NRXN1//GPHN//EFNA5//FLRT1//IL1RAP//WNT7A//DLG5//NFIA//SYBU//LRP8//GLRB//NPTN//FMR1//TSC1// |
| GO:0021700 | developmental maturation | Biological process | 14 | 256 | 574 | 17653 | 1.68187881097561 | 0.0408510148071084 | 0.374356928374626 | 1.38879715040077 | 0.024390243902439 | EREG//YTHDF2//GJA1//TDRD5//IGF1//NR4A2//CNTNAP2//GATA2//PPARG//RFX3//CBFB//NFIA//SYBU//NRXN1// |
| GO:0001570 | vasculogenesis | Biological process | 6 | 78 | 574 | 17653 | 2.36571964620745 | 0.0413121505724836 | 0.375963105970314 | 1.38392219651574 | 0.0104529616724739 | WNT7A//SPRED1//RAPGEF2//ZFPM2//TGFBR2//QKI// |
| GO:0072401 | signal transduction involved in DNA integrity checkpoint | Biological process | 6 | 78 | 574 | 17653 | 2.36571964620745 | 0.0413121505724836 | 0.375963105970314 | 1.38392219651574 | 0.0104529616724739 | GADD45A//CNOT6L//CNOT7//MDM4//CNOT6//SOX4// |
| GO:0072422 | signal transduction involved in DNA damage checkpoint | Biological process | 6 | 78 | 574 | 17653 | 2.36571964620745 | 0.0413121505724836 | 0.375963105970314 | 1.38392219651574 | 0.0104529616724739 | GADD45A//CNOT6L//CNOT7//MDM4//CNOT6//SOX4// |
| GO:0002068 | glandular epithelial cell development | Biological process | 3 | 24 | 574 | 17653 | 3.84429442508711 | 0.0416933800795114 | 0.375963105970314 | 1.3799328952221 | 0.00522648083623693 | CDK6//DLL1//RFX3// |
| GO:0003309 | type B pancreatic cell differentiation | Biological process | 3 | 24 | 574 | 17653 | 3.84429442508711 | 0.0416933800795114 | 0.375963105970314 | 1.3799328952221 | 0.00522648083623693 | CDK6//DLL1//RFX3// |
| GO:0006688 | glycosphingolipid biosynthetic process | Biological process | 3 | 24 | 574 | 17653 | 3.84429442508711 | 0.0416933800795114 | 0.375963105970314 | 1.3799328952221 | 0.00522648083623693 | ST8SIA3//PRKAA1//ST8SIA5// |
| GO:0010738 | regulation of protein kinase A signaling | Biological process | 3 | 24 | 574 | 17653 | 3.84429442508711 | 0.0416933800795114 | 0.375963105970314 | 1.3799328952221 | 0.00522648083623693 | AKAP11//AKAP4//AKAP6// |
| GO:0010996 | response to auditory stimulus | Biological process | 3 | 24 | 574 | 17653 | 3.84429442508711 | 0.0416933800795114 | 0.375963105970314 | 1.3799328952221 | 0.00522648083623693 | NEUROG1//CNTNAP2//NRXN1// |
| GO:0021591 | ventricular system development | Biological process | 3 | 24 | 574 | 17653 | 3.84429442508711 | 0.0416933800795114 | 0.375963105970314 | 1.3799328952221 | 0.00522648083623693 | CDK6//ANP32B//RAPGEF2// |
| GO:0032800 | receptor biosynthetic process | Biological process | 3 | 24 | 574 | 17653 | 3.84429442508711 | 0.0416933800795114 | 0.375963105970314 | 1.3799328952221 | 0.00522648083623693 | EDN1//PPARG//TNF// |
| GO:0060444 | branching involved in mammary gland duct morphogenesis | Biological process | 3 | 24 | 574 | 17653 | 3.84429442508711 | 0.0416933800795114 | 0.375963105970314 | 1.3799328952221 | 0.00522648083623693 | AR//ESR1//LRP6// |
| GO:0060969 | negative regulation of gene silencing | Biological process | 3 | 24 | 574 | 17653 | 3.84429442508711 | 0.0416933800795114 | 0.375963105970314 | 1.3799328952221 | 0.00522648083623693 | ATAD2//PPARG//ESR1// |
| GO:0000003 | reproduction | Biological process | 58 | 1420 | 574 | 17653 | 1.25616381214114 | 0.0418941606329275 | 0.377270583702382 | 1.37784650642817 | 0.101045296167247 | SPO11//SYCP1//TEX11//CEBPB//ADCYAP1//INHBB//DMRTA1//SOD1//ESR1//EREG//YTHDF2//AGFG1//PPARG//GATA2//BMPR2//SYCE1//MYBL1//SYCP3//ACVR1//DDX6//RAI14//AR//ARID4B//BTG1//SPATA9//ADAM18//TDRD5//STRBP//QKI//FOSL1//TRPC3//AKAP4//CRISP1//GLRB//ZFPM2//WNT7A//SPHK2//TGFBR2//PTGFR//NHLH2//RNF38//ARID5B//CBL//MGST1//NEUROG1//LRP6//GJA1//GJB2//PAQR5//FOXF2//RBBP8//EDN1//IGF1//SYT6//ZNF568//SULF1//EGLN1//SLIT2// |
| GO:0032635 | interleukin-6 production | Biological process | 8 | 120 | 574 | 17653 | 2.05029036004646 | 0.042132869046934 | 0.378915682665445 | 1.37537896648846 | 0.0139372822299652 | TNF//SOCS5//ADCYAP1//MYD88//CEBPB//EREG//PTPN22//IL1RAP// |
| GO:0030098 | lymphocyte differentiation | Biological process | 17 | 330 | 574 | 17653 | 1.58431527821772 | 0.0422936642063155 | 0.379396117283825 | 1.37372468714805 | 0.029616724738676 | GPR183//DLL1//ITM2A//IFNA1//PTPN22//IL2//PPP3CB//SOX4//IFNG//SOD1//IRF1//TSC1//CD8A//CBFB//MYB//SOCS5//TGFBR2// |
| GO:0002791 | regulation of peptide secretion | Biological process | 22 | 455 | 574 | 17653 | 1.48702377761611 | 0.0422984877172858 | 0.379396117283825 | 1.37367515952279 | 0.0383275261324042 | TNF//RAPGEF4//IFNG//NEUROD1//PPP3CB//RFX3//SNAP25//GJA1//SOX4//SYBU//INHBB//ARFIP1//IGF1//AGTR2//IL2//IL5//ADCYAP1//EFNA5//NRXN1//AIMP1//PTPN22//IL1RAP// |
| GO:0030534 | adult behavior | Biological process | 9 | 142 | 574 | 17653 | 1.94921970849487 | 0.0424597412861016 | 0.379397255103065 | 1.37202265638671 | 0.0156794425087108 | GLRB//KCNJ10//EN1//NR4A2//TMOD1//TSC1//CNTNAP2//RNF180//NRXN1// |
| GO:0032387 | negative regulation of intracellular transport | Biological process | 5 | 59 | 574 | 17653 | 2.6063013051438 | 0.0424669114465504 | 0.379397255103065 | 1.37194932343352 | 0.00871080139372822 | MDFIC//CD84//SOX4//FMR1//ARHGAP1// |
| GO:0060135 | maternal process involved in female pregnancy | Biological process | 5 | 59 | 574 | 17653 | 2.6063013051438 | 0.0424669114465504 | 0.379397255103065 | 1.37194932343352 | 0.00871080139372822 | BMPR2//GJA1//GJB2//ESR1//AR// |
| GO:0048732 | gland development | Biological process | 21 | 430 | 574 | 17653 | 1.50195689166194 | 0.0425703867071337 | 0.379819954222091 | 1.37089240469692 | 0.0365853658536585 | AK4//JARID2//MET//ARID5B//ADCYAP1//GATA2//POU3F2//AR//HOXA3//PTF1A//CEBPB//CRKL//SOD1//GJA1//TGFBR2//LRP6//ESR1//TNF//SULF1//BTBD7//FRZB// |
| GO:0072358 | cardiovascular system development | Biological process | 34 | 769 | 574 | 17653 | 1.35975043384095 | 0.043347361627616 | 0.384861792924771 | 1.36303733106984 | 0.0592334494773519 | ELK3//EREG//HOXA3//MEOX2//CCL2//VEGFB//WNT7A//MMRN2//AIMP1//CRKL//SPHK2//TGFBR2//RAPGEF2//EDN1//ACVR1//ZFPM2//QKI//ANP32B//PPP3CB//BMPR2//SLIT2//SPRED1//SULF1//PPARG//SOX4//EGLN1//GATA2//BTG1//C5AR1//ADAM12//GJA1//PIK3CB//FOSL1//DLL1// |
| GO:0006654 | phosphatidic acid biosynthetic process | Biological process | 4 | 41 | 574 | 17653 | 3.00042491714116 | 0.0434041799841664 | 0.384861792924771 | 1.36246844429756 | 0.00696864111498258 | DDHD2//LCLAT1//GPD1//PLA2G12A// |
| GO:0030204 | chondroitin sulfate metabolic process | Biological process | 4 | 41 | 574 | 17653 | 3.00042491714116 | 0.0434041799841664 | 0.384861792924771 | 1.36246844429756 | 0.00696864111498258 | SLC35D1//CHST7//IDS//DSEL// |
| GO:0048469 | cell maturation | Biological process | 10 | 165 | 574 | 17653 | 1.86390032731496 | 0.0434633626657325 | 0.384861792924771 | 1.3618766762972 | 0.0174216027874564 | EREG//YTHDF2//GJA1//TDRD5//NR4A2//CNTNAP2//RFX3//GATA2//PPARG//CBFB// |
| GO:0008344 | adult locomotory behavior | Biological process | 6 | 79 | 574 | 17653 | 2.33577382790103 | 0.0435338269388511 | 0.384861792924771 | 1.36117315353243 | 0.0104529616724739 | GLRB//KCNJ10//EN1//NR4A2//TMOD1//TSC1// |
| GO:0071674 | mononuclear cell migration | Biological process | 6 | 79 | 574 | 17653 | 2.33577382790103 | 0.0435338269388511 | 0.384861792924771 | 1.36117315353243 | 0.0104529616724739 | CCL2//CCL8//C5AR1//SLIT2//TNF//CCR1// |
| GO:0072395 | signal transduction involved in cell cycle checkpoint | Biological process | 6 | 79 | 574 | 17653 | 2.33577382790103 | 0.0435338269388511 | 0.384861792924771 | 1.36117315353243 | 0.0104529616724739 | GADD45A//CNOT6L//CNOT7//MDM4//CNOT6//SOX4// |
| GO:0003231 | cardiac ventricle development | Biological process | 8 | 121 | 574 | 17653 | 2.03334581161632 | 0.0438924442694534 | 0.387525588243229 | 1.35761023361896 | 0.0139372822299652 | TGFBR2//SOX4//ZFPM2//UBE4B//POU4F1//EGLN1//BMPR2//ACVR1// |
| GO:0048285 | organelle fission | Biological process | 22 | 457 | 574 | 17653 | 1.4805160149132 | 0.0440113959378135 | 0.38756643490961 | 1.35643485642058 | 0.0383275261324042 | TEX11//CDC27//CDC14A//SYCP1//SPO11//SYCE1//MYBL1//SYCP3//EREG//CLASP2//HECW2//NEUROG1//MTBP//EDN1//IGF1//BTC//KIF4B//MIEF2//DDHD2//BNIP3//MZT1//TNKS// |
| GO:0048660 | regulation of smooth muscle cell proliferation | Biological process | 9 | 143 | 574 | 17653 | 1.93558880144246 | 0.0440689913419326 | 0.38756643490961 | 1.35586688957735 | 0.0156794425087108 | EDN1//EREG//IGF1//MYB//TGFBR2//TNF//IFNG//OGN//PPARG// |
| GO:0051384 | response to glucocorticoid | Biological process | 9 | 143 | 574 | 17653 | 1.93558880144246 | 0.0440689913419326 | 0.38756643490961 | 1.35586688957735 | 0.0156794425087108 | FOSL1//SLIT2//ADCYAP1//EDN1//FIBIN//AGTR2//GJB2//TNF//CASP3// |
| GO:0014897 | striated muscle hypertrophy | Biological process | 7 | 100 | 574 | 17653 | 2.15280487804878 | 0.0444704391448308 | 0.390589064852585 | 1.35192858187926 | 0.0121951219512195 | MTPN//EDN1//IGF1//JARID2//AGTR2//AKAP6//TRPC3// |
| GO:0045732 | positive regulation of protein catabolic process | Biological process | 12 | 212 | 574 | 17653 | 1.74081256985077 | 0.0447992451982253 | 0.392457636367355 | 1.3487293031672 | 0.0209059233449477 | RNF180//RNF125//LONRF3//LONRF1//SOCS5//SH3D19//IFNG//TNF//FMR1//LDLR//GJA1//HECW2// |
| GO:0070663 | regulation of leukocyte proliferation | Biological process | 12 | 212 | 574 | 17653 | 1.74081256985077 | 0.0447992451982253 | 0.392457636367355 | 1.3487293031672 | 0.0209059233449477 | CASP3//GPR183//IL2//IL5//PTPN22//NCK1//IGF1//CEBPB//DLG5//CCL8//TGFBR2//IRF1// |
| GO:0001505 | regulation of neurotransmitter levels | Biological process | 16 | 308 | 574 | 17653 | 1.59762885198425 | 0.0449182309729103 | 0.392933006077955 | 1.34757735601325 | 0.0278745644599303 | SNAP25//NOS1//ARL6IP1//WNT7A//NRXN1//SLC44A1//RNF180//AGTR2//EDN1//IFNG//TNF//FMR1//SYT6//KCNJ10//RAB3GAP1//SLC1A1// |
| GO:0062013 | positive regulation of small molecule metabolic process | Biological process | 10 | 166 | 574 | 17653 | 1.85267201209017 | 0.0449697097004787 | 0.392933006077955 | 1.34707991598748 | 0.0174216027874564 | CALM2//NOS1//PRKAA1//MID1IP1//IGF1//GPD1//PPARG//IFNG//TNF//MYOG// |
| GO:0015800 | acidic amino acid transport | Biological process | 5 | 60 | 574 | 17653 | 2.56286295005807 | 0.0451412749135061 | 0.393923151277473 | 1.34542617941684 | 0.00871080139372822 | ARL6IP1//SLC1A1//SNAP25//KCNJ10//RAB3GAP1// |
| GO:0042063 | gliogenesis | Biological process | 14 | 260 | 574 | 17653 | 1.65600375234522 | 0.04548828501852 | 0.396439782964241 | 1.34210043646161 | 0.024390243902439 | CDK6//DLL1//POU3F2//SOX4//KCNJ10//SOD1//APCDD1//CCL2//BNIP3//PPARG//MYB//ADCYAP1//LDLR//GPR183// |
| GO:0003148 | outflow tract septum morphogenesis | Biological process | 3 | 25 | 574 | 17653 | 3.69052264808362 | 0.0462704728570971 | 0.399565029160248 | 1.33469606224251 | 0.00522648083623693 | ZFPM2//BMPR2//TGFBR2// |
| GO:0009299 | mRNA transcription | Biological process | 3 | 25 | 574 | 17653 | 3.69052264808362 | 0.0462704728570971 | 0.399565029160248 | 1.33469606224251 | 0.00522648083623693 | MYOG//C5AR1//EREG// |
| GO:0043372 | positive regulation of CD4-positive, alpha-beta T cell differentiation | Biological process | 3 | 25 | 574 | 17653 | 3.69052264808362 | 0.0462704728570971 | 0.399565029160248 | 1.33469606224251 | 0.00522648083623693 | IFNG//MYB//SOCS5// |
| GO:0046885 | regulation of hormone biosynthetic process | Biological process | 3 | 25 | 574 | 17653 | 3.69052264808362 | 0.0462704728570971 | 0.399565029160248 | 1.33469606224251 | 0.00522648083623693 | IFNG//TNF//TRERF1// |
| GO:0060384 | innervation | Biological process | 3 | 25 | 574 | 17653 | 3.69052264808362 | 0.0462704728570971 | 0.399565029160248 | 1.33469606224251 | 0.00522648083623693 | SULF1//LRIG1//POU4F1// |
| GO:0071514 | genetic imprinting | Biological process | 3 | 25 | 574 | 17653 | 3.69052264808362 | 0.0462704728570971 | 0.399565029160248 | 1.33469606224251 | 0.00522648083623693 | ARID4B//DIRAS3//PCGF5// |
| GO:0044087 | regulation of cellular component biogenesis | Biological process | 38 | 881 | 574 | 17653 | 1.32652157233426 | 0.0465654152694297 | 0.399565029160248 | 1.3319365196624 | 0.0662020905923345 | SLIT2//CNOT6L//PAN3//CNOT6//PMEPA1//LDLRAD4//NCK1//STMN2//MET//CLIP1//IFNG//TNF//MIEF2//IQGAP2//ARFIP1//ESR1//PRKAA1//FMR1//TSC1//CLASP2//TMOD1//EFNA5//FLRT1//IL1RAP//WNT7A//DLG5//NRXN1//EDN1//FAM110C//IL5//RAPGEF2//MTM1//ATR//FOSL1//RAB3GAP1//GJA1//CBFB//MTPN// |
| GO:0031668 | cellular response to extracellular stimulus | Biological process | 13 | 237 | 574 | 17653 | 1.68694776459519 | 0.0466215066013835 | 0.399565029160248 | 1.33141369610727 | 0.0226480836236934 | DSC2//INHBB//RRAGD//BMPR2//PLEKHF2//PRKAA1//DCTPP1//PPARG//SOD1//TSC1//CBL//NR4A2//FOSL1// |
| GO:0015711 | organic anion transport | Biological process | 22 | 460 | 574 | 17653 | 1.4708604756855 | 0.046674908096134 | 0.399565029160248 | 1.33091652851292 | 0.0383275261324042 | ARL6IP1//SLC6A14//SLC6A6//SLC1A1//MID1IP1//SLC17A5//SNAP25//CA1//SLC4A4//SLC10A2//SLC35D1//SERINC3//GJA1//PPARG//TNFAIP8L3//LDLR//AGTR2//EDN1//ACSL1//PLA2G12A//KCNJ10//RAB3GAP1// |
| GO:0019538 | protein metabolic process | Biological process | 217 | 6078 | 574 | 17653 | 1.09800841098243 | 0.0466880329580676 | 0.399565029160248 | 1.3307944232089 | 0.378048780487805 | GADD45A//DIRAS3//CSF2RA//SPRED1//EREG//RASGEF1A//IL2//IL5//MAP3K1//MET//MAP3K7CL//CCL2//BTC//TNF//LAMTOR3//RAPGEF2//CRKL//IGF1//NTF3//PIK3CB//PRKAA1//SOD1//C5AR1//DUSP22//UBE4B//RNF145//ZNRF2//RNF180//RNF125//UBE2W//HECW2//SKP1//THOP1//UBE2G1//LONRF3//CBFB//TNKS//CBL//UBE3B//LONRF1//SECISBP2L//LRP6//PPP1R15B//SLIT2//EFNA5//FMR1//NPTN//IFNG//SOCS5//RNF38//KLHL3//SPOPL//MDM4//DCAF8//TRIM8//KBTBD8//CPEB2//CPEB3//MRPL35//CPEB1//METTL17//EIF3B//MTPN//DDX6//TSC1//AIMP1//YES1//IMMP1L//CDK6//FASTK//PDIK1L//CDK19//PAN3//PPP3CB//CCL8//TGFBR2//RUNX3//ACVR1//RPS6KA5//FLRT1//NCK1//DBNDD2//PTPN22//MTM1//CPPED1//HS3ST5//BTG1//FUT9//FUT2//ST8SIA5//MAN1A1//ST8SIA3//CCDC126//TET2//VEGFB//PIGP//PIGA//GGT6//PRSS55//MEP1A//DESI2//PGC//NLN//LRP8//NPEPL1//ADAM12//CASP3//ADAM18//LMLN//USP28//GPHN//ANP32B//EGLN3//PPARG//VSIG4//DLL1//MDFIC//CALM2//INHBB//BMP3//BMPR2//A2ML1//WFDC13//SPOCK1//NDST3//PRR16//SOX4//PIGS//GNPTG//EPC2//ARID4B//MIER1//TBL1XR1//ARID5B//ESR1//OTUD3//AR//WDR20//NFATC2IP//CNOT7//VKORC1L1//ATR//STK33//NOS1//EGLN1//DCUN1D4//PAK6//MSRB3//SULF1//DSEL//SLC35D1//CHST7//IDS//JARID2//CDC27//RAP2C//EDN1//WNT7A//PHACTR2//AGTR2//AKAP6//IFNA1//PRKAR2B//ESCO2//GATA2//CNOT6//PTPRG//PTP4A1//CDC14A//BNIP3//PCGF5//IL1RAP//LDLR//ITM2A//ARL6IP1//UBXN2B//CD2AP//PLAA//VPS37A//ARSK//RAB12//IRF1//NMI//CEBPB//GJA1//CCNY//ADCYAP1//GRIK2//ZMYND11//ULK2//SAMSN1//SH3D19//ARRDC3//MYB//STRAP//PMEPA1//LDLRAD4//POLR2K//CNOT6L//YTHDF2//QKI//ATG16L1//CCR1//GPR183//NPNT//TNFAIP8L3//KDM2A//RFX4//RCOR1//UBA5//ACSL1//GLRX//TMED10//NCOA7//RAB3GAP1//ST18// |
| GO:0043949 | regulation of cAMP-mediated signaling | Biological process | 4 | 42 | 574 | 17653 | 2.9289862286378 | 0.0467876315532465 | 0.399565029160248 | 1.32986893876571 | 0.00696864111498258 | ADCYAP1//RAPGEF2//NOS1//PEX5L// |
| GO:0045429 | positive regulation of nitric oxide biosynthetic process | Biological process | 4 | 42 | 574 | 17653 | 2.9289862286378 | 0.0467876315532465 | 0.399565029160248 | 1.32986893876571 | 0.00696864111498258 | AGTR2//EDN1//IFNG//TNF// |
| GO:1904407 | positive regulation of nitric oxide metabolic process | Biological process | 4 | 42 | 574 | 17653 | 2.9289862286378 | 0.0467876315532465 | 0.399565029160248 | 1.32986893876571 | 0.00696864111498258 | AGTR2//EDN1//IFNG//TNF// |
| GO:0044344 | cellular response to fibroblast growth factor stimulus | Biological process | 9 | 145 | 574 | 17653 | 1.90889102487084 | 0.047408884103096 | 0.399565029160248 | 1.32414026686141 | 0.0156794425087108 | SPRED1//FLRT1//POLR2K//CBL//SULF1//OTX2//SHISA2//NPTN//CCL2// |
| GO:0006950 | response to stress | Biological process | 146 | 3965 | 574 | 17653 | 1.13244284703701 | 0.0474096979482612 | 0.399565029160248 | 1.32413281160408 | 0.254355400696864 | INTS7//ATR//USP28//SOD1//SPO11//SYCP1//RBBP8//NABP1//WRNIP1//ADCYAP1//GRIK2//EGLN3//MYB//NOS1//NR4A2//EGLN1//PRKAA1//BNIP3//TGFBR2//VEGFB//CASP3//SKP1//RPS6KA5//MYD88//TNF//ACVR1//GJA1//PPARG//REG3G//MAP3K1//PGC//GJB2//DNAJB4//INHBB//CAMLG//CD84//CEBPB//PROCR//ENPP4//MMRN1//RCOR1//ZFPM2//GATA2//IFNA1//IRF1//IRF2//NFE2//PRKAR2B//RAD9B//GADD45A//EPC2//UBE2W//UNG//POLR2K//ESCO2//KDM2A//CCR1//AGTR2//IL1RAP//IL5//PIK3CB//PXK//IL36G//PTGFR//CCL2//CCL8//C5AR1//CHST1//NMI//AIMP1//PLAA//VSIG4//FOSL1//FMR1//ZBTB4//ZMAT3//CBL//CNOT6L//CNOT7//MDM4//CNOT6//SOX4//MSRB3//BTG1//PPP1R15B//CRKL//MDFIC//RAET1E//BCL2A1//DSC2//RRAGD//BMPR2//PLEKHF2//IGF1//EDN1//WNT7A//DGKH//TRPC3//FZD6//DGKE//UBE4B//PAK6//ESR1//PTPN22//CALCOCO2//CPEB2//VKORC1L1//CETN1//TFEC//NRBF2//UBA5//NCK1//RNF125//ELK3//EREG//DCTPP1//ULK2//MTPN//MYOG//SPRED1//SERINC3//TREM1//YES1//TRIM8//ZMYND11//DUSP22//IL2//SOCS5//CDK19//LDLR//IFNG//XAF1//HSBP1//GSTM3//RAB12//MGST1//CPEB1//ATG16L1//C5ORF30//PTGES3//HSPB8//MET//NCOA7//TSC1//CLASP2//CDK6// |
| GO:0007159 | leukocyte cell-cell adhesion | Biological process | 17 | 335 | 574 | 17653 | 1.5606687815279 | 0.0475105617373475 | 0.399565029160248 | 1.32320983470418 | 0.029616724738676 | YES1//IFNG//IL2//NCK1//IGF1//CEBPB//DLG5//IRF1//MYB//SOCS5//CASP3//PTPN22//DUSP22//CCL2//TNF//TGFBR2//CCL28// |
| GO:0050804 | modulation of chemical synaptic transmission | Biological process | 17 | 335 | 574 | 17653 | 1.5606687815279 | 0.0475105617373475 | 0.399565029160248 | 1.32320983470418 | 0.029616724738676 | SNAP25//EDN1//FMR1//CPEB3//PPP3CB//RAPGEF2//GRIK2//KCNJ10//NPTN//RAB3GAP1//ADCYAP1//NRXN1//SYBU//WNT7A//NTF3//PXK//LRP8// |
| GO:0099177 | regulation of trans-synaptic signaling | Biological process | 17 | 335 | 574 | 17653 | 1.5606687815279 | 0.0475105617373475 | 0.399565029160248 | 1.32320983470418 | 0.029616724738676 | SNAP25//EDN1//FMR1//CPEB3//PPP3CB//RAPGEF2//GRIK2//KCNJ10//NPTN//RAB3GAP1//NTF3//PXK//LRP8//ADCYAP1//NRXN1//SYBU//WNT7A// |
| GO:0001821 | histamine secretion | Biological process | 2 | 11 | 574 | 17653 | 5.59170098194488 | 0.0477965560536213 | 0.399565029160248 | 1.32060339503626 | 0.00348432055749129 | ADCYAP1//EDN1// |
| GO:0002182 | cytoplasmic translational elongation | Biological process | 2 | 11 | 574 | 17653 | 5.59170098194488 | 0.0477965560536213 | 0.399565029160248 | 1.32060339503626 | 0.00348432055749129 | CPEB2//CPEB3// |
| GO:0016264 | gap junction assembly | Biological process | 2 | 11 | 574 | 17653 | 5.59170098194488 | 0.0477965560536213 | 0.399565029160248 | 1.32060339503626 | 0.00348432055749129 | GJA1//GJB2// |
| GO:0031987 | locomotion involved in locomotory behavior | Biological process | 2 | 11 | 574 | 17653 | 5.59170098194488 | 0.0477965560536213 | 0.399565029160248 | 1.32060339503626 | 0.00348432055749129 | ARRDC3//PPP3CB// |
| GO:0042178 | xenobiotic catabolic process | Biological process | 2 | 11 | 574 | 17653 | 5.59170098194488 | 0.0477965560536213 | 0.399565029160248 | 1.32060339503626 | 0.00348432055749129 | ACSL1//GSTM3// |
| GO:0042368 | vitamin D biosynthetic process | Biological process | 2 | 11 | 574 | 17653 | 5.59170098194488 | 0.0477965560536213 | 0.399565029160248 | 1.32060339503626 | 0.00348432055749129 | IFNG//TNF// |
| GO:0045793 | positive regulation of cell size | Biological process | 2 | 11 | 574 | 17653 | 5.59170098194488 | 0.0477965560536213 | 0.399565029160248 | 1.32060339503626 | 0.00348432055749129 | EDN1//PRR16// |
| GO:0051024 | positive regulation of immunoglobulin secretion | Biological process | 2 | 11 | 574 | 17653 | 5.59170098194488 | 0.0477965560536213 | 0.399565029160248 | 1.32060339503626 | 0.00348432055749129 | IL2//IL5// |
| GO:0060099 | regulation of phagocytosis, engulfment | Biological process | 2 | 11 | 574 | 17653 | 5.59170098194488 | 0.0477965560536213 | 0.399565029160248 | 1.32060339503626 | 0.00348432055749129 | GATA2//PPARG// |
| GO:0060211 | regulation of nuclear-transcribed mRNA poly(A) tail shortening | Biological process | 2 | 11 | 574 | 17653 | 5.59170098194488 | 0.0477965560536213 | 0.399565029160248 | 1.32060339503626 | 0.00348432055749129 | CPEB3//CNOT7// |
| GO:0060213 | positive regulation of nuclear-transcribed mRNA poly(A) tail shortening | Biological process | 2 | 11 | 574 | 17653 | 5.59170098194488 | 0.0477965560536213 | 0.399565029160248 | 1.32060339503626 | 0.00348432055749129 | CPEB3//CNOT7// |
| GO:0060439 | trachea morphogenesis | Biological process | 2 | 11 | 574 | 17653 | 5.59170098194488 | 0.0477965560536213 | 0.399565029160248 | 1.32060339503626 | 0.00348432055749129 | TGFBR2//LRP6// |
| GO:0071803 | positive regulation of podosome assembly | Biological process | 2 | 11 | 574 | 17653 | 5.59170098194488 | 0.0477965560536213 | 0.399565029160248 | 1.32060339503626 | 0.00348432055749129 | IL5//TNF// |
| GO:0090009 | primitive streak formation | Biological process | 2 | 11 | 574 | 17653 | 5.59170098194488 | 0.0477965560536213 | 0.399565029160248 | 1.32060339503626 | 0.00348432055749129 | LRP6//OTX2// |
| GO:1900247 | regulation of cytoplasmic translational elongation | Biological process | 2 | 11 | 574 | 17653 | 5.59170098194488 | 0.0477965560536213 | 0.399565029160248 | 1.32060339503626 | 0.00348432055749129 | CPEB2//CPEB3// |
| GO:0046622 | positive regulation of organ growth | Biological process | 5 | 61 | 574 | 17653 | 2.52084880333581 | 0.0479139907457843 | 0.399706953180305 | 1.3195376553432 | 0.00871080139372822 | ZFPM2//EDN1//IGF1//AKAP6//ARX// |
| GO:0002573 | myeloid leukocyte differentiation | Biological process | 11 | 191 | 574 | 17653 | 1.77119324297207 | 0.0480499412887162 | 0.399706953180305 | 1.31830713865105 | 0.0191637630662021 | PPARG//GPR183//TNF//GATA2//TGFBR2//IL5//CDK6//CEBPB//CCR1//IFNG//POU4F1// |
| GO:0010810 | regulation of cell-substrate adhesion | Biological process | 11 | 191 | 574 | 17653 | 1.77119324297207 | 0.0480499412887162 | 0.399706953180305 | 1.31830713865105 | 0.0191637630662021 | PIK3CB//TSC1//CDK6//CCL28//NPNT//VWC2//SMOC2//SPOCK1//EFNA5//CLASP2//CRKL// |
| GO:0034341 | response to interferon-gamma | Biological process | 11 | 191 | 574 | 17653 | 1.77119324297207 | 0.0480499412887162 | 0.399706953180305 | 1.31830713865105 | 0.0191637630662021 | IFNG//IRF1//IRF2//TRIM8//NMI//PPARG//EDN1//RAB12//CCL2//CCL8//CALCOCO2// |
| GO:0070542 | response to fatty acid | Biological process | 6 | 81 | 574 | 17653 | 2.27810040005162 | 0.0481979707162661 | 0.400445793555415 | 1.31697124651801 | 0.0104529616724739 | ACSL1//EDN1//PPARG//PRKAA1//LDLR//PTGFR// |
| GO:2000146 | negative regulation of cell motility | Biological process | 16 | 311 | 574 | 17653 | 1.58221764119341 | 0.0482674681390747 | 0.400531149723389 | 1.31634548141887 | 0.0278745644599303 | SLIT2//STRAP//PTPRG//C5ORF30//SULF1//RAP2C//LDLRAD4//DLG5//AGTR2//PPARG//SPRED1//MEOX2//MMRN2//CCL28//CLASP2//CCL2// |
| GO:0014896 | muscle hypertrophy | Biological process | 7 | 102 | 574 | 17653 | 2.11059301769488 | 0.0485999038460506 | 0.402302508825998 | 1.31336458997991 | 0.0121951219512195 | MTPN//EDN1//IGF1//JARID2//AGTR2//AKAP6//TRPC3// |
| GO:1900180 | regulation of protein localization to nucleus | Biological process | 7 | 102 | 574 | 17653 | 2.11059301769488 | 0.0485999038460506 | 0.402302508825998 | 1.31336458997991 | 0.0121951219512195 | AGTR2//IFNG//IGF1//MDFIC//CD2AP//TRIM8//CCT6A// |
| GO:0008654 | phospholipid biosynthetic process | Biological process | 15 | 287 | 574 | 17653 | 1.60737049132562 | 0.0488488526796687 | 0.403868937252567 | 1.31114563216507 | 0.0261324041811847 | PIGP//PIGA//DDHD2//LCLAT1//GPD1//PLA2G12A//CDS1//ABHD3//SLC44A1//MTM1//PIK3CB//MTMR9//SPHK2//PIGS//DGKE// |
| GO:0048659 | smooth muscle cell proliferation | Biological process | 9 | 146 | 574 | 17653 | 1.89581642881008 | 0.0491401374287756 | 0.405781134836153 | 1.30856363327565 | 0.0156794425087108 | EDN1//EREG//IGF1//MYB//TGFBR2//TNF//IFNG//OGN//PPARG// |
| GO:0070374 | positive regulation of ERK1 and ERK2 cascade | Biological process | 13 | 239 | 574 | 17653 | 1.67283104689983 | 0.0492509562429183 | 0.406200264720557 | 1.3075853329432 | 0.0226480836236934 | ADCYAP1//CCR1//GPR183//NPNT//PTPN22//NPTN//TNFAIP8L3//MET//CCL2//CCL8//TNF//C5AR1//RAPGEF2// |
| GO:0003206 | cardiac chamber morphogenesis | Biological process | 8 | 124 | 574 | 17653 | 1.98415196133528 | 0.0494590811990698 | 0.406924289719354 | 1.30575395578468 | 0.0139372822299652 | ZFPM2//BMPR2//TGFBR2//SOX4//UBE4B//POU4F1//ACVR1//EGLN1// |
| GO:0045995 | regulation of embryonic development | Biological process | 8 | 124 | 574 | 17653 | 1.98415196133528 | 0.0494590811990698 | 0.406924289719354 | 1.30575395578468 | 0.0139372822299652 | CLASP2//GATA2//DLL1//OTX2//FZD3//AR//SULF1//IGF1// |
| GO:0097190 | apoptotic signaling pathway | Biological process | 27 | 593 | 574 | 17653 | 1.4002826236405 | 0.0498425225363238 | 0.409341168059631 | 1.30239998609441 | 0.0470383275261324 | BCL2A1//TNF//UBE4B//BNIP3//CASP3//USP28//CEBPB//POU4F1//ZMAT3//IGF1//SIVA1//IFNG//APPL1//IL2//DYNLL2//SOD1//SERINC3//NCK1//NR4A2//INHBB//ZMYND11//AR//ACVR1//AGTR2//IL20RA//ST18//FASTK// |
| GO:0022402 | cell cycle process | Biological process | 57 | 1408 | 574 | 17653 | 1.24502717176117 | 0.0499072676653159 | 0.409341168059631 | 1.30183620633822 | 0.0993031358885017 | CDK6//RBBP8//ACVR1//NPAT//RRM2//CCNY//PRKAR2B//SKP1//KIF4B//SPO11//SYCP1//TEX11//PAK6//GADD45A//CNOT6L//CNOT7//MDM4//CNOT6//SOX4//HBP1//MTBP//IFNG//IRF1//PRKAA1//RRAGD//TSC1//LAMTOR3//CLASP2//TNKS//DYNLL2//DYNC1LI2//CLIP1//CENPO//CDC27//NABP1//CDC14A//HEPACAM2//SYCE1//MYBL1//SYCP3//EREG//CHMP3//HECW2//NEUROG1//RAD9B//KIF13A//CALM2//EDN1//IGF1//BTC//NEUROD1//ZNF268//MYOG//POU4F1//MZT1//YTHDF2//ZNF655// |
| GO:0048667 | cell morphogenesis involved in neuron differentiation | Biological process | 26 | 567 | 574 | 17653 | 1.41025262860338 | 0.049957231904519 | 0.409341168059631 | 1.30140163358649 | 0.0452961672473868 | OGN//POU4F1//WNT7A//SLIT2//ARX//DOK1//DPYSL2//EFNA5//OTX2//PIK3CB//ENAH//KLF7//RPS6KA5//NRXN1//NR4A2//FZD3//ULK2//PPP3CB//HPRT1//FMR1//HECW2//BMPR2//POU3F2//RAPGEF2//SOD1//LRP8// |
